# Discovery of structurally diverse sesquiterpenoids from *Streptomyces fulvorobeus* isolated from *Elephas maximus* feces and their antifungal activities

**DOI:** 10.1007/s13659-024-00481-9

**Published:** 2024-12-02

**Authors:** Lu Cao, Jun-Feng Tan, Zeng-Guang Zhang, Jun-Wei Yang, Yu Mu, Zhi-Long Zhao, Yi Jiang, Xue-Shi Huang, Li Han

**Affiliations:** 1 Institute of Microbial Pharmaceuticals, College of Life and Health Sciences, Northeastern University, Shenyang, 110819 China; 2 Pharmacological Laboratory, Liaoning Provincial Institute of Drug Inspection and Testing, Shenyang, 110036 China; 3 Yunnan Institute of Microbiology, Yunnan University, Kunming, 650091 China

**Keywords:** Sesquiterpenoids, *Streptomyces fulvorobeus*, Fermentation, Antifungal activity

## Abstract

Thirty-six structurally diverse sesquiterpenoids, including caryolanes (**1–12**), germacranes (**13–16**), isodaucane (**17**), cadinanes (**18–22**), epicubenols (**23**, **24**), oplopanane (**25**), pallenanes (**26**, **27**), and eudesmanes (**28–36**), were isolated from the fermentation broth of *Streptomyces fulvorobeus* derived from *Elephas maximus* feces. Pallenane is a kind of rarely reported sesquiterpene with a distinctive C5/C3 bicyclic skeleton and was firstly found from microbial source. The structures of fifteen new compounds (**1–4**, **13–15**, **17**, **18**, **22**, **23**, **25–28**) were established through detailed spectroscopic data analysis, which included data from experimental and calculated ECD spectra as well as Mosher’s reagent derivative method. Compound **34** exhibited moderate antifungal activity against *Cryptococcus neoformans* and *Cryptococcus gattii* with MIC values of 50 μg/mL. It effectively inhibited biofilm formation and destroyed the preformed biofilm, as well as hindered the adhesion of *Cryptococcus* species. The current work would enrich the chemical diversity of sesquiterpenoid family.

**Graphical Abstract:**

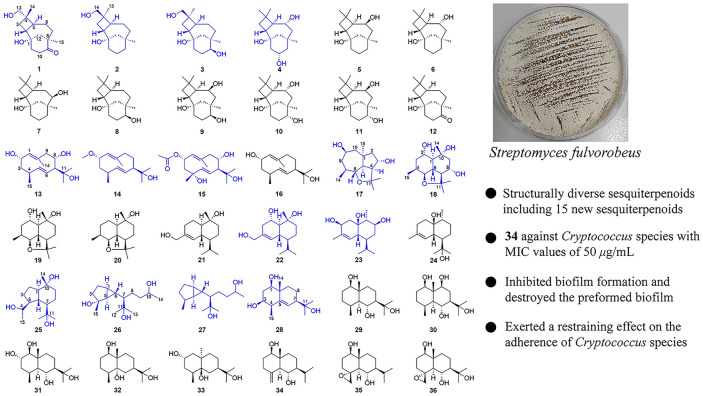

**Supplementary Information:**

The online version contains supplementary material available at 10.1007/s13659-024-00481-9.

## Introduction

A huge amount of microorganisms colonize the guts of mammals [1, 2]. These microbes and their metabolites possess the ability to regulate intestinal epithelial cell proliferation, angiogenesis, host energy, lipid metabolism, and inflammatory immune response [3–6]. The bacterial community in fresh and unpolluted feces could largely represent the distal gut bacteria. Due to its noninvasive and convenient collection, fecal samples are commonly used for studying gut bacteria [7, 8]. In the past few years, the authors have reported on research regarding the structural diversity, antimicrobial, anti-inflammatory, and cytotoxic activities associated with animal feces [9–15]. These findings demonstrated that animal gut microorganisms could be considered as an abundant and significant microbial resource, which has prompted an investigation into the secondary metabolites produced by actinobacteria inhabiting in the animal intestinal tract.

Sesquiterpenoids undergo diverse cyclization cascades with their substrate farnesyl diphosphate (FPP), resulting in a variety of structural skeleton types and are widely distributed in plants, fungi, and red algae [16–18]. However, the discovery of these metabolites in bacteria has been limited due to difficulties in separation, low yield, and the absence of chromophores [19, 20]. With further research, the genome mining technology was applied and it has been found that terpene synthase and cyclase are also widely distributed in bacteria, especially actinomycetes [20–22]. According to the literatures, germacrane, pentalenene, zizaane, cadinane, and caryolane are the five most commonly detected type of sesquiterpenoids in bacteria [21, 23]. The exploration of a wider range of sesquiterpenoids in bacteria holds great prospect.

In an ongoing search for structurally diverse sesquiterpenes from actinomycetes associated with animal feces, we systematically investigated the secondary metabolites of *Streptomyces fulvorobeus* (YIM 103582), which was isolated from *Elephas maximus* feces. The chemical analysis of fermentation broth of *S. fulvorobeus* led to obtain fifteen new compounds, (1*S*,2*R*,4*S*,5*S*,8*R*)-9-oxocaryolane-1,13-diol (**1**), (1*S*,2*R*,4*R*,5*S*,8*R*)-caryolane-1,14-diol (**2**), (1*S*,2*R*,4*R*,5*S*,8*R*,9*S*)-caryolane-1,9,14-triol (**3**), caryolane-1,6*α*,10*α*-triol (**4**), (2*S*,4*S*,7*S*,8*S*)-1(10)*E*,5*E*-germacradiene-2,8,11-triol (**13**), (2*S*,4*S*,7*R*)-1(10)*E*,5*E*-germacradiene-2,11-diol 2-methyl ether (**14**), (2*S*,4*R*,7*S*,8*S*)-1(10)*E*,5*E*-germacradiene-2,4,8,11-tetraol 2-acetate (**15**), (1*α*,3*α*,4*β*,5*α*,6*α*,7*β*,9*β*)-6,11-epoxyisodaucane-3,9-diol (**17**), 8*α*-hydroxyganodermanol L (**18**), (1*S*,2*S*,6*R*,7*S*,10*R*)-cadinane-2,10,15-triol (**22**), (1*R*,3*S*,6*R*,7*S*,9*S*,10*R*)-3,9-dihydroxyepicubenol (**23**), oplopanane-4,10*α*,11-triol (**25**), 4-*epi*-pallenane-4*α*,10,11-triol (**26**), 4-*epi*-pallenane-10,11-diol (**27**), (1*R*,3*S*,4*R*,7*R*,10*R*)-eudesm-5-ene-1,3,11-triol (**28**) as well as twenty-one known analogues. The types of structural skeleton include caryolane, germacrene, isodaucane, cadinane, epicubenol, oplopanane, pallenane, and eudesmane. The structures of the new compounds were elucidated based on detailed spectroscopic data analysis. In this study, we report the fermentation, isolation, structural elucidation, and evaluation of antimicrobial activities of the isolated compounds.

## Results and discussion

**Fig. 1:**
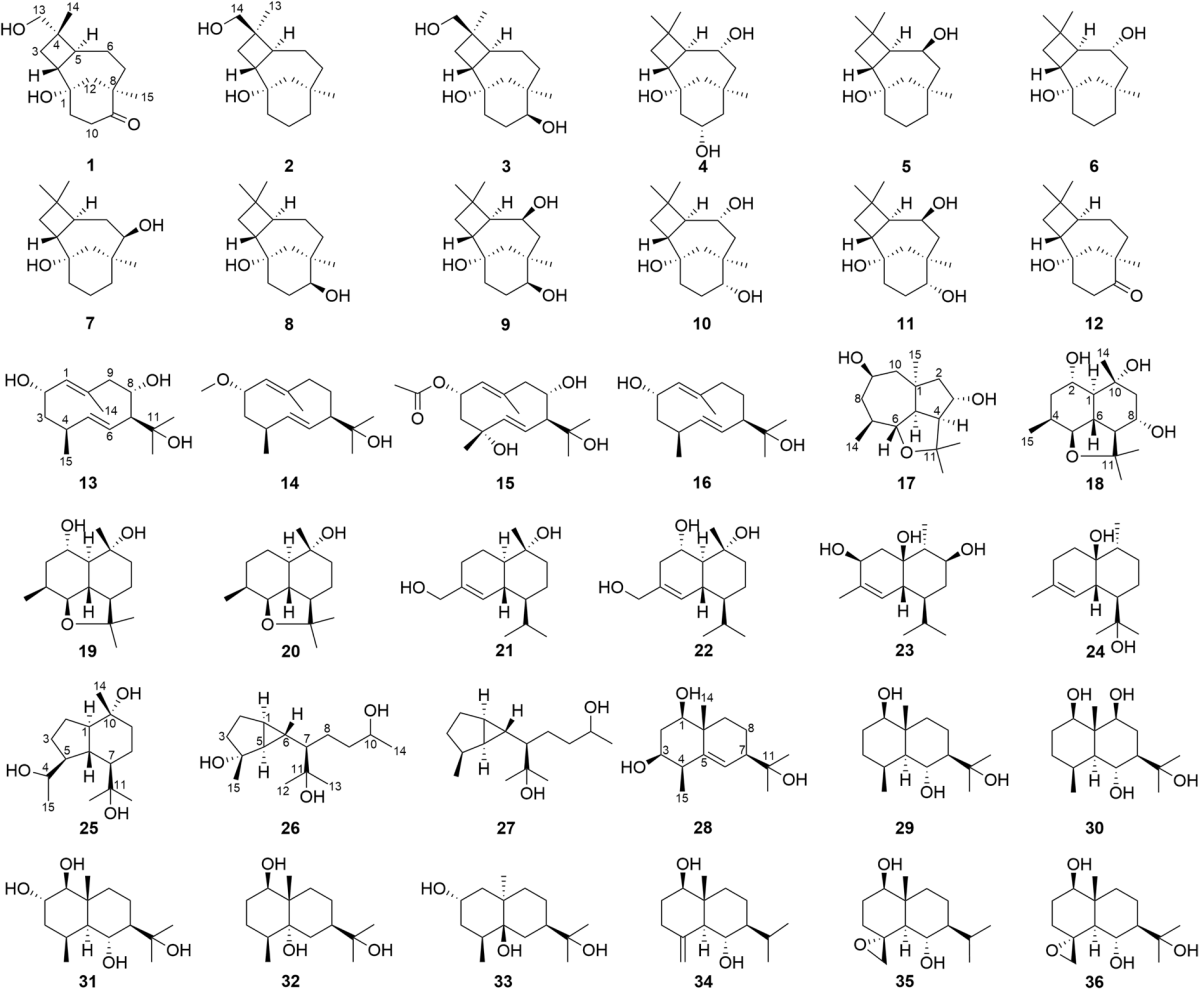
Chemical structures of compounds **1–36** from *S. fulvorobeus*

The *S. fulvorobeus* was obtained from fresh feces of *E. maximus* collected in the Xishuangbanna National Nature Reserve. The fermentation broth of *S. fulvorobeus* was clarified with a centrifuge to collected 150 L of culture supernatant. The clarified supernatant was extracted with ethyl acetate and the extract was isolated by repeated column chromatography over silica gel, Sephadex LH-20, and ODS to afford thirty-six sequiterpenoids (Fig. 1).

### Structural elucidation of isolated new compounds

**Fig. 2:**
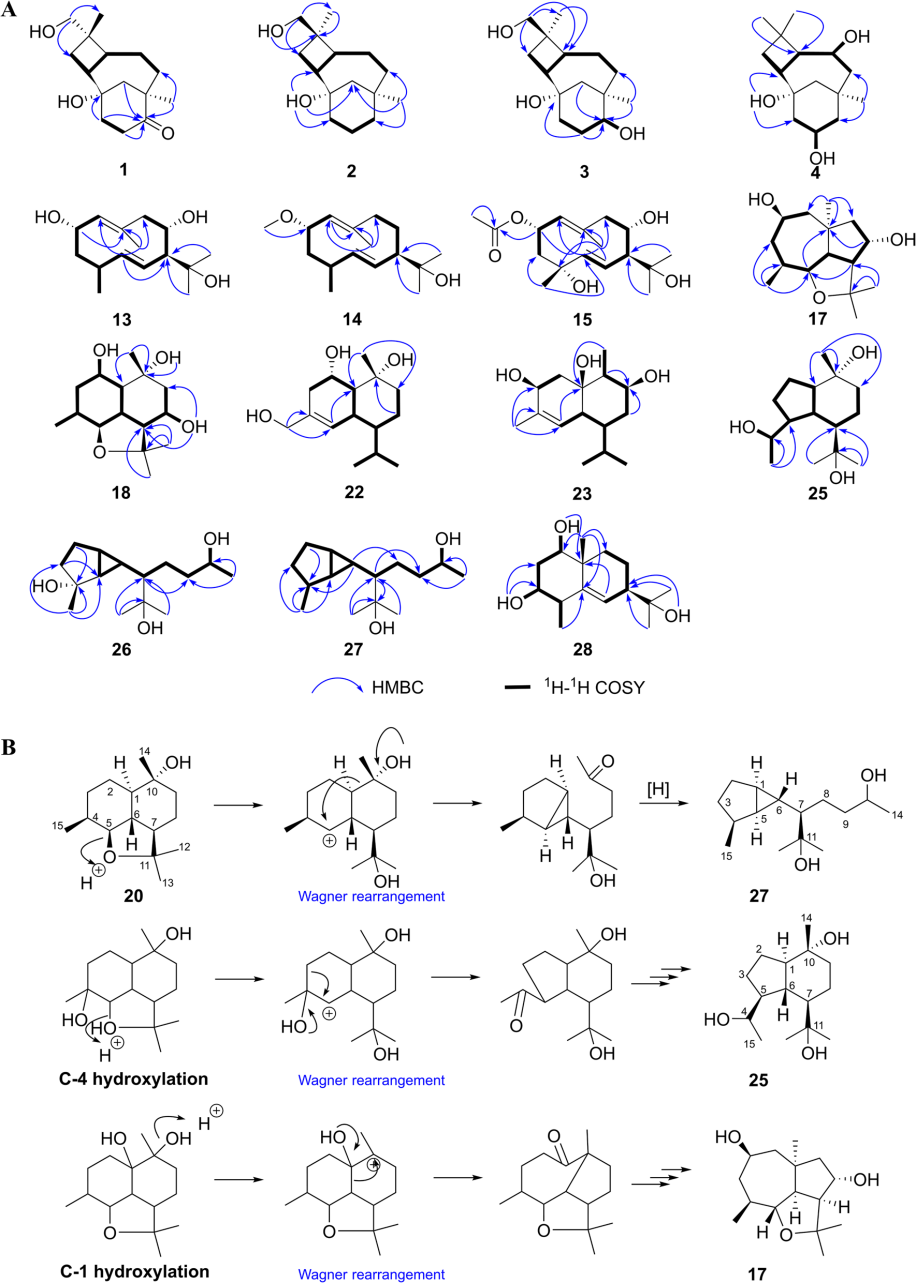
Main HMBC and COSY correlations of compounds **1–4**, **13–15**, **17**, **18**, **22**, **23**, **25–28** (**A)**. proposed biosynthetic pathway of compounds **17**, **25**, **27** (**B**)

**Fig. 3:**
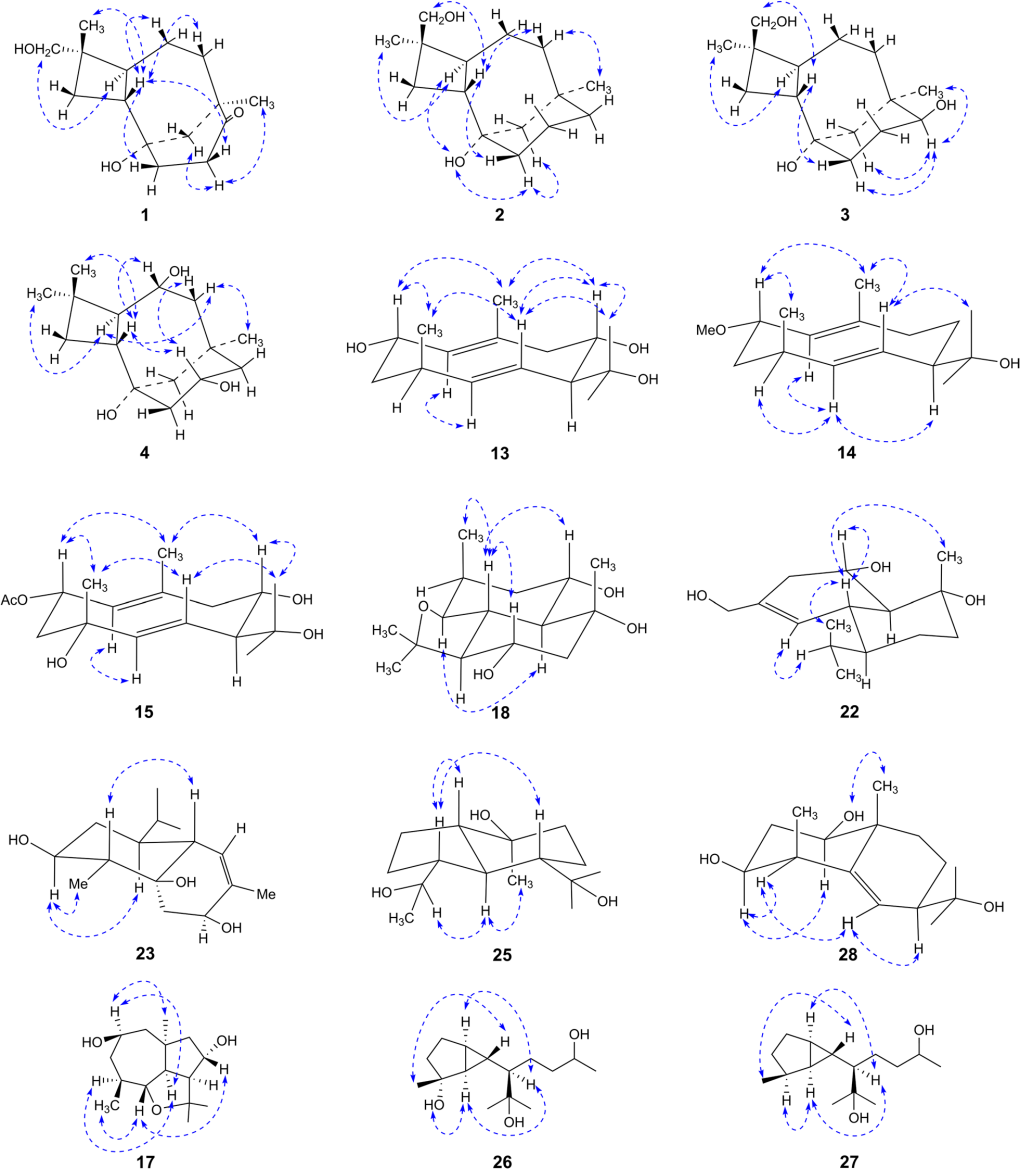
Main NOE correlations of compounds **1–4**, **13–15**, **17**, **18**, **22**, **23**, **25–28**

**Fig. 4:**
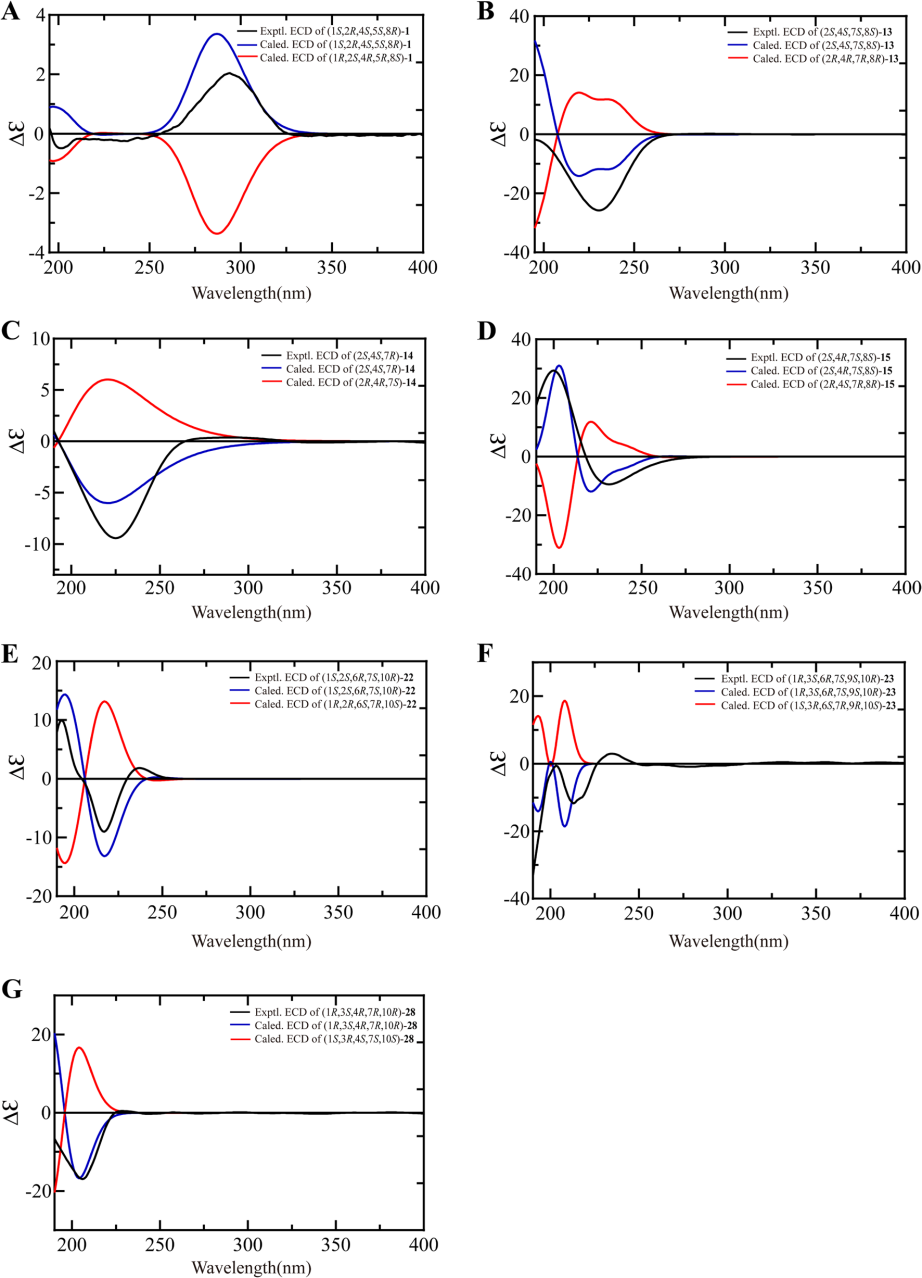
Experimental ECD spectra and calculated ECD spectra of compounds **1** (**A**), **13–15** (**B**−**D**), **22** (**E**), **23** (**F**), **28** (**G**)

Compound **1** was isolated as a colorless oil with the molecular formula C_15_H_24_O_3_ based on HRESIMS and ^13^C NMR data. The IR spectrum of **1** showed characteristic absorption bands for hydroxy groups (3382 cm^−1^) and carbonyl (1699 cm^−1^). The ^1^H NMR (Table 1) of **1** displayed the presence of an oxygenated methylene (*δ*_H_ 3.14, 3.11), two singlet methyls (*δ*_H_ 1.01, 0.88), and other aliphatic residues at *δ*_H_ 0.98–2.82. The ^13^C NMR data (Table 2) of **1** showed 15 carbons signals, including one carbonyl (*δ*_C_ 216.9), two oxygen bearing carbons (*δ*_C_ 71.0, 68.1), two quaternary carbons (*δ*_C_ 46.5, 38.4), six methylenes (*δ*_C_ 49.6, 38.6, 36.9, 34.7, 30.3, 26.4), two methines (*δ*_C_ 43.5, 39.9), and two methyls (*δ*_C_ 31.1, 17.7). Analysis of the 1D and 2D NMR data indicated that **1** was consistent with a caryolane-type sesquiterpenoid and exhibited similarity to the reported bacaryolane A [19], except for the presence of an additional hydroxymethyl (*δ*_C_ 71.0, *δ*_H_ 3.14, 3.11) and the absence of a methyl. The HMBC correlations from H-13 (*δ*_H_ 3.14, 3.11) to C-3 (*δ*_C_ 30.3) and C-14 (*δ*_C_ 17.7), from H-3*β* (*δ*_H_ 1.78) to C-13 (*δ*_C_ 71.0), and from H-14 (*δ*_H_ 0.88) to C-13 (*δ*_C_ 71.0) established the hydroxymethyl was located at C-4. Moreover, the HMBC correlations from H-10 (*δ*_H_ 2.82, 2.17), H-11 (*δ*_H_ 1.84, 1.70), H-12 (*δ*_H_ 1.89), and H_3_-15 (*δ*_H_ 1.01) to C-9 (*δ*_C_ 216.9) confirmed that the carbonyl was located at C-9 (Fig. 2A). Caryolanes derived from plants and bacteria possessed varied stereochemical structures as they were biosynthesized by different cyclization from the humulyl cation [19]. The carbon skeleton of caryolanes in the current study was defined the same as those from bacteria such as bacaryolanes A-C [19]. The NOESY correlations found between H-13 (*δ*_H_ 3.14, 3.11) and H-5 (*δ*_H_ 1.82), between H_3_-14 (*δ*_H_ 0.88) and H-2 (*δ*_H_ 1.59) established that H-2 and H_3_-14 were *β*-orientated, whereas H-5 and H_2_-13 were *α*-orientated. In addition, NOE correlations between H_3_-15 (*δ*_H_ 1.01) and H-10*α* (*δ*_H_ 2.82), between H-10*β* (*δ*_H_ 2.17) and H-2 (*δ*_H_ 1.59) further confirmed the structure (Fig. 3). The absolute configuration 1*S*,2*R*,4*S*,5*S*,8*R* were deduced from comparison of experimental and calculated ECD spectra of **1** (Fig. 4A). According to the literature, the proton signals of the oxymethylene protons in the (*S*)- and (*R*)-*α*-methoxy-*α*-trifluoromethylphenylacetyl (MTPA) esters of primary alcohol showed a unique split pattern [24]. **1** was treated with (*R*)-MTPA-Cl and (*S*)-MTPA-Cl to afford the (*S*)- or (*R*)-MTPA ester derivatives **1a** and **1b**, respectively. The signals of oxymethylene protons at C-13 for the (*S*)-MTPA ester **1a** appeared at *δ*_low_ 4.26 and *δ*_high_ 4.24 (Δ*δ* 0.02), while those for the (*R*)-MTPA ester **1b** were observed as two separated doublet signals at *δ*_low_ 4.30 and *δ*_high_ 4.22 (Δ*δ* 0.08). The (*R*)-MTPA esters of primary alcohol analogue possessing 4*S*-configuration had relatively larger Δ*δ* (*δ*_low_–*δ*_high_) values. Therefore, the structure of **1** was elucidated as (1*S*,2*R*,4*S*,5*S*,8*R*)-9-oxocaryolane-1,13-diol.

**Table 1:** ^1^H NMR (600 MHz, DMSO-*d*_6_) spectroscopic data for compounds **1–4**

No	**1**	**2**	**3**	**4**
2	1.59, q (9.6)	2.22, brq (10.8)	2.27, brq (11.5)	1.86, brq (10.2)
3	1.78, t (10.2) 1.26, t (9.6)	1.61, m 1.40, m	1.59, m 1.35, m	1.50, t (10.0) 1.37, t (9.0)
5	1.82, m	1.85, dt (12.1, 7.6)	1.89, ddd (12.2, 9.4, 6.3)	1.61, dd (12.0, 8.4)
6	1.34, m 1.11, qd (12.6, 5.4)	1.48, m 1.38, m	1.49, m 1.37, m	3.51, m
7	1.87, m 0.98, td (12.7, 5.4)	1.50, m 1.00, m	1.58, m 0.87, m	1.51, m 1.15, dd (14.4, 7.8)
9		1.31, m 0.95, m	3.10, t (10.2)	1.57, dd (12.0, 4.2) 0.88, t (12.0)
10	2.82, ddd (16.6, 12.0, 4.8) 2.17, ddd (16.6, 9.6, 3.6)	1.63, m 1.53, m	1.63, m 1.58, m	3.68, m
11	1.84, m 1.70, m	1.45, m 1.16, td (12.0, 5.2)	1.43, m 1.30, m	1.74, brd (11.4) 1.05, m
12	1.89, brs 1.89, brs	1.57, brd (12.8) 0.91, brd (12.8)	1.46, m 0.89, m	1.83, brd (12.7) 0.92, d (12.7)
13	3.14, d (10.2) 3.11, d (10.2)	0.98, s	0.98, s	1.01, s
14	0.88, s	3.48, dd (10.6, 4.8) 3.40, dd (10.6, 5.4)	3.49, d (10.4) 3.42, d (10.4)	1.03, s
15	1.01, s	0.81, s	0.82, s	0.95, s
1-OH		3.94, s	3.97, brs	4.08, s
6-OH				4.08, s
9-OH			4.25, brs	
10-OH				4.37, s
14-OH		4.28, t (5.2)	4.33, brs

**Table 2:** ^13^C NMR (150 MHz, DMSO-*d*_6_) spectroscopic data for compounds **1–4**, **13–15**

No	**1**	**2**	**3**	**4**	**13**	**14**	**15**
1	68.1, C	69.4, C	69.2, C	69.5, C	136.1, CH	131.4, CH	129.8, CH
2	43.5, CH	39.6, CH	37.5, CH	41.5, CH	63.8, CH	73.8, CH	69.3, CH
3	30.3, CH_2_	30.6, CH_2_	30.3, CH_2_	35.5, CH_2_	40.5, CH_2_	40.0, CH_2_	45.4, CH_2_
4	38.4, C	39.7, C	39.9, C	34.1, C	32.3, CH	32.0, CH	70.5, C
5	39.9, CH	44.1, CH	43.4, CH	52.6, CH	139.7, CH	138.9, CH	142.1, CH
6	26.4, CH_2_	21.5, CH_2_	19.7, CH_2_	70.2, CH	121.7, CH	125.6, CH	123.7, CH
7	38.6, CH_2_	36.8, CH_2_	30.1, CH_2_	49.7, CH_2_	61.8, CH	58.7, CH	60.9, CH
8	46.5, C	34.6, C	38.8, C	34.4, C	67.6, CH	21.9, CH_2_	68.6, CH
9	216.9, C	37.6, CH_2_	77.1, CH	48.6, CH_2_	52.1, CH_2_	41.4, CH_2_	51.6, CH_2_
10	34.7, CH_2_	20.7, CH_2_	30.0, CH_2_	66.1, CH	132.9, C	138.2, C	137.7, C
11	36.9, CH_2_	38.6, CH_2_	38.3, CH_2_	50.0, CH_2_	73.4, C	70.8, C	73.4, C
12	49.6, CH_2_	49.3, CH_2_	48.4, CH_2_	48.2, CH_2_	30.8, CH_3_	29.8, CH_3_	30.8, CH_3_
13	71.0, CH_2_	26.6, CH_3_	26.6, CH_3_	31.8, CH_3_	24.5, CH_3_	26.0, CH_3_	24.6, CH_3_
14	17.7, CH_3_	65.3, CH_2_	65.3, CH_2_	21.5, CH_3_	18.4, CH_3_	17.5, CH_3_	18.4, CH_3_
15	31.1, CH_3_	33.6, CH_3_	29.6, CH_3_	36.9, CH_3_	16.1, CH_3_	16.0, CH_3_	24.3, CH_3_
2-OAc							169.9, C
							21.5, CH_3_
2-OCH_3_						54.5, CH_3_

Compound **2** had the molecular formula C_15_H_26_O_2_ by its HRESIMS and ^13^C NMR data. Its ^1^H and ^13^C NMR data revealed that **2** was a caryolane type sesquiterpenoid and related to the known compound caryolan-1-ol [25]. The distinct difference was that a methyl of caryolan-1-ol was replaced by a hydroxymethyl (*δ*_C_ 65.3, *δ*_H_ 3.48, 3.40) in **2**. The HMBC correlations between H-14 (*δ*_H_ 3.48, 3.40) and C-3 (*δ*_C_ 30.6) and C-13 (*δ*_C_ 26.6), between H-3 (*δ*_H_ 1.61, 1.40), H-13 (*δ*_H_ 0.98) and C-14 (*δ*_C_ 65.3) determined the hydroxymethyl at C-4 (Fig. 2A). NOE correlations between H-5 (*δ*_H_ 1.85) and 1-OH (*δ*_H_ 3.94), H_3_-13 (*δ*_H_ 0.98), between H-2 (*δ*_H_ 2.22) and H-14 (*δ*_H_ 3.48, 3.40), H-7*β* (*δ*_H_ 1.50), between H-7*α* (*δ*_H_ 1.00) and H_3_-15 (*δ*_H_ 0.81) indicated that H-2, H-14 were *β*-orientation whereas 1-OH, H-5, H_3_-13, H_3_-15 were *α*-orientation (Fig. 3). The absolute configuration of C-4 was determined by Mosher’s method. Treatment of **2** with (*R*)-MTPA-Cl or (*S*)-MTPA-Cl obtained the *S* or *R* Mosher’s esters **2a** and **2b**. The signals of oxymethylene protons at C-14 for the (*S*)-MTPA ester **2a** showed two separated doublet signals at *δ*_low_ 4.69 and *δ*_high_ 4.55 (Δ*δ* 0.14), while those for the (*R*)-MTPA ester **2b** were presented at *δ*_H_ 4.63 as a broad singlet peak. These findings suggested the *R*-configuration of C-4 in **2** by comparing the Δ*δ* values of oxymethylene protons with those of 4*S*-configuration analogue **1**. Thus, **2** was deduced to be (1*S*,2*R*,4*R*,5*S*,8*R*)-caryolane-1,14-diol as the same biosynthesis pathway from bacteria.

Compound **3** afforded a molecular formula of C_15_H_26_O_3_ on the basis of HRESIMS and ^13^C NMR data. Analysis of its ^1^H and ^13^C NMR data (Tables 1, 2) indicated that **3** has a close structural relationship to **2**. The only difference was that one methylene was absent and one oxygenated methine was present (*δ*_C_ 77.1, *δ*_H_ 3.10) in **3**. The HMBC correlations from H-10 (*δ*_H_ 1.63, 1.58), H-12 (*δ*_H_ 1.46, 0.89), H_3_-15 (*δ*_H_ 0.82) to C-9 (*δ*_C_ 77.1) as well as ^1^H–^1^H COSY correlations between H-9 (*δ*_H_ 3.10) and H-10 (*δ*_H_ 1.63, 1.58) confirmed a hydroxy at C-9 (Fig. 2A). The NOE cross peaks observed between H_3_-13 (*δ*_H_ 0.98) and H-5 (*δ*_H_ 1.89), between H_3_-15 (*δ*_H_ 0.82) and H-9 (*δ*_H_ 3.10), between H-9 (*δ*_H_ 3.10) and H-11*α* (*δ*_H_ 1.30), and between H-2 (*δ*_H_ 2.27) and H-14 (*δ*_H_ 3.49, 3.42) and H-11*β* (*δ*_H_ 1.43) indicated that H-2, H-14, and 9-OH were *β*-oriented, while H-5, H-9, H_3_-13, and H_3_-15 were *α*-oriented (Fig. 3). The (*S*)-MTPA ester (3a) or (*R*)-MTPA ester (3b) were obtained by the same derivative process as those of **1** and **2**. Both 9-OH and 14-OH in compound **3** were esterified by MTPA-Cl according to NMR data. Unfortunately, it was impossible to assign the absolute configuration of C-4 as the Δ*δ* values of two separated peaks of H_2_-14 for 3a and 3b were very approximate (Δ*δ* = 0.12 for **3a** and Δ*δ* = 0.10 for **3b**) being influenced by two MTPA groups. As **3** presented almost identical ^13^C NMR data of C-4, C-13, and C-14 with those of compound **2**, the *R*-configuration of C-4 could be determined. The configuration of C-9 could be deduced by comparing the chemical shift of H_3_-15 in (*S*)- and (*R*)-MTPA esters. The signal of H_3_-15 for (*S*)-MTPA ester 3a was observed at lower field (*δ*_H_ 0.99) compared to the signal for (*R*)-MTPA ester **3b** (*δ*_H_ 0.88), which exhibited the absolute configuration of C-9 to be *S*. Thus, the structure of **3** was confirmed as (1*S*,2*R*,4*R*,5*S*,8*R*,9*S*)-caryolane-1,9,14-triol.

The molecular formula of **4** was deduced as C_15_H_26_O_3_ according to its HRESIMS and ^13^C NMR data. The ^1^H NMR and ^13^C NMR data (Tables 1, 2) indicated that **4** was similar to bacaryolane C (**6**) [19], also isolated in the current study. The obvious alteration was that a methylene in **6** was replaced by an oxygenated methine (*δ*_C_ 66.1, *δ*_H_ 3.68) in **4**. The HMBC correlations from H-9 (*δ*_H_ 1.57, 0.88), H-11 (*δ*_H_ 1.74, 1.05) to C-10 (*δ*_C_ 66.1) confirmed that a hydroxy was located at C-10. The COSY correlations from H-10 (*δ*_H_ 3.68) to H-9 (*δ*_H_ 1.57, 0.88), H-11 (*δ*_H_ 1.74, 1.05), and 10-OH (*δ*_H_ 4.37) further supported the above inference (Fig. 2A). The NOESY cross peaks from H-2 (*δ*_H_ 1.86) to H-6 (*δ*_H_ 3.51) and H-10 (*δ*_H_ 3.68), from H-10 (*δ*_H_ 3.68) to H-7*β* (*δ*_H_ 1.51) indicated that H-2, H-6, and H-10 were on the same side. Correspondingly, the NOESY correlations from H_3_-15 (*δ*_H_ 0.95) to H-7*α* (*δ*_H_ 1.15), from H-7*α* (*δ*_H_ 1.15) to H-5 (*δ*_H_ 1.61) suggested that H-5, H_3_-15 were on the opposite side (Fig. 3). Consequently, **4** was deduced to be caryolane-1,6*α*,10*α*-triol.

Compound **13** was isolated as a colorless oil with a molecular formula C_15_H_26_O_3_ by HRESIMS and ^13^C NMR data. Its IR spectrum revealed the presence of hydroxy groups (3315 cm^−1^) and double bonds (1668 cm^−1^). The ^1^H NMR data (Table 3) showed three olefinic hydrogens (*δ*_H_ 5.41, 4.90, 4.65), two oxygenated methines (*δ*_H_ 4.19, 3.80), three singlet methyls (*δ*_H_ 1.53, 1.14, 0.93), one doublet methyl (*δ*_H_ 0.97), and other aliphatic hydrogens (*δ*_H_ 1.37–2.41). The ^13^C NMR data (Table 2) of **13** displayed 15 carbon signals, including four olefinic carbons (*δ*_C_ 139.7, 136.1, 132.9, 121.7), three oxygen-bearing carbons (*δ*_C_ 73.4, 67.6, 63.8), two methines (*δ*_C_ 61.8, 32.3), two methylenes (*δ*_C_ 52.1, 40.5), and four methyls (*δ*_C_ 30.8, 24.5, 18.4, 16.1), as determined in an HSQC experiment. The ^1^H and ^13^C NMR data of **13** were very similar to those of 1(10)*E*,5*E*-germacradiene-2*α*,11-diol (**16**) [26], a germacrane-type sesquiterpenoid. The major differences were the disappearance of a methylene in **16** and the existence of an oxygen-bearing methine (*δ*_C_ 67.6, *δ*_H_ 3.80) in **13**. The ^1^H–^1^H COSY correlations from H-8 (*δ*_H_ 3.80) to H-7 (*δ*_H_ 2.28) and H-9 (*δ*_H_ 2.27, 2.25) as well as the HMBC correlations between H-7 (*δ*_H_ 2.28), H-9 (*δ*_H_ 2.27, 2.25) and C-8 (*δ*_C_ 67.6) indicated that a hydroxy was connected with C-8 (Fig. 2A). Thus, the planar structure of compound **13** was established. The NOE interactions observed between H-2 (*δ*_H_ 4.19) and H_3_-15 (*δ*_H_ 0.97), between H-6 (*δ*_H_ 4.65) and H-8 (*δ*_H_ 3.80), H_3_-15 (*δ*_H_ 0.97), between H_3_-13 (*δ*_H_ 1.14) and H-6 (*δ*_H_ 4.65), H-8 (*δ*_H_ 3.80) revealed that H-2, H-8, and H_3_-15 were on the same side, while 2-OH, 8-OH, H-4, and H-7 were located on the opposite side (Fig. 3). Furthermore, the coupling constants of H-2 (*δ*_H_ 4.19, td, *J* = 10.2, 4.2 Hz) suggested that the 2-OH presented in the equatorial position. In addition, NOE interactions observed from H_3_-14 (*δ*_H_ 1.53) to H-2 (*δ*_H_ 4.19) and H-8 (*δ*_H_ 3.80), from H-1 (*δ*_H_ 4.90) to H-5 (*δ*_H_ 5.41) as well as the large coupling constant of H-5/H-6 elucidated the *E* configurations of C-1/C-10 and C-5/C-6 double bonds. The experimental ECD spectrum of **13** had a consistent trend with its corresponding calculated ECD curve which determined the 2*S*,4*S*,7*S*,8*S*-configurations (Fig. 4B). Consequently, **13** was established as (2*S*,4*S*,7*S*,8*S*)-1(10)*E*,5*E*-germacradiene-2,8,11-triol.

**Table 3:** ^1^H NMR (600 MHz, DMSO-*d*_6_) spectroscopic data for compounds **13–15**, **17**, **18**

No	**13**	**14**	**15**	**17**	**18**
1	4.90, d (9.6)	4.80, d (10.2)	4.82, d (10.2)		1.22, dd (12.5, 9.7)
2	4.19, td (10.2, 4.2)	3.90, td (10.2, 3.6)	5.21, dd (11.4, 4.2)	1.82, m	3.67, brt (9.6)
				1.53, dd (13.8, 3.6)	
3	1.67, dt (13.2, 4.2) 1.37, ddd (12.6, 10.8, 3.6)	1.76, dt (13.2, 4.2) 1.52, ddd (13.8, 10.8, 3.6)	1.76, dd (12.0, 3.6) 1.48, t (12.0)	4.17, quint (5.6)	1.69, ddd (13.8, 4.2, 3.0) 1.42, m
4	2.41, m	2.41, m		2.30, dd (8.4, 4.8)	2.05, m
5	5.41, dd (15.6, 3.6)	5.35, dd (15.6, 3.0)	5.20, d (15.6)	1.94, t (9.5)	3.46, dd (10.2, 4.8)
6	4.65, ddd (15.6, 10.2, 2.4)	4.84, ddd (15.6, 9.6, 1.8)	4.72, dd (15.6, 10.2)	3.13, t (9.9)	1.16, m
7	2.28, m	2.10, brq (10.8)	2.25, t (10.2)	1.42, m	1.38, m
8	3.80, td (9.6, 3.6)	1.71, m 1.24, brq (11.4)	3.87, td (10.2, 4.2)	1.82, m 1.04, q (12.0)	3.38, m
9	2.27, m 2.25, m	2.27, dd (12.6, 4.2) 2.16, td (12.6, 1.8)	2.32, dd (12.3, 9.7) 2.29, dd (12.3, 4.0)	3.51, td (10.2, 1.8)	1.77, dd (12.0, 3.6) 1.40, m
10				1.62, brd (13.6)	
				1.48, dd (13.6, 10.2)	
12	0.93, s	0.99, s	0.98, s	1.17, s	1.26, s
13	1.14, s	0.94, s	1.14, s	1.17, s	1.07, s
14	1.53, s	1.57, s	1.61, s	0.87, d (6.0)	1.17, s
15	0.97, d (6.6)	1.02, d (6.6)	1.15, s	1.16, s	0.84, d (7.2)
2-OH					4.34, d (3.0)
2-OAc			1.93, s		
2-OCH_3_		3.06, s			
8-OH					4.56, d (5.4)
10-OH					4.33, s
11-OH		4.04, s			

The ^1^H and ^13^C NMR data (Tables 2, 3) as well as a molecular formula of C_16_H_28_O_2_ exhibited that **14** bore a close resemblance to the known compound **16** [26], except for the presence of an additional methoxy (*δ*_C_ 54.5, *δ*_H_ 3.06). The HMBC correlation between methoxy (*δ*_H_ 3.06) and C-2 (*δ*_C_ 73.8) determined that the methoxy was located at C-2 (Fig. 2A). In NOESY spectrum, the key cross peaks from H-2 (*δ*_H_ 3.90) to H_3_-15 (*δ*_H_ 1.02), from H-5 (*δ*_H_ 5.35) to H-4 (*δ*_H_ 2.41) and H-7 (*δ*_H_ 2.10) indicated that H-2 and H_3_-15 were *β*-orientation, while H-4 and H-7 were *α*-orientation (Fig. 3). Moreover, the double bonds at C-5/C-6, C-1/C-10 were elucidated as *E* geometry based on the NOE correlations from H_3_-14 (*δ*_H_ 1.57) to H-2 (*δ*_H_ 3.90) and H-6 (*δ*_H_ 4.84), from H-1 (*δ*_H_ 4.80) to H-5 (*δ*_H_ 5.35) as well as the large coupling constant of H-5/H-6 (*J* = 15.6 Hz). The absolute configurations of C-2, C-4, and C-7 were elucidated as 2*S*,4*S*,7*R* based on comparing the experimental and calculated ECD spectra (Fig. 4C). Therefore, the structure of **14** was confirmed as (2*S*,4*S*,7*R*)-1(10)*E*,5*E*-germacradiene-2,11-diol 2-methyl ether.

Compound **15** was assigned a molecular formula of C_17_H_28_O_5_ based on its HRESIMS and ^13^C NMR data. The characteristic ^1^H and ^13^C NMR data (Tables 2, 3) of **15** suggested that it was a germacrane-type sesquiterpenoid and had a close structural relationship to **13**, except for the different oxygenated position and an additional acetyl (*δ*_C_ 169.9, 21.5). HMBC correlations from H-3 (*δ*_H_ 1.76, 1.48), H-6 (*δ*_H_ 4.72) and H_3_-15 (*δ*_H_ 1.15) to C-4 (*δ*_C_ 70.5) confirmed that the extra hydroxy was located at C-4. HMBC correlations from H-2 (*δ*_H_ 5.21) to C-16 (*δ*_C_ 169.9) as well as ^1^H–^1^H COSY correlations from H-2 (*δ*_H_ 5.21) to H-1 (*δ*_H_ 4.82) and H-3 (*δ*_H_ 1.76, 1.48) indicated that the acetyl was located at C-2. (Fig. 2A). **15** presented the same relative configuration as those of **13** and **14** on the basis of NOE correlations between H-2 (*δ*_H_ 5.21) and H_3_-15 (*δ*_H_ 1.15), H_3_-14 (*δ*_H_ 1.61), between H_3_-14 (*δ*_H_ 1.61) and H-8 (*δ*_H_ 3.87), between H_3_-13 (*δ*_H_ 1.14) and H-6 (*δ*_H_ 4.72), H-8 (*δ*_H_ 3.87) (Fig. 3). Besides, the geometry at C-5/C-6 double bonds was assigned as *E* according to the large coupling constants of H-5/H-6 (*J* = 15.6 Hz). The (+) Cotton effect at 200 nm (+ 29.25) and (−) Cotton effect at 231 nm (− 9.42) of **15** detected by CD spectrum was consistent with those of the calculated ECD curve of 2*S*,4*R*,7*S*,8*S*-**15** (Fig. 4D). Thus, **15** was identified as (2*S*,4*R*,7*S*,8*S*)-1(10)*E*,5*E*-germacradiene-2,4,8,11-tetraol 2-acetate.

The HRESIMS and ^13^C NMR data determined the molecular formula of **17** as C_15_H_26_O_3_. The NMR data (Tables 3, 4) suggested **17** was a 6,11-epoxyisodaucane type sesquiterpenoid and related to the known compound 6,11-epoxyisodaucane [27]. The difference was that two oxygenated methines replaced two methylenes in **17**. ^1^H–^1^H COSY correlations from H-3 (*δ*_H_ 4.17) to H-4 (*δ*_H_ 2.30) and H-2 (*δ*_H_ 1.82, 1.53), from H-9 (*δ*_H_ 3.51) to H-10 (*δ*_H_ 1.62, 1.48) and H-8 (*δ*_H_ 1.82, 1.04) determined the two hydroxys at C-3 and C-9, respectively. The HMBC correlations between H_3_-15 (*δ*_H_ 1.16) and C-1 (*δ*_C_ 38.9), C-2 (*δ*_C_ 51.9), C-5 (*δ*_C_ 61.6), and C-10 (*δ*_C_ 51.7), between H-6 (*δ*_H_ 3.13) and C-1 (*δ*_C_ 38.9), C-8 (*δ*_C_ 47.7), between H-5 (*δ*_H_ 1.94) and C-2 (*δ*_C_ 51.9), C-3 (*δ*_C_ 73.0), C-10 (*δ*_C_ 51.7), and C-15 (*δ*_C_ 31.3), between H-12 (*δ*_H_ 1.17) and C-4 (*δ*_C_ 64.2), C-11 (*δ*_C_ 80.0) confirmed the planar structure (Fig. 2A). The absolute configuration of 6,11-epoxyisodaucane was determined by total synthesis method [27]. The similar coupling constants of H-5 (*δ*_H_ 1.94, t, *J* = 9.5 Hz), H-6 (*δ*_H_ 3.13, t, *J* = 9.9 Hz) indicated that **17** possessed the same 4*α*-H, 5*α*-H, 6*β*-H, 7*β*-methyl configuratinos as those of synthesized 6,11-epoxyisodaucane. The NOE interactions from H-5 (*δ*_H_ 1.94) to H-9 (*δ*_H_ 3.51), H-7 (*δ*_H_ 1.42), from H_3_-14 (*δ*_H_ 0.87) to H-6 (*δ*_H_ 3.13), from H-6 (*δ*_H_ 3.13) to H-3 (*δ*_H_ 4.17), from H_3_-15 (*δ*_H_ 1.16) to H-9 (*δ*_H_ 3.51) determined the configurations as in Fig. 3.

**Table 4:** ^13^C NMR (150 MHz, DMSO-*d*_6_) spectroscopic data for compounds **17**, **18**, **22**, **23**, **25–28**

No	**17**	**18**	**22**	**23**	**25**	**26**	**27**	**28**
1	38.9, C	52.9, CH	53.8, CH	74.6, C	58.9, CH	23.8, CH	23.8, CH	76.5, CH
2	51.9, CH_2_	66.7, CH	70.1, CH	34.3, CH_2_	25.7, CH_2_	26.2, CH_2_	28.0, CH_2_	35.6, CH_2_
3	73.0, CH	39.8, CH_2_	36.9, CH_2_	68.0, CH	24.2, CH_2_	37.2, CH_2_	30.2, CH_2_	68.7, CH
4	64.2, CH	31.0, CH	136.9, C	136.4, C	67.2, CH	78.8, C	35.0, CH	45.5, CH
5	61.6, CH	80.8, CH	120.7, CH	124.2, CH	48.1, CH	36.3, CH	30.1, CH	145.9, C
6	82.3, CH	40.0, CH	40.5, CH	47.4, CH	44.3, CH	23.1, CH	18.9, CH	126.6, CH
7	38.2, CH	59.1, CH	46.5, CH	45.7, CH	55.5, CH	52.0, CH	52.1, CH	48.2, CH
8	47.7, CH_2_	67.0, CH	21.8, CH_2_	34.3, CH_2_	28.4, CH_2_	27.6, CH_2_	27.7, CH_2_	21.1, CH_2_
9	66.7, CH	52.8, CH_2_	41.4, CH_2_	71.3, CH	42.8, CH_2_	39.4, CH_2_	39.4, CH_2_	38.3, CH_2_
10	51.7, CH_2_	74.1, C	73.0, C	51.3, CH	71.2, C	67.1, CH	67.1, CH	39.3, C
11	80.0, C	83.0, C	26.5, CH	26.6, CH	72.4, C	72.9, C	73.0, C	71.5, C
12	24.8, CH_3_	24.4, CH_3_	15.4, CH_3_	15.5, CH_3_	24.5, CH_3_	26.5, CH_3_	26.3, CH_3_	28.2, CH_3_
13	32.8, CH_3_	31.1, CH_3_	21.8, CH_3_	22.0, CH_3_	31.9, CH_3_	30.6, CH_3_	30.7, CH_3_	25.6, CH_3_
14	20.3, CH_3_	23.4, CH_3_	21.7, CH_3_	11.5, CH_3_	20.6, CH_3_	24.2, CH_3_	24.3, CH_3_	21.1, CH_3_
15	31.3, CH_3_	11.9, CH_3_	64.9, CH_2_	20.3, CH_3_	23.3, CH_3_	26.4, CH_3_	18.9, CH_3_	16.7, CH_3_

Analysis of ^1^H NMR, ^13^C NMR (Tables 3, 4), and HRESIMS data of **18** indicated it was a sesquiterpenoid and was related to the reported ganodermanol L (**19**) [28]. The only difference was one more oxygenated methine (*δ*_C_ 67.0, *δ*_H_ 3.38) in **18** replaced a methylene in **19**. The HMBC correlations between 8-OH (*δ*_H_ 4.56) and C-7 (*δ*_C_ 59.1), C-8 (*δ*_C_ 67.0), and C-9 (*δ*_C_ 52.8) determined the C-8 position of extra hydroxy. Moreover, COSY correlations from H-8 (*δ*_H_ 3.38) to H-7 (*δ*_H_ 1.38), H-9 (*δ*_H_ 1.77, 1.40), and 8-OH (*δ*_H_ 4.56) further supported the conclusion (Fig. 2A). NOE cross peaks observed between H-6 (*δ*_H_ 1.16) and H-2 (*δ*_H_ 3.67), H-8 (*δ*_H_ 3.38), H_3_-15 (*δ*_H_ 0.84), between H-5 (*δ*_H_ 3.46) and H-1 (*δ*_H_ 1.22) demonstrated that H-2, H-6, H-8, and H_3_-15 were located on the same side, whereas H-1, H-5, and H-7 were located on the opposite side (Fig. 3). There were potential inaccuracies of NOE correlations due to the overlapped signals of H-6 (*δ*_H_ 1.16) and H_3_-14 (*δ*_H_ 1.17). The configuration of C-10 was then confirmed through comparing the ^13^C NMR data of C-1, C-2, C-10, and C-14 with those of ganodermanol L (**19**) [28], which was elucidated the structure by X-ray crystallographic analyses. In addition, the peak shapes and coupling constants of H-1 (*δ*_H_ 1.22, dd, *J* = 12.5, 9.7 Hz), H-2 (*δ*_H_ 3.67, brt *J* = 9.6 Hz), and H-5 (*δ*_H_ 3.46, dd, *J* = 10.2, 4.8 Hz) further confirmed the relative configuration.

The ^1^H and ^13^C NMR data (Tables 4, 5) as well as molecular formula, C_15_H_26_O_3_, of **22** showed it was a cadinene-type sesquiterpenoid and related to 15-hydroxy-*α*-cadinol (**21**) [29], except an additional oxygenated methine (*δ*_C_ 70.1, *δ*_H_ 3.82) in **22** instead of a methylene in **21**. ^1^H–^1^H COSY correlations from H-2 (*δ*_H_ 3.82) to H-3 (*δ*_H_ 2.20, 1.89) and H-1 (*δ*_H_ 1.30) as well as HMBC correlations from H-1 (*δ*_H_ 1.30), H-3 (*δ*_H_ 2.20, 1.89) to C-2 (*δ*_C_ 70.1) revealed that a hydroxy was located at C-2 (Fig. 2A). The large coupling constants of H-1 (*δ*_H_ 1.30, brt, *J* = 10.6 Hz), H-6 (*δ*_H_ 1.73, brt, *J* = 10.6 Hz) indicated a *trans* fusion of the bicyclic system. NOE correlations from H-6 (*δ*_H_ 1.73) to H-2 (*δ*_H_ 3.82), H_3_-14 (*δ*_H_ 1.18), and H_3_-12 (*δ*_H_ 0.73) indicated that the H-2, H-6, and H_3_-14 were *β*-orientated, whereas H-1, H-7, 2-OH, and 10-OH were *α*-orientated (Fig. 3). Comparison of the experimental and calculated ECD spectra of **22** revealed the absolute configuration as 1*S*,2*S*,6*R*,7*S*,10*R* (Fig. 4E). Thus, compound **22** was elucidated as (1*S*,2*S*,6*R*,7*S*,10*R*)-cadinane-2,10,15-triol.

**Table 5:** ^1^H NMR (600 MHz, DMSO-*d*_6_) spectroscopic data for compounds **22**, **23**, **25–28**

No	**22**	**23**	**25**	**26**	**27**	**28**
1	1.30, t (10.6)		1.26, m	1.04, m	0.97, m	3.03, dt (11.6, 4.8)
2	3.82, td (10.2, 6.0)	1.80, dd (12.8, 6.8) 1.19, m	1.53, m 1.00, m	1.87, m 1.56, dd (12.0, 7.9)	1.65, m 1.65, m	1.65, brq (12.0) 1.60, m
3	2.20, dd (16.2, 4.8) 1.89, m	3.96, brt (8.0)	1.66, ddd (11.5, 7.8, 3.0) 1.22, m	1.34, dd (13.4, 8.4) 1.11, m	1.54, m 0.70, dq (12.8, 10.0)	3.52, m
4			4.23, m		2.15, m	2.39, quint (7.0)
5	5.61, s	5.33, d (5.6)	2.00, brt (9.0)	1.13, dd (6.0, 3.6)	1.12, dt (6.3, 3.5)	
6	1.73, brt (10.6)	1.56, dd (11.2, 4.8)	1.37, td (10.9, 8.0)	0.13, dt (10.3, 3.4)	0.27, dt (10.3, 3.3)	5.53, m
7	1.02, m	1.16, m	1.18, ddd (13.3, 10.5, 3.0)	0.57, ddd (10.0, 8.0, 3.5)	0.56, ddd (10.6, 7.8, 3.5)	2.02, ddd (11.0, 6.2, 2.0)
8	1.51, m 1.04, m	1.67, dt (11.6, 4.3) 0.91, brd (11.6)	1.61, dt (13.3, 3.5) 0.95, m	1.62, m 1.26, m	1.65, m 1.27, m	1.56, m 1.19, m
9	1.61, dt (12.6, 3.0) 1.38, td (12.6, 3.6)	3.00, td (10.8, 4.4)	1.57, dt (12.6, 3.4) 1.28, m	1.60, m 1.25, m	1.61, m 1.25, m	1.80, dt (12.4, 3.2) 1.06, m
10		1.23, m		3.50, m	3.51, m	
11	2.10, m	1.95, m				
12	0.73, d (7.2)	0.78, d (7.2)	1.02, s	1.05, s	1.06, s	1.04, s
13	0.88, d (6.6)	0.86, d (7.2)	1.09, s	1.16, s	1.16, s	0.97, s
14	1.18, s	0.96, d (6.4)	0.98, s	1.04, d (6.1)	1.03, d (6.1)	0.95, s
15	3.77, brd (10.8) 3.75, brd (10.8)	1.64, s	0.94, d (6.3)	1.22, s	0.99, d (6.6)	0.96, d (6.4)
1-OH						4.45, d (4.8)
3-OH						4.50, d (4.0)
4-OH			4.04, d (5.6)	4.24, s		
10-OH			4.03, s	4.26, d (4.4)	4.27, brs	
11-OH			4.08, brs	3.95, s	3.93, brs	4.15, s

The molecular formula of **23** was determined to be C_15_H_26_O_3_ according to HRESIMS and ^13^C NMR data. Analysis of its ^1^H and ^13^C NMR data (Tables 4, 5) indicated that **23** possessed a resemble structural relationship to 3*β*-hydroxyepicubenol [13], except for one oxygenated methine (*δ*_C_ 71.3, *δ*_H_ 3.00) instead of a methylene in **23**. ^1^H–^1^H COSY correlations from H-9 (*δ*_H_ 3.00) to H-8 (*δ*_H_ 1.67, 0.91) and H-10 (*δ*_H_ 1.23) supported that an additional hydroxy was located at C-9. This conclusion was confirmed by HMBC correlations between H-8 (*δ*_H_ 1.67, 0.91), H-10 (*δ*_H_ 1.23) and C-9 (*δ*_C_ 71.3) (Fig. 2A). The relative configuration of **23** was established from detailed analysis of NOE correlations. NOE interactions from H-9 (*δ*_H_ 3.00) to H-7 (*δ*_H_ 1.16) and H_3_-14 (*δ*_H_ 0.96), from H-6 (*δ*_H_ 1.56) to H-10 (*δ*_H_ 1.23) revealed that H-7, H-9, and H_3_-14 were *α*-orientation, while H-6 and H-10 were *β*-orientation (Fig. 3). Although there was no obvious NOE correlation to demonstrate the configuration of 1-OH and 3-OH, comparing the ^13^C NMR data of C-1 (*δ*_C_ 74.6), C-2 (*δ*_C_ 34.3), C-3 (*δ*_C_ 68.0), C-4 (*δ*_C_ 136.4), C-5 (*δ*_C_ 124.2), and C-6 (*δ*_C_ 47.4) of **23** with those of the analogues indicated that **23** possessed the same configurations of C-1 and C-3 as 3*β*-hydroxyepicubenol [13] and muurol-4-ene-1*β*,3*β*,10*β*-triol [30]. The absolute configurations of the chiral carbons were determined to be 1*R*,3*S*,6*R*,7*S*,9*S*,10*R* according to the experimental ECD spectrum of **23** closely matched the calculated ECD curve (Fig. 4F).

Compound **25** had a molecular formula of C_15_H_28_O_3_ from its HRESIMS and ^13^C NMR data. The characteristic ^1^H and ^13^C NMR data (Tables 4, 5) of **25** demonstrated an oplopanane sequiterpenoid and related to the known oplopanane-4,10*α*-diol [31]. The difference was a 2-hydroxypropan-2-yl (*δ*_C_ 72.4, 31.9, 24.5, *δ*_H_ 1.09, s, *δ*_H_ 1.02, s) in **25** at C-7 replaced an isopropyl, which was confirmed by HMBC correlations between H_3_-12 (*δ*_H_ 1.02) and C-7 (*δ*_C_ 55.5), C-11 (*δ*_C_ 72.4), between H_3_-13 (*δ*_H_ 1.09) and C-7 (*δ*_C_ 55.5), C-11 (*δ*_C_ 72.4). The large coupling constants of H-5 (*δ*_H_ 2.00, brt, *J* = 9.0 Hz), H-6 (*δ*_H_ 1.37, td, *J* = 10.9, 8.0 Hz), and H-7 (*δ*_H_ 1.18, ddd, *J* = 13.3, 10.5, 3.0 Hz) indicated the both *trans* configurations of H-5/H-6, H-6/H-7. In addition, the *trans* configuration of H-1/H-6 also could be deduced from the peak shape and coupling constants of H-6 (*δ*_H_ 1.37, td, *J* = 10.9, 8.0 Hz). The relative configuration of **25** was further determined by NOE correlations observed from H-5 (*δ*_H_ 2.00) to H-7 (*δ*_H_ 1.18) and H-1 (*δ*_H_ 1.26), from H-6 (*δ*_H_ 1.37) to H_3_-14 (*δ*_H_ 0.98) and H-4 (*δ*_H_ 4.23) (Fig. 3). Furthermore, comparing the ^13^C NMR data of C-14 (*δ*_C_ 20.6) with that of oplopanone (*δ*_C_ 20.3) or 10-*epi*-oplopanone (*δ*_C_ 28.2) further confirmed above inference [31, 32]. Thus, the structure of **25** was identified as 4,10*α*,11-oplopananetriol and the configuration of C-4 undetermined as the less amount.

The molecular formula of compound **26** was determined as C_15_H_28_O_3_ based on HRESIMS and ^13^C NMR data. The ^1^H NMR data (Table 5) presented one oxygenated methine (*δ*_H_ 3.50), three singlet methyls (*δ*_H_ 1.22, 1.16, 1.05), one doublet methyl (*δ*_H_ 1.04), and three active hydrogens (*δ*_H_ 4.26, 4.24, 3.95). The ^13^C NMR data (Table 4) of **26** displayed 15 carbon signals, which were assigned to three oxygen-bearing carbons (*δ*_C_ 78.8, 72.9, 67.1), four methines (*δ*_C_ 52.0, 36.3, 23.8, 23.1), four methylenes (*δ*_C_ 39.4, 37.2, 27.6, 26.2), and four methyls (*δ*_C_ 30.6, 26.5, 26.4, 24.2) with the aid of HSQC experiment. The obviously upfield of H-6 (*δ*_H_ 0.13, dt, *J* = 10.3, 3.4 Hz) and H-7 (*δ*_H_ 0.57, ddd, *J* = 10.0, 8.0, 3.5 Hz) hinted **26** was a pallenane and related to 3*β*,4*β*-dihydroxypallenone [33, 34]. The C5/C3 bicyclic skeleton of **26** was established by ^1^H–^1^H COSY correlations between H-1/H-2/H-3, between H-1/H-6, H-1/H-5, and H-5/H-6. Furthermore, the ^1^H–^1^H COSY correlations from H-6/H-7/H-8/H-9/H-10/H_3_-14 and H-10/10-OH revealed a 4-hydroxypentyl were connected with C-6. HMBC correlations from H-12 (*δ*_H_ 1.05) to C-7 (*δ*_C_ 52.0), C-11 (*δ*_C_ 72.9), from H-13 (*δ*_H_ 1.16) to C-7 (*δ*_C_ 52.0), C-11 (*δ*_C_ 72.9) elucidated a 2-hydroxypropan-2-yl was connected with C-7. Moreover, HMBC correlations from H-3 (*δ*_H_ 1.34) to C-4 (*δ*_C_ 78.8), C-5 (*δ*_C_ 36.3), from H-6 (*δ*_H_ 0.13) to C-2 (*δ*_C_ 26.2) and C-4 (*δ*_C_ 78.8), from H-7 (*δ*_H_ 0.57) to C-6 (*δ*_C_ 23.1), C-8 (*δ*_C_ 27.6), C-9 (*δ*_C_ 39.4), and C-11 (*δ*_C_ 72.9), from H_3_-15 (*δ*_H_ 1.22) to C-3 (*δ*_C_ 37.2), C-4 (*δ*_C_ 78.8), C-5 (*δ*_C_ 36.3) further confirmed the planar structure (Fig. 2A). Comparing the ^1^H NMR data of H-5 (*δ*_H_ 1.13, dd, *J* = 6.0, 3.6 Hz), H-6 (*δ*_H_ 0.13, dt, *J* = 10.3, 3.4 Hz), and H-7 (*δ*_H_ 0.57, ddd, *J* = 10.0, 8.0, 3.5 Hz) with those of 3*β*,4*β*-dihydroxypallenone [33, 34] indicated **26** possessed the same H-1/H-5 *cis*, H-1/H-6 *trans*, H-5/H-6 *trans* configurations. This conclusion was confirmed by NOE correlations observed from H-7 (*δ*_H_ 0.57) to H-1 (*δ*_H_ 1.04) and H-5 (*δ*_H_ 1.13) (Fig. 3). The methyl at C-4 was determined as *β* deduced from NOE correlations observed from H-6 (*δ*_H_ 0.13) to H_3_-15 (*δ*_H_ 1.22), from H-5 (*δ*_H_ 1.13) to 4-OH (*δ*_H_ 4.24). As there were potential inaccuracies of NOE correlations as the overlapped signals of H-1 and H_3_-14. 1D and 2D NMR spectra of **26** were remeasured in pyridine-*d*_5_ to get the distinct signals of H-1, H-5, H-6 and analysis of NOE correlations (in pyridine-*d*_5_) further confirmed the configurations (Table S1, Additional file 1). Due to the configuration of methyl at C-4 differed from the congeners, **26** was named as 4-*epi*-pallenane-4*α*,10,11-triol.

The characteristic ^1^H and ^13^C NMR data (Tables 4, 5) of **27** showed it was also a pallenane sesquiterpenoid. The NMR data of **27** were similar to those of **26**, except for the presence of one methine (*δ*_C_ 35.0) instead of one oxygenated quaternary carbon as well as a doublet methyl replaced a singlet methyl. ^1^H–^1^H COSY correlations from H-4 (*δ*_H_ 2.15) to H_3_-15 (*δ*_H_ 0.99) combining with HMBC correlations between H_3_-15 (*δ*_H_ 0.99) and C-3 (*δ*_C_ 30.2), C-4 (*δ*_C_ 35.0), C-5 (*δ*_C_ 30.1) confirmed the methyl was located at C-4. (Fig. 2A). The similar ^1^H NMR data of H-5 (*δ*_H_ 1.12, dt, *J* = 6.3, 3.5 Hz), H-6 (*δ*_H_ 0.27, dt, *J* = 10.3, 3.3 Hz), and H-7 (*δ*_H_ 0.56, ddd, *J* = 10.6, 7.8, 3.5 Hz) with those of **26** suggested they possessed the identical configurations. In NOESY spectrum, the key cross peaks from H-6 (*δ*_H_ 0.27) to H_3_-15 (*δ*_H_ 0.99), from H-4 (*δ*_H_ 2.15) to H-5 (*δ*_H_ 1.12), from H-7 (*δ*_H_ 0.56) to H-1 (*δ*_H_ 0.97) and H-5 (*δ*_H_ 1.12) further indicated that H-1, H-4, H-5, and H-7 were *α*-oriented, while H-6, and H_3_-15 were *β*-oriented (Fig. 3). Finally, the structure of compound **27** was confirmed. Pallenane is a kind of rarely reported sesquiterpene with a distinctive C5/C3 bicyclic skeleton. Till now, only two pallenane congeners (3*β*,4*β*-dihydroxypallenone and 3*β*-acetoxy*-*4*β*-hydroxypallenone) were found from the plant *Pallenis spinosa* [33, 34]. The current compounds **26** and **27** were firstly obtained from streptomycete. According to the literature [33, 34], the isodaucane (**17**), oplopanane (**25**), and pallenane (**26**, **27**) skeleton metabolites were derived from the pinacol-type cadinane glycols through Wagner rearrangement in organisms (Fig. 2B). The 2-hydroxypropan-2-yl groups at C-7 in **26** and **27** were suggested as *β*-orientation according to the biogenetic way.

The characteristic ^1^H and ^13^C NMR signals implied that **28** was an eudesmane-type sesquiterpene and related to eudesmane-1*β*,6*α*,11-triol (**29**) [10]. The alterations were one more oxygenated methine replaced a methylene and a double bond replaced two methines. The HMBC correlations from H-4 (*δ*_H_ 2.39), H-7 (*δ*_H_ 2.02) to C-5 (*δ*_C_ 145.9) and H-4 (*δ*_H_ 2.39), H-7 (*δ*_H_ 2.02) to C-6 (*δ*_C_ 126.6) indicated double bond was located at C-5 and C-6, from 3-OH (*δ*_H_ 4.50) to C-2 (*δ*_C_ 35.6) and C-3 (*δ*_C_ 68.7) demonstrated an extra hydroxy was connected with C-3 (Fig. 2A). The relative configuration was determined by analysis of coupling constants and NOE correlations. The large coupling constants of H-1 (*δ*_H_ 3.03, dt, *J* = 11.6, 4.8 Hz), H-3 (*δ*_H_ 3.52, m) and H-7 (*δ*_H_ 2.02, ddd, *J* = 11.0, 6.2, 2.0 Hz) suggested that H-1, H-3, H-7 were axial orientation. While the peak shape and coupling constants of H-4 (*δ*_H_ 2.39, quint, *J* = 7.0 Hz) suggested H-4 was equatorial orientation. NOE interactions from H-3 (*δ*_H_ 3.52) to H-4 (*δ*_H_ 2.39), H-1 (*δ*_H_ 3.03), from H-6 (*δ*_H_ 5.53) to H-4 (*δ*_H_ 2.39), H-7 (*δ*_H_ 2.02), from H_3_-14 (*δ*_H_ 0.95) to 1-OH (*δ*_H_ 4.45) demonstrated that H-1, H-3, H-4, and H-7 were *α*-oriented, while 1-OH, 3-OH, H_3_-14, and H_3_-15 were *β*-oriented (Fig. 3). The configurations of chiral carbons of **28** were identified as 1*R*,3*S*,4*R*,7*R*,10*R* by comparing the experimental ECD spectrum of **28** with calculated ECD spectrum (Fig. 4G). Hence, the structure of **28** was determined as (1*R*,3*S*,4*R*,7*R*,10*R*)-eudesm-5-ene-1,3,11-triol.

The twenty-one known compounds were identified as bacaryolane B (**5**) [19], bacaryolane C (**6**) [19], caryolane-1,7*α*-diol (**7**) [35], caryolane-1,9*α*-diol (**8**) [36], 6*α*,9*α*-dihydroxy-*β*-caryolanol (**9**) [37], 6*β*,9*β*-dihydroxy-*β*-caryolanol (**10**) [37], caryolane-1,6*β*,9*α*-triol (**11**) [10], bacaryolane A (**12**) [19], 1(10)*E*,5*E*-germacradiene-2,11-diol (**16**) [26], ganodermanol L (**19**) [28], (1*α*,4*β*,5*β*,6*β*,7*β*,10*α*)-5,11-epoxy-10-cadinanol (**20**) [10], 15-hydroxy-*α*-cadinol (**21**) [29], pubinernoid C (**24**) [38], eudesmane-1*β*,6*α*,11-triol (**29**) [10], eudesmane-1*β*,6*α*,9*β*,11-tetrol (**30**) [10], ganodermanol J (**31**) [39], eudesmane-1*β*,5*α*,11-triol (**32**) [10], (2*α*,4*β*,5*β*,7*β*,10*α*)-2,5,11-eudesmanetriol (**33**) [10], *ent*-4(15)-eudesmene-1*β*,6*α*-diol (**34**) [40], 1*β*,6*α*-dihydroxy-4*β*(15)-epoxyeudesmane (**35**) [41] and 4*α*,15-epoxyeudesmane-1*β*,6*α*,11-triol (**36**) [28] by comparing their spectroscopic data with those reported in the literatures.

### Evaluation of antimicrobial activity in vitro

The isolated sesquiterpenoids which amounted to more than 3 mg were chosen to evaluate their antimicrobial activities against five bacteria and four fungi. As presented in Table S2, Additional file 1, compounds **5–7**, **9**, **12**, **31–34** inhibited the growth against *C. albicans* and *C. parapsilosis* with the MIC_80_ values ranged from 100 to 400 μg/mL. Furthermore, **34** exhibited moderate antifungal activity against *C. neoformans* and *C. gattii* with MIC values of 50 μg/mL. The caryolane sesquiterpenoids **5–7** exhibited weak antibacterial activity against *B. subtilis*, *E. faecium*, and *E. coli* with MIC values ranged from 100–200 μg/mL, respectively (Table S3, Additional file 1). Meanwhile, the other tested compounds (**10**, **11**, **13**, **16**, **19–21**, **23**, **29**, **30**, **35**, **36**) didn’t exhibit antimicrobial activity against test microorganisms at a concentration of 400 μg/mL.

### 34 inhibited *Cryptococcus* species in vitro

**Fig. 5:**
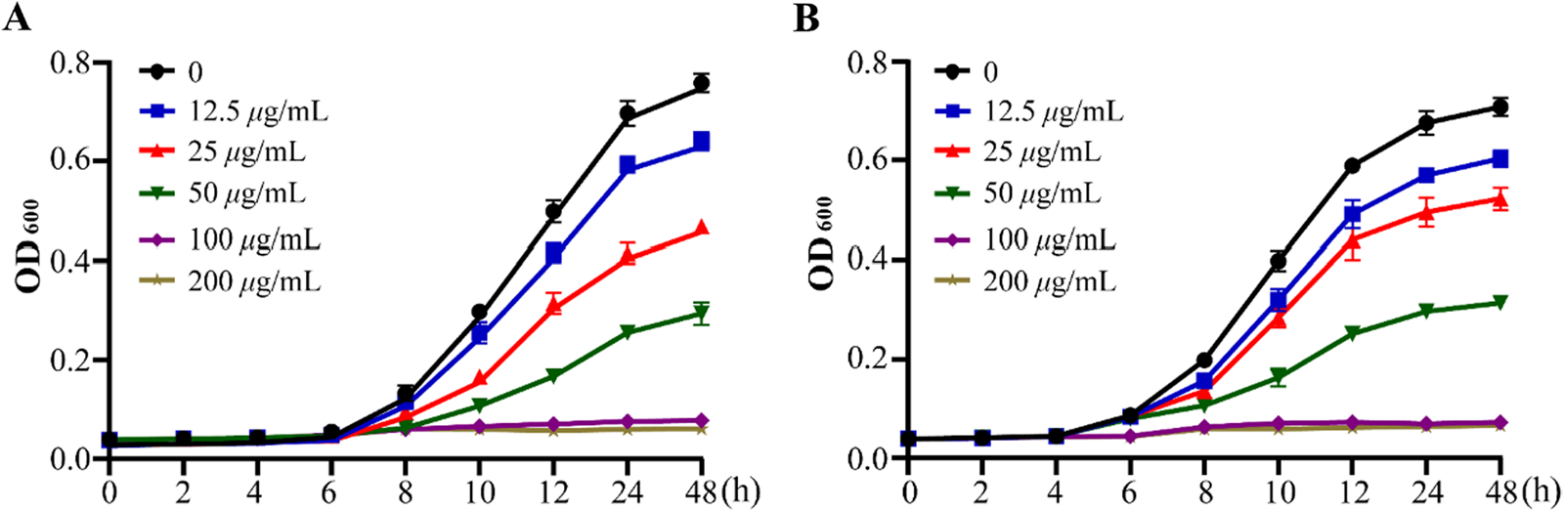
The growth curve of **34** against *C. neoformans* (**A**) and *C. gattii* (**B**). Data are presented as mean ± SD of three independent determinations

The optimal antifungal activity of **34** prompted us to further investigation its effect on *Cryptococcus* species. As shown in Fig. 5A, B, 34 consistently suppressed the growth of *Cryptococcus* species cells at all tested time points. Notably, at a concentration of 100 μg/mL, **34** could effectively eliminate almost all *Cryptococcus* species fungi. The above data demonstrated that **34** significantly inhibited the growth of *Cryptococcus* species in a time- and dose-dependent manner.

### Effect of 34 on biofilm

**Fig. 6:**
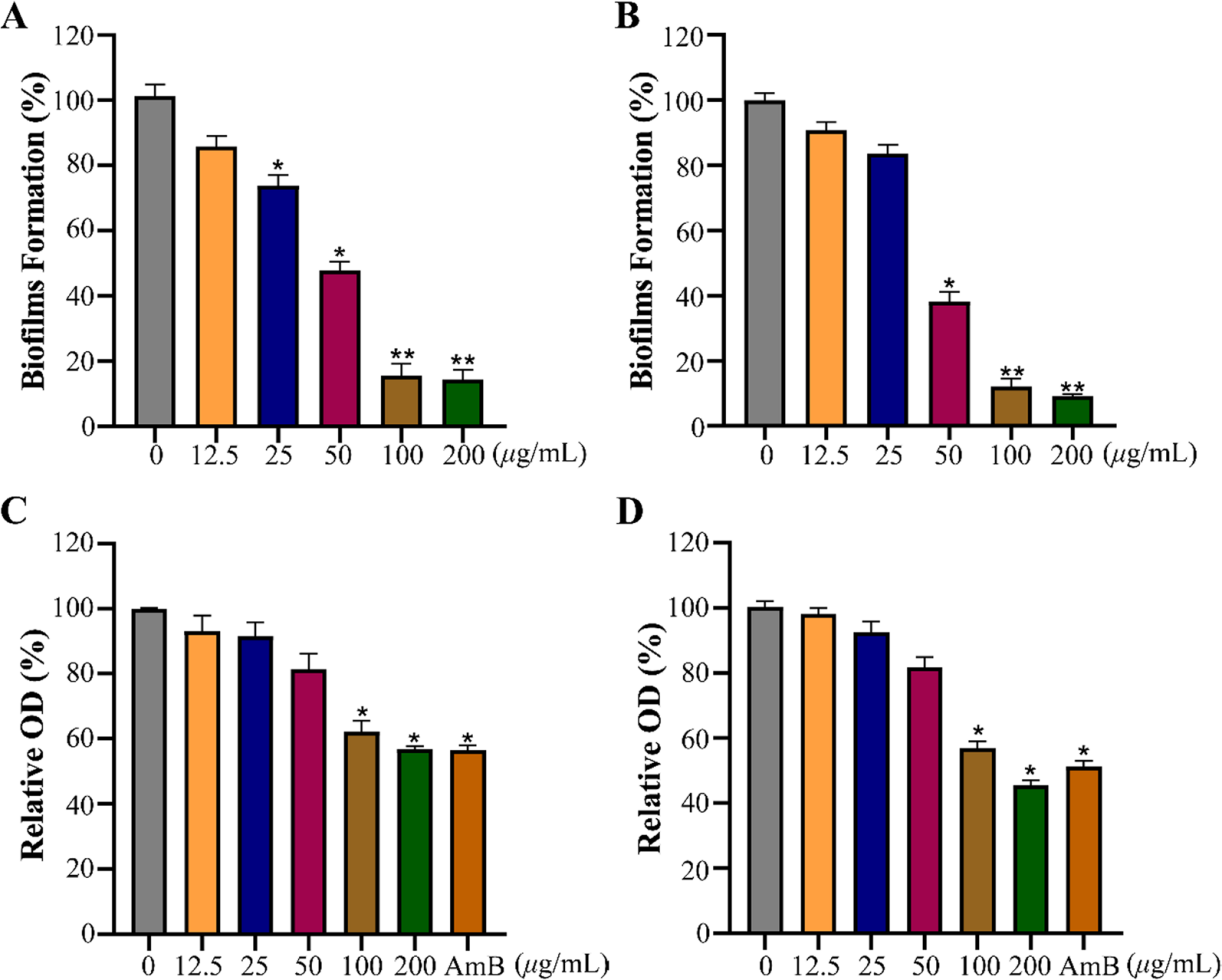
The effect of **34** on the biofilm of *Cryptococcus* species. Effect of **34** on biofilm formation of *C. neoformans* (**A**) and *C. gattii* (**B**). Effect of **34** on the preformed biofilms of *C. neoformans* (**C**) and *C. gattii* (**D**). 2 μg/mL AMB was used as positive control. Data are presented as mean ± SD of three independent determinations. *P < 0.05, **P < 0.01 vs. control group

The majority of fungal infections are attributed to biofilm, so inhibiting biofilm formation is crucial for effective antifungal treatment [42]. Thereafter, the effect of **34** was further investigated on biofilm formation and preformed biofilm of *Cryptococcus* species. As displayed in Fig. 6A, B, the formation rate of biofilms for *C. neoformans* and *C. gattii* were decreased to 47.8% and 38.3%, respectively, at a dose of 50 μg/mL. As shown in Fig. 6C, D, 34 effectively destroyed the preformed biofilms of *Cryptococcus* species at a concentration of 100 μg/mL. These results indicated that **34** could not only inhibit biofilm formation, but also destroy preformed biofilms, which has the potential source for discovering novel therapeutic agents for treatment of fungal diseases.

### 34 inhibited the adhesion of *Cryptococcus* species

**Fig. 7:**
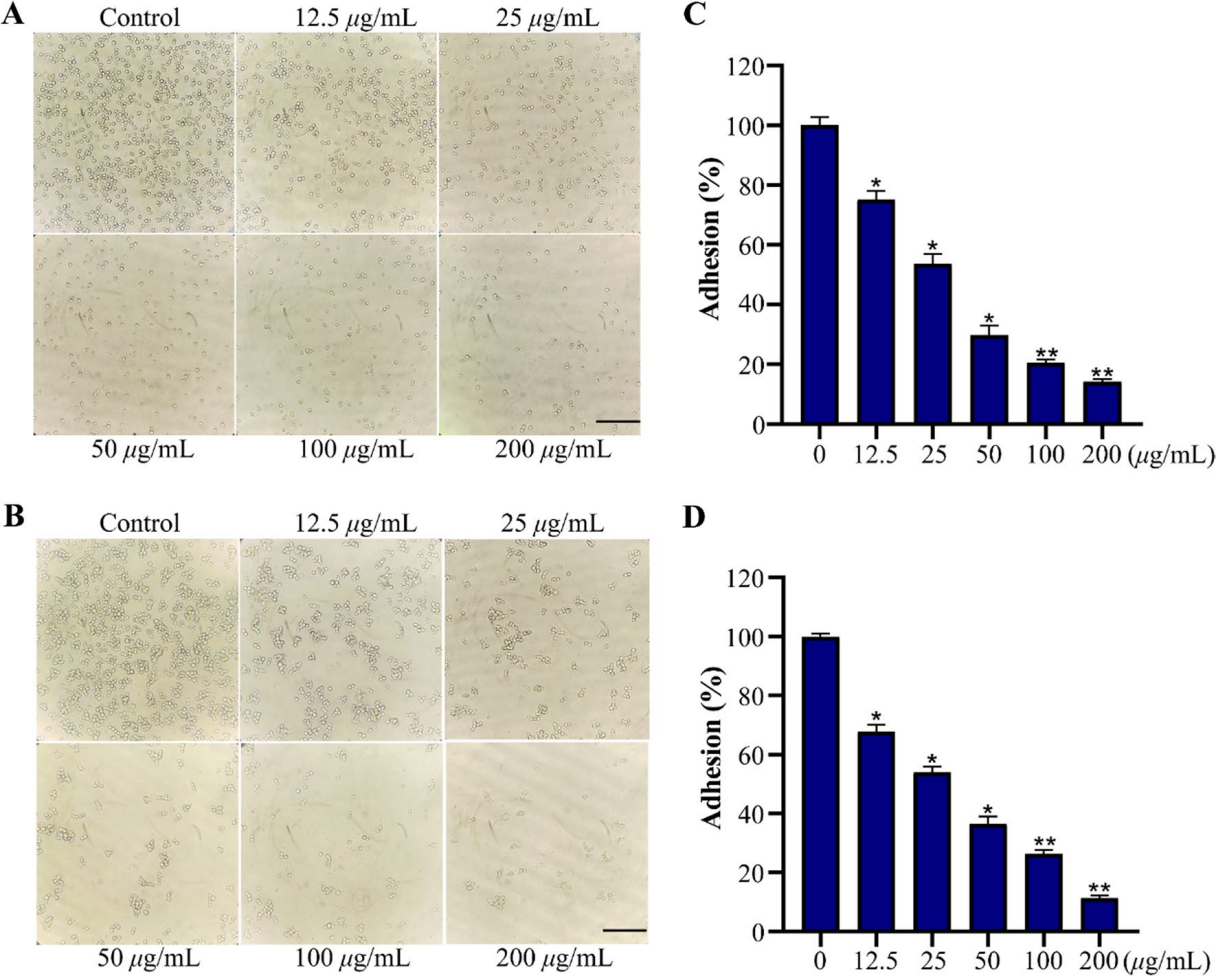
The effect of **34** on *Cryptococcus* species adhesion. The photographs of *C. neoformans* cells (**A**) and *C. gattii* cells (**B**) treated with **34** for 4 h at 37 °C; The adhesion rates of *C. neoformans* cells (**C**) and *C. gattii* cells (**D**) was measured by XTT reduction assay. The bar in panel (**A**, **B**) indicates 40 µm. Data are presented as mean ± SD of three independent determinations. *P < 0.05, **P < 0.01 vs. control group

Adhesion is the initial step in host colonization and dissemination [43]. **34** was further evaluated for its ability to inhibit the adhesion of *Cryptococcus* species. Following treatment with various concentrations of **34**, the number of adherent *Cryptococcus* sp. cells exhibited a dose-dependent reduction trend compared to the untreated group (Fig. 7A, B). The above observation was further confirmed by the results of the XTT reduction assay. As shown in Fig. 7C, D, the adhesion rates for *C. neoformans* and *C. gattii* significantly decreased from 75.2% to 14.1% and from 67.7% to 11.3%, respectively after treated with **34** (12.5 to 200 μg/mL).

## Experimental procedures

### General

Optical rotation was determined using an Anton Paar MCP200 automatic polarimeter (Graz, Austria). IR spectra were recorded with a Bruker Tensor 27 FT-IR spectrometer. A biologic MOS-450 spectra polarimeter (Biologic Science, Claix, France) was used to measured ECD spectra. NMR spectra were recorded on a Bruker Advance III-600 MHz spectrometer (Bruker, Rheinstetten, Germany). ESIMS were recorded on an Agilent 1290–6420 Triple Quadrupole LC-MS spectrometer (Santa Clara, CA, USA). HRESIMS experiments were conducted using a Bruker Micro TOF-Q mass spectrometer (Bruker Daltonics, Billerica, MA). Silica gel (100–200 mesh, 200–300 mesh, Qingdao Marine Chemical, Ltd., Qingdao, China), Sephadex LH-20 (GE Healthcare Biosciences AB, Uppsala, Sweden), YMC*GEL ODS-A (S-50 μm, 12 nm) (YMC Co., Ltd., Kyoto, Japan) were used for column chromatography. Mosher’s reagents R-MTPA-Cl and S-MTPA-Cl were purchased from Sigma Aldrich (Shanghai) Trading Co., Ltd. XTT and antimicrobial assays were analyzed using a microplate reader (BioTek Synergy H1, BioTek Instruments, Inc., Vermont, USA). The images of cells were observed directly with a microscope (Olympus IX71, Olympus, Tokyo, Japan).

### Microbial material

The producing organism was derived from fresh fecal samples excreted by healthy adult *E. maximus* living in Xishuangbanna National Nature Reserve, Xishuangbanna, Yunnan Province, China. The strain was identified to be *S. fulvorobeus* by Dr. Yi Jiang based on morphological characteristics and 16S rRNA gene sequences. The BLAST result showed that the sequence was most similar (99.89%) to the sequence of *S. fulvorobeus* (strain: NBRC 15897, GenBank accession no. AB184711). The strain (No. YIM 103582) was deposited at the Yunnan Institute of Microbiology, Yunnan University, China.

### Fermentation, extraction, and isolation

The strain was inoculated to 100 mL seed medium consisting of 4 g/L yeast extract, 4 g/L glucose, 5 g/L malt extract, 1.0 mL of multiple vitamin solution, and 1.0 mL of trace element solution at a pH of 7.2 without adjustment. The flasks were cultured for 2 days at 28 °C on a rotary shaker at 160 rpm, followed by inoculation to fermentation medium (24 g/L soluble starch, 3 g/L beef extract, 1 g/L glucose, 3 g/ L peptone, 5 g/L yeast extract, 4 g/L CaCO_3_, pH 7.0) with a 10% volume. The fermentation was incubated at 28 °C for 7 days on a rotary shaker at 160 rpm.

The completed fermentation culture (150 L) was centrifuged (4000 rpm, 5 min) to separate into supernatant and mycelium, and the supernatant was extracted with ethyl acetate three times and evaporated to yield a crude extract 60 g. The dried extract was subjected to a silica gel column chromatography eluting with a CH_2_Cl_2_-MeOH solvent system (from 100:1 to 30:1, 10:1, and finally 1:1) to yield five fractions Fr.1–5. Fraction 1 was subjected to Sephadex LH-20 chromatography (MeOH) to produce three subfractions Fr.1.1-Fr.1.3. Fr.1.3 was subjected to silica gel column chromatography (P. ether-EtOAc 10:1) to yield three subfractions Fr.1.3.1-Fr.1.3.9. Fr.1.3.3 was isolated through ODS column chromatography MeOH-H_2_O (60:40) to yield compounds **5** (4.6 mg), **6** (3.0 mg), and **7** (14.3 mg). Fr.1.3.5 was purified by silica gel column chromatography (P. ether-EtOAc 7:1) to afford compound **12** (8.8 mg), **20** (26.7 mg), **25** (2.0 mg), and **35** (5.0 mg). Fraction 2 was subjected to Sephadex LH-20 chromatography (MeOH) to obtain three subfractions Fr.2.1-Fr.2.3. Fr.2.3 was separated by silica gel column chromatoraphy (P. ether-EtOAc 4:1), followed by eluting with MeOH-H_2_O (40:60) using ODS column chromatography to afford compounds **8** (2.2 mg), **16** (4.0 mg), **34** (16.6 mg), and **36** (6.2 mg). Fraction 3 was subjected to silica gel column chromatography (CH_2_Cl_2_-MeOH 50:1) to yield seven subfractions Fr. 3.1-Fr. 3.7. Fr. 3.1was put on an ODS column and eluted with MeOH-H_2_O (40:60) to yield three subfractions Fr. 3.1.1-Fr. 3.1.3. Compounds **2** (2.5 mg), **18** (2.3 mg), **19** (57.8 mg), **29** (23.3 mg), and compound **33** (16.0 mg) were obtained from Fr.3.1.1 through a silica gel column chromatography (P. ether-EtOAc 6:1). Fr.3.3 was subjected to ODS column chromatography, eluting with MeOH-H_2_O (45:55) to yield nine subfractions Fr.3.3.1-Fr.3.3.9. Fr.3.3.3 was fractionated by silica gel column chromatography (CH_2_Cl_2_-MeOH 50:1) to give compounds **9** (4.0 mg), **11** (15.0 mg), and **26** (1.6 mg). Fr.3.3.4 was separated by silica gel column chromatography (P. ether-EtOAc 2:1), then further purified by silica gel column chromatography (CH_2_Cl_2_-MeOH 50:1) to give compounds **1** (2.8 mg), **15** (2.3 mg), **17** (2.6 mg), **30** (23.7 mg), and **31** (3.2 mg). Compounds **21** (6.2 mg) and **32** (5.8 mg) were obtained from Fr.3.3.5 through a silica gel column chromatography (CH_2_Cl_2_-MeOH 60:1). Similarly, compounds **13** (5.2 mg) and **14** (2.6 mg) were isolated from Fr. 3.3.7 through a silica gel column chromatography (CH_2_Cl_2_-MeOH 60:1). Fr. 3.3.9 was purified by silica gel column chromatography (P. ether-EtOAc 2:1) to afford compounds **22** (2.3 mg) and **24** (2.0 mg). Fr.3.5 was separated by ODS column chromatography and eluted with MeOH-H_2_O (30:70) to yield six fractions Fr.3.5.1–3.5.6. Fr.3.5.2 was purified by silica gel column chromatography (EtOAc–MeOH 40:1) to afford compounds **3** (2.0 mg) and **4** (2.6 mg). Fr.3.5.4 was purified by silica gel column chromatography (CH_2_Cl_2_-MeOH 25:1) to afford compounds **10** (3.8 mg) and **27** (1.5 mg). Fr.3.5.5 was firstly subjected to silica gel column chromatography (CH_2_Cl_2_-MeOH 25:1), then purified by silica gel column chromatography (P. ether-EtOAc 2:1) to give compounds **23** (3.2 mg) and **28** (2.0 mg).

### Spectroscopic data of compounds

#### (1*S*,2*R*,4*S*,5*S*,8*R*)-9-Oxocaryolane-1,13-diol (1)

Colorless oil; $[\alpha ]_{\text{D}}^{20}$ + 60.0 (*c* 0.20, MeOH); IR (film) *ν*_max_ 3727, 3382, 2938, 2866, 1699, 1455, 1054, 1033, 1013 cm^−1^; CD (0.5 mg/mL, MeOH) *λ*_max_ (Δ*ε*) 202 (− 0.48), 294 (+ 2.04) nm; ^1^H and ^13^C NMR see Tables 1 and 2; HRESIMS *m/z* 275.1623 [M+Na]^+^ (calcd for C_15_H_24_NaO_3_^+^, 275.1618).

#### (1*S*,2*R*,4*R*,5*S*,8*R*)-Caryolane-1,14-diol (2)

Colorless oil; $[\alpha ]_{\text{D}}^{20}$ − 53.0 (*c* 0.30, MeOH); IR (film) *ν*_max_ 3726, 3347, 2926, 2867, 1456, 1053, 1033 cm^−1^; ^1^H and ^13^C NMR see Tables 1 and 2; HRESIMS *m/z* 239.2021 [M+H]^+^ (calcd for C_15_H_27_O_2_^+^, 239.2006).

#### (1*S*,2*R*,4*R*,5*S*,8*R*,9*S*)-Caryolane-1,9,14-triol (3)

Colorless oil; $[\alpha ]_{\text{D}}^{20}$ − 40.5 (*c* 0.20, MeOH); IR (film) *ν*_max_ 3355, 2940, 2865, 1457, 1055, 1033, 1018 cm^−1^; ^1^H and ^13^C NMR see Tables 1 and 2; HRESIMS *m/z* 277.1780 [M+Na]^+^ (calcd for C_15_H_26_NaO_3_^+^, 277.1774).

#### Caryolane-1,6*α*,10*α*-triol (4)

Colorless solid; $[\alpha ]_{\text{D}}^{20}$ − 54.0 (*c* 0.37, MeOH); IR (film) *ν*_max_ 3348, 2928, 2866, 1461, 1363, 1109, 1040, 1004 cm^−1^; ^1^H and ^13^C NMR see Tables 1 and 2; HRESIMS *m/z* 277.1778 [M+Na]^+^ (calcd for C_15_H_26_NaO_3_^+^, 277.1774).

#### (2*S*,4*S*,7*S*,8*S*)-1(10)*E*,5*E*-Germacradiene-2,8,11-triol (13)

Colorless oil; $[\alpha ]_{\text{D}}^{20}$ − 80.5 (*c* 0.20, MeOH); IR (film) *ν*_max_ 3315, 2968, 2930, 1668, 1382, 1046, 1010 cm^−1^; CD (0.5 mg/mL, MeOH) *λ*_max_ (Δ *ε*) 231 (− 25.77) nm; ^1^H and ^13^C NMR see Tables 2 and 3; HRESIMS *m/z* 277.1780 [M+Na]^+^ (calcd for C_15_H_26_NaO_3_^+^, 277.1774).

#### (2*S*,4*S*,7*R*)-1(10)*E*,5*E*-Germacradiene-2,11-diol 2-methyl ether (14)

Colorless oil; $[\alpha ]_{\text{D}}^{20}$ − 112.2 (*c* 0.50, MeOH); IR (film) *ν*_max_ 3728, 2970, 2930, 1447, 1381, 1088, 934 cm^−1^; CD (0.5 mg/mL, MeOH) *λ*_max_ (Δ *ε*) 225 (− 9.42), 290 (+ 0.38) nm; ^1^H and ^13^C NMR see Tables 2 and 3; HRESIMS *m/z* 275.1986 [M+Na]^+^ (calcd for C_16_H_28_NaO_2_^+^, 275.1982).

#### (2*S*,4*R*,7*S*,8*S*)-1(10)*E*,5*E*-Germacradiene-2,4,8,11-tetraol 2-acetate (15)

White powder; $[\alpha ]_{\text{D}}^{20}$ − 120.5 (*c* 0.20, MeOH); IR (film) *ν*_max_ 3348, 2973, 2929, 1730, 1373, 1244, 1022 cm^−1^; CD (0.5 mg/mL, MeOH) *λ*_max_ (Δ *ε*) 200 (+ 29.35), 231 (− 9.42) nm; ^1^H and ^13^C NMR see Tables 2 and 3; HRESIMS *m/z* 335.1833 [M+Na]^+^ (calcd for C_17_H_28_NaO_5_^+^, 335.1829).

#### (1*α*,3*α*,4*β*,5*α*,6*α*,7*β*,9*β*)-6,11-Epoxyisodaucane-3,9-diol (17)

White powder; $[\alpha ]_{\text{D}}^{20}$ + 13.3 (*c* 0.30, MeOH); IR (film) *ν*_max_ 3360, 2927, 2869, 1460, 1366, 1236, 1049, 1028 cm^−1^; ^1^H and ^13^C NMR see Tables 3 and 4; HRESIMS *m/z* 277.1788 [M+Na]^+^ (calcd for C_15_H_26_NaO_3_^+^, 277.1774).

#### 8*α*-Hydroxyganodermanol L (18)

White powder; $[\alpha ]_{\text{D}}^{20}$ − 40.6 (*c* 0.20, MeOH); IR (film) *ν*_max_ 3355, 2966, 2927, 1461, 1379, 1172, 1046 cm^−1^; ^1^H and ^13^C NMR see Tables 3 and 4; HRESIMS *m/z* 293.1729 [M+Na]^+^ (calcd for C_15_H_26_NaO_4_^+^, 293.1723).

#### (1*S*,2*S*,6*R*,7*S*,10*R*)-Cadinane-2,10,15-triol (22)

Yellow oil; $[\alpha ]_{\text{D}}^{20}$ − 40.2 (*c* 0.30, MeOH); IR (film) *ν*_max_ 3312, 2959, 2932, 2871, 1462, 1378, 1125, 1069, 1016 cm^−1^; CD (0.25 mg/mL, MeOH) *λ*_max_ (Δ *ε*) 194 (+ 9.63), 217 (− 9.01), 237 (+ 1.85) nm; ^1^H and ^13^C NMR see Tables 4 and 5; HRESIMS *m/z* 277.1777 [M+Na]^+^ (calcd for C_15_H_26_NaO_3_^+^, 277.1774).

#### (1*R*,3*S*,6*R*,7*S*,9*S*,10*R*)-3,9-Dihydroxyepicubenol (23)

Yellow oil; $[\alpha ]_{\text{D}}^{20}$ − 16.0 (*c* 0.50, MeOH); IR (film) *ν*_max_ 3354, 2960, 2877, 1450, 1659, 1369, 1060, 1030, 1007 cm^−1^; CD (0.25 mg/mL, MeOH) *λ*_max_ (Δ *ε*) 191 (− 29.60), 213 (− 11.77), 234 (+ 2.98) nm; ^1^H and ^13^C NMR see Tables 4 and 5; HRESIMS *m/z* 277.1775 [M+Na]^+^ (calcd for C_15_H_26_NaO_3_^+^, 277.1774).

#### Oplopanane-4,10*α*,11-triol (25)

White amorphous power; $[\alpha ]_{\text{D}}^{20}$ − 50.0 (*c* 0.24, MeOH); IR (film) *ν*_max_ 3331, 2925, 2862, 1597, 1454, 1261, 1069, 1014 cm^−1^; ^1^H and ^13^C NMR see Tables 4 and 5; HRESIMS *m/z* 279.1937 [M+Na]^+^ (calcd for C_15_H_28_NaO_3_^+^, 279.1931).

#### 4-*epi*-Pallenane-4*α*,10,11-triol (26)

Colorless oil; $[\alpha ]_{\text{D}}^{20}$ − 202.0 (*c* 0.10, MeOH); IR (film) *ν*_max_ 3358, 2965, 2867, 1600, 1458, 1372, 1185, 1124, 1003 cm^−1^; ^1^H and ^13^C NMR see Tables 4 and 5; HRESIMS *m/z* 279.1936 [M+Na]^+^ (calcd for C_15_H_28_NaO_3_^+^, 279.1931).

#### 4-*epi*-Pallenane-10,11-diol (27)

Colorless oil; $[\alpha ]_{\text{D}}^{20}$ − 80.6 (*c* 0.20, MeOH); IR (film) *ν*_max_ 3349, 2927, 2865, 1602, 1457, 1372, 1126, 1082 cm^−1^; ^1^H and ^13^C NMR see Tables 4 and 5; HRESIMS *m/z* 223.2039 [M−H_2_O+H]^+^ (calcd for C_15_H_27_O^+^, 223.2056).

#### (1*R*,3*S*,4*R*,7*R*,10*R*)-Eudesm-5-ene-1,3,11-triol (28)

Colorless oil; $[\alpha ]_{\text{D}}^{20}$ − 40.4 (*c* 0.20, MeOH); IR (film) *ν*_max_ 3350, 2971, 2936, 1659, 1468, 1376, 1145, 1066, 1007 cm^−1^; CD (0.5 mg/mL, MeOH) *λ*_max_ (Δ*ε*) 206 (− 16.93), 229 (+ 0.46) nm; ^1^H and ^13^C NMR see Tables 4 and 5; HRESIMS *m/z* 277.1779 [M+Na]^+^ (calcd for C_15_H_26_NaO_3_^+^, 277.1774).

### Esterification using Mosher′s reagent

The 1 mg sample of compound **1** was dissolved in 0.5 mL of pyridine-*d*_5_ and subsequently transferred into a pristine NMR tube. Then, the solution was subjected to treatment with (*R*)-MTPA-Cl (5 μL) for 12 h to obtained (*S*)-MTPA ester of **1** (**1a**). The (*R*)-MTPA ester of **1** (**1b**) was prepared by the same process. Subsequently, the tubes were directly employed for ^1^H-NMR measurements. The (*S*)-MTPA esters (**2a** and **3a**) as well as (*R*)-MTPA esters (**2b** and **3b**) of compounds **2** and **3** were prepared in a completely analog manner.

### ECD calculations

The ECD calculations of compounds **1**, **13–15**, **22**, **23**, and **28** were performed with Gaussian 09 [44]. The CONFLEX software was used to perform a stable conformational analysis of all enantiomers, employing the MMFF94S molecular force field and an energy cut off of 3 kcal/mol. The selected stable conformers were further optimized using the Gaussian 09 program package at the B3LYP/6-31G+(d) level of theory. Then, the ECD was theoretically calculated at the B3LYP/6-311 g++ (2d, p) level in a methanol solution using the PCM model. SpecDis 1.51 software was used to generate the global theoretical ECD curve based on the Boltzmann weights of each conformer.

### Antimicrobial assay

The antimicrobial activities were evaluated using a microbroth dilution method to determine the minimum inhibitory concentrations (MICs) [9]. The tested strains included five bacteria (*Bacillus subtilis* ATCC 6633, *Staphylococcus aureus* ATCC 25923, *Enterococcus faecium* ATCC19434, *Escherichia coli* ATCC 25922, *Pseudomonas aeruginosa* ATCC27853), as well as four fungi (*Candida albicans* ATCC MYA-2867, *C. parapsilosis* ATCC 22019, and *Cryptococcus neoformans* ATCC 208821, *C. gattii* CGMCC 2.3159). Antibacterial and antifungal tests were performed in Luria–Bertani and RPMI-1640 broth, respectively. Test compounds were dissolved in DMSO and twofold serially diluted to six different concentrations (40.0–1.25 mg/mL). Each well of the 96-well microtiter plates was added with 1 μL of test sample solutions and 100 μL of suspensions, which contained a concentration of 1 × 10^6^ cfu/mL for bacteria (2 × 10^3^ cfu/mL for fungi). The plates were subsequently incubated for 24 h at 28 °C for bacteria, 48 h at 28 °C for fungi. The XTT reduction assay was performed as described in the literature [45] and the absorbance at 490 nm was measured using a microplate reader for each well. The MIC was defined as the minimum concentration of the antimicrobial agent that completely inhibited visual growth of a microorganism, while the MIC_80_ was defined as the minimum concentration of the antimicrobial agent that inhibited 80% of the visible growth of a microorganism. Ciprofloxacin and amphotericin B were used as positive controls against bacteria and fungi, respectively.

### Growth curve assay

The growth curve of *Cryptococcus* species was conducted according to the previously described method [45]. Briefly, the fungal suspensions were normalized to in the YPD liquid medium. Then, 100 μL fungal suspensions (1 × 10^6^ cfu/mL) and different concentrations of compound **34** were inoculated into 96-well plate. The final concentrations of **34** were 12.5, 25, 50, 100 or 200 μg/mL. After incubating for 2, 4, 6, 8, 10, 12 and 24 h at 37 °C, the absorbance at 600 nm was determined by microplate reader (BioTek Synergy H1, BioTek Instruments, Inc., Vermont, USA).

### Biofilm formation assay

The strains *C. neoformans* and *C. gattii* were selected to investigate the anti-biofilm activity of compound **34**. Biofilm formation was assessed by XTT reduction assay in 96-well plate [45]. Briefly, 100 μL of fungal suspensions (1 × 10^6^ cfu/mL) were added to 96-well plates with varying different concentrations of **34** (12.5, 25, 50, 100 or 200 μg/mL). After incubation for 24 h at 30 °C, the plate was gently washed twice with sterile phosphate-buffered saline (PBS) to remove free-floating fungal cells. The biofilm of *Cryptococcus* species was assayed by XTT reduction assay.

### Preformed biofilms assay

To evaluate the potential ability of compound **34** to disrupt preformed biofilms was assessed according to previously reported method [45]. 100 μL of fungal suspensions (1 × 10^6^ cfu/mL) were dispensed into 96-well plate and cultured for 24 h at 37 °C. Then, the resulting preformed biofilm were washed twice with sterile PBS and treated with various concentrations of **34** (12.5, 25, 50, 100 or 200 μg/mL) or 2 μg/mL AMB. The plates were further incubated for 24 h, and then XTT reduction assay was conducted as described previously.

### Adhesion assay

The impact of **34** on *Cryptococcus* species adhesion on 96-well plate was examined by XTT reduction assay according to a previously described method [45]. 100 μL suspensions of *C. neoformans* and *C. gattii* (1 × 10^6^ cfu/mL) were respectively inoculated into 96-well plate, along with 1 μL of compound **34** at different concentrations (12.5, 25, 50, 100 or 200 μg/mL) and incubated at 37 °C for 4 h without shaking. Then, the cell supernatants were discarded and the plate was gently washed three times with sterile phosphate-buffered saline (PBS) to remove non-adherent cells. Then, the adherent *C. neoformans* and *C. gattii* were directly observed under a microscope in bright field mode and detected by XTT reduction assay.

## Conclusion

In summary, we report the discovery of thirty-six structurally diverse sesquiterpenoids, including fifteen new compounds (**1–4**, **13–15**, **17**, **18**, **22**, **23**, **25–28**), along with twenty-one known analogues. These compounds featured eight distinctive carbon skeletons: caryolane, germacrane, cadinane, epicubenol, isaodaucane, oplopanane, pallenane, and eudesmane. It has been proven that *Streptomyces* possesses unparalleled ability to produce structurally diverse and novel secondary metabolites. Notably, parenane is a rare sesquiterpene with a unique C5/C3 bicyclic skeleton, which was first discovered in microorganisms. To our knowledge, *S. fulvorobeus* is the first actinomycete to produce such a substantial quantity of sesquiterpenoids. Additionally, the isolated sesquiterpenoid **34** exhibited optimal antifungal activity against *C. neoformans* and *C. gattii* with MIC values of 50 μg/mL. Further experiments showed that **34** significantly inhibited biofilm formation, destroyed the preformed biofilm of fungi, and prevented adhesion of *Cryptococcus* species. This work not only enriches the structural diversity of bacterial terpenoids but also provides support for the genetic capacity of actinomycetes to synthesize a diverse array of terpenoids.

## Supplementary Information

Additional file 1: The antimicrobial activity of some compounds, HRESI-MS, 1D and 2D NMR spectra of new compounds **1–4**, **13–15**, **17**, **18**, **22**, **23**, **25–28**, experimental ECD spectra and calculated ECD spectra of compound **12**.

## Data Availability

Data will be made available on request.
