# Supplementary material for: Discovery of structurally diverse sesquiterpenoids from Streptomyces fulvorobeus isolated from Elephas maximus feces and their antifungal activities
Source: Nat Prod Bioprospect. 2024 Dec 2;14(1):61. doi: 10.1007/s13659-024-00481-9 (PMC11609139; doi:10.1007/s13659-024-00481-9)
Supplement: Supplementary file 1 — Additional file 1: The antimicrobial activity of some compounds, HRESI-MS, 1D and 2D NMR spectra of new compounds 1–4, 13–15, 17, 18, 22, 23, 25–28, experimental ECD spectra and calculated ECD spectra of compound 12. [file 13659_2024_481_MOESM1_ESM.pdf]

Supplementary Information

Discovery of structurally diverse sesquiterpenoids from *Streptomyces fulvorobeus* isolated from *Elephas maximus* feces and their antifungal activities

Lu Cao[1], Jun-Feng Tan[1], Zeng-Guang Zhang[1], Jun-Wei Yang[1], Yu Mu[1], Zhi-Long Zhao[2], Yi Jiang[3*], Xue-Shi Huang[1*] and Li Han[1*]

[1] Institute of Microbial Pharmaceuticals, College of Life and Health Sciences, Northeastern University, Shenyang, 110819, China

[2] Pharmacological Laboratory, Liaoning Provincial Institute of Drug Inspection and Testing, Shenyang, 110036, China

[3] Yunnan Institute of Microbiology, Yunnan University, Kunming, 650091, China

[*]Correspondence:

Yi Jiang

jiangyi@ynu.edu.cn

Xue-Shi Huang

huangxs@mail.neu.edu.cn

Li Han

hanli@mail.neu.edu.cn.

Table S1. ^{1}H NMR (600 MHz) and ^{13}C NMR (150 MHz) data for compound **26** in pyridine-d_5

Table S2. Antifungal activities of test compounds (MIC μg/mL, MIC_{80} μg/mL)

Table S3. Antibacterial activities of compounds **5**−**7** (MIC μg/mL)

Figure S1. HRESI-MS spectrum of the new compound **1**

Figure S2. ^{1}H NMR (600 MHz, DMSO-d_6) spectrum of the new compound **1**

Figure S3. ^{13}C NMR (150 MHz, DMSO-d_6) spectrum of the new compound **1**

Figure S4. HSQC spectrum of the new compound **1**

Figure S5. HMBC spectrum of the new compound **1**

Figure S6. COSY spectrum of the new compound **1**

Figure S7. NOESY spectrum of the new compound **1**

Figure S8. ^{1}H NMR (600 MHz, in pyridine-d_5) spectrum of the new compound **1**

Figure S9. ^{1}H NMR spectrum of (*S*)-MTPA ester (**1a**) in pyridine-d_5

Figure S10. ^{1}H NMR spectrum of (*R*)-MTPA ester (**1b**) in pyridine-d_5

Figure S11. Comparison of ^{1}H NMR spectra of (*S*)-MTPA ester (**1a**) and (*R*)-MTPA ester (**1b**) in pyridine-d_5

Figure S12. HRESI-MS spectrum of the new compound **2**

Figure S13. ^{1}H NMR (600 MHz, DMSO-d_6) spectrum of the new compound **2**

Figure S14. ^{13}C NMR (150 MHz, DMSO-d_6) spectrum of the new compound **2**

Figure S15. HSQC spectrum of the new compound **2**

Figure S16. HMBC spectrum of the new compound **2**

Figure S17. COSY spectrum of the new compound **2**

Figure S18. NOESY spectrum of the new compound **2**

Figure S19. ^{1}H NMR (600 MHz, in pyridine-d_5) spectrum of the new compound **2**

Figure S20. ^{1}H NMR spectrum of (*S*)-MTPA ester (**2a**) in pyridine-d_5

Figure S21. ^{1}H NMR spectrum of (*R*)-MTPA ester (**2b**) in pyridine-d_5

Figure S22. Comparison of ^{1}H NMR spectra of (*S*)-MTPA ester (**2a**) and (*R*)-MTPA ester (**2b**) in pyridine-d_5

Figure S23. HRESI-MS spectrum of the new compound **3**

Figure S24. ^{1}H NMR (600 MHz, DMSO-d_6) spectrum of the new compound **3**

Figure S25. ^{13}C NMR (150 MHz, DMSO-d_6) spectrum of the new compound **3**

Figure S26. HSQC spectrum of the new compound **3**

Figure S27. HMBC spectrum of the new compound **3**

Figure S28. COSY spectrum of the new compound **3**

Figure S29. NOESY spectrum of the new compound **3**

Figure S30. ^{1}H NMR (600 MHz, in pyridine-d_5) spectrum of the new compound **3**

Figure S31. ^{1}H NMR spectrum of (*S*)-MTPA ester (**3a**) in pyridine-d_5

Figure S32. ^{1}H NMR spectrum of (*R*)-MTPA ester (**3b**) in pyridine-d_5

Figure S33. Comparison of ^{1}H NMR spectra of (*S*)-MTPA ester (**3a**) and (*R*)-MTPA ester (**3b**) in pyridine-d_5

Figure S34. HRESI-MS spectrum of the new compound **4**

Figure S35. ^{1}H NMR (600 MHz, DMSO-d_6) spectrum of the new compound **4**

Figure S36. ^{13}C NMR (150 MHz, DMSO-d_6) spectrum of the new compound **4**

Figure S37. HSQC spectrum of the new compound **4**

Figure S38. HMBC spectrum of the new compound **4**

Figure S39. COSY spectrum of the new compound **4**

Figure S40. NOESY spectrum of the new compound **4**

Figure S41. HRESI-MS spectrum of the new compound **13**

Figure S42. ^{1}H NMR (600 MHz, DMSO-d_6) spectrum of the new compound **13**

Figure S43. ^{13}C NMR (150 MHz, DMSO-d_6) spectrum of the new compound **13**

Figure S44. HSQC spectrum of the new compound **13**

Figure S45. HMBC spectrum of the new compound **13**

Figure S46. COSY spectrum of the new compound **13**

Figure S47. NOESY spectrum of the new compound **13**

Figure S48. HRESI-MS spectrum of the new compound **14**

Figure S49. ^{1}H NMR (600 MHz, DMSO-d_6) spectrum of the new compound **14**

Figure S50. ^{13}C NMR (150 MHz, DMSO-d_6) spectrum of the new compound **14**

Figure S51. HSQC spectrum of the new compound **14**

Figure S52. HMBC spectrum of the new compound **14**

Figure S53. COSY spectrum of the new compound **14**

Figure S54. NOESY spectrum of the new compound **14**

Figure S55. HRESI-MS spectrum of the new compound **15**

Figure S56. ^{1}H NMR (600 MHz, DMSO-d_6) spectrum of the new compound **15**

Figure S57. ^{13}C NMR (150 MHz, DMSO-d_6) spectrum of the new compound **15**

Figure S58. HSQC spectrum of the new compound **15**

Figure S59. HMBC spectrum of the new compound **15**

Figure S60. COSY spectrum of the new compound **15**

Figure S61. NOESY spectrum of the new compound **15**

Figure S62. HRESI-MS spectrum of the new compound **17**

Figure S63. ^{1}H NMR (600 MHz, DMSO-d_6) spectrum of the new compound **17**

Figure S64. ^{13}C NMR (150 MHz, DMSO-d_6) spectrum of the new compound **17**

Figure S65. HSQC spectrum of the new compound **17**

Figure S66. HMBC spectrum of the new compound **17**

Figure S67. COSY spectrum of the new compound **17**

Figure S68. NOESY spectrum of the new compound **17**

Figure S69. HRESI-MS spectrum of the new compound **18**

Figure S70. ^{1}H NMR (600 MHz, DMSO-d_6) spectrum of the new compound **18**

Figure S71. ^{13}C NMR (150 MHz, DMSO-d_6) spectrum of the new compound **18**

Figure S72. HSQC spectrum of the new compound **18**

Figure S73. HMBC spectrum of the new compound **18**

Figure S74. COSY spectrum of the new compound **18**

Figure S75. NOESY spectrum of the new compound **18**

Figure S76. HRESI-MS spectrum of the new compound **22**

Figure S77. ^{1}H NMR (600 MHz, DMSO-d_6) spectrum of the new compound **22**

Figure S78. ^{13}C NMR (150 MHz, DMSO-d_6) spectrum of the new compound **22**

Figure S79. HSQC spectrum of the new compound **22**

Figure S80. HMBC spectrum of the new compound **22**

Figure S81. COSY spectrum of the new compound **22**

Figure S82. NOESY spectrum of the new compound **22**

Figure S83. HRESI-MS spectrum of the new compound **23**

Figure S84. ^{1}H NMR (600 MHz, DMSO-d_6) spectrum of the new compound **23**

Figure S85. ^{13}C NMR (150 MHz, DMSO-d_6) spectrum of the new compound **23**

Figure S86. HSQC spectrum of the new compound **23**

Figure S87. HMBC spectrum of the new compound **23**

Figure S88. COSY spectrum of the new compound **23**

Figure S89. NOESY spectrum of the new compound **23**

Figure S90. HRESI-MS spectrum of the new compound **25**

Figure S91. ^{1}H NMR (600 MHz, DMSO-d_6) spectrum of the new compound **25**

Figure S92. ^{13}C NMR (150 MHz, DMSO-d_6) spectrum of the new compound **25**

Figure S93. HSQC spectrum of the new compound **25**

Figure S94. HMBC spectrum of the new compound **25**

Figure S95. COSY spectrum of the new compound **25**

Figure S96. NOESY spectrum of the new compound **25**

Figure S97. HRESI-MS spectrum of the new compound **26**

Figure S98. ^{1}H NMR (600 MHz, DMSO-d_6) spectrum of the new compound **26**

Figure S99. ^{13}C NMR (150 MHz, DMSO-d_6) spectrum of the new compound **26**

Figure S100. HSQC spectrum of the new compound **26**

Figure S101. HMBC spectrum of the new compound **26**

Figure S102. COSY spectrum of the new compound **26**

Figure S103. NOESY spectrum of the new compound **26**

Figure S104. ^{1}H NMR (600 MHz, in pyridine-d_5) spectrum of the new compound **26**

Figure S105. ^{13}C NMR (150 MHz, in pyridine-d_5) spectrum of the new compound **26**

Figure S106. HSQC spectrum of the new compound **26** in pyridine-d_5

Figure S107. HMBC spectrum of the new compound **26** in pyridine-d_5

Figure S108. COSY spectrum of the new compound **26** in pyridine-d_5

Figure S109. NOESY spectrum of the new compound **26** in pyridine-d_5

Figure S110. HRESI-MS spectrum of the new compound **27**

Figure S111. ^{1}H NMR (600 MHz, DMSO-d_6) spectrum of the new compound **27**

Figure S112. ^{13}C NMR (150 MHz, DMSO-d_6) spectrum of the new compound **27**

Figure S113. HSQC spectrum of the new compound **27**

Figure S114. HMBC spectrum of the new compound **27**

Figure S115. COSY spectrum of the new compound **27**

Figure S116. NOESY spectrum of the new compound **27**

Figure S117. HRESI-MS spectrum of the new compound **28**

Figure S118. ^{1}H NMR (600 MHz, DMSO-d_6) spectrum of the new compound **28**

Figure S119. ^{13}C NMR (150 MHz, DMSO-d_6) spectrum of the new compound **28**

Figure S120. HSQC spectrum of the new compound **28**

Figure S121. HMBC spectrum of the new compound **28**

Figure S122. COSY spectrum of the new compound **28**

Figure S123. NOESY spectrum of the new compound **28**

Figure S124. Experimental ECD spectra and calculated ECD spectra of compound **12**

Table S1 ^{1}H NMR (600 MHz) and ^{13}C NMR (150 MHz) data for compound **26** in pyridine-d_5

no.	δ_C,type	δ_H (J in Hz)		HMBC	NOESY
1	25.4, CH	1.44, m			H-7
2	27.6, CH_2	2.25, m	1.80, m		
3	38.9, CH_2	1.81, m	1.37, m		
4	80.3, C				
5	38.1, CH	1.77, m		C-1, C-3, C-4, C-6	H-7
6	24.8, CH	0.55, dt (10.2, 3.3)		C-2, C-4	H-15
7	53.7, CH	1.08, ddd (10.2, 8.3, 3.7)		C-6, C-9, C-11, C-12	H-1, H-5
8	29.4, CH_2	2.30, m	1.90, m		
9	41.0, CH_2	2.18, m	1.75, m	C-8, C-10	
10	68.7, CH	4.09, m			
11	74.4, C				
12	27.9, CH_3	1.48, s		C-7, C-11, C-13	
13	31.6, CH_3	1.57, s		C-7, C-11, C-12	
14	25.4, CH_3	1.42, d (6.2)		C-9, C-10	
15	27.4, CH_3	1.67, s		C-3, C-4, C-5	H-6
4-OH		5.71, s			
10-OH		5.80, s			
11-OH		5.32, s			

Table S2 Antifungal activities of test compounds (MIC μg/mL, MIC_{80} μg/mL)

Comp	*C. albicans*		*C. parapsilosis*		*C. neoformans*		*C. gattii*	
	MIC	MIC_{80}	MIC	MIC_{80}	MIC	MIC_{80}	MIC	MIC_{80}
**5**	＞400	100	＞400	100	＞400	＞400	＞400	＞400
**6**	＞400	200	＞400	200	＞400	＞400	＞400	＞400
**7**	＞400	200	＞400	100	＞400	＞400	＞400	＞400
**9**	＞400	100	＞400	100	＞400	＞400	＞400	＞400
**12**	＞400	200	＞400	200	＞400	＞400	＞400	＞400
**31**	＞400	200	＞400	100	＞400	＞400	＞400	＞400
**32**	＞400	400	＞400	400	＞400	＞400	＞400	＞400
**33**	＞400	400	＞400	400	＞400	＞400	＞400	＞400
**34**	＞400	＞400	＞400	＞400	50	25	50	25
AmB	2	0.5	2	0.5	2	0.25	2	0.25

AmB, amphotericin B was served as the positive control.

Table S3 Antibacterial activities of compounds **5−7** (MIC μg/mL)

Comp	MIC				
	B. subtilis	*S. aureus*	*E. faecium*	*E. coli*	*P. aeruginosa*
**5**	100	>200	200	100	>200
**6**	200	>200	200	200	>200
**7**	>200	>200	>200	200	>200
Cip	0.13	0.13	0.25	0.13	0.13

Cip, Ciprofloxacin was served as the positive control.

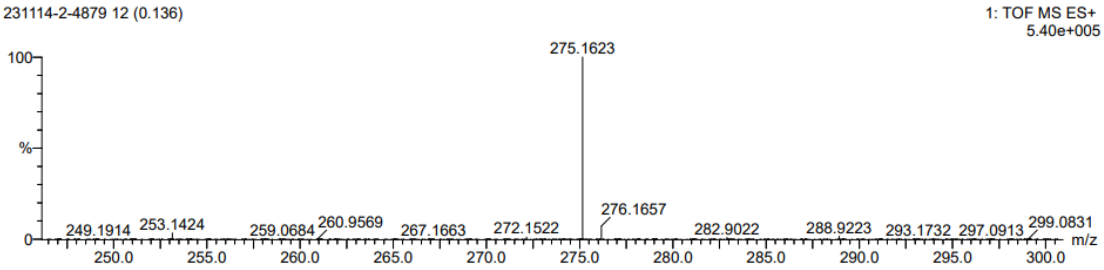

Figure S1. HRESI-MS spectrum of the new compound **1**

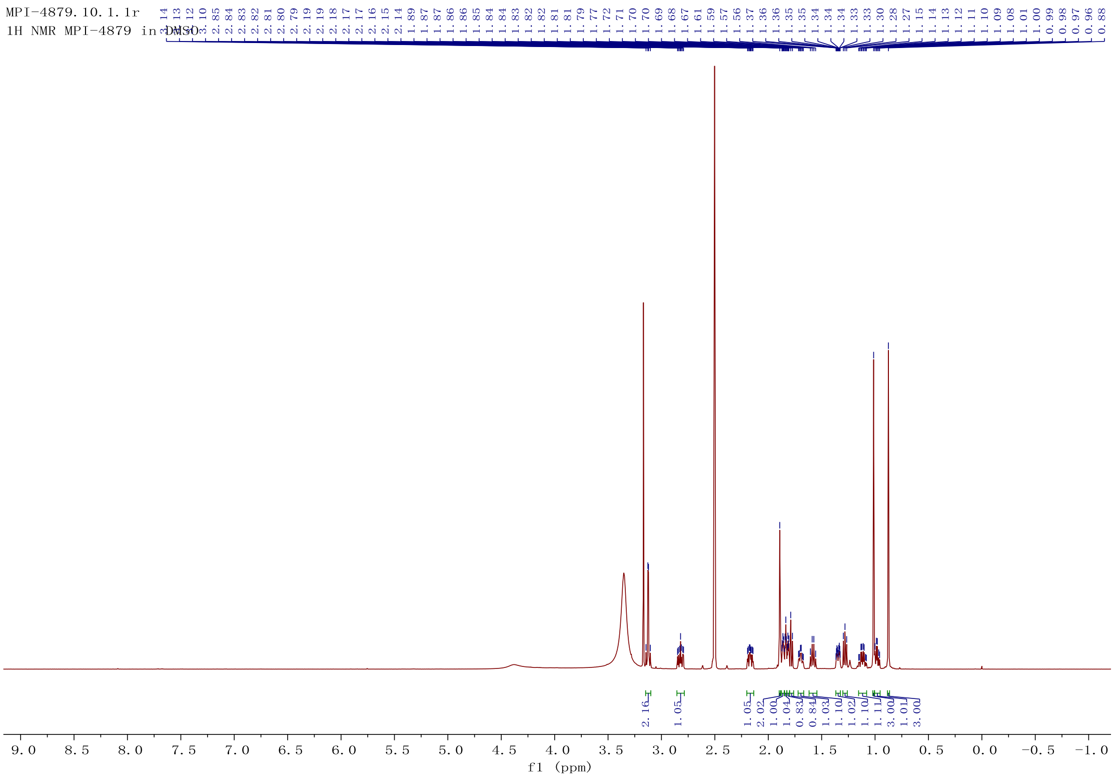

Figure S2. ^{1}H NMR (600 MHz, DMSO-d_6) spectrum of the new compound **1**

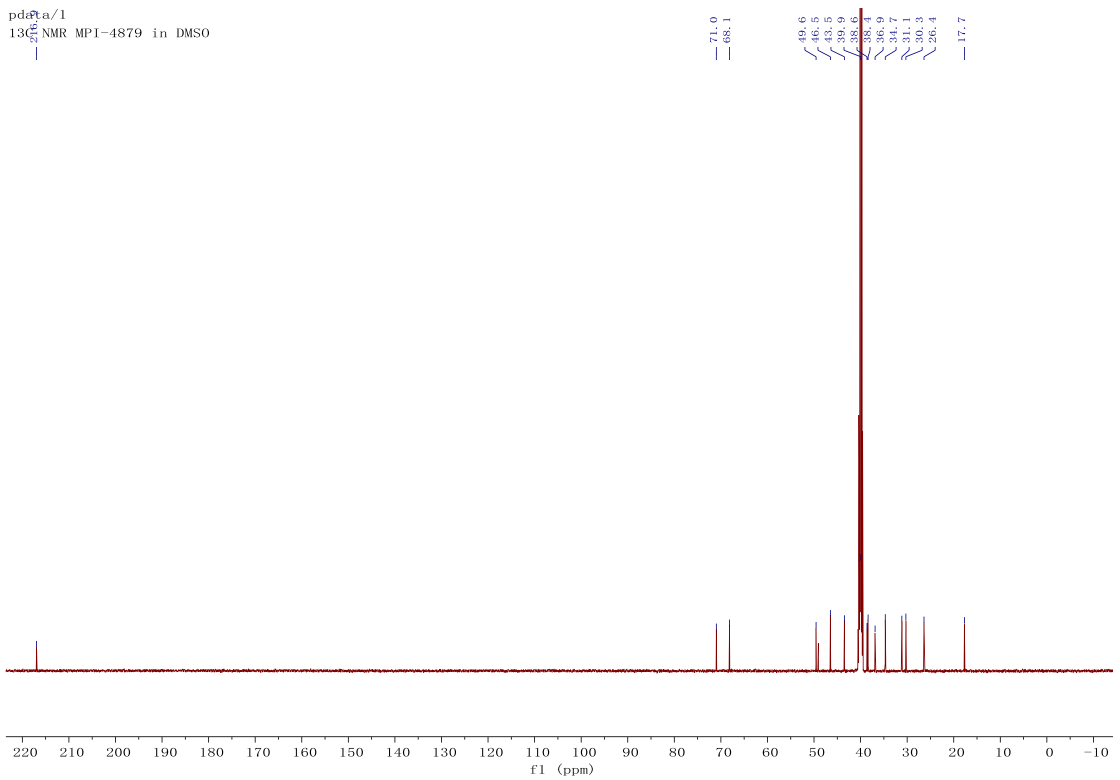

Figure S3. ^{13}C NMR (150 MHz, DMSO-d_6) spectrum of the new compound **1**

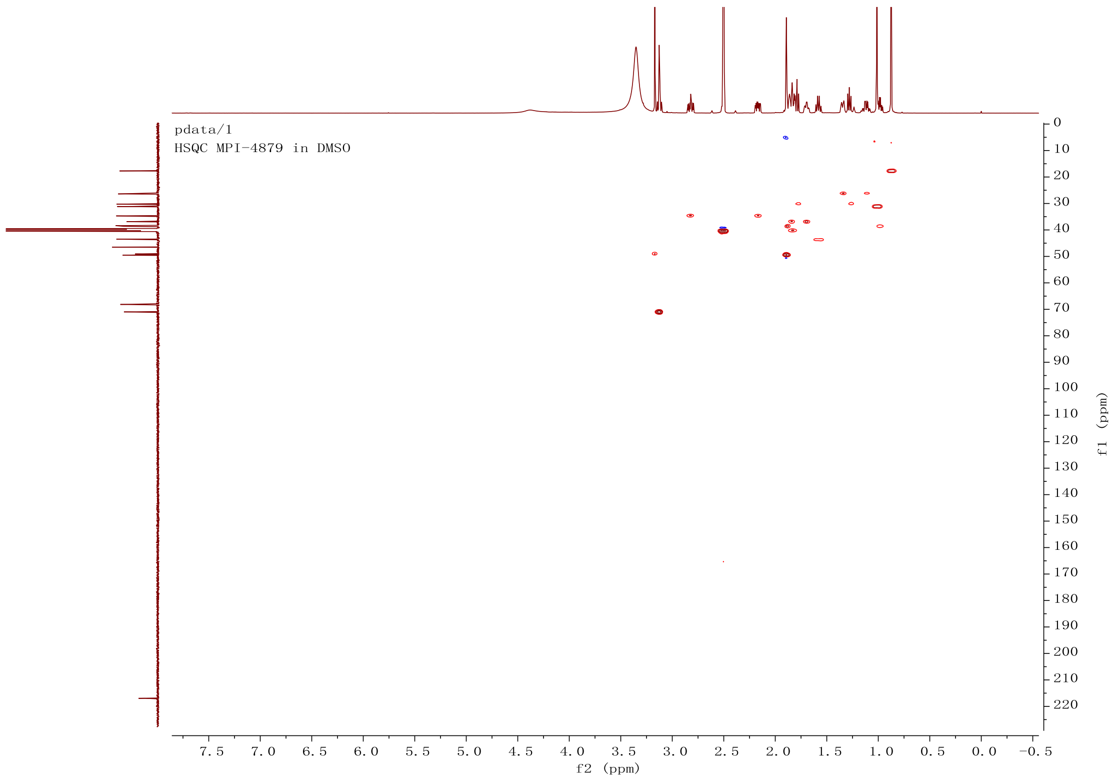

Figure S4. HSQC spectrum of the new compound **1**

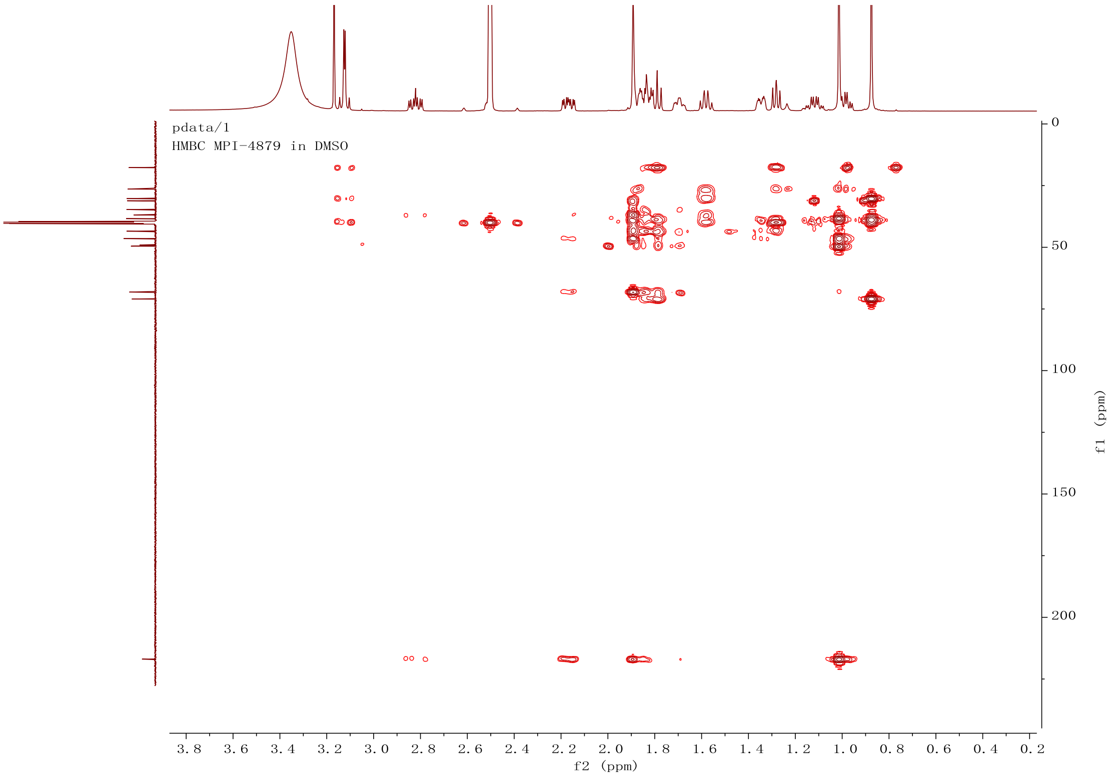

Figure S5. HMBC spectrum of the new compound **1**

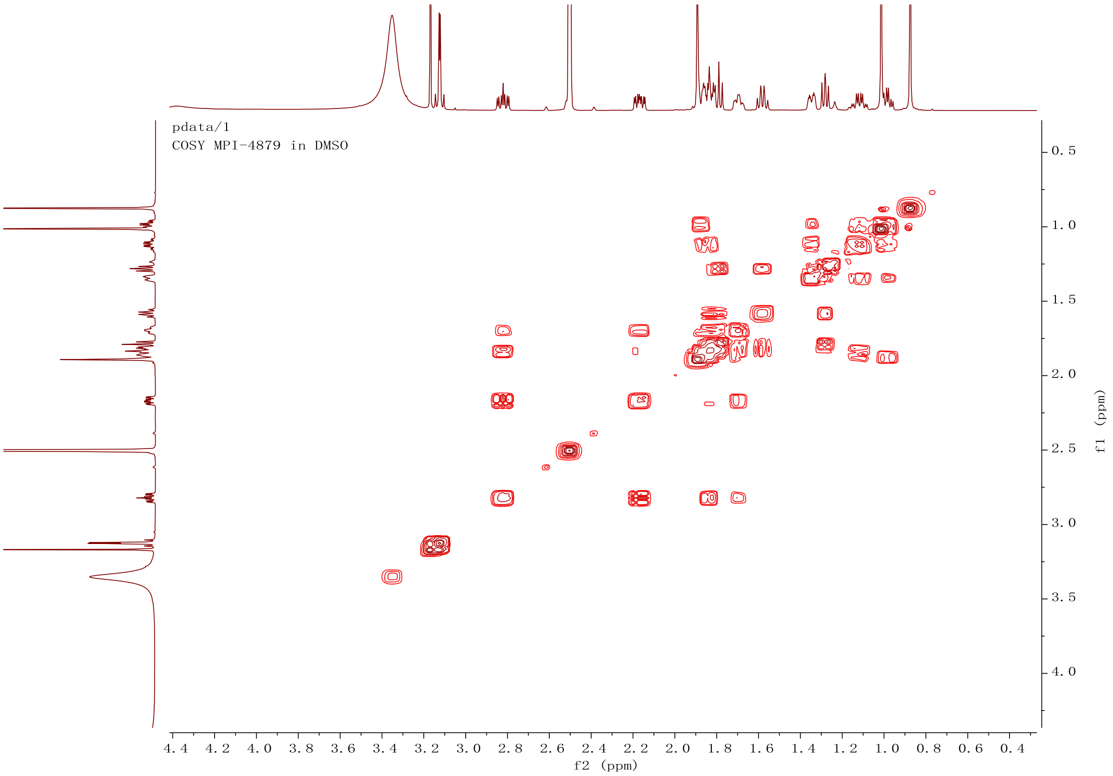

Figure S6. COSY spectrum of the new compound **1**

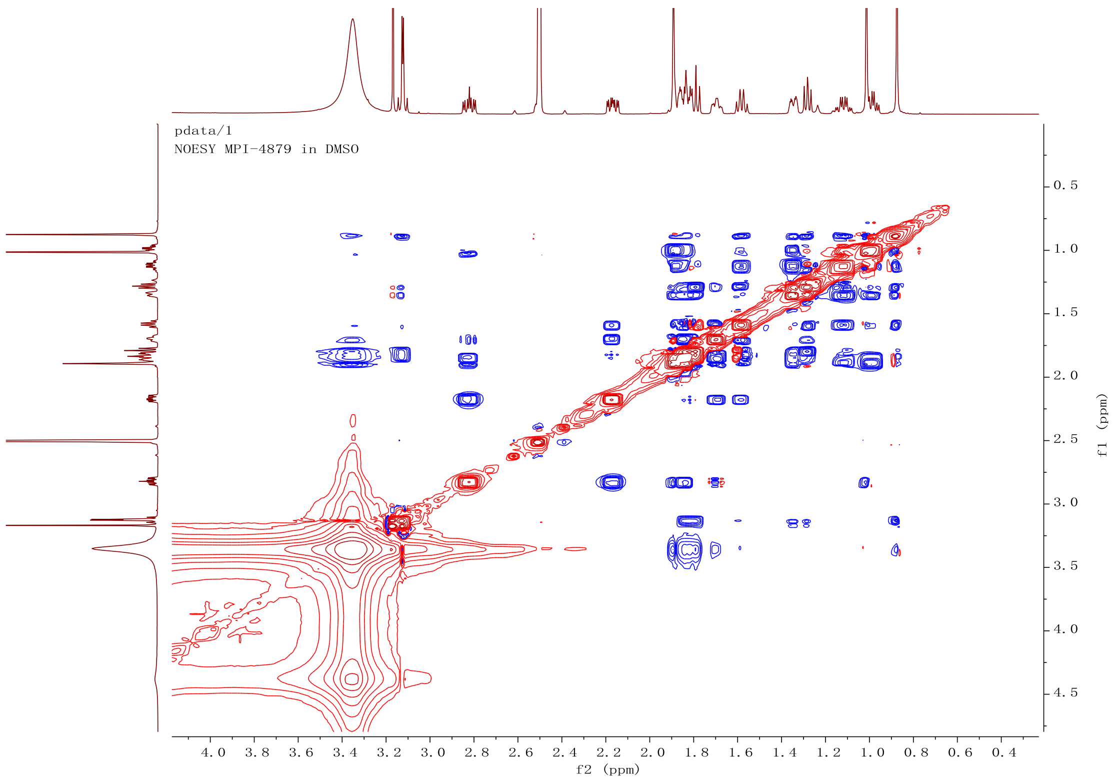

Figure S7. NOESY spectrum of the new compound **1**

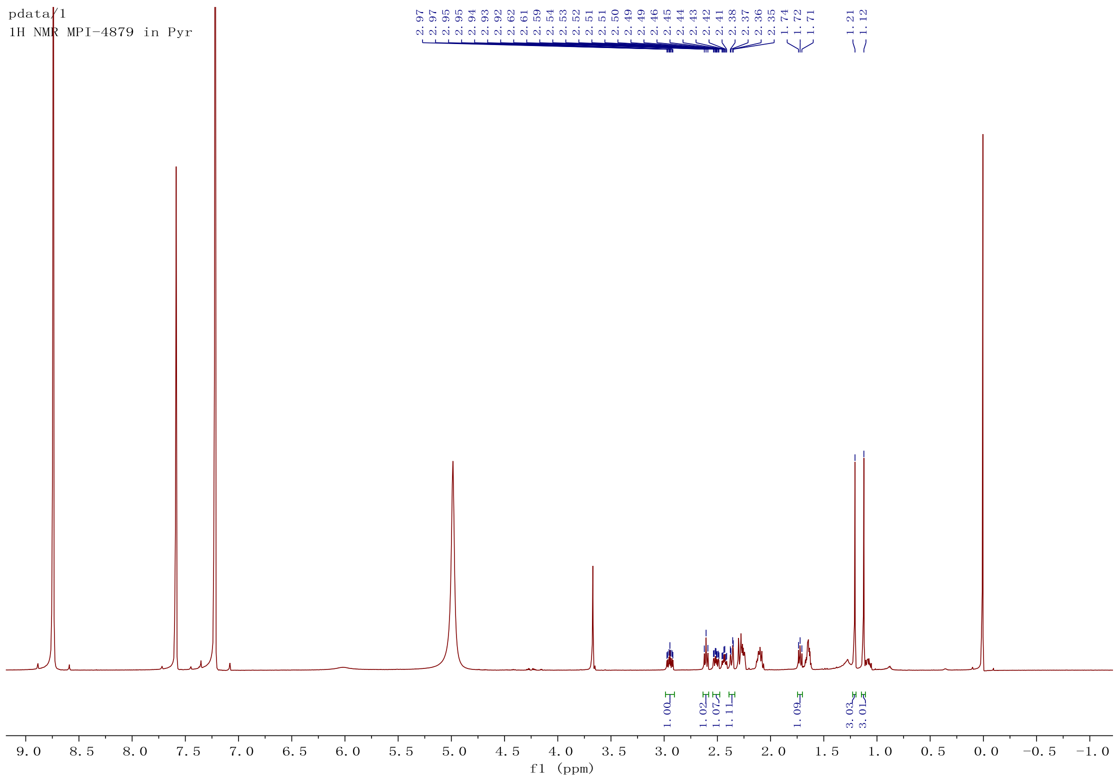

Figure S8. ^{1}H NMR (600 MHz, in pyridine-d_5) spectrum of the new compound **1**

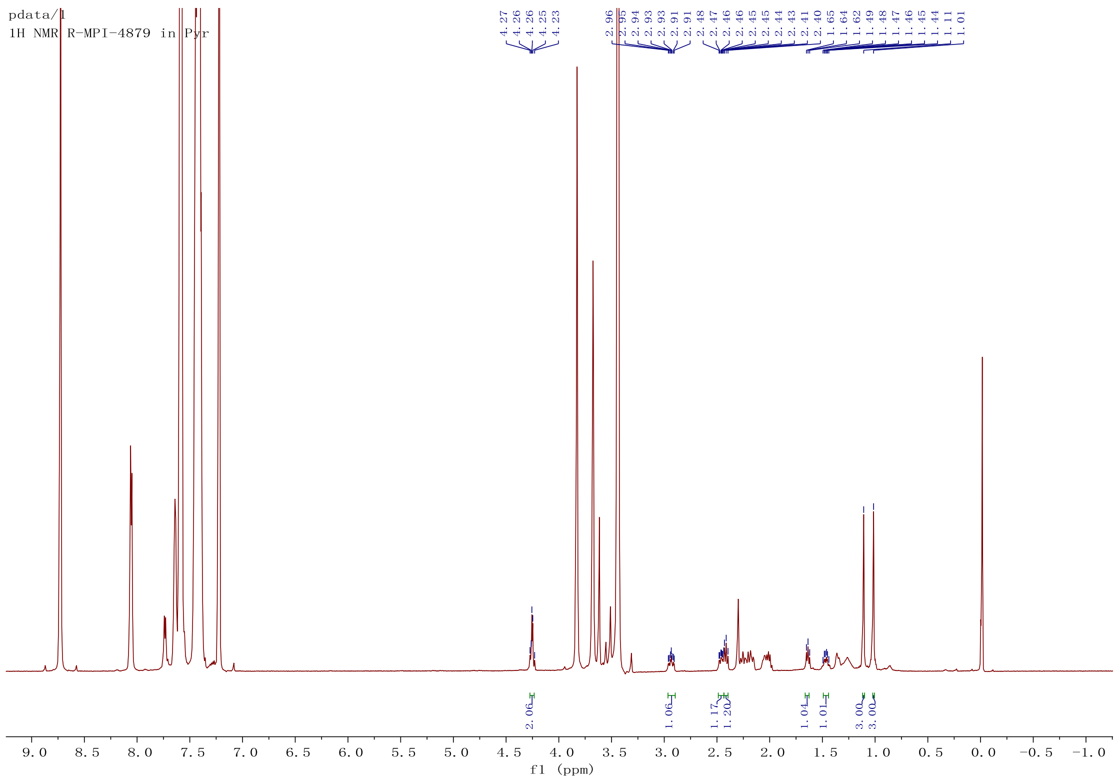

Figure S9. ^{1}H NMR spectrum of (*S*)-MTPA ester (**1a**) in pyridine-d_5

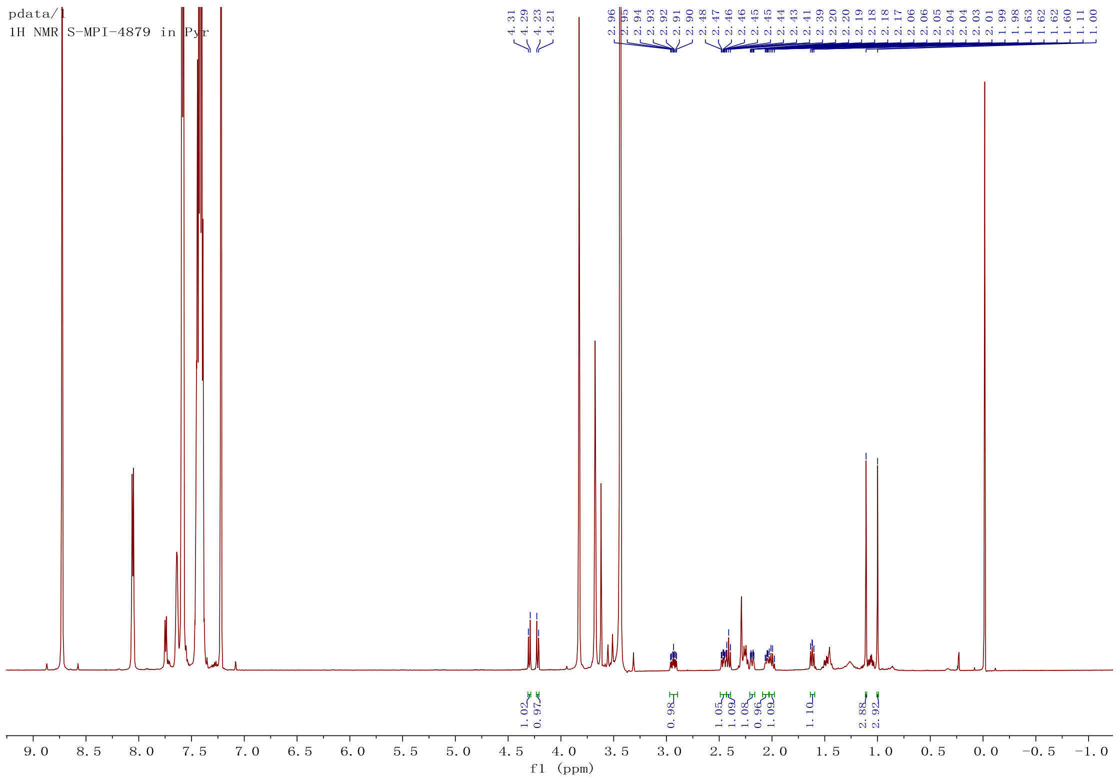

Figure S10. ^{1}H NMR spectrum of (*R*)-MTPA ester (**1b**) in pyridine-d_5

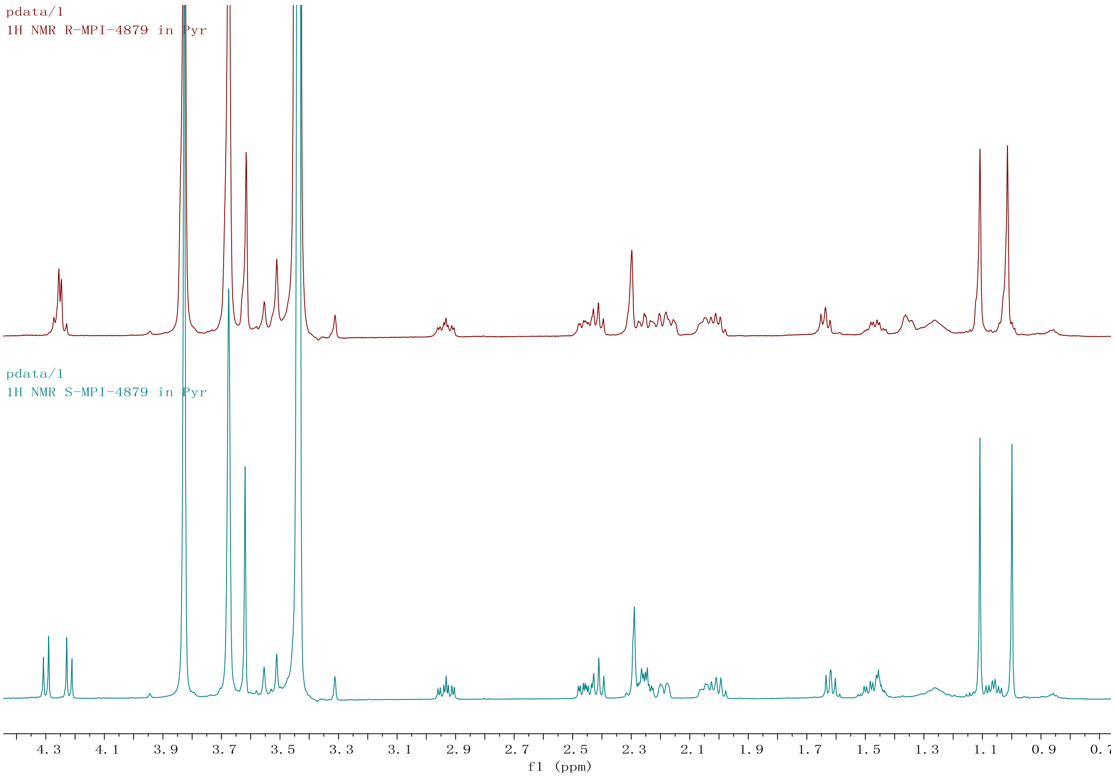

Figure S11. Comparison of ^{1}H NMR spectra of (*S*)-MTPA ester (**1a**) and (*R*)-MTPA ester (**1b**) in pyridine-d_5

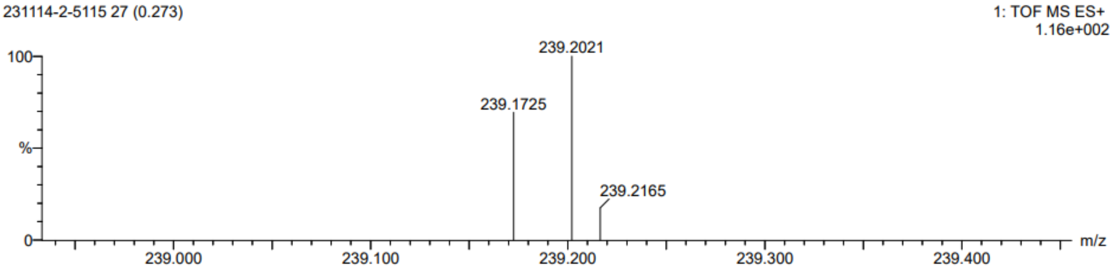

Figure S12. HRESI-MS spectrum of the new compound **2**

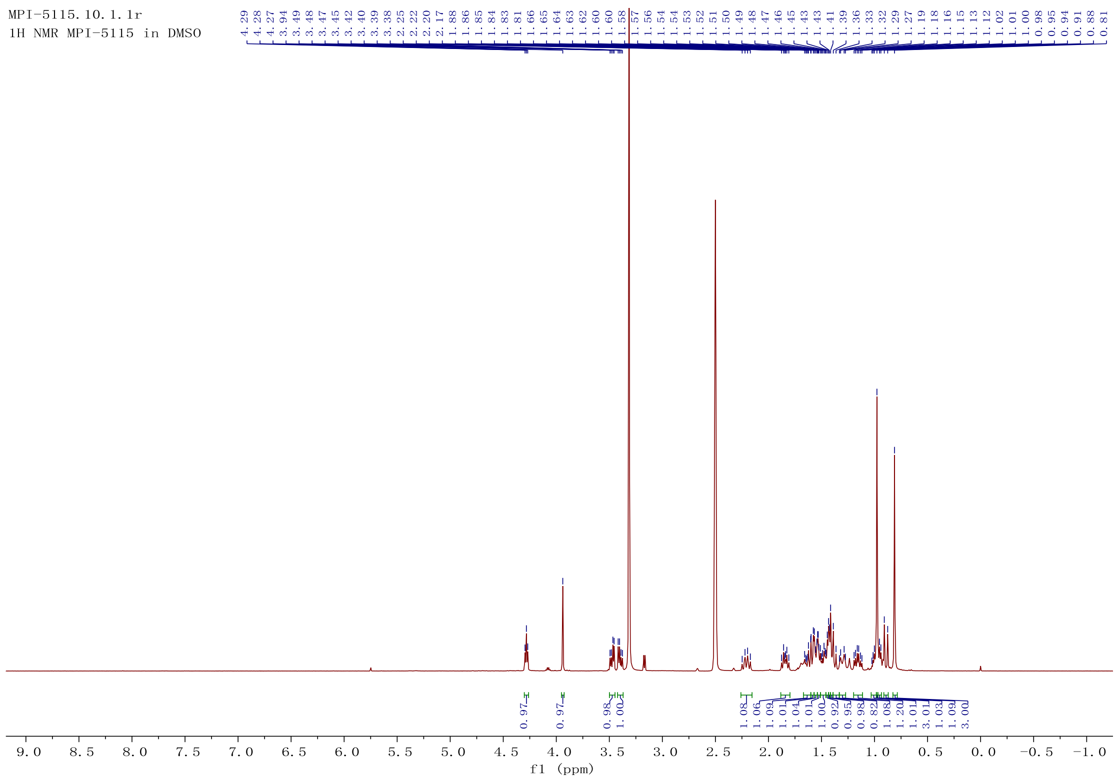

Figure S13. ^{1}H NMR (600 MHz, DMSO-d_6) spectrum of the new compound **2**

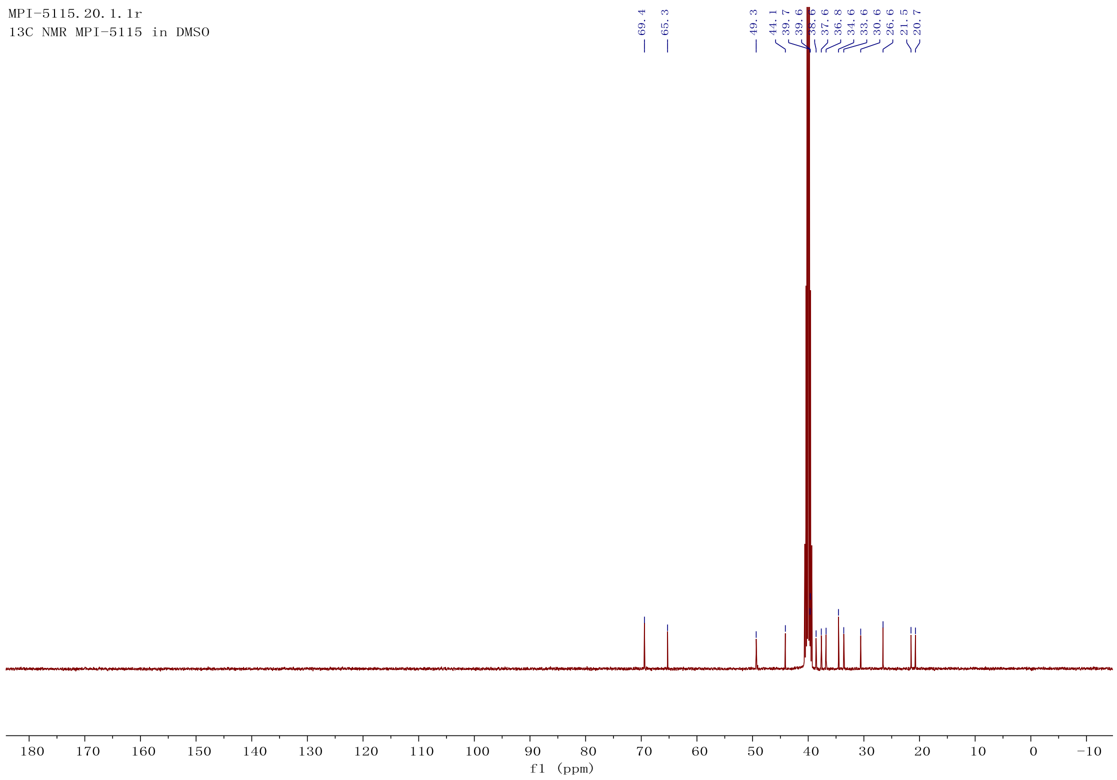

Figure S14. ^{13}C NMR (150 MHz, DMSO-d_6) spectrum of the new compound **2**

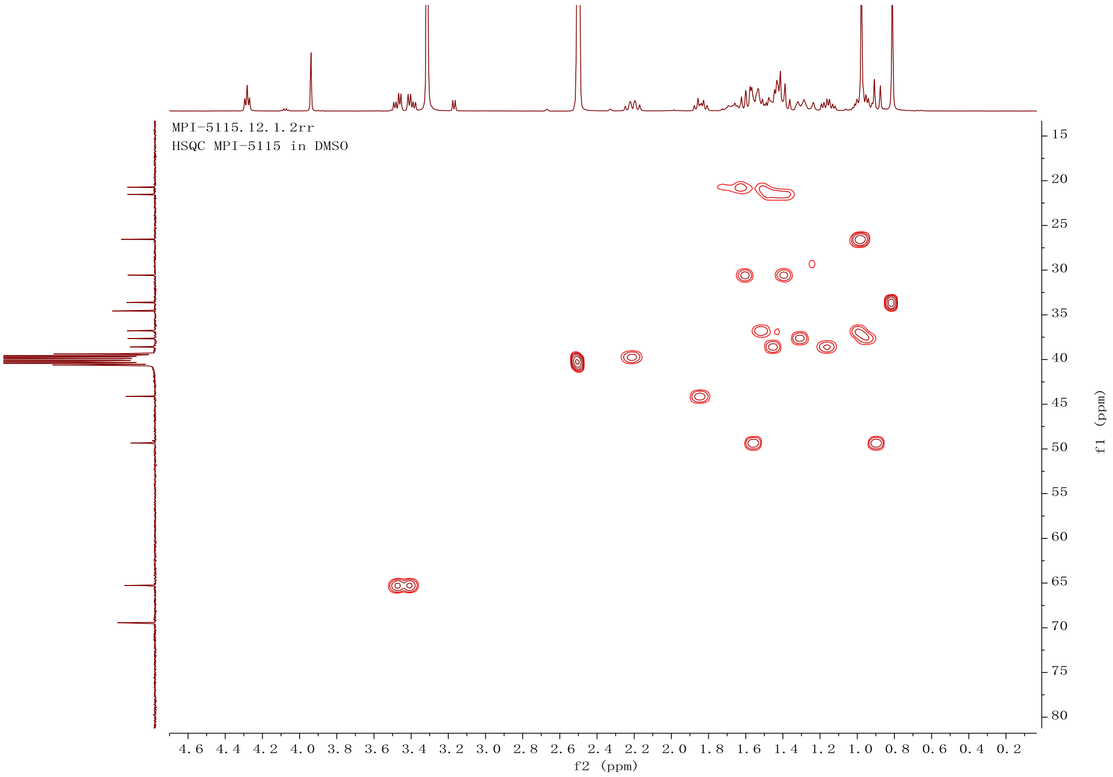

Figure S15. HSQC spectrum of the new compound **2**

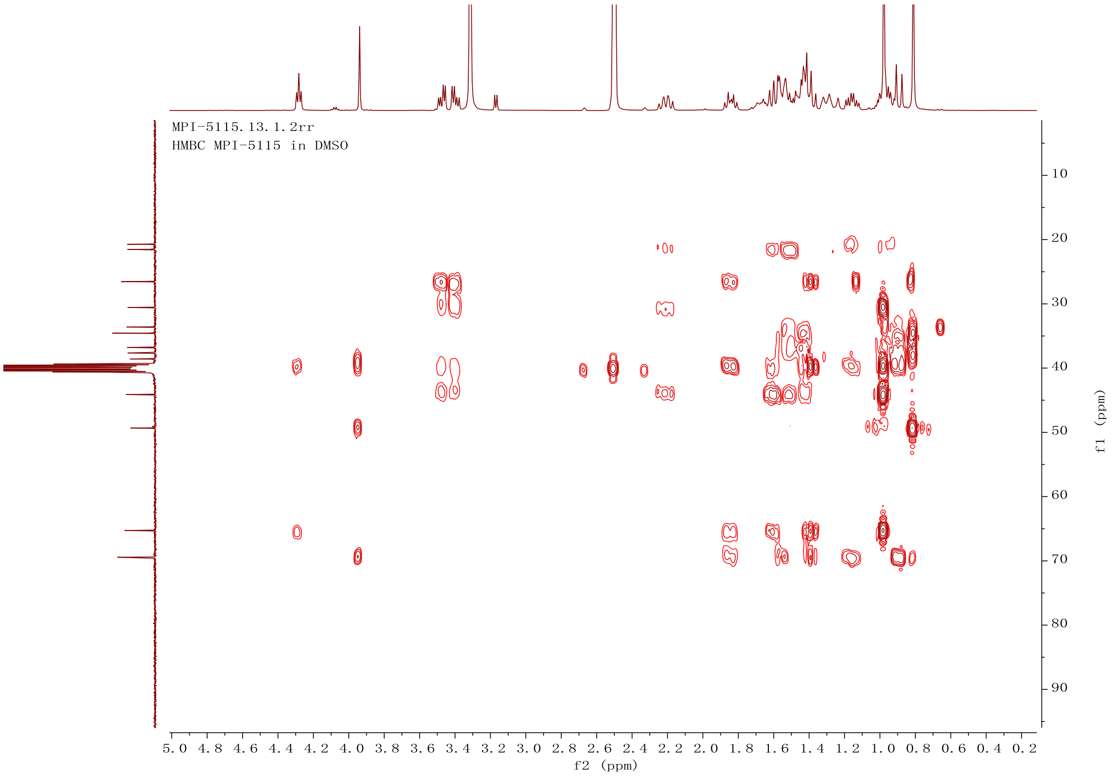

Figure S16. HMBC spectrum of the new compound **2**

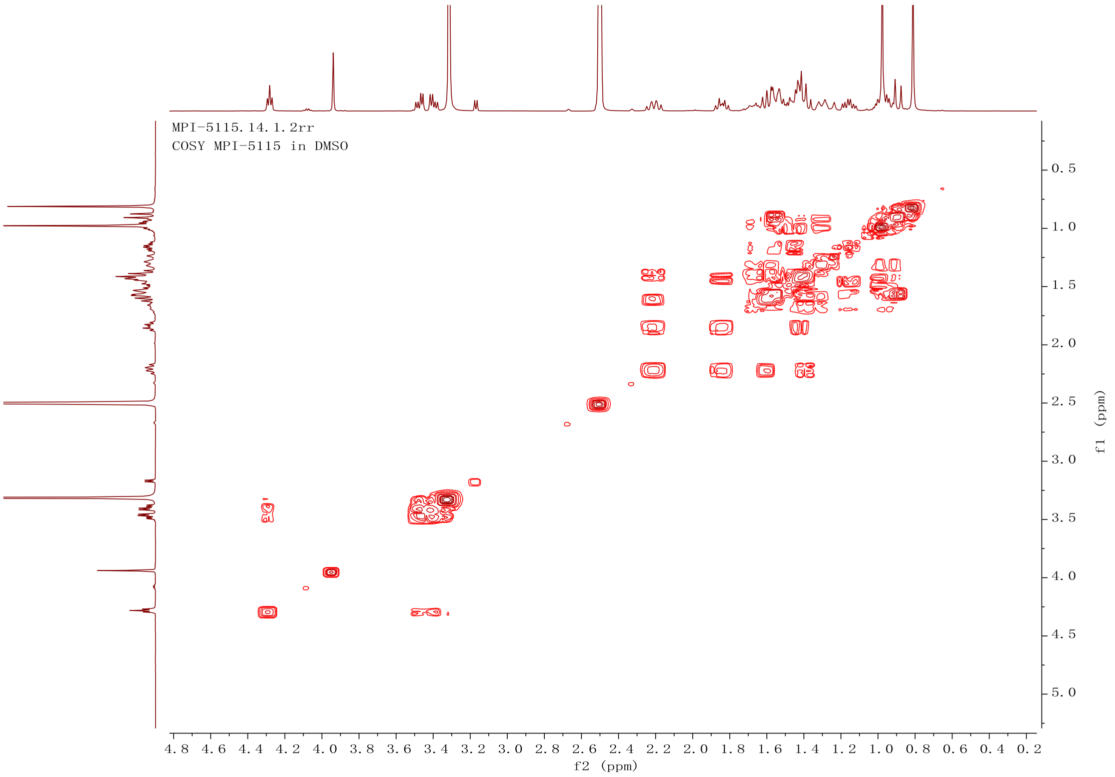

Figure S17. COSY spectrum of the new compound **2**

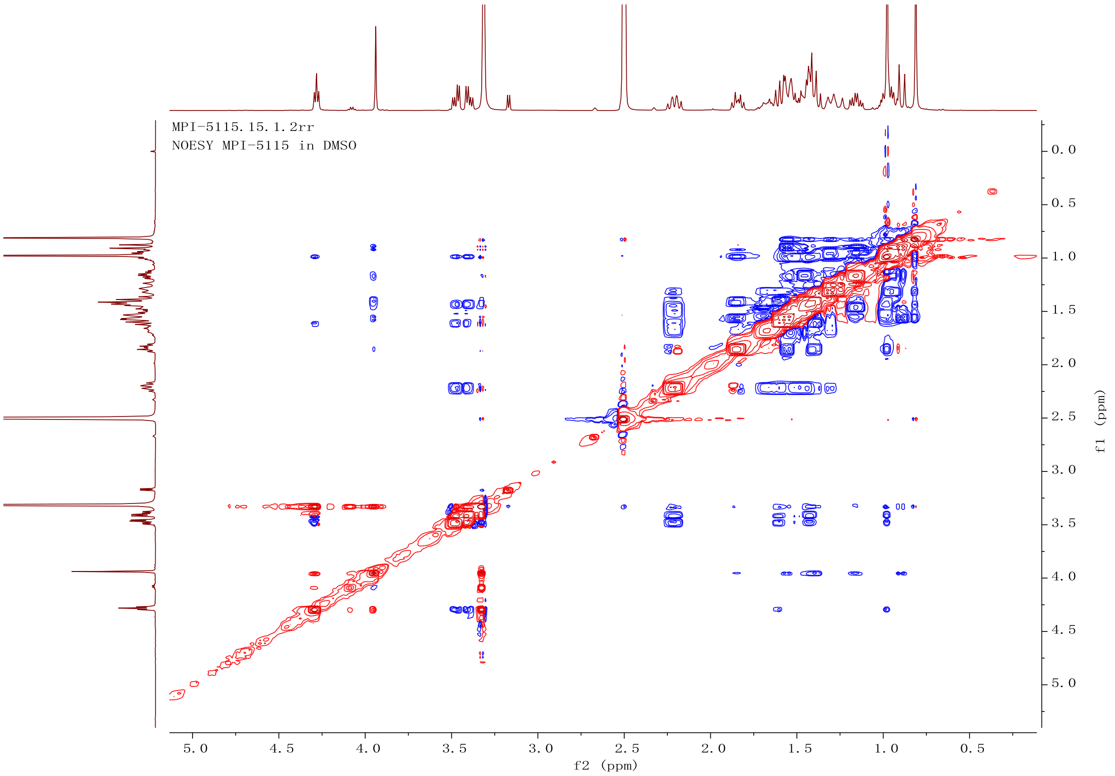

Figure S18. NOESY spectrum of the new compound **2**

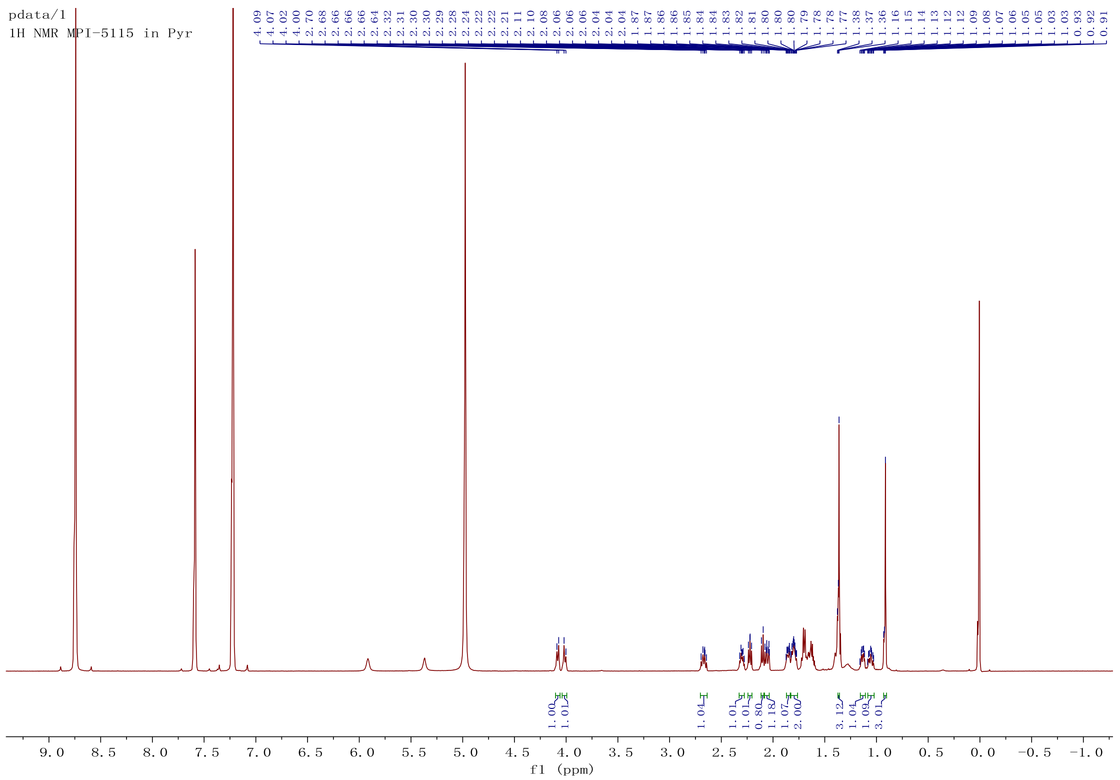

Figure S19. ^{1}H NMR (600 MHz, in pyridine-d_5) spectrum of the new compound **2**

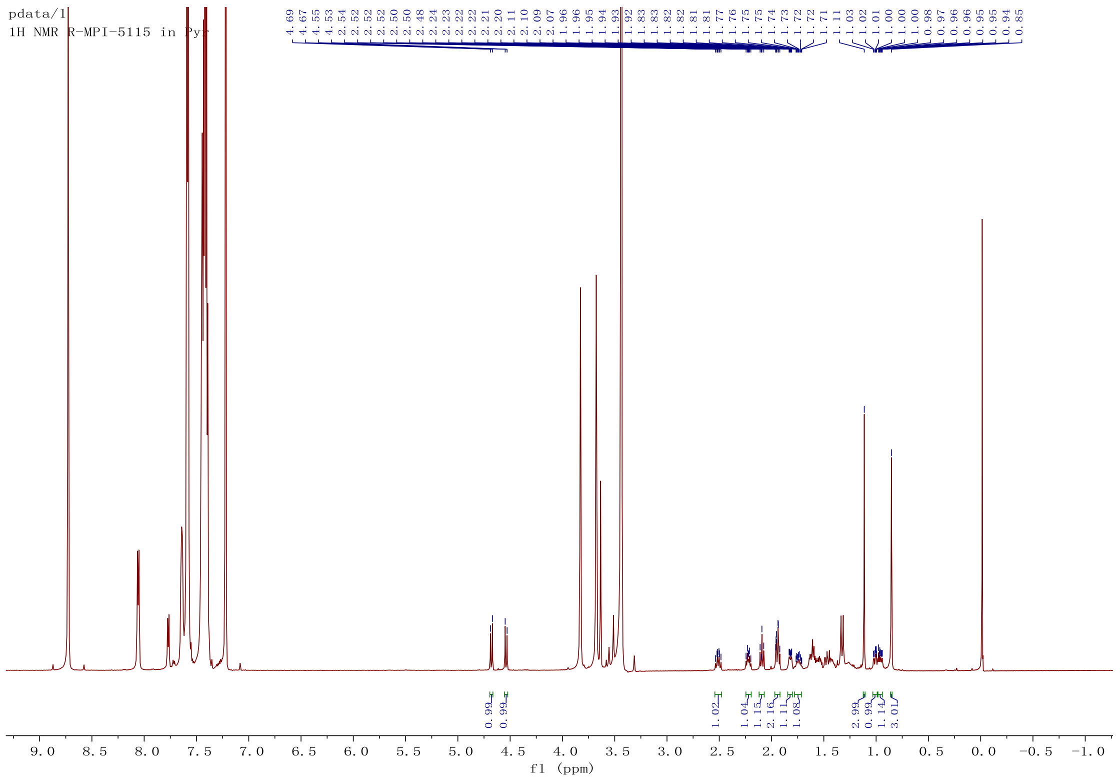

Figure S20. ^{1}H NMR spectrum of (*S*)-MTPA ester (**2a**) in pyridine-d_5

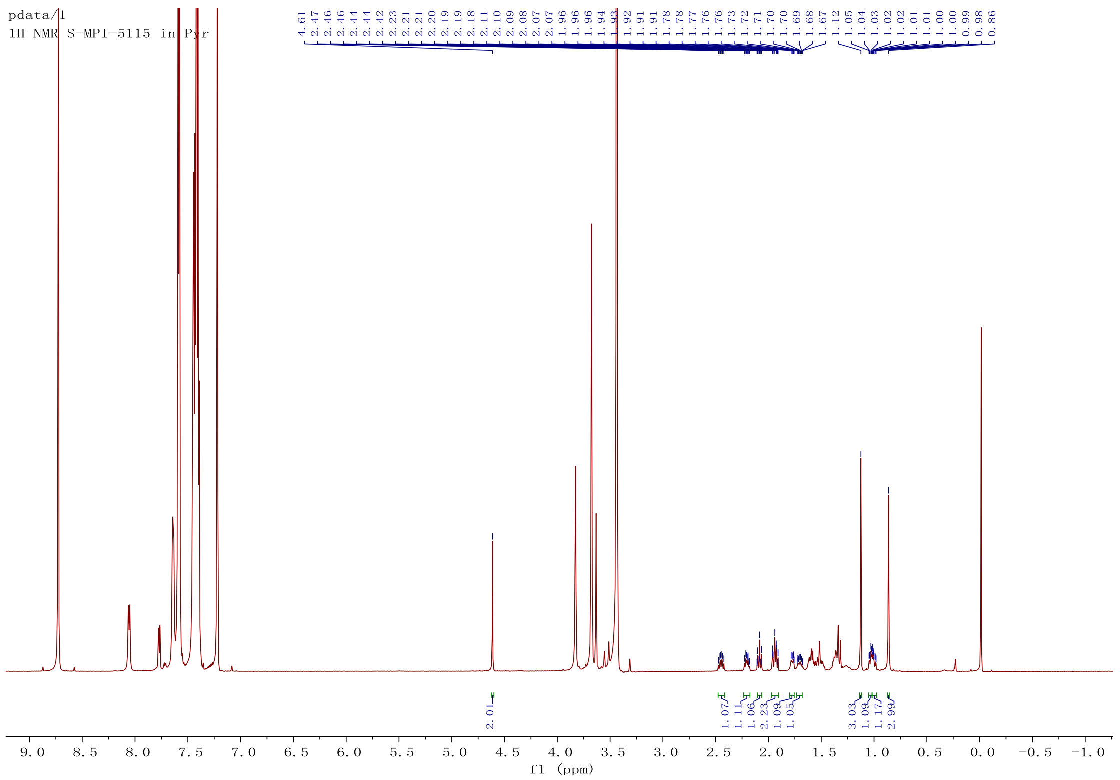

Figure S21. ^{1}H NMR spectrum of (*R*)-MTPA ester (**2b**) in pyridine-d_5

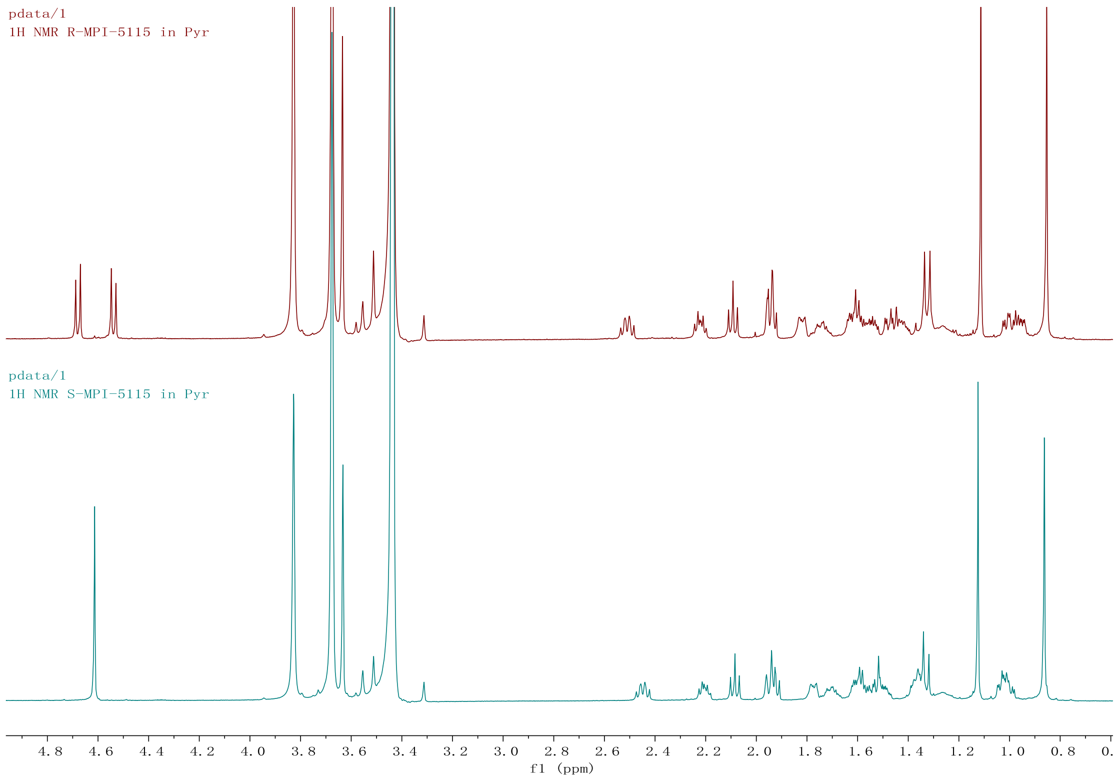

Figure S22. Comparison of ^{1}H NMR spectra of (*S*)-MTPA ester (**2a**) and (*R*)-MTPA ester (**2b**) in pyridine-d_5

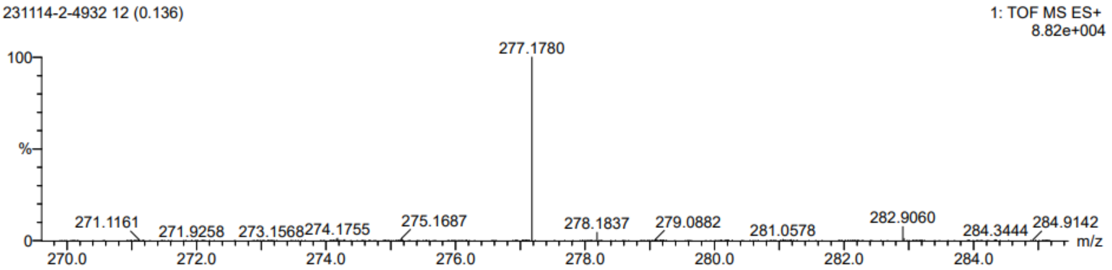

Figure S23. HRESI-MS spectrum of the new compound **3**

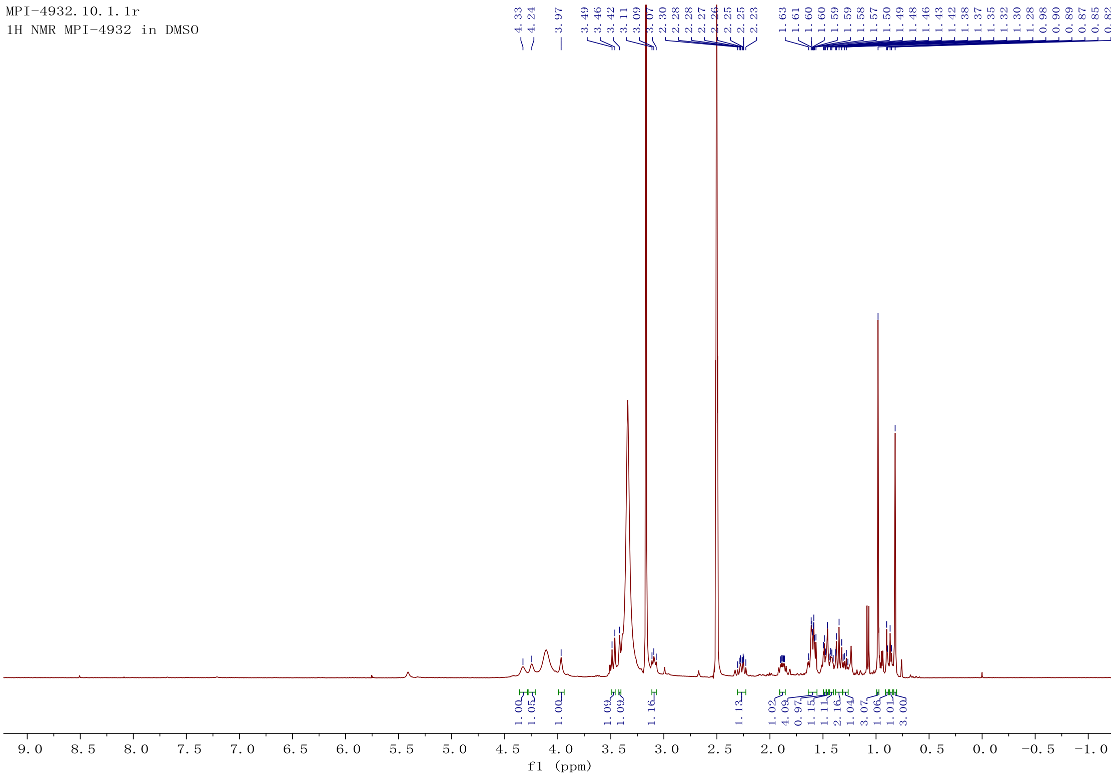

Figure S24. ^{1}H NMR (600 MHz, DMSO-d_6) spectrum of the new compound **3**

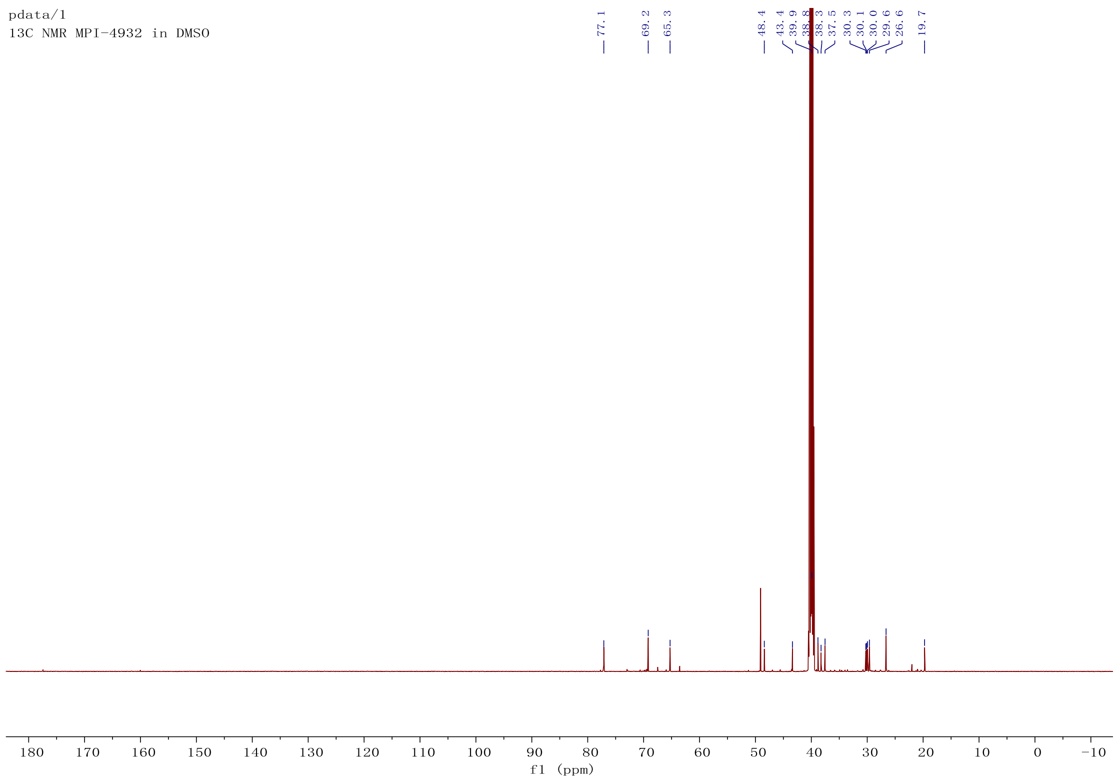

Figure S25. ^{13}C NMR (150 MHz, DMSO-d_6) spectrum of the new compound **3**

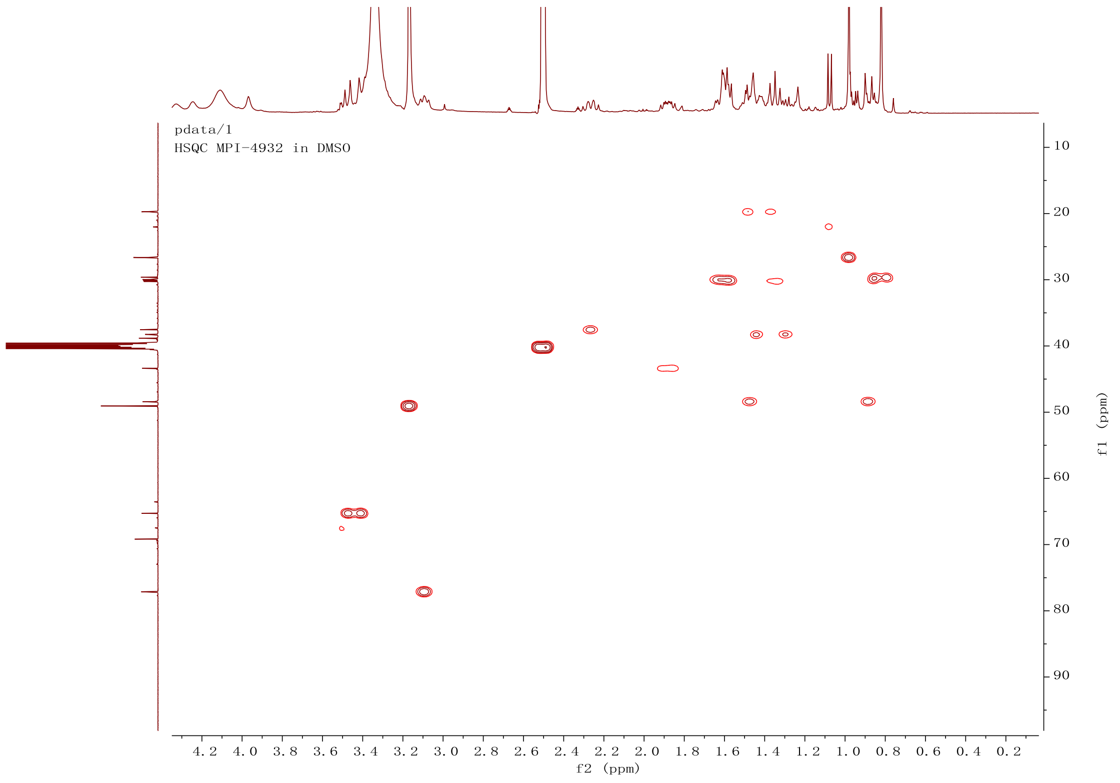

Figure S26. HSQC spectrum of the new compound **3**

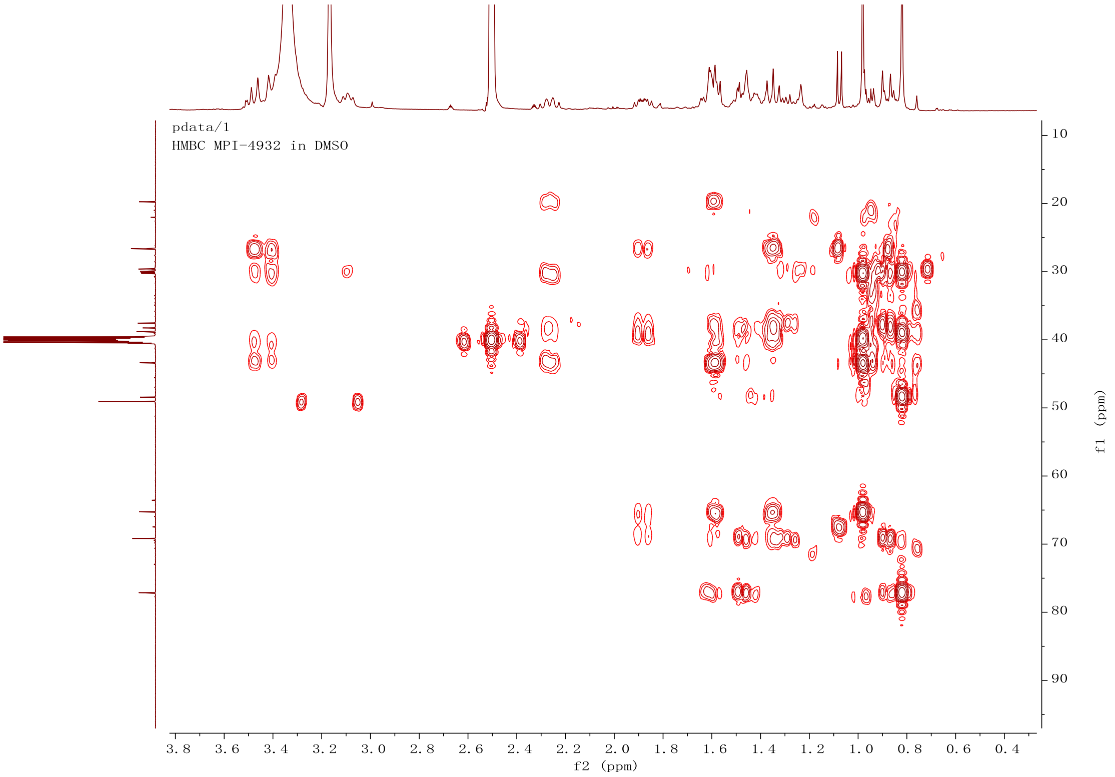

Figure S27. HMBC spectrum of the new compound **3**

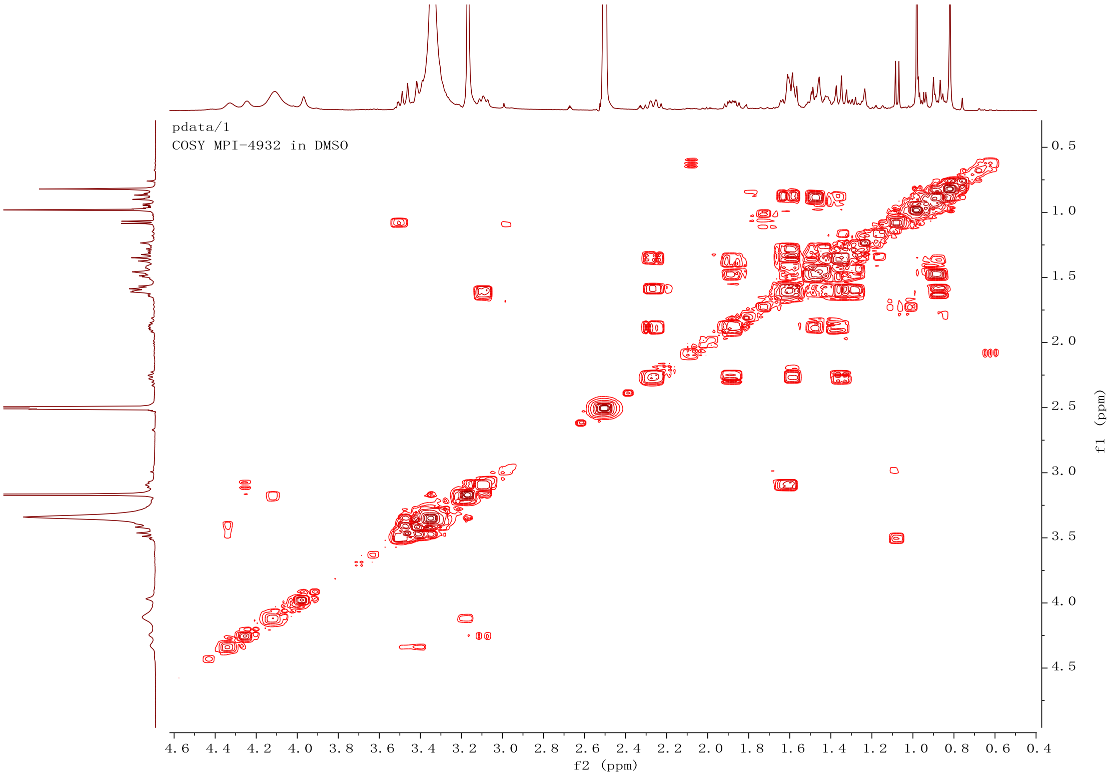

Figure S28. COSY spectrum of the new compound **3**

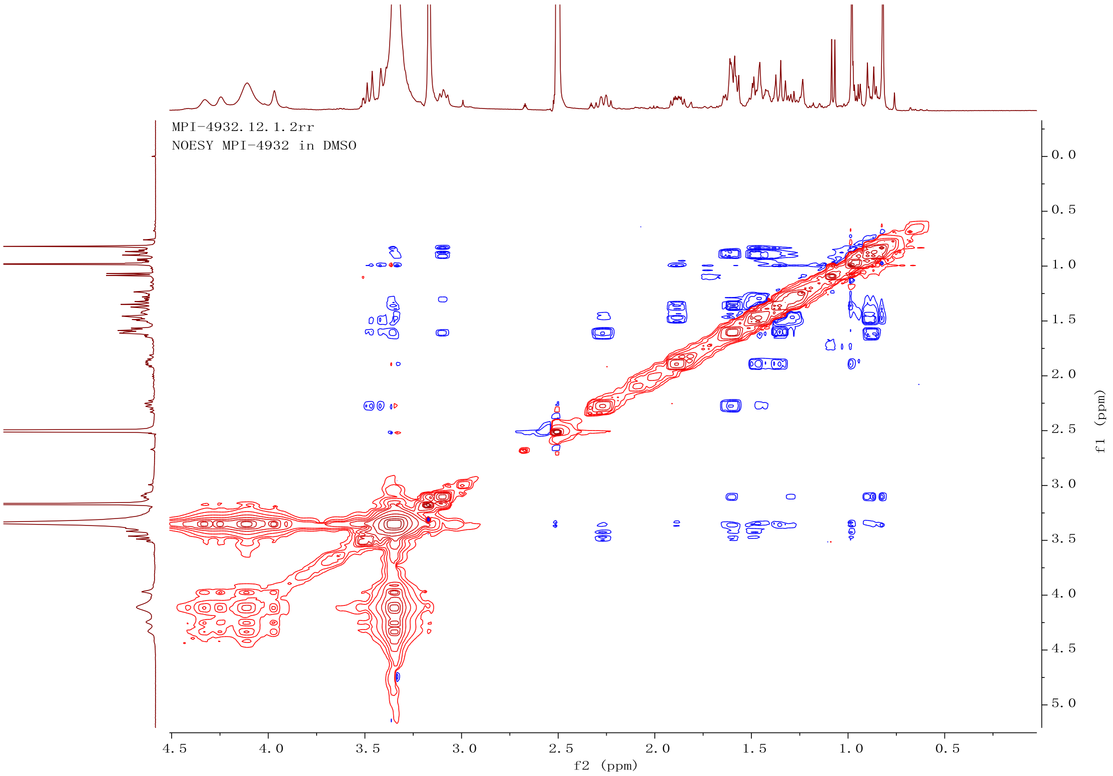

Figure S29. NOESY spectrum of the new compound **3**

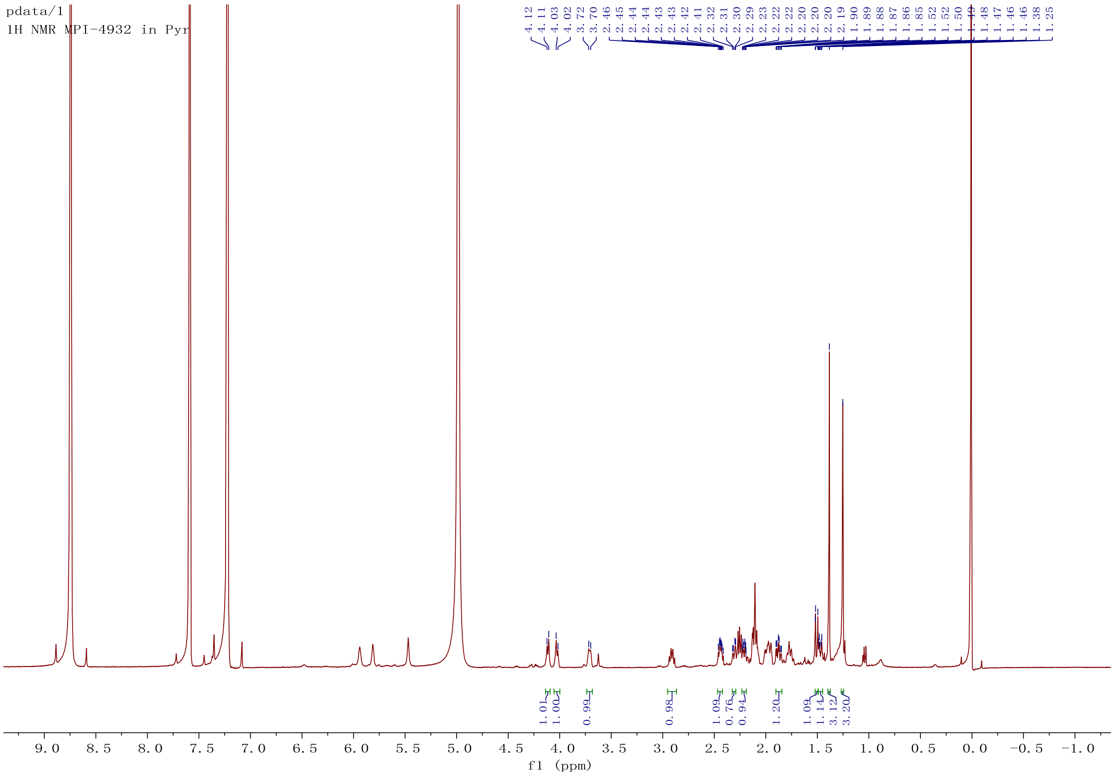

Figure S30. ^{1}H NMR (600 MHz, in pyridine-d_5) spectrum of the new compound **3**

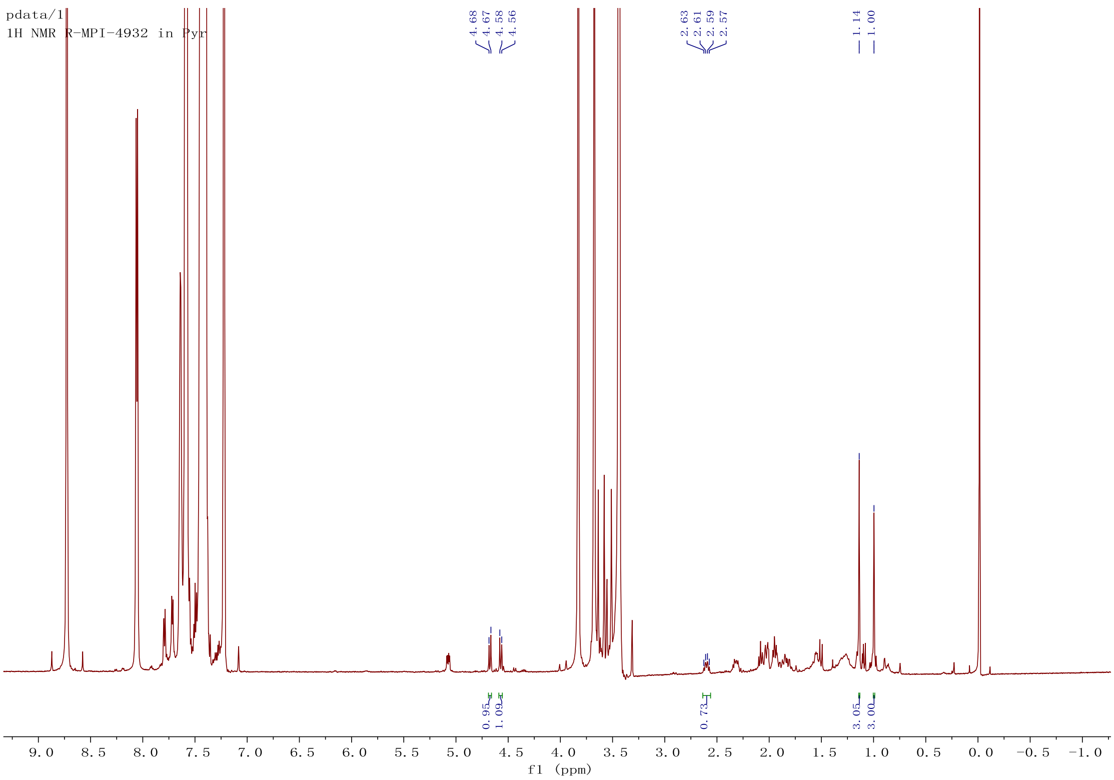

Figure S31. ^{1}H NMR spectrum of (*S*)-MTPA ester (**3a**) in pyridine-d_5

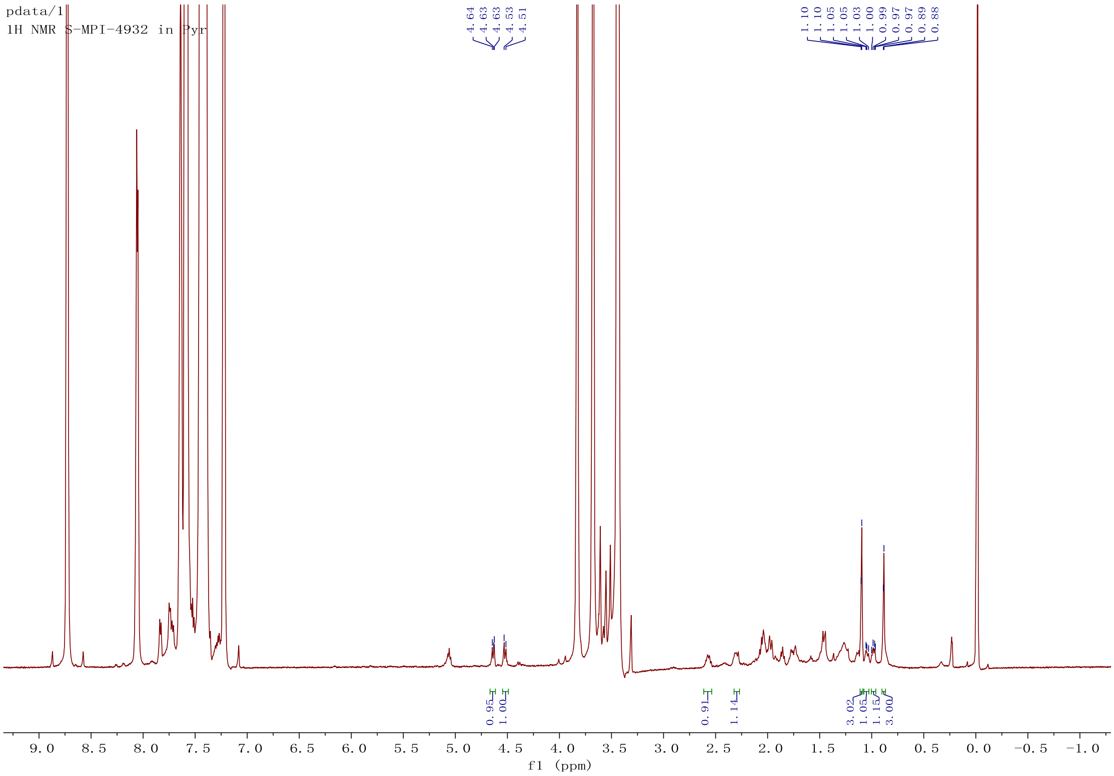

Figure S32. ^{1}H NMR spectrum of (*R*)-MTPA ester (**3b**) in pyridine-d_5

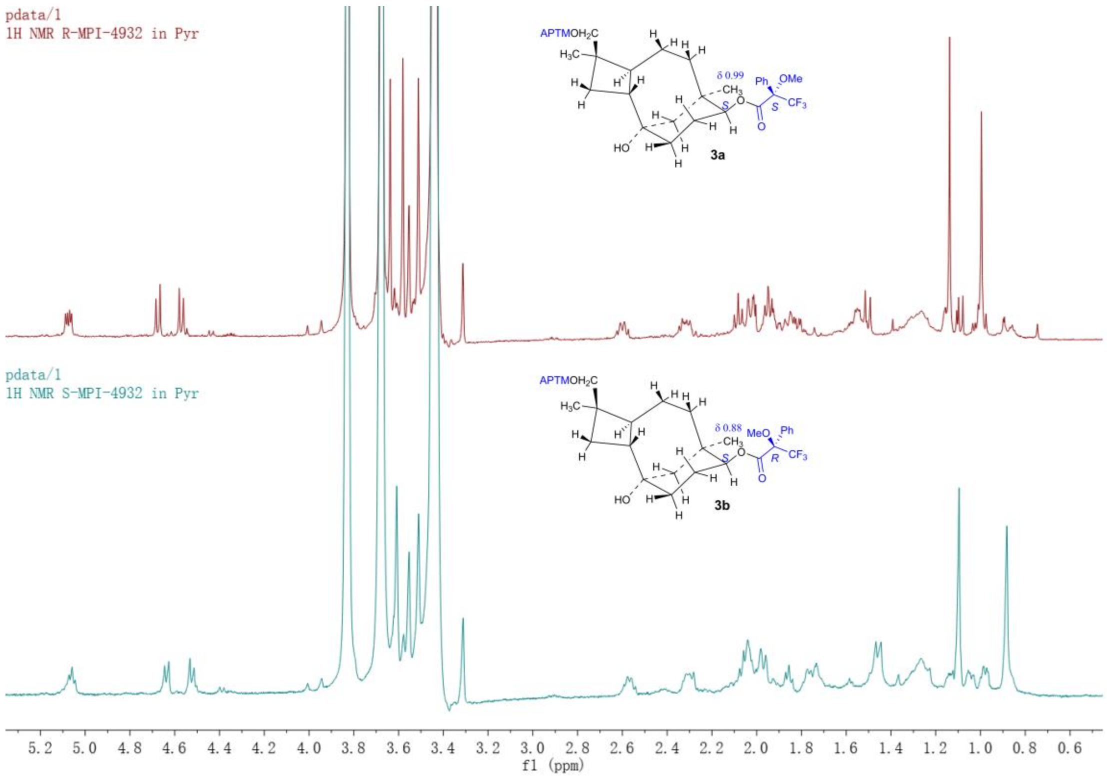

Figure S33. Comparison of ^{1}H NMR spectra of (*S*)-MTPA ester (**3a**) and (*R*)-MTPA ester (**3b**) in pyridine-d_5

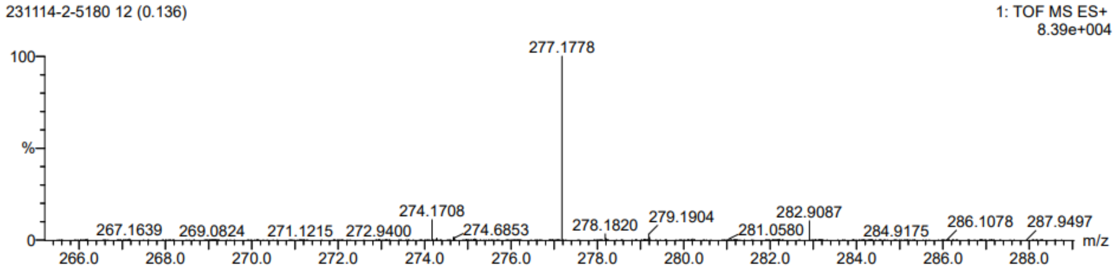

Figure S34. HRESI-MS spectrum of the new compound **4**

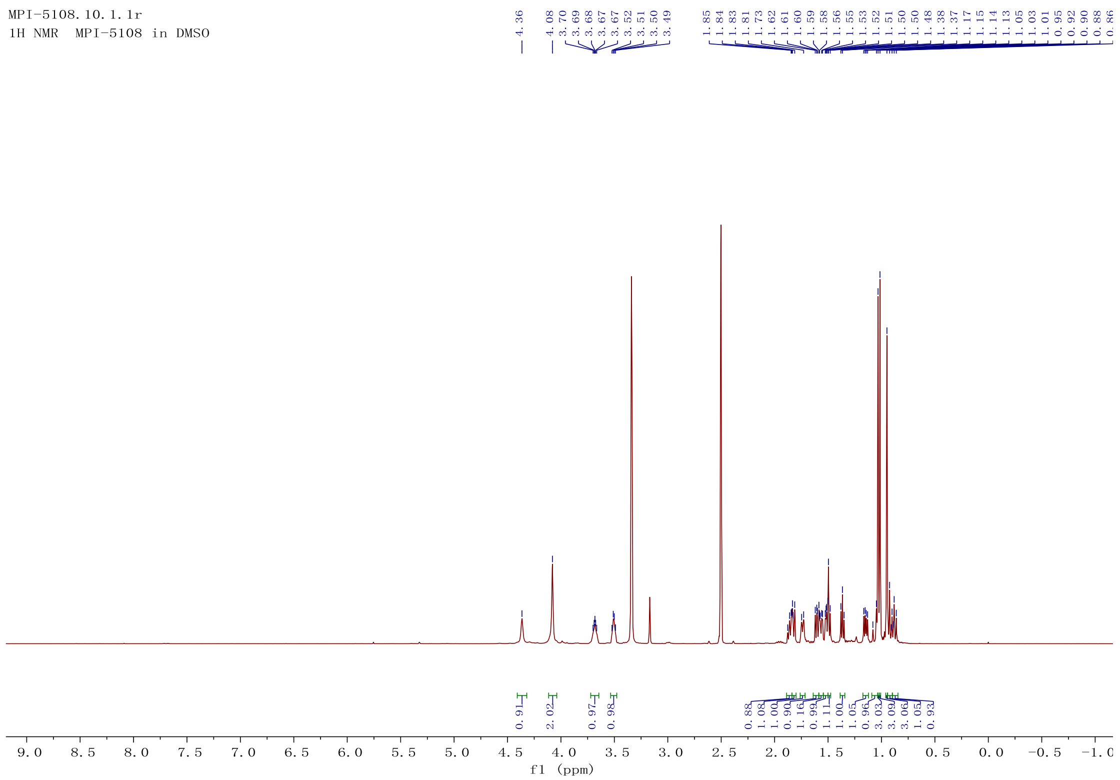

Figure S35. ^{1}H NMR (600 MHz, DMSO-d_6) spectrum of the new compound **4**

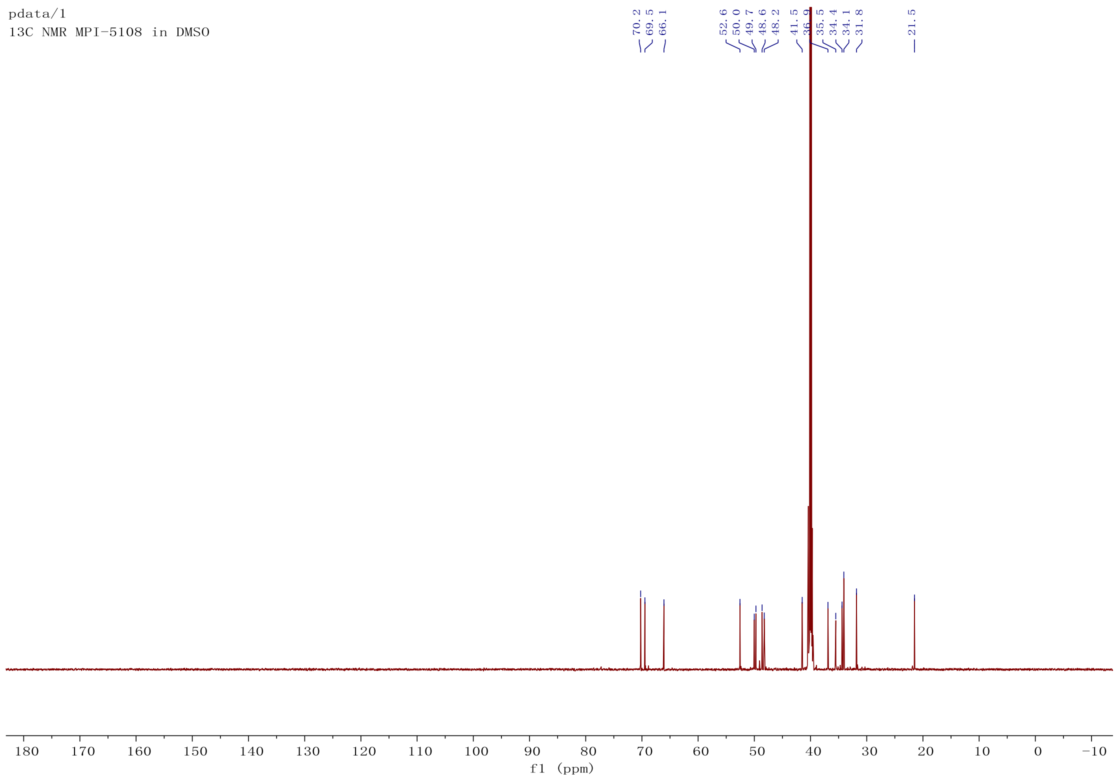

Figure S36. ^{13}C NMR (150 MHz, DMSO-d_6) spectrum of the new compound **4**

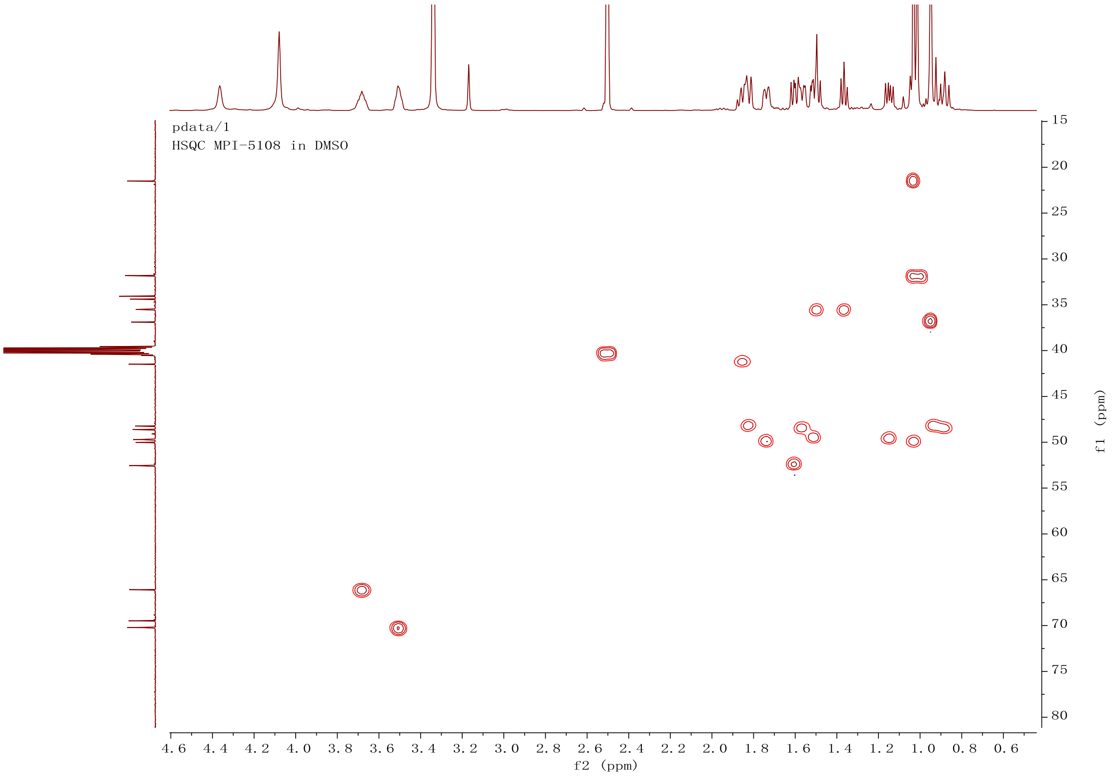

Figure S37. HSQC spectrum of the new compound **4**

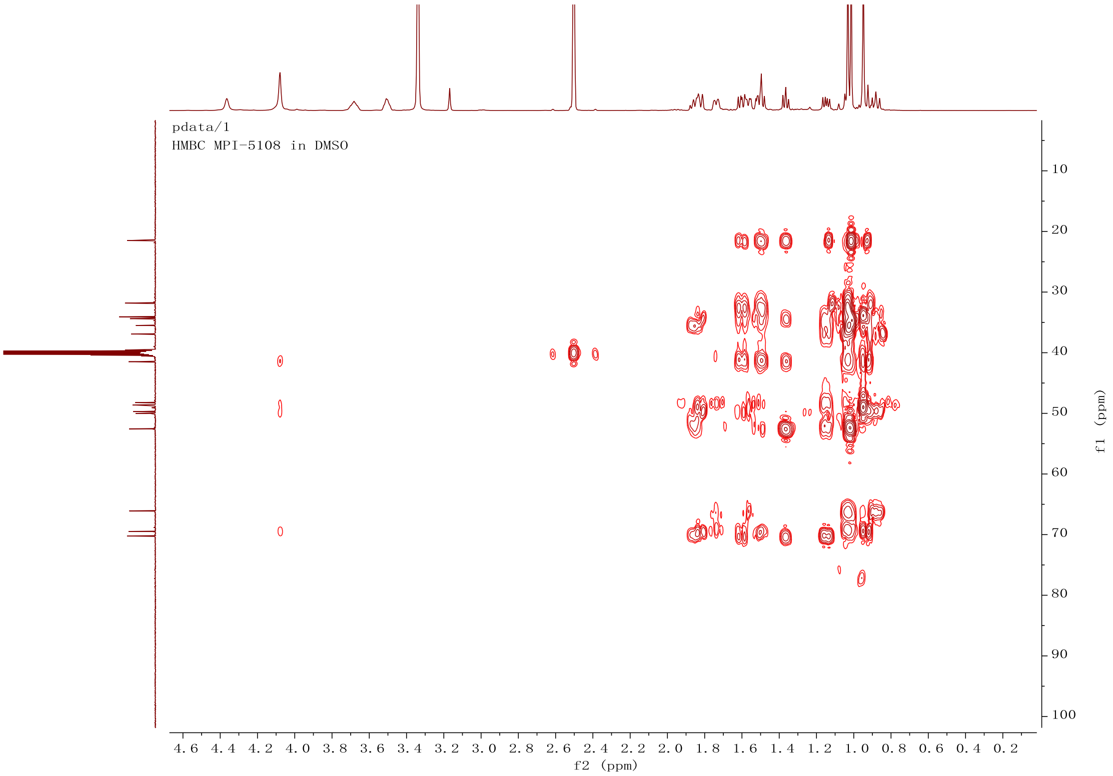

Figure S38. HMBC spectrum of the new compound **4**

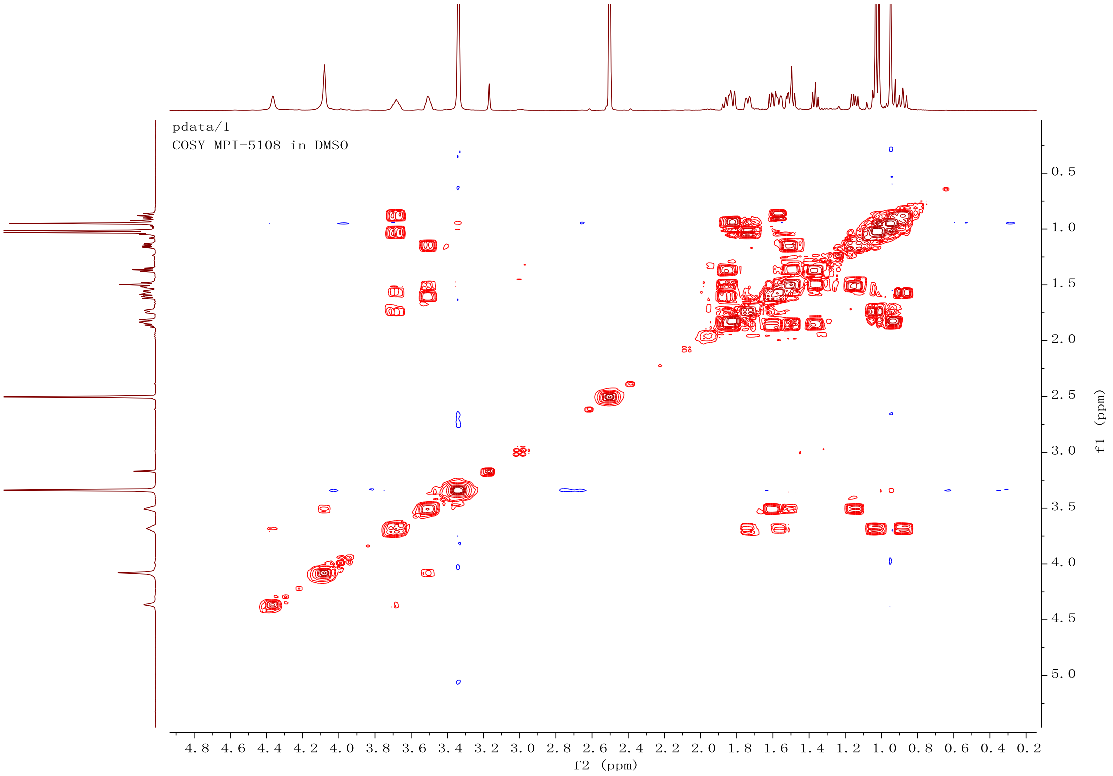

Figure S39. COSY spectrum of the new compound **4**

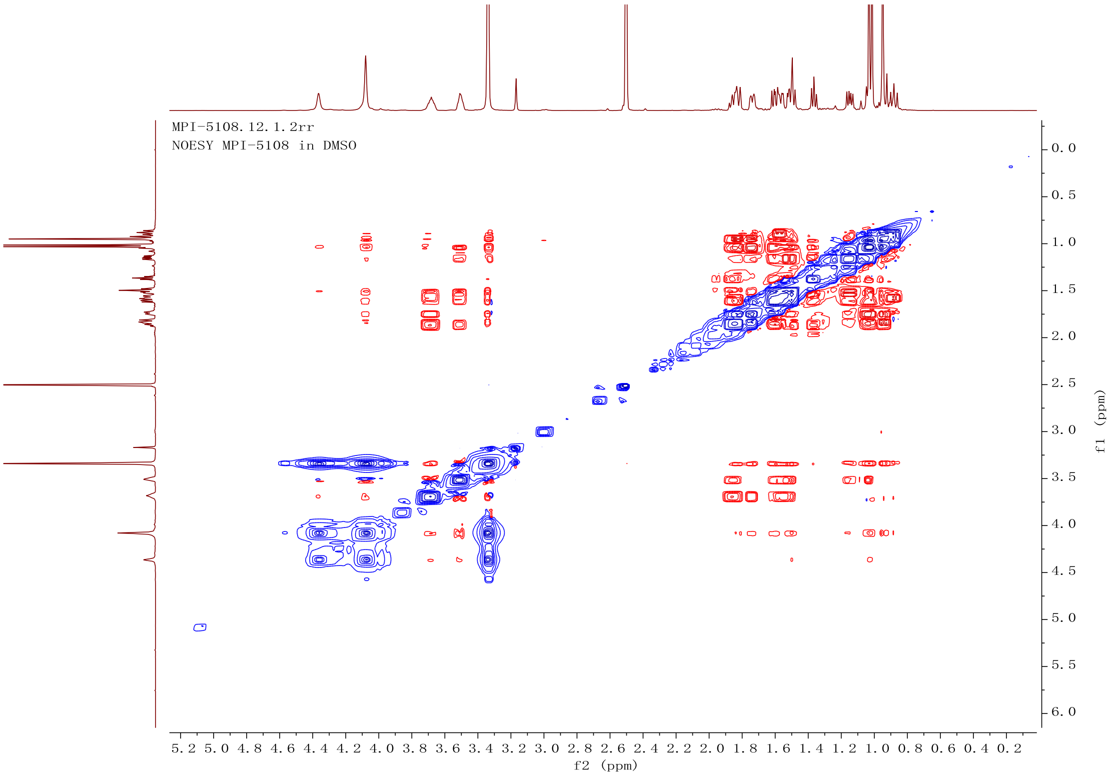

Figure S40. NOESY spectrum of the new compound **4**

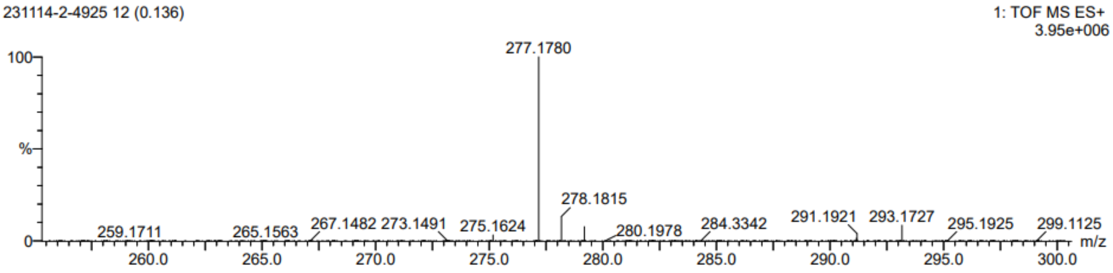

Figure S41. HRESI-MS spectrum of the new compound **13**

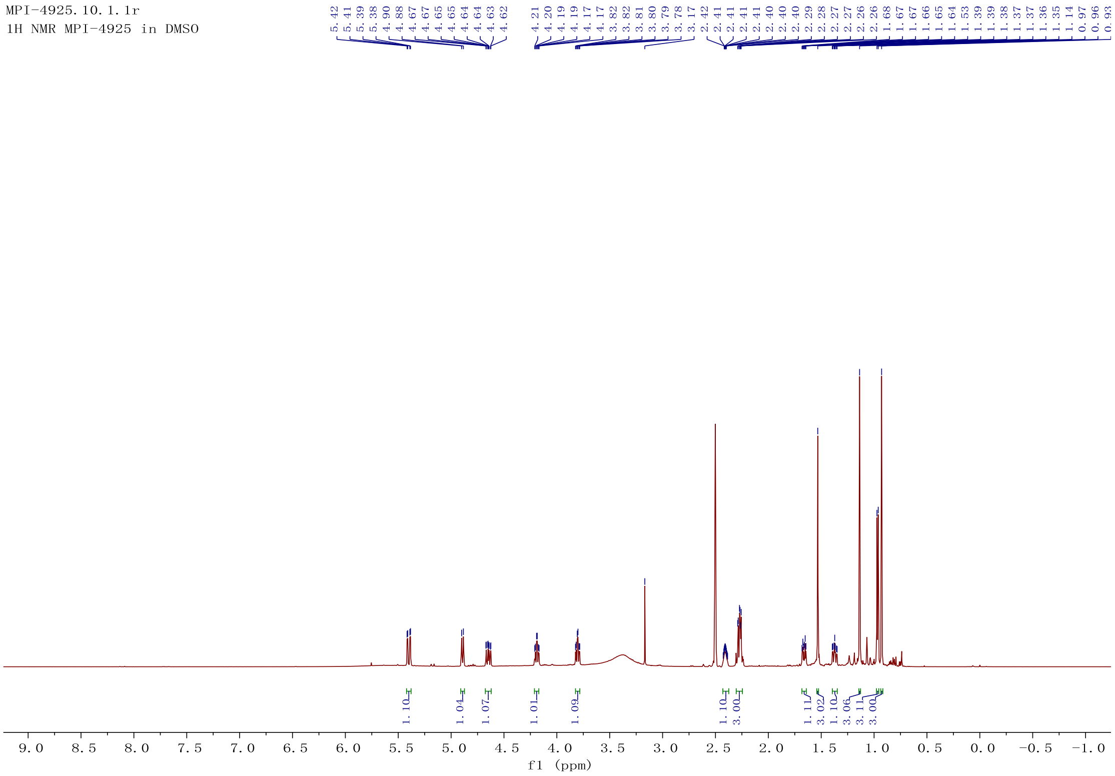

Figure S42. ^{1}H NMR (600 MHz, DMSO-d_6) spectrum of the new compound **13**

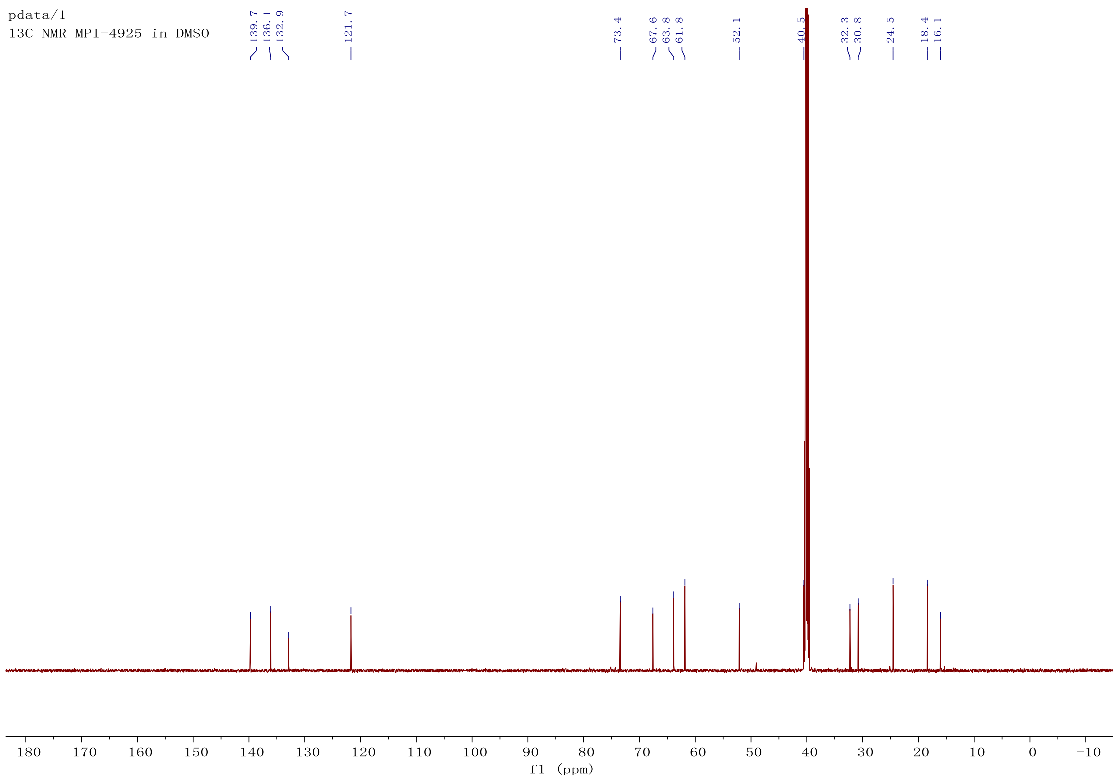

Figure S43. ^{13}C NMR (150 MHz, DMSO-d_6) spectrum of the new compound **13**

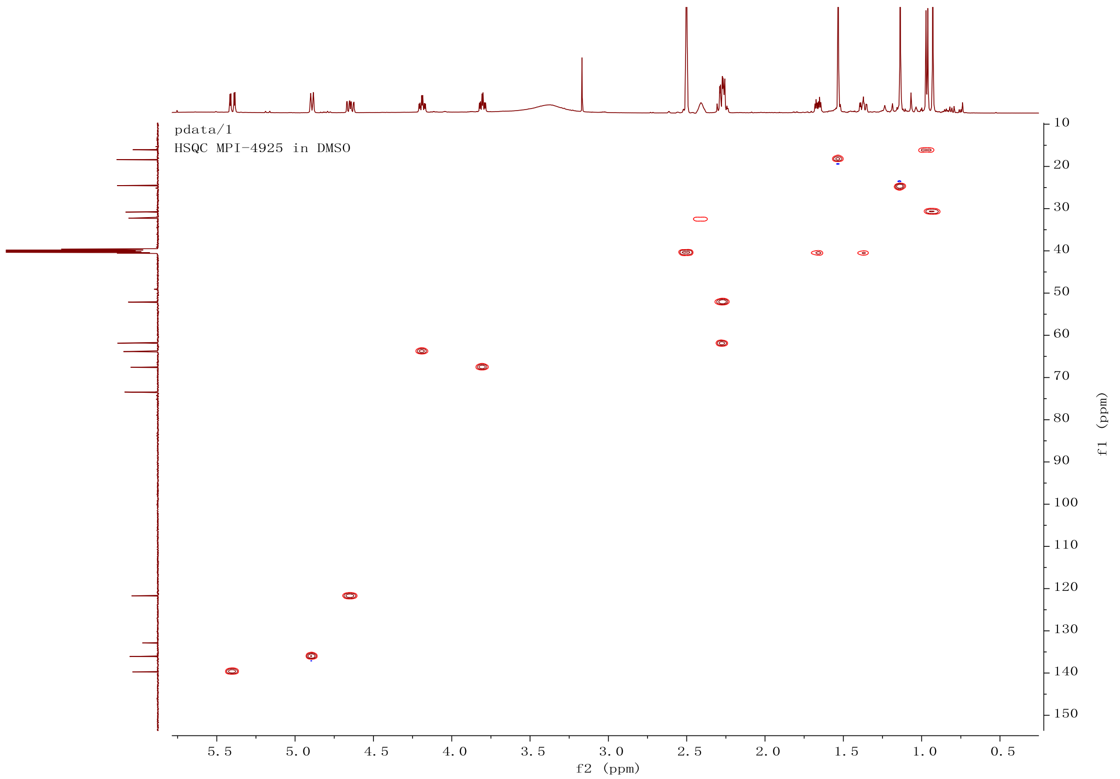

Figure S44. HSQC spectrum of the new compound **13**

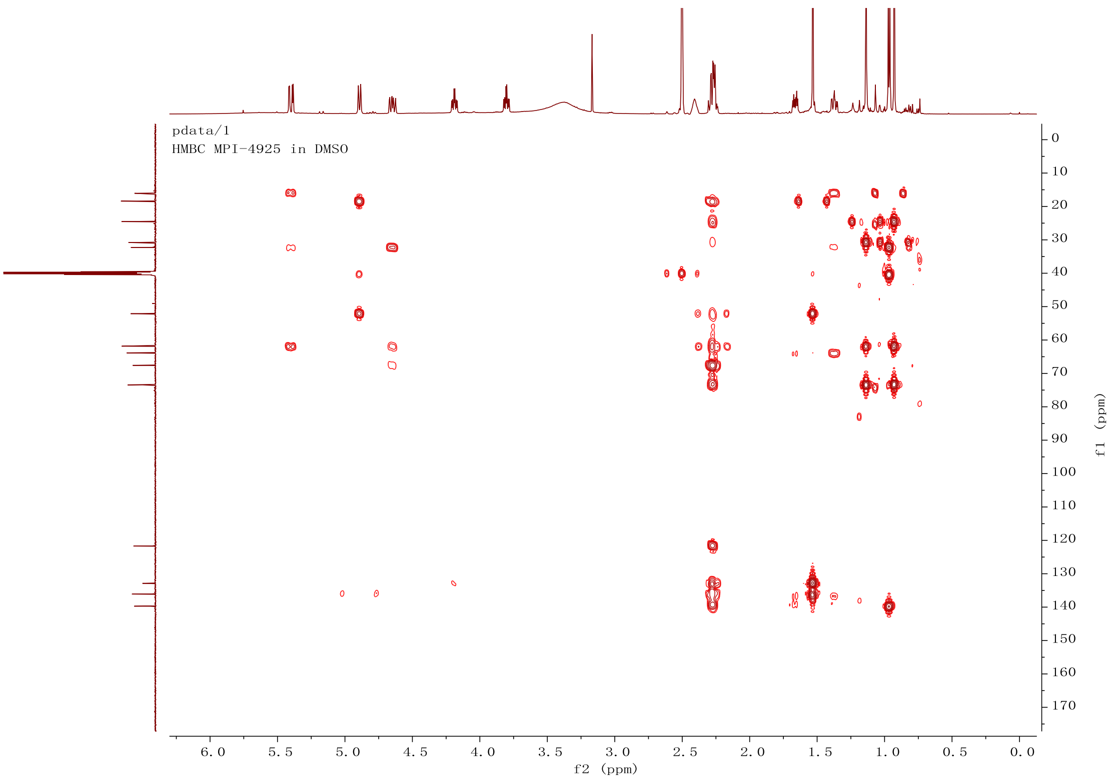

Figure S45. HMBC spectrum of the new compound **13**

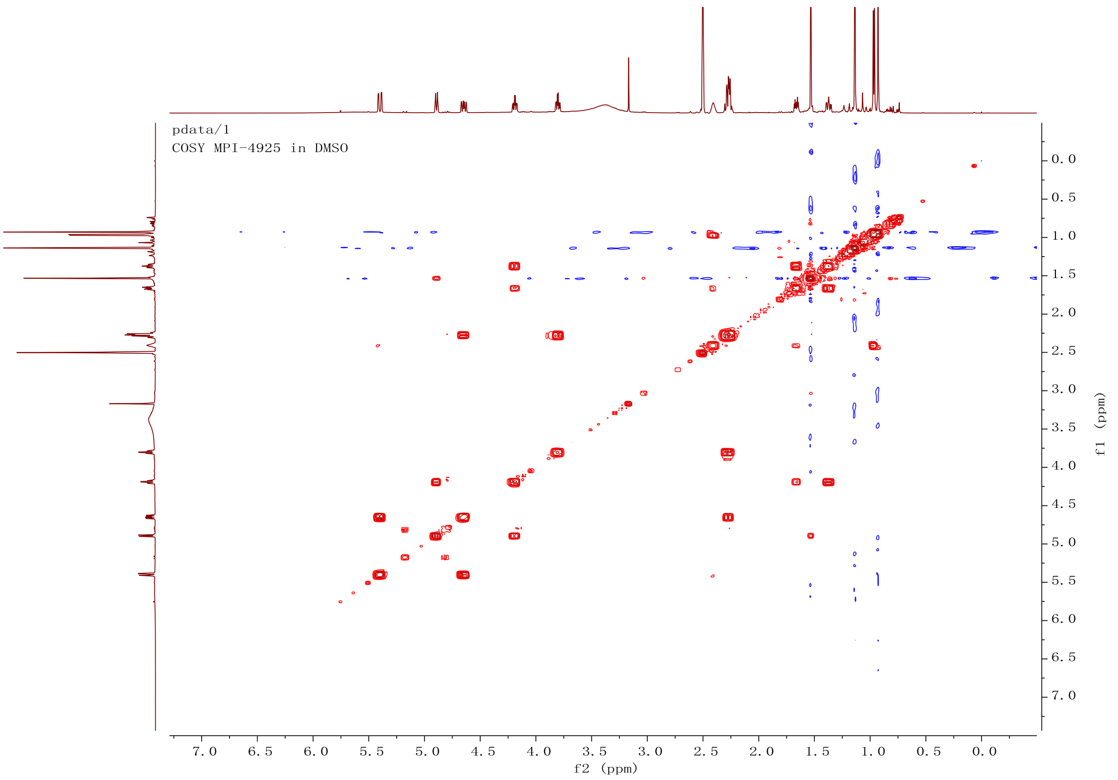

Figure S46. COSY spectrum of the new compound **13**

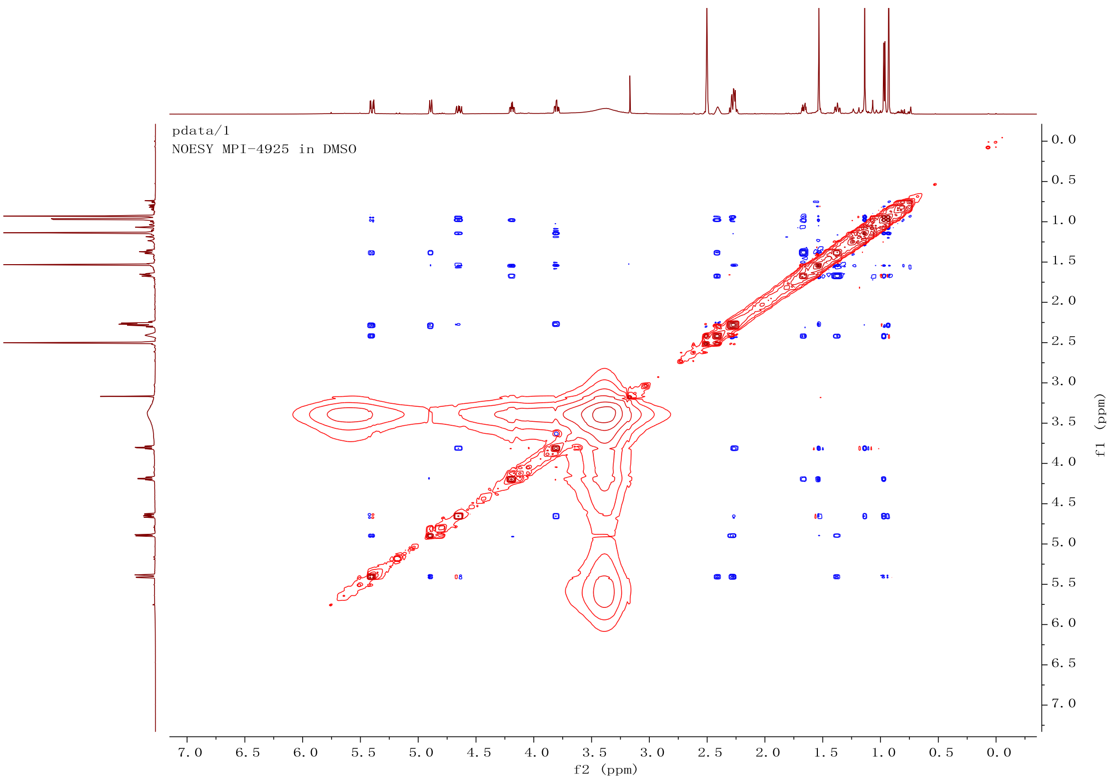

Figure S47. NOESY spectrum of the new compound **13**

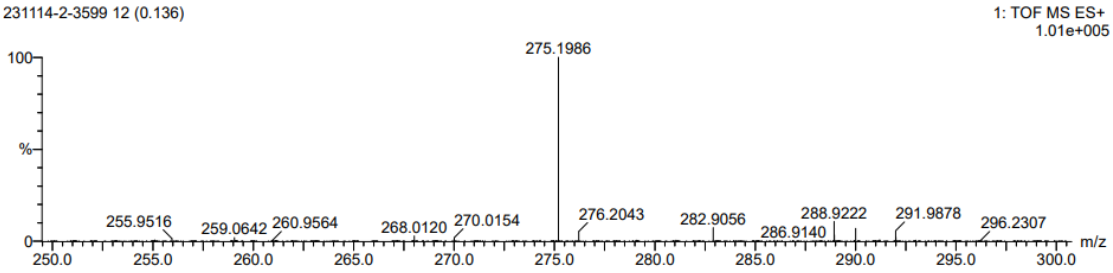

Figure S48. HRESI-MS spectrum of the new compound **14**

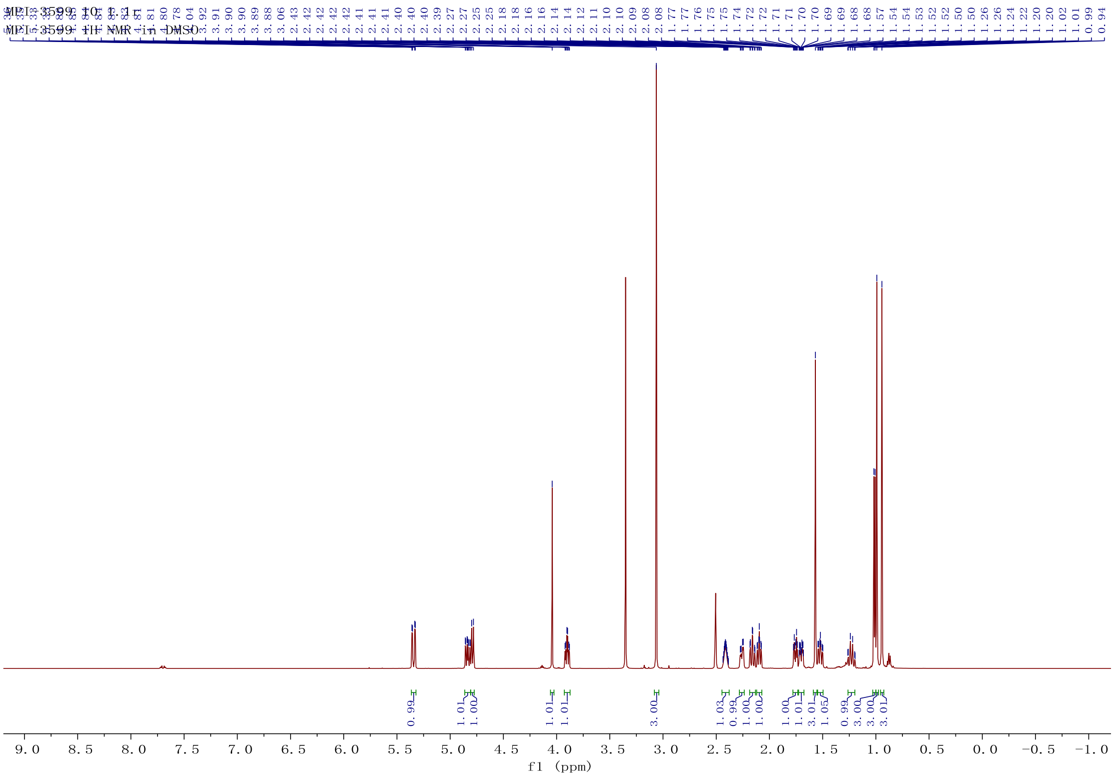

Figure S49. ^{1}H NMR (600 MHz, DMSO-d_6) spectrum of the new compound **14**

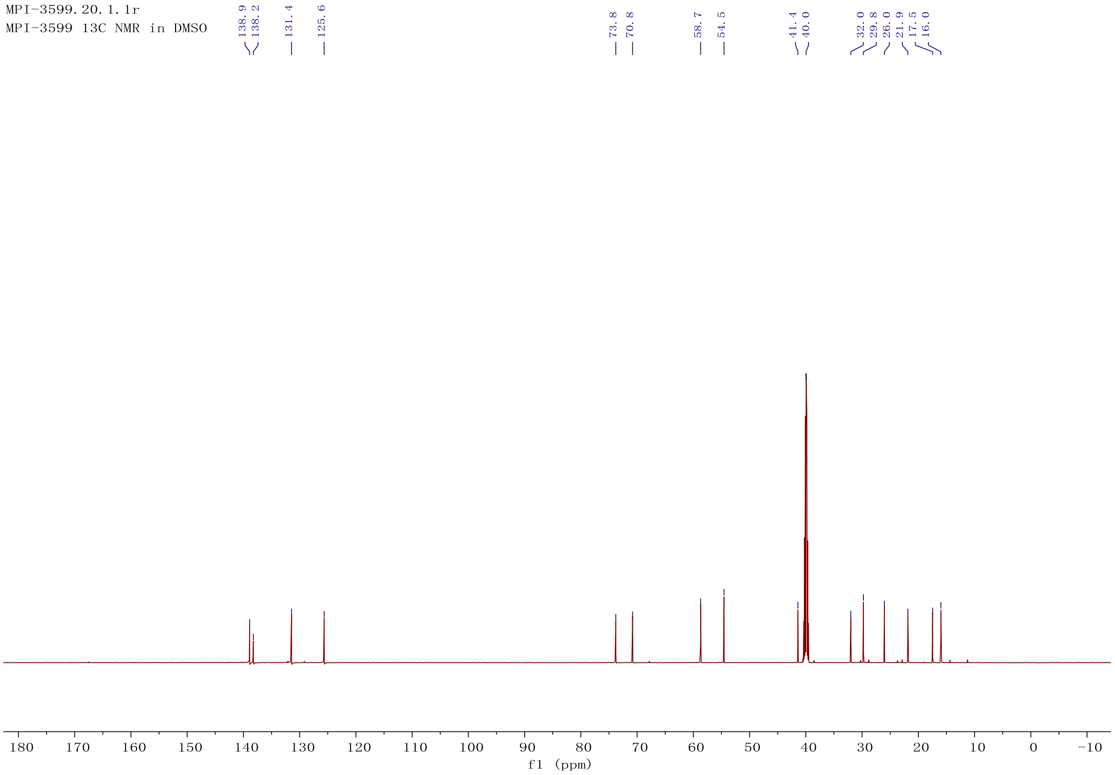

Figure S50. ^{13}C NMR (150 MHz, DMSO-d_6) spectrum of the new compound **14**

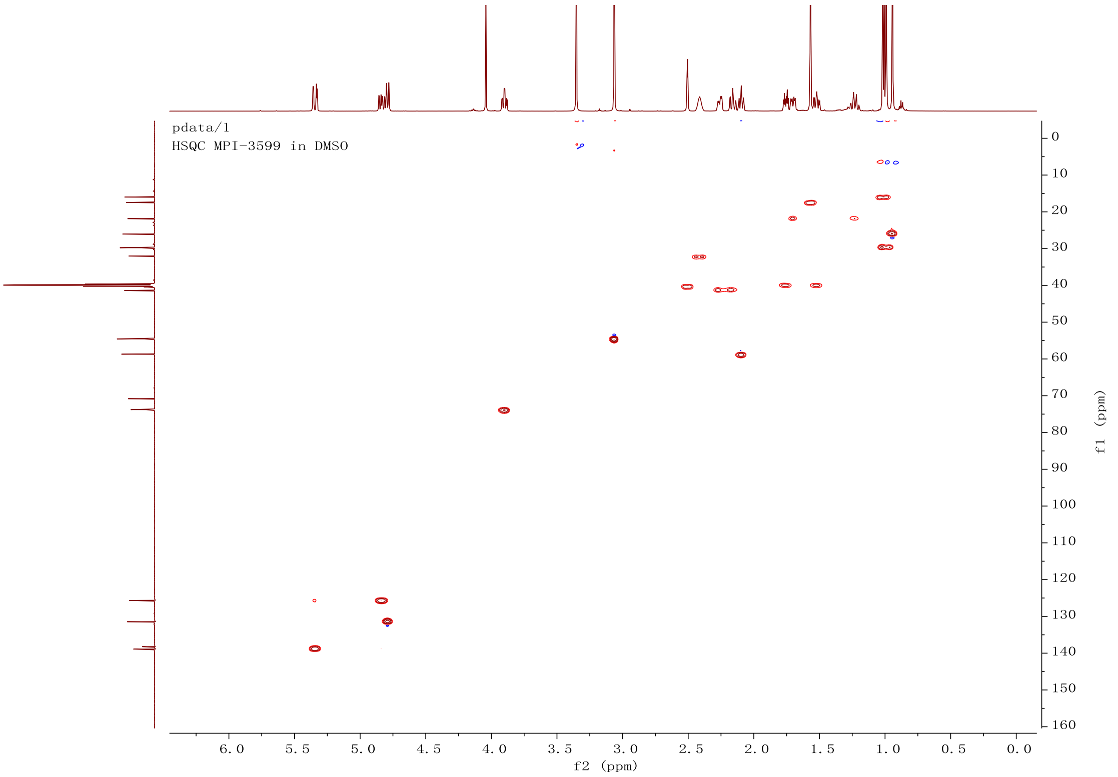

Figure S51. HSQC spectrum of the new compound **14**

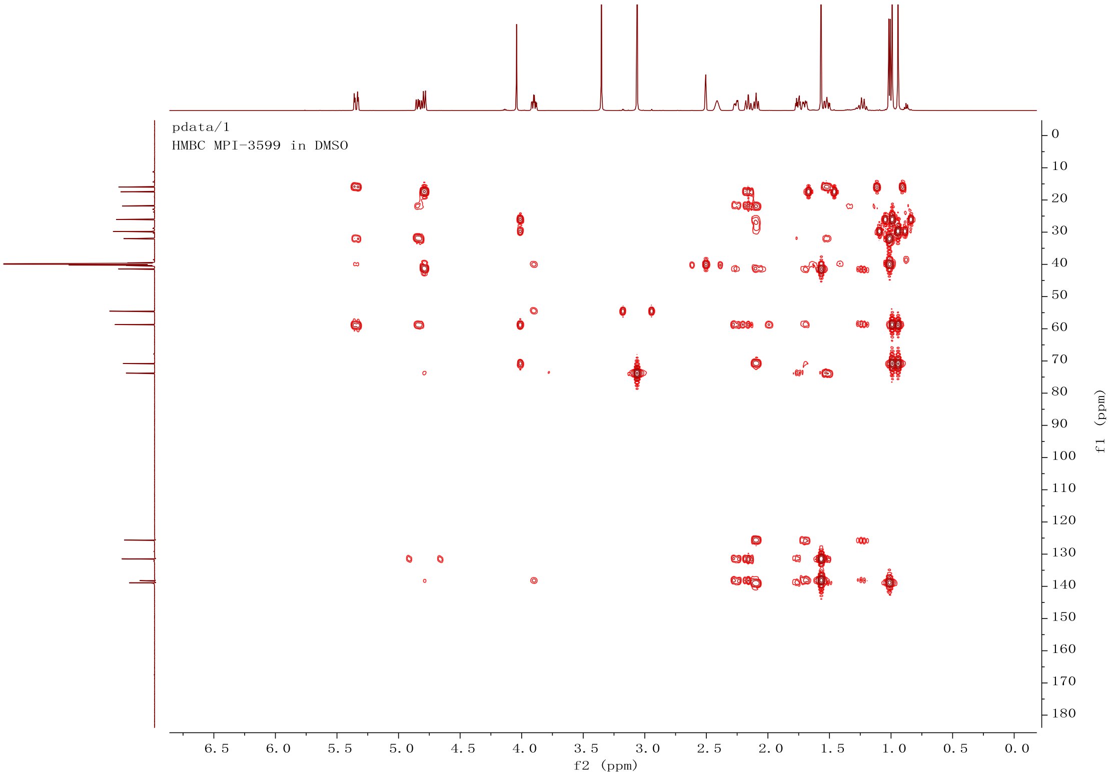

Figure S52. HMBC spectrum of the new compound **14**

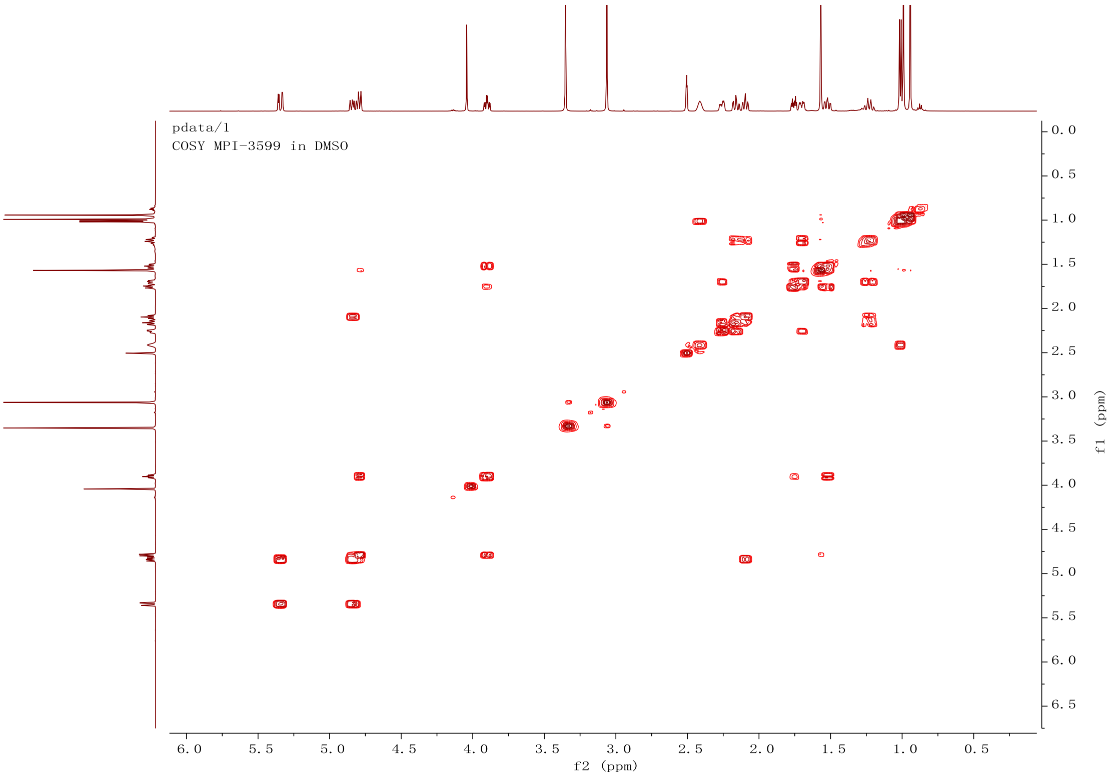

Figure S53. COSY spectrum of the new compound **14**

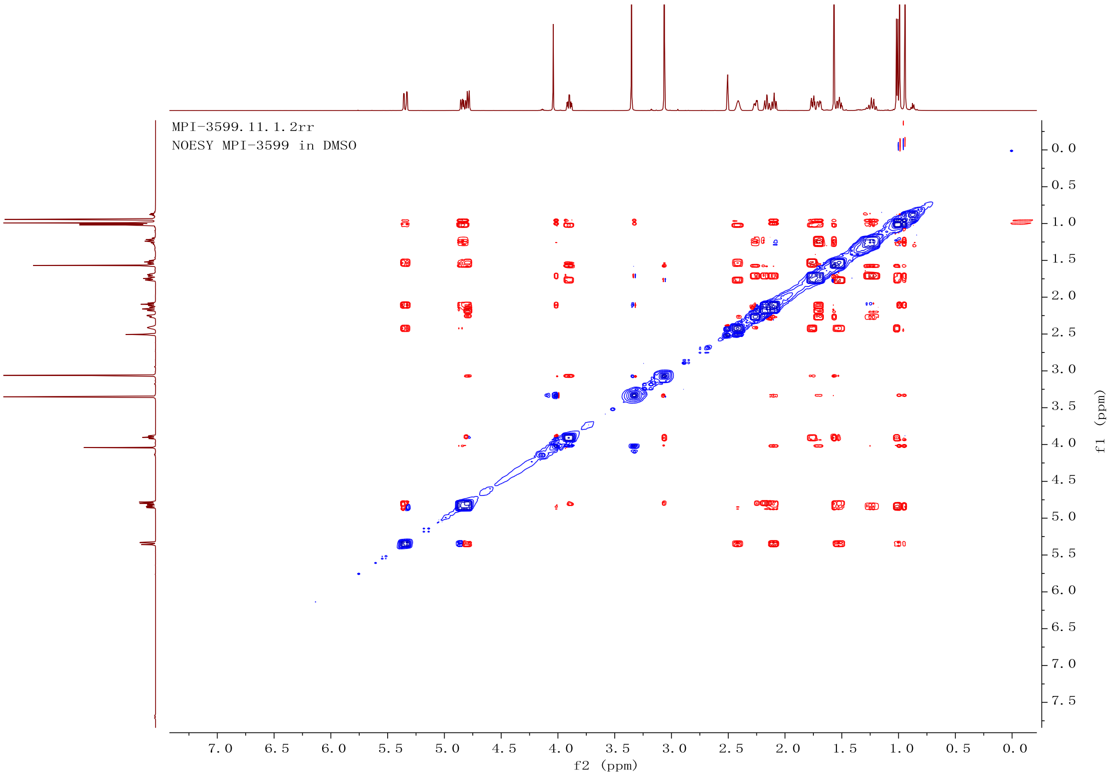

Figure S54. NOESY spectrum of the new compound **14**

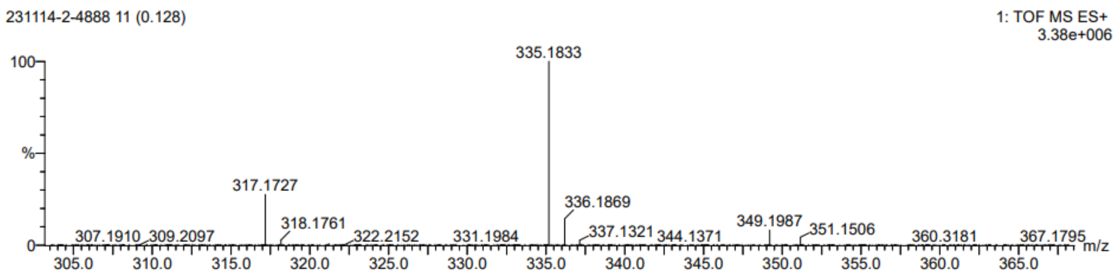

Figure S55. HRESI-MS spectrum of the new compound **15**

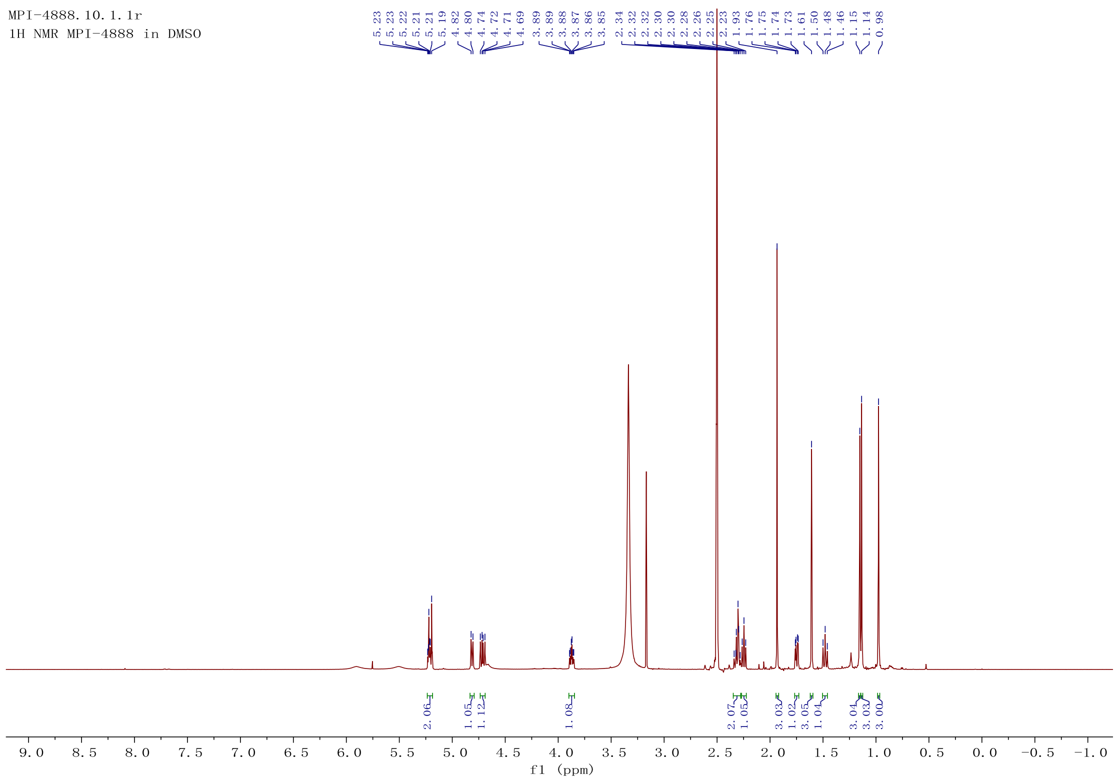

Figure S56. ^{1}H NMR (600 MHz, DMSO-d_6) spectrum of the new compound **15**

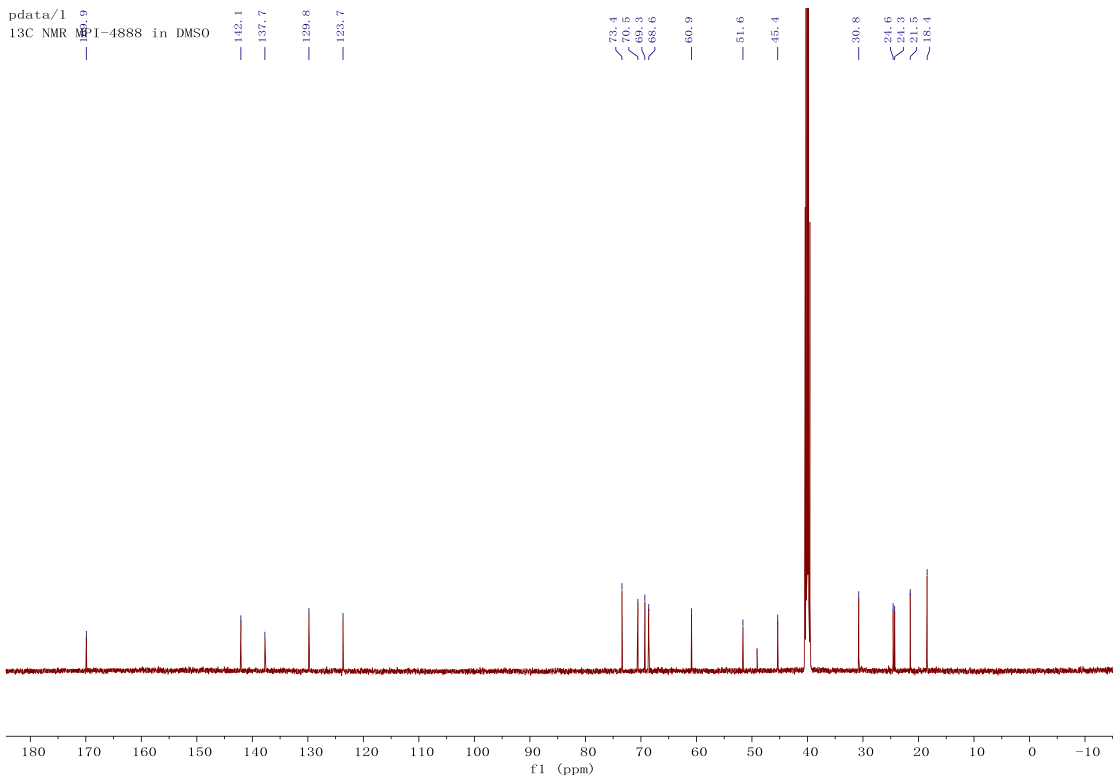

Figure S57. ^{13}C NMR (150 MHz, DMSO-d_6) spectrum of the new compound **15**

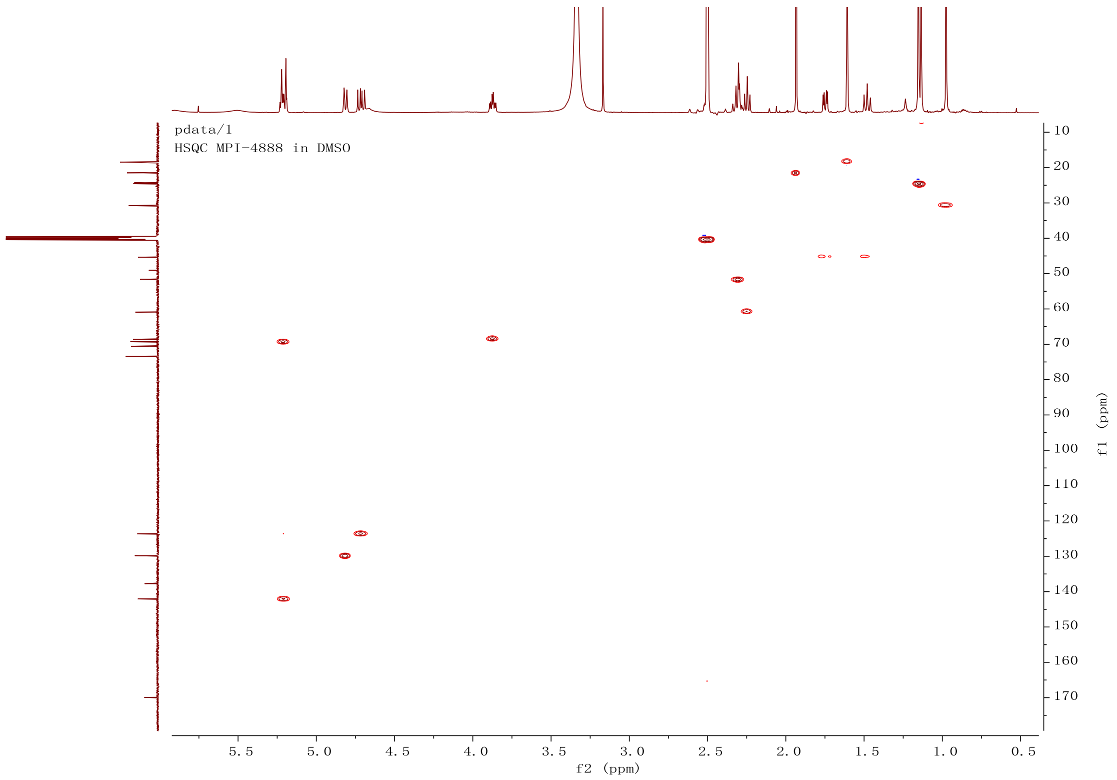

Figure S58. HSQC spectrum of the new compound **15**

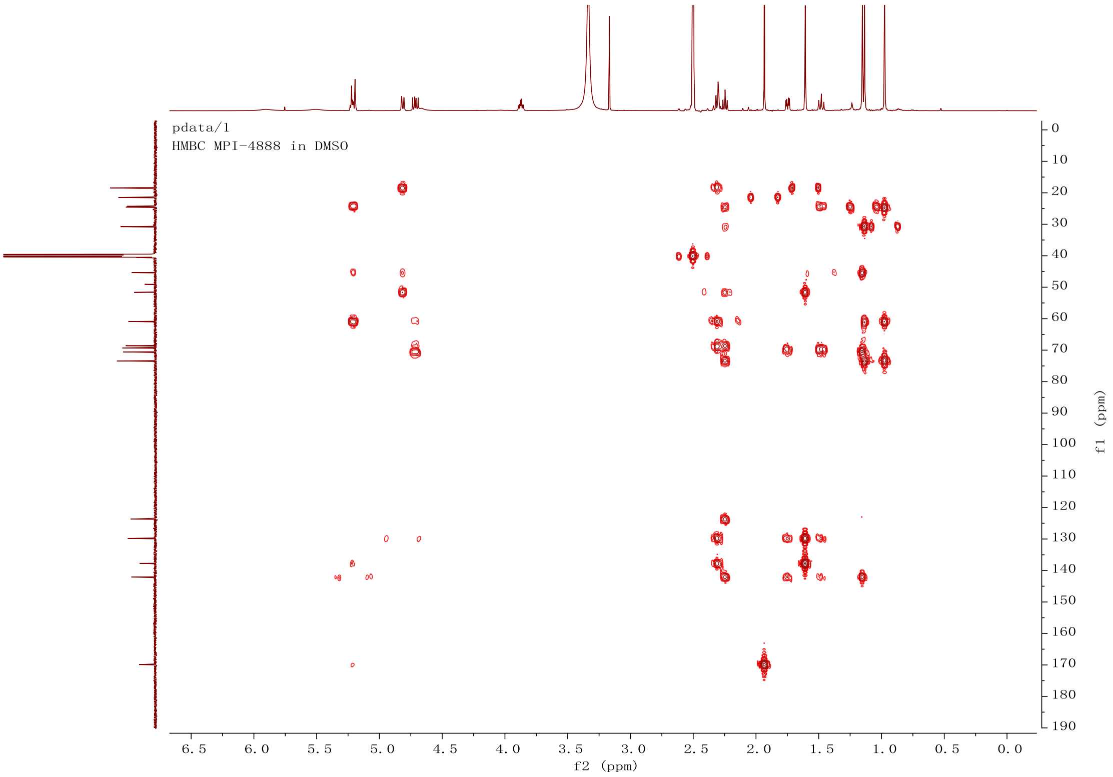

Figure S59. HMBC spectrum of the new compound **15**

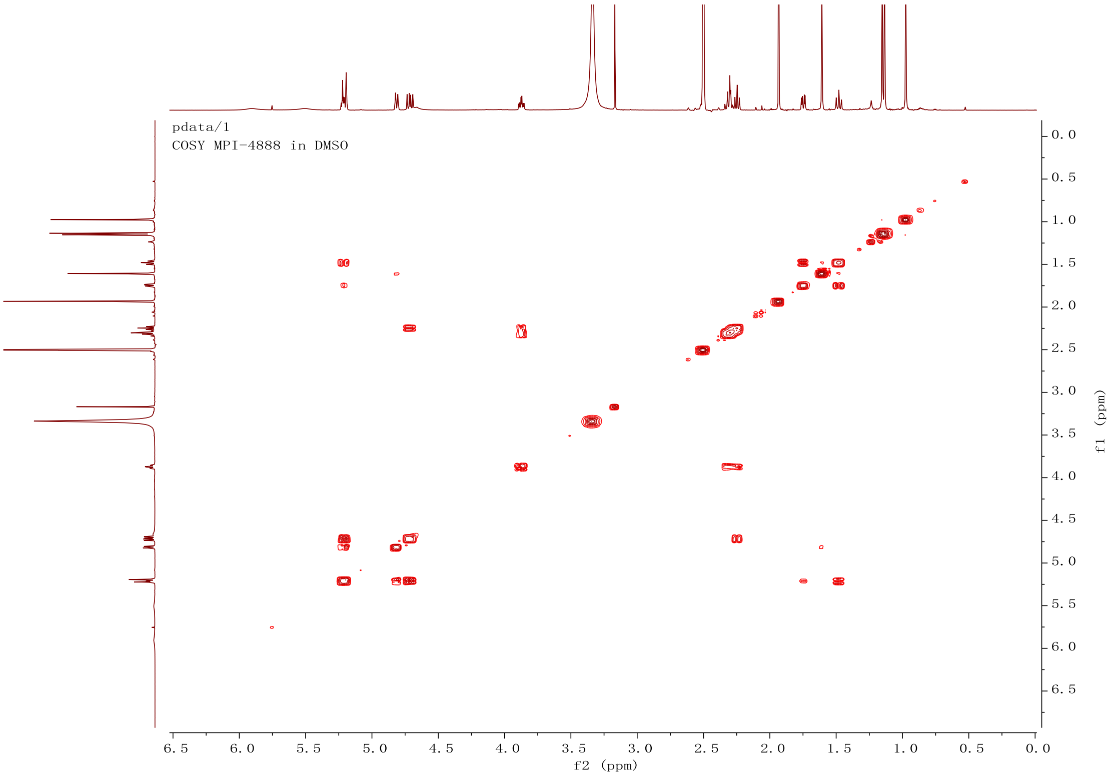

Figure S60. COSY spectrum of the new compound **15**

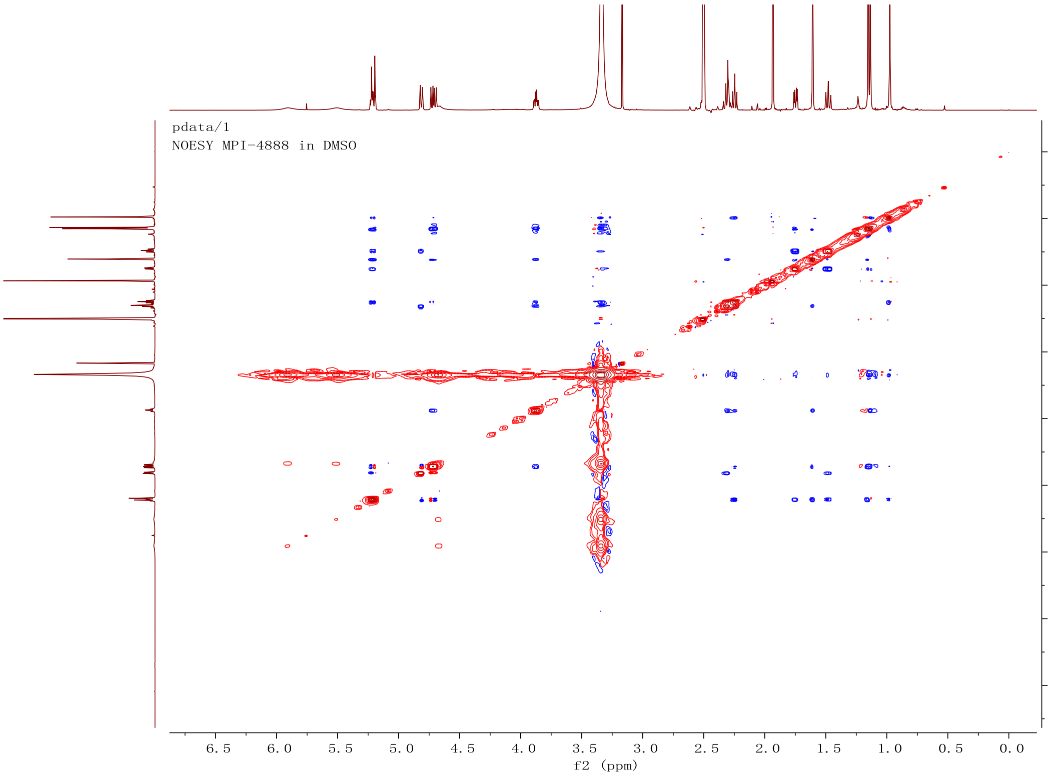

Figure S61. NOESY spectrum of the new compound **15**

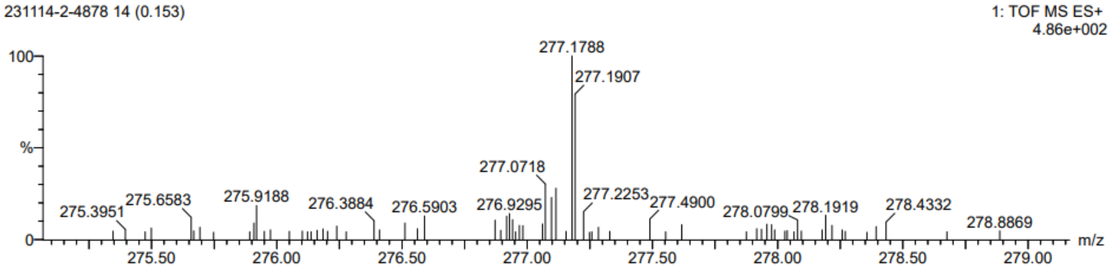

Figure S62. HRESI-MS spectrum of the new compound **17**

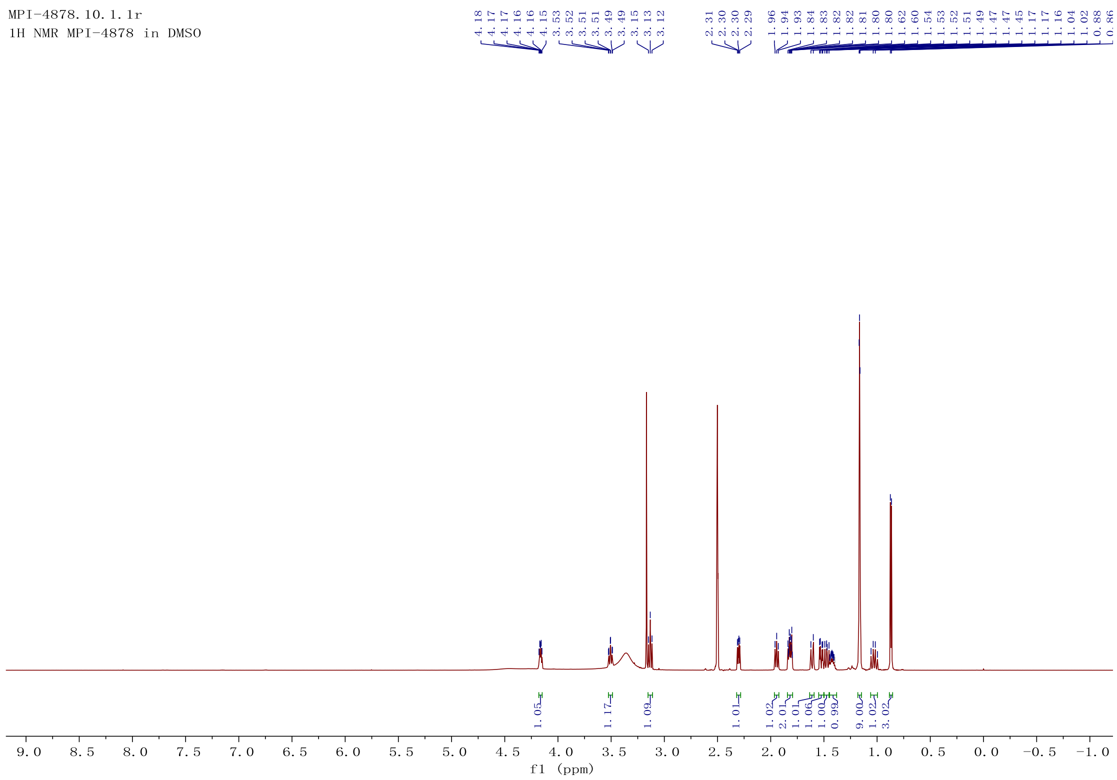

Figure S63. ^{1}H NMR (600 MHz, DMSO-d_6) spectrum of the new compound **17**

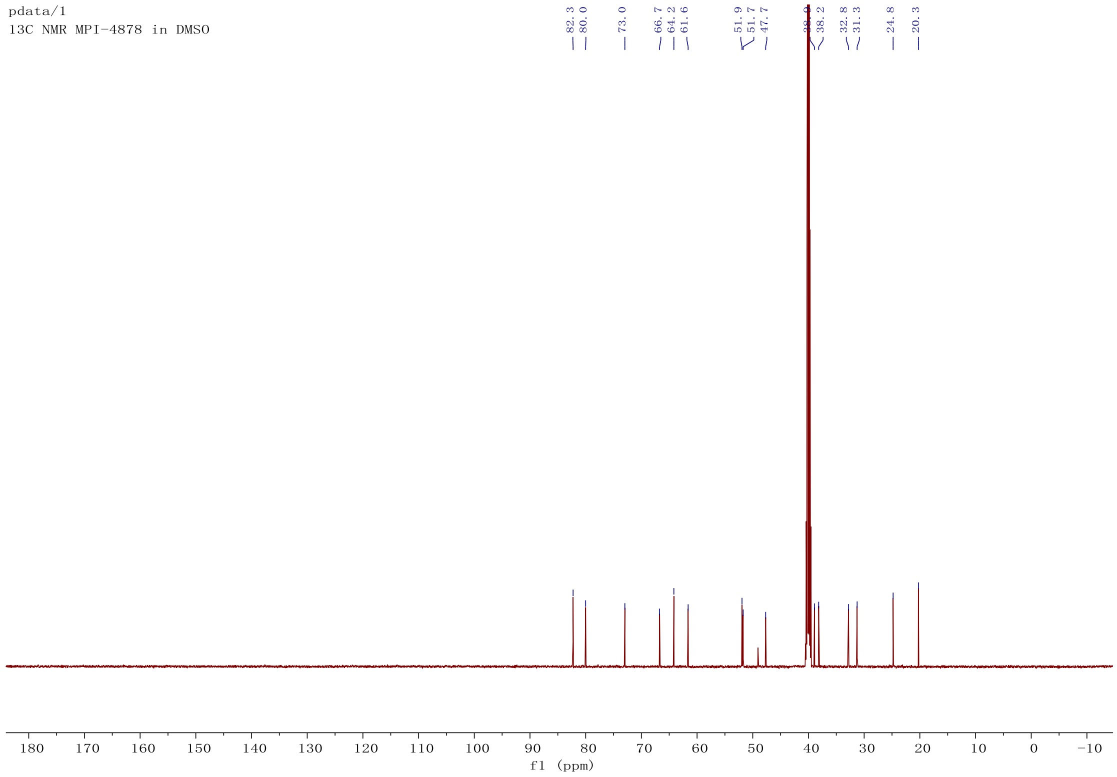

Figure S64. ^{13}C NMR (150 MHz, DMSO-d_6) spectrum of the new compound **17**

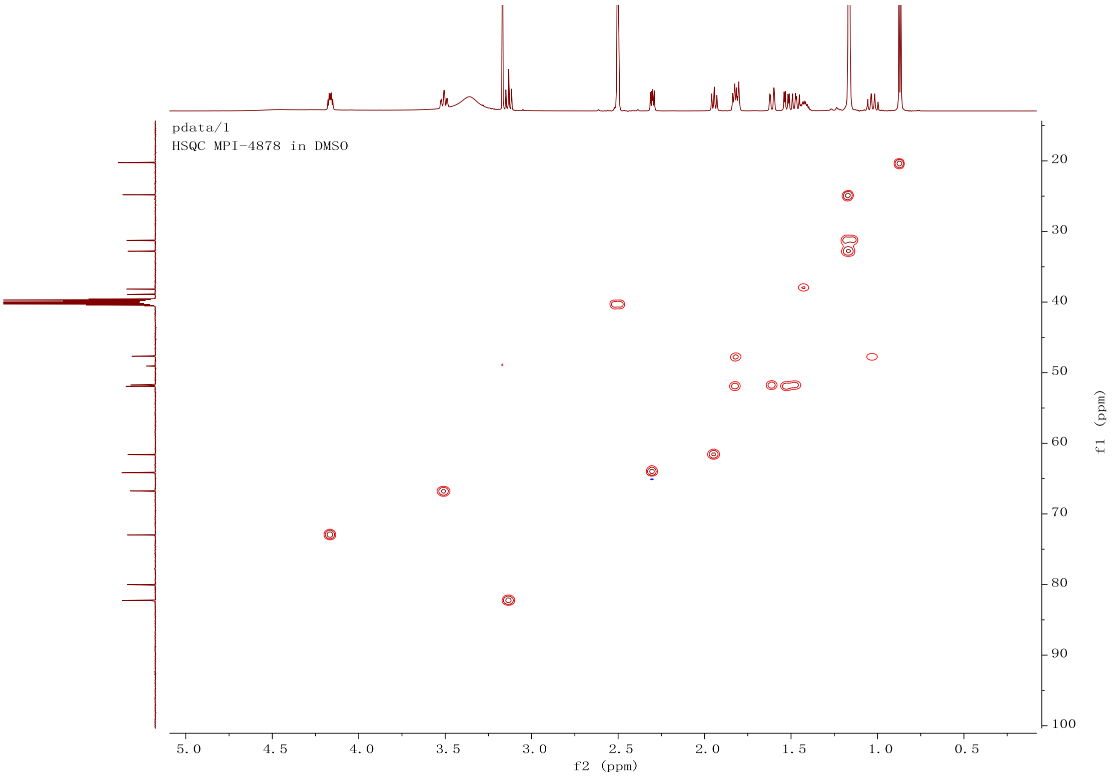

Figure S65. HSQC spectrum of the new compound **17**

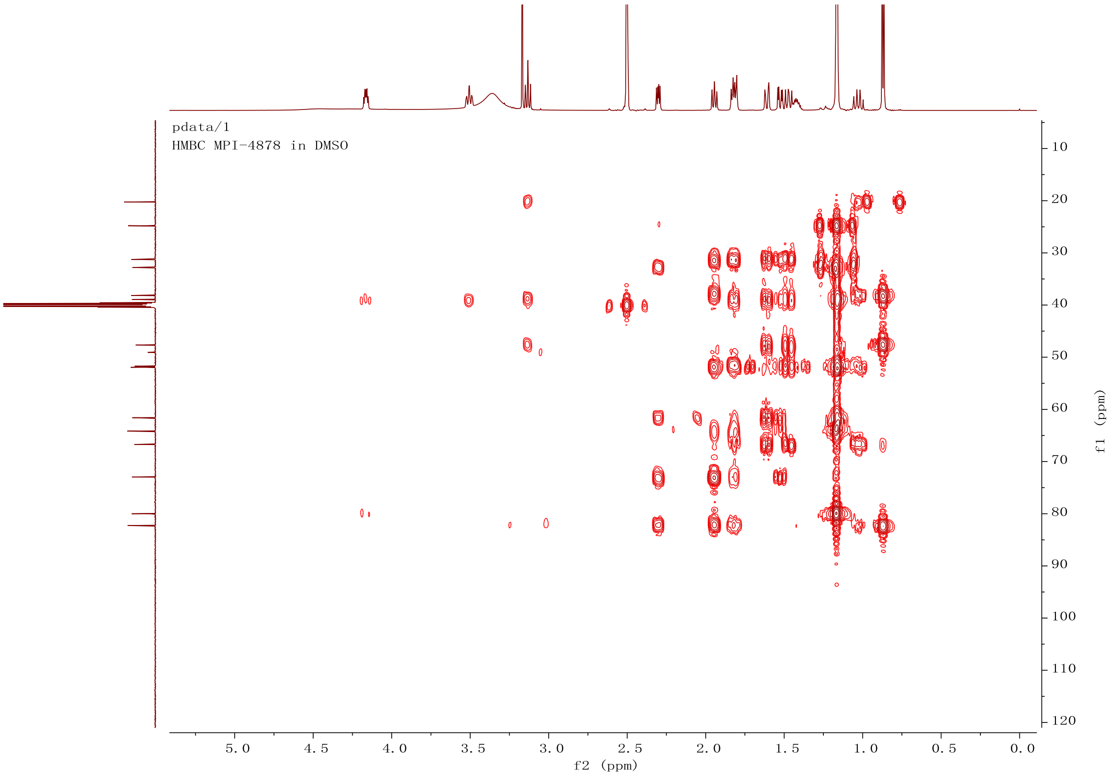

Figure S66. HMBC spectrum of the new compound **17**

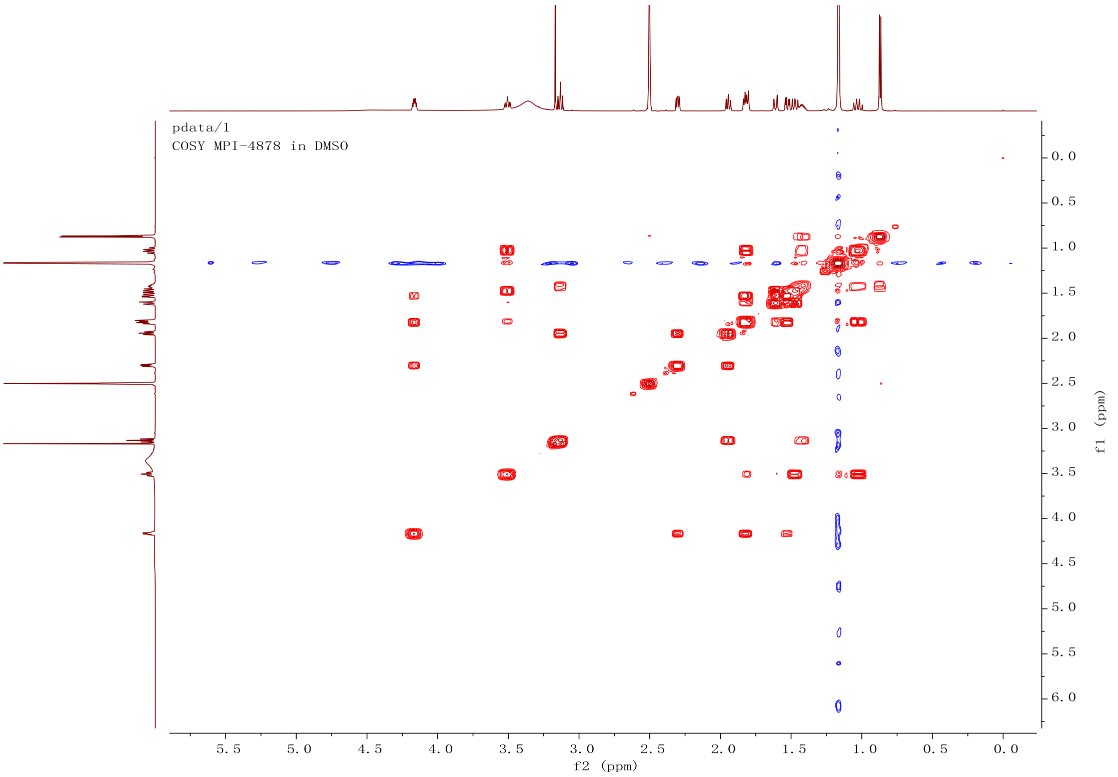

Figure S67. COSY spectrum of the new compound **17**

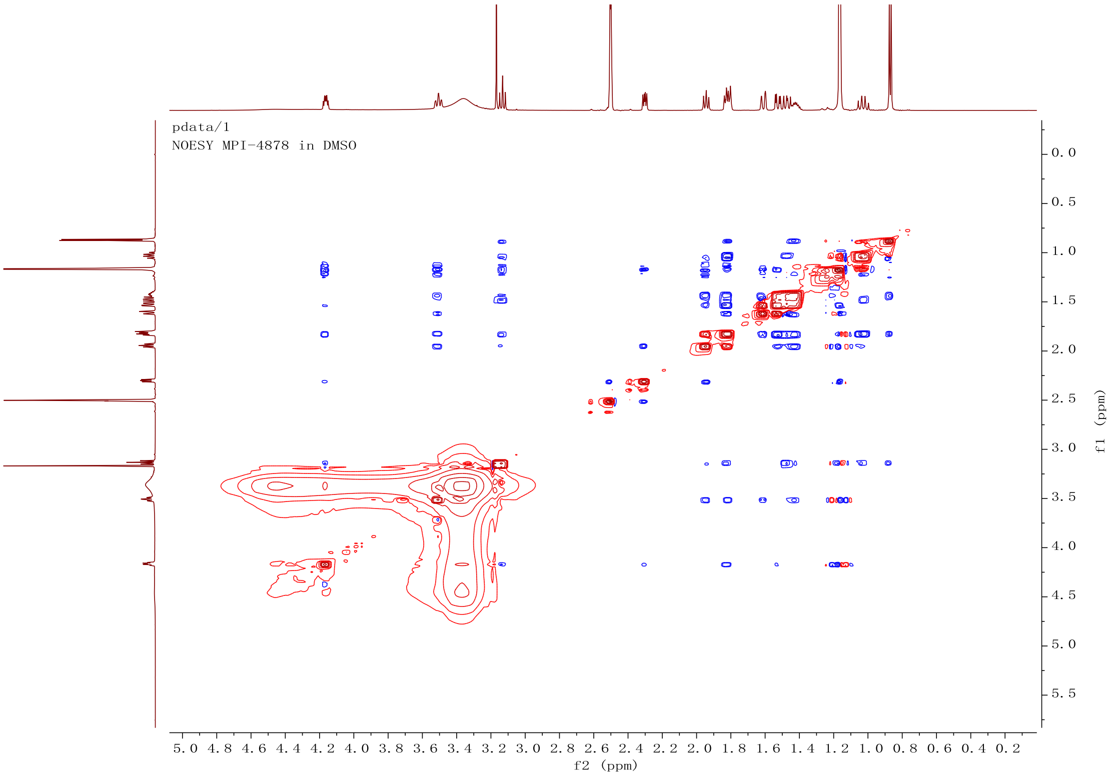

Figure S68. NOESY spectrum of the new compound **17**

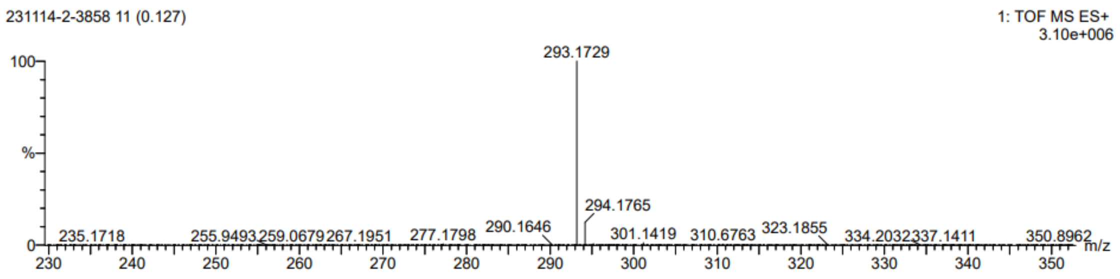

Figure S69. HRESI-MS spectrum of the new compound **18**

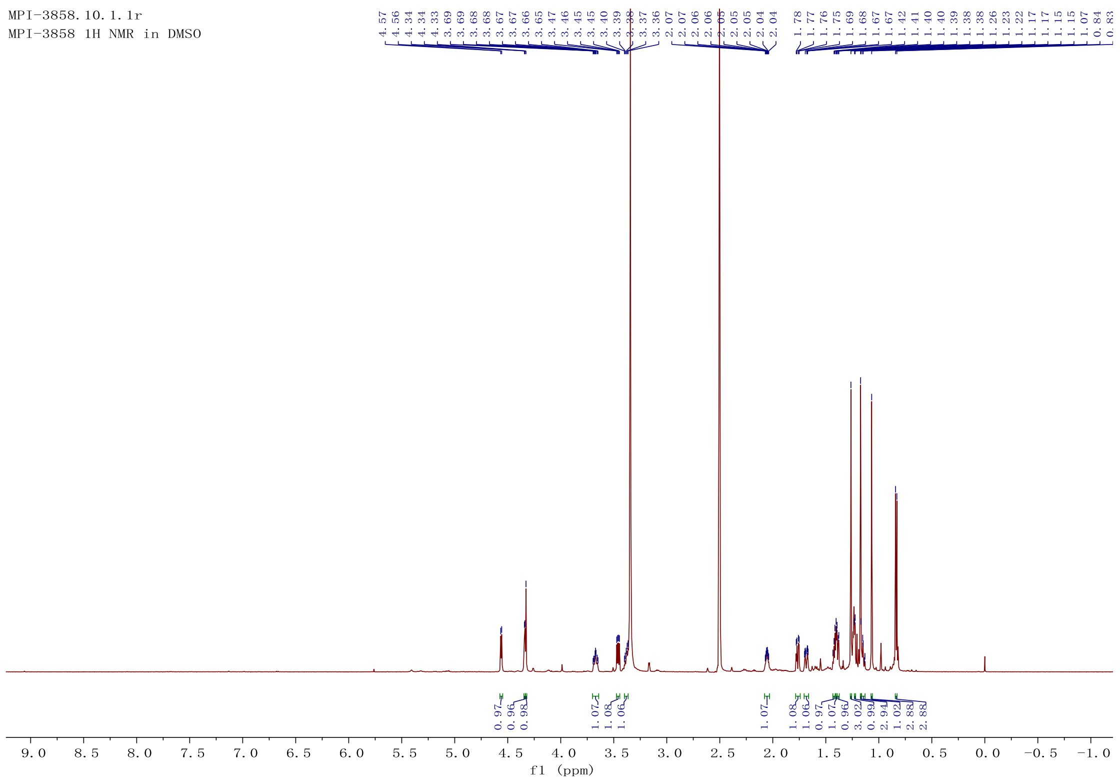

Figure S70. ^{1}H NMR (600 MHz, DMSO-d_6) spectrum of the new compound **18**

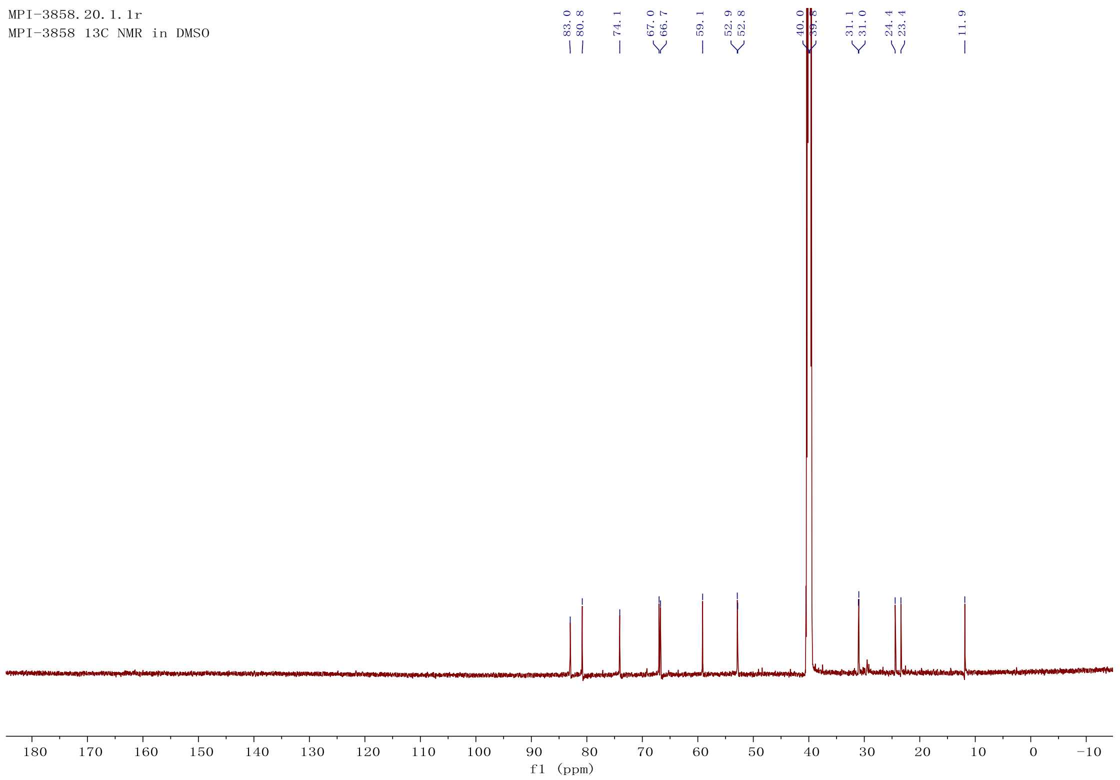

Figure S71. ^{13}C NMR (150 MHz, DMSO-d_6) spectrum of the new compound **18**

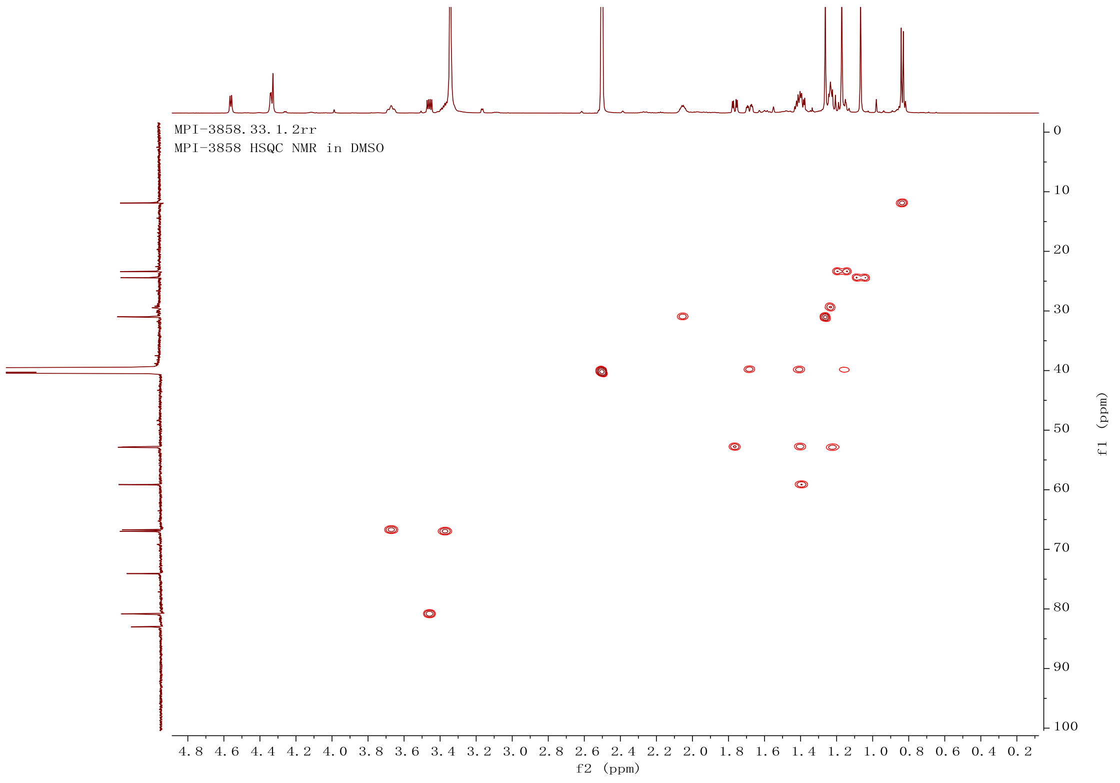

Figure S72. HSQC spectrum of the new compound **18**

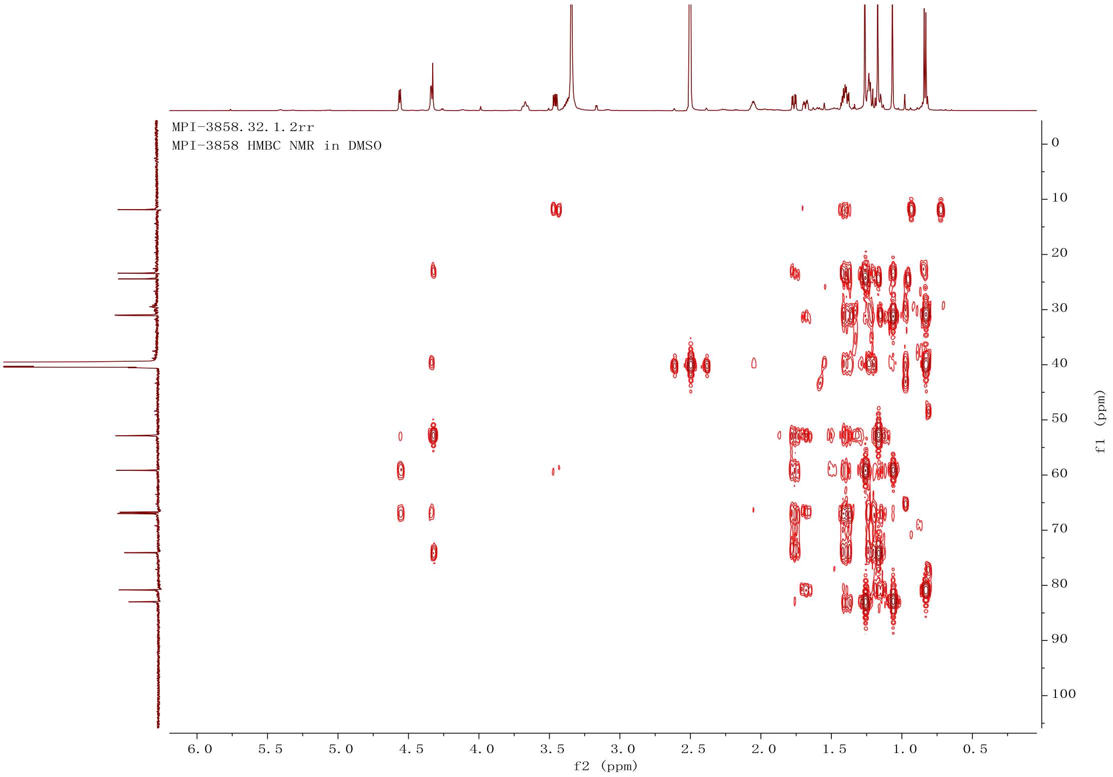

Figure S73. HMBC spectrum of the new compound **18**

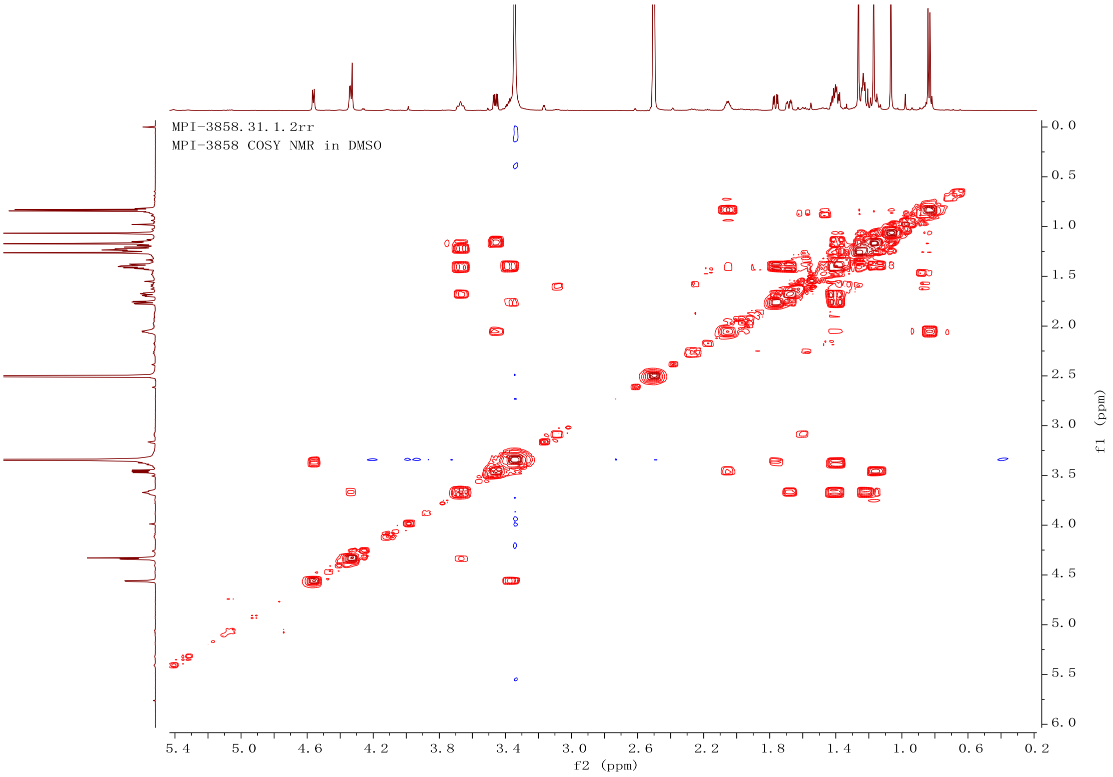

Figure S74. COSY spectrum of the new compound **18**

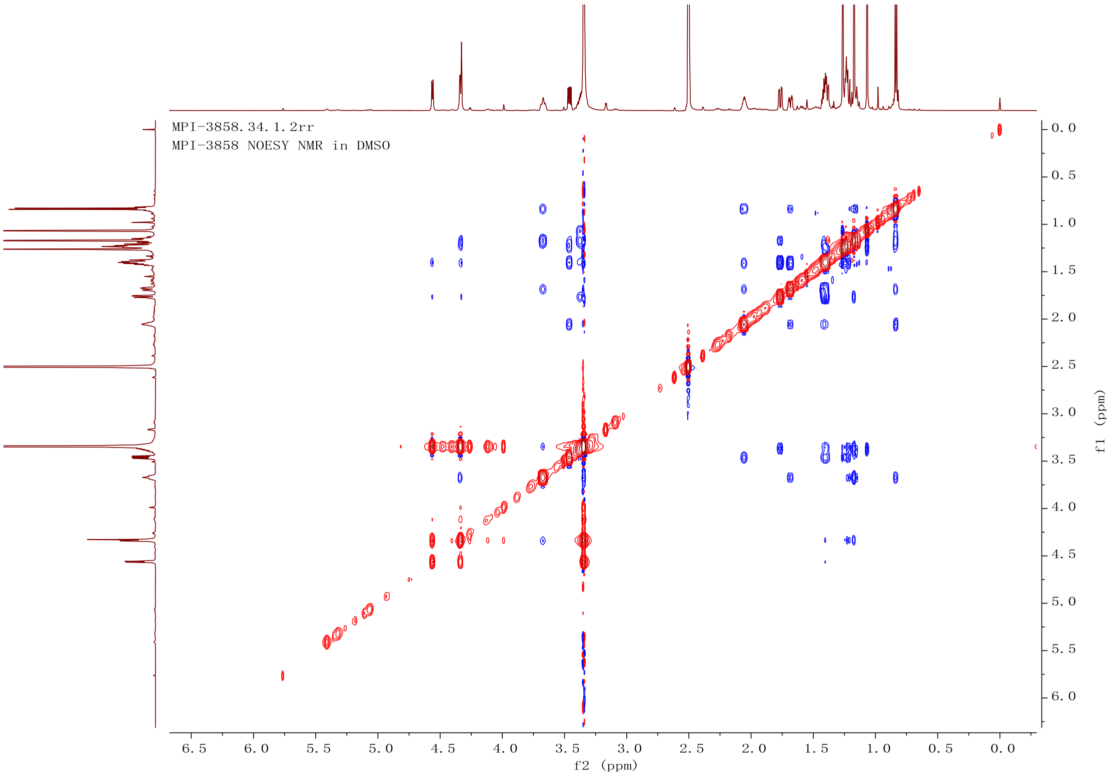

Figure S75. NOESY spectrum of the new compound **18**

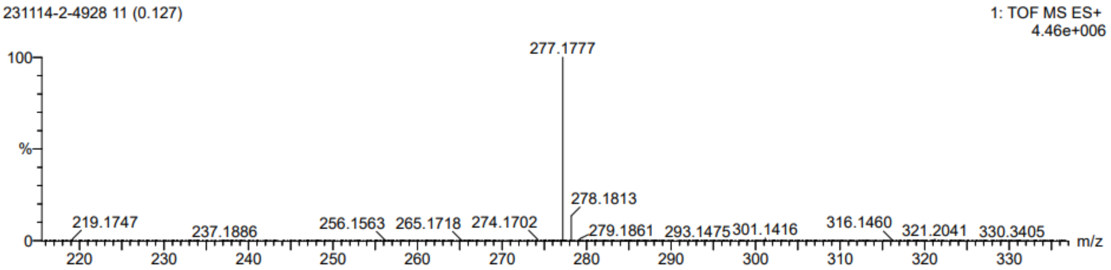

Figure S76. HRESI-MS spectrum of the new compound **22**

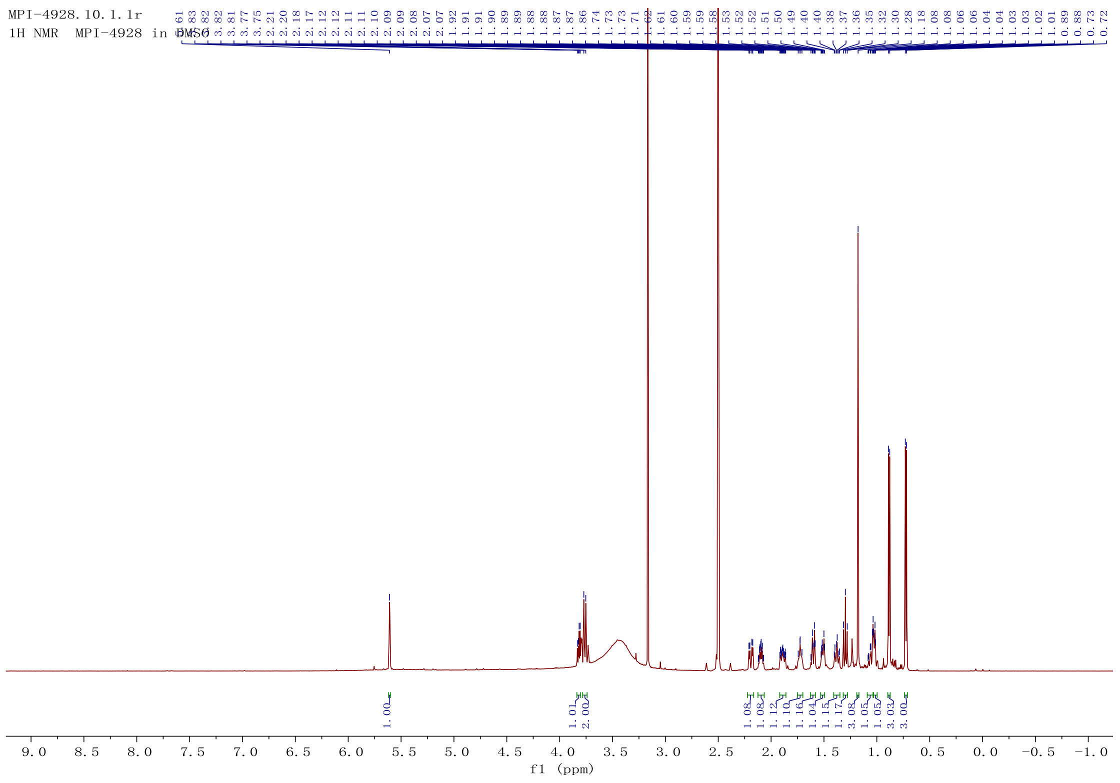

Figure S77. ^{1}H NMR (600 MHz, DMSO-d_6) spectrum of the new compound **22**

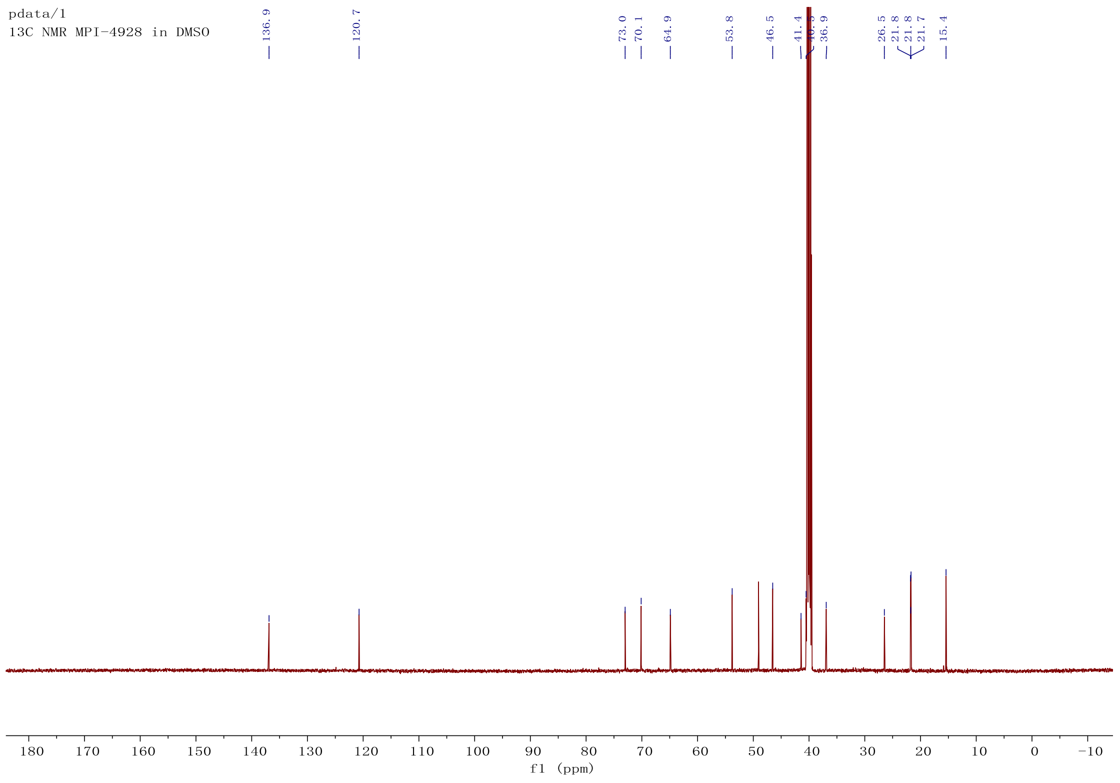

Figure S78. ^{13}C NMR (150 MHz, DMSO-d_6) spectrum of the new compound **22**

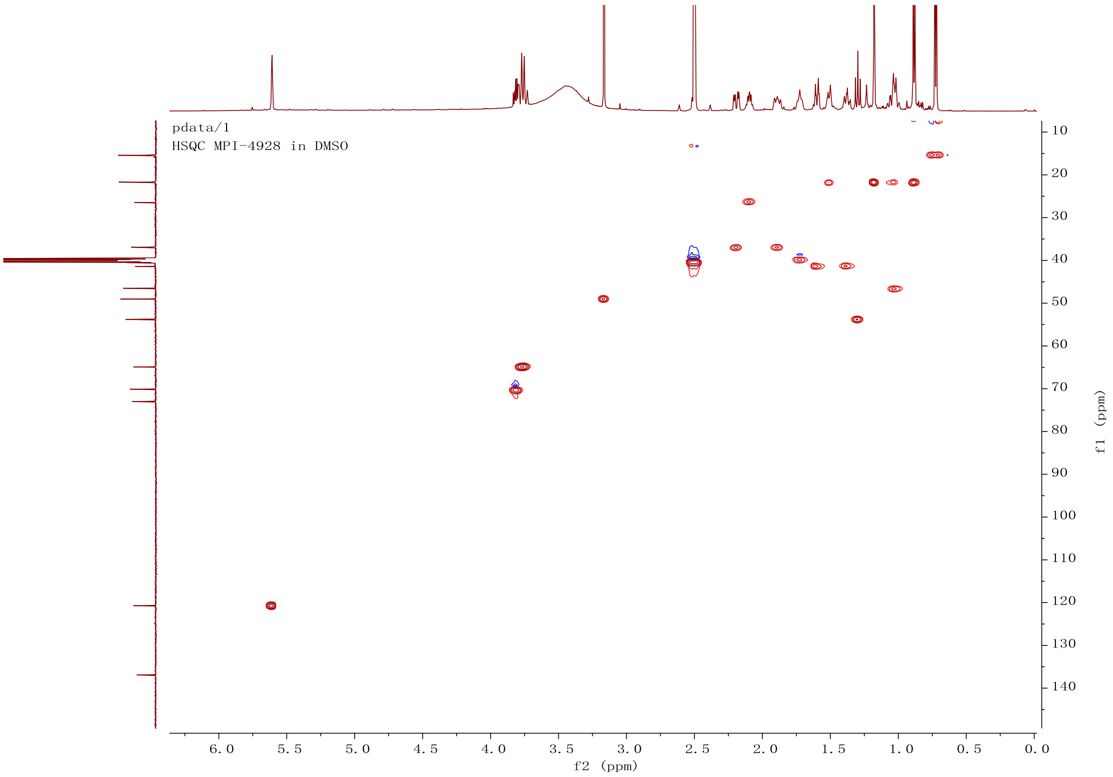

Figure S79. HSQC spectrum of the new compound **22**

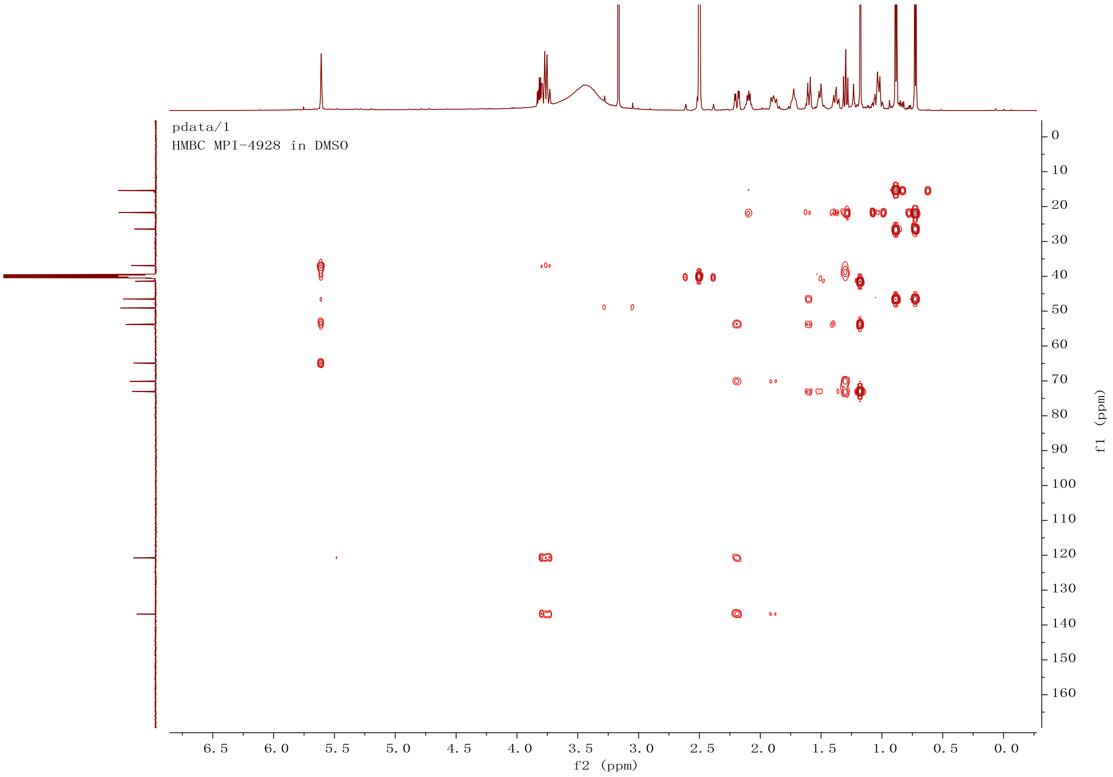

Figure S80. HMBC spectrum of the new compound **22**

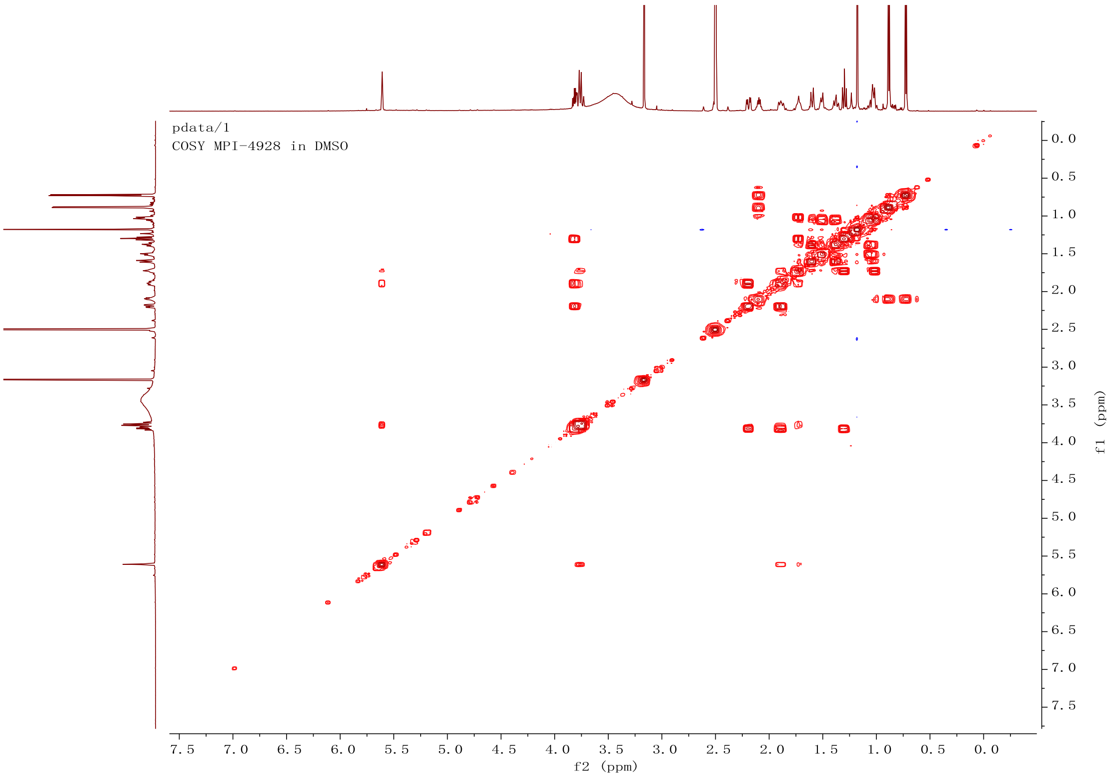

Figure S81. COSY spectrum of the new compound **22**

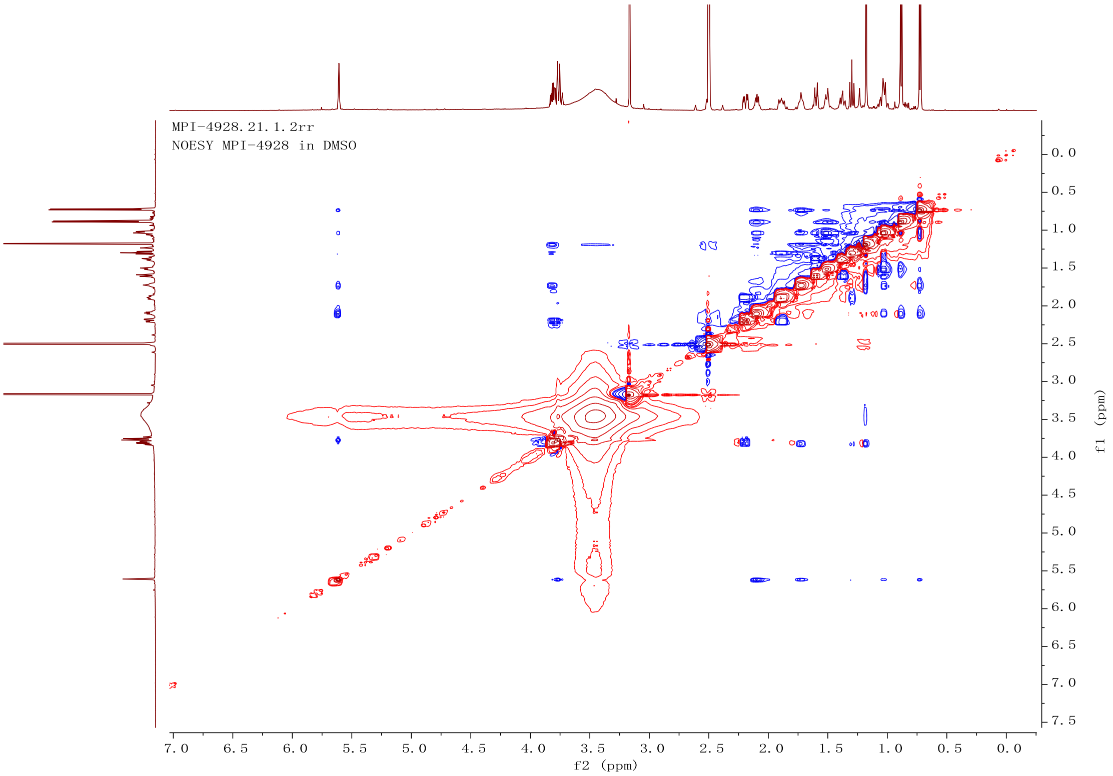

Figure S82. NOESY spectrum of the new compound **22**

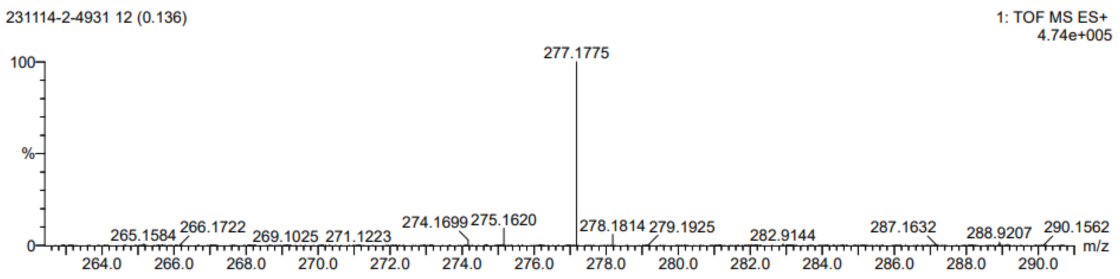

Figure S83. HRESI-MS spectrum of the new compound **23**

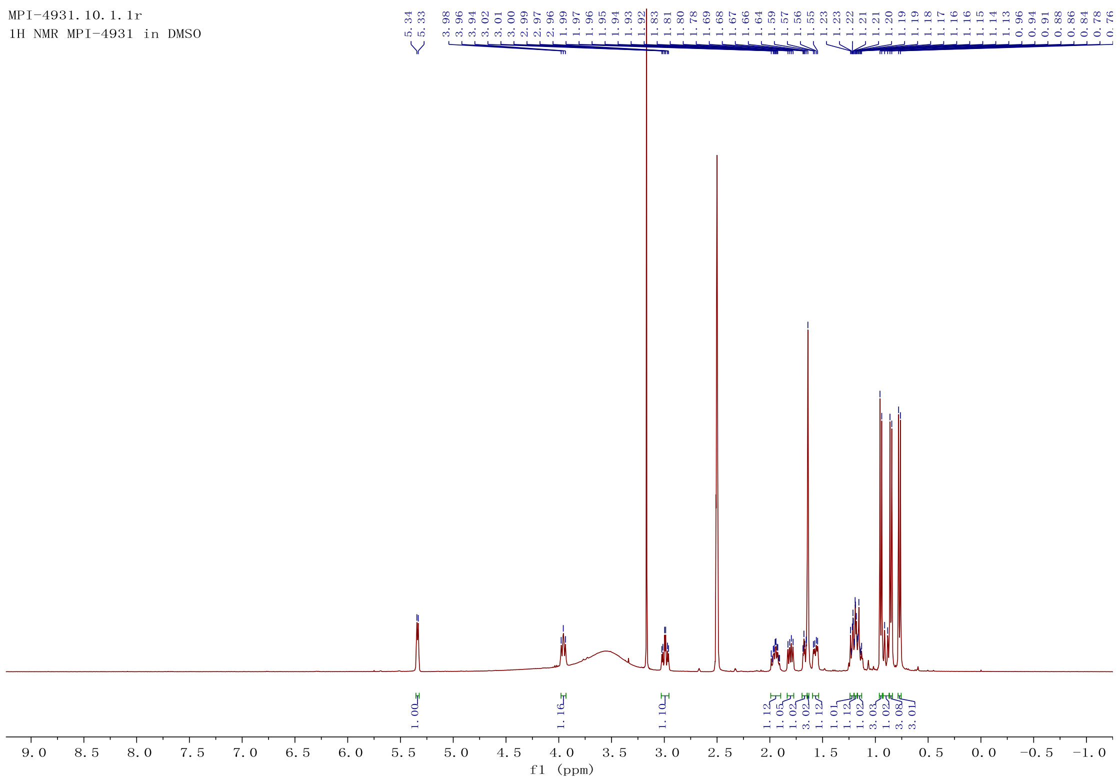

Figure S84. ^{1}H NMR (600 MHz, DMSO-d_6) spectrum of the new compound **23**

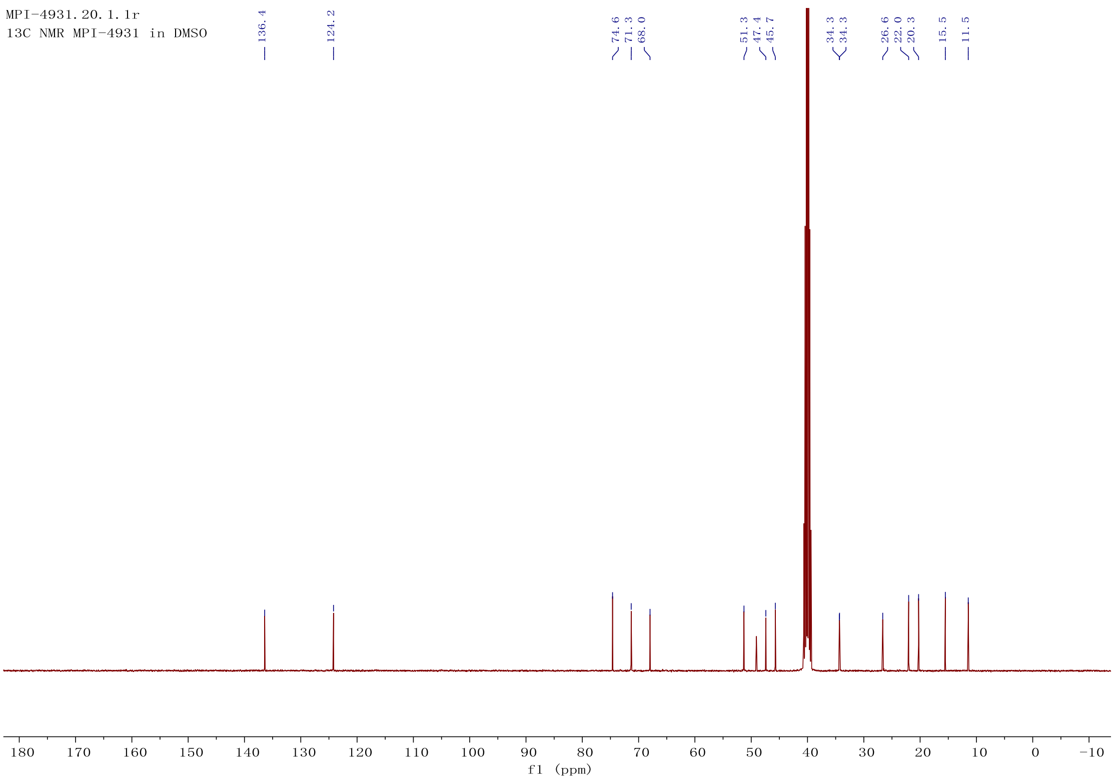

Figure S85. ^{13}C NMR (150 MHz, DMSO-d_6) spectrum of the new compound **23**

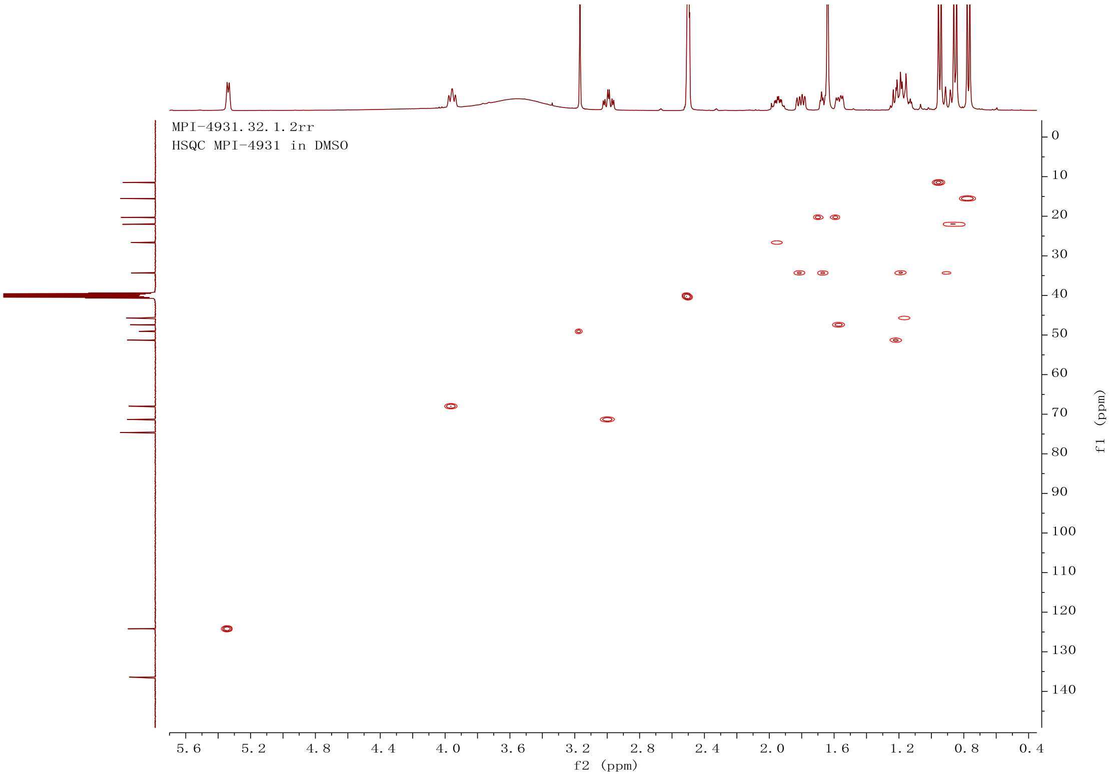

Figure S86. HSQC spectrum of the new compound **23**

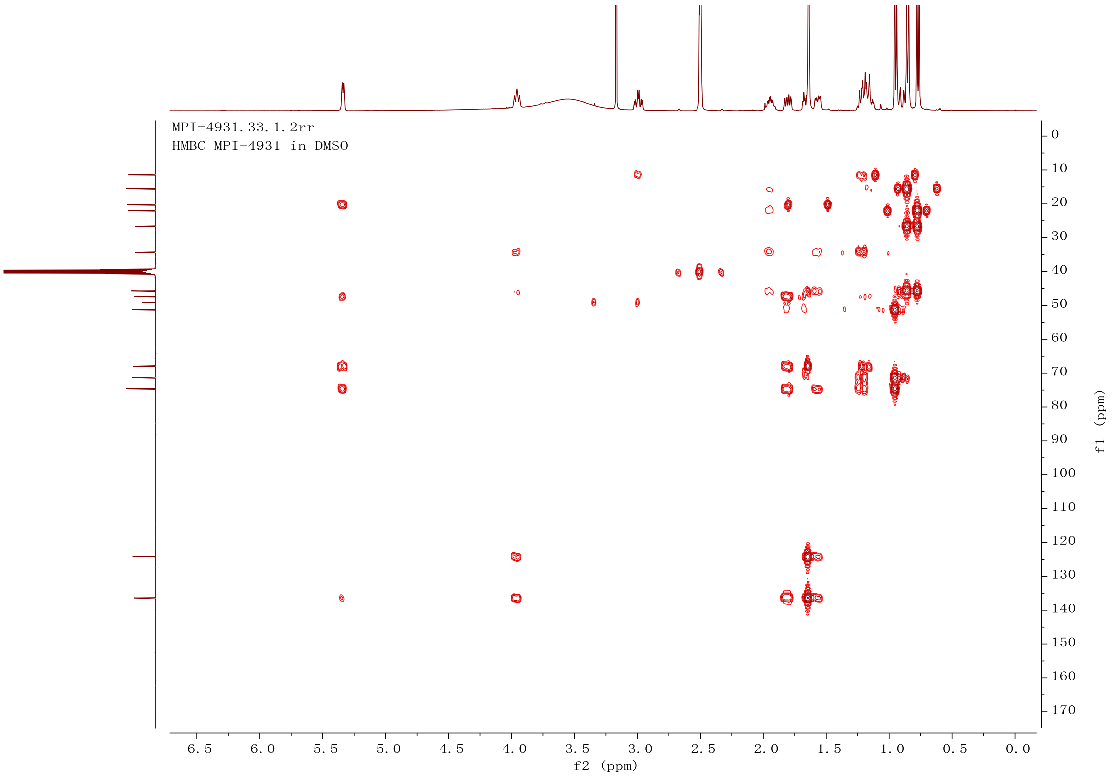

Figure S87. HMBC spectrum of the new compound **23**

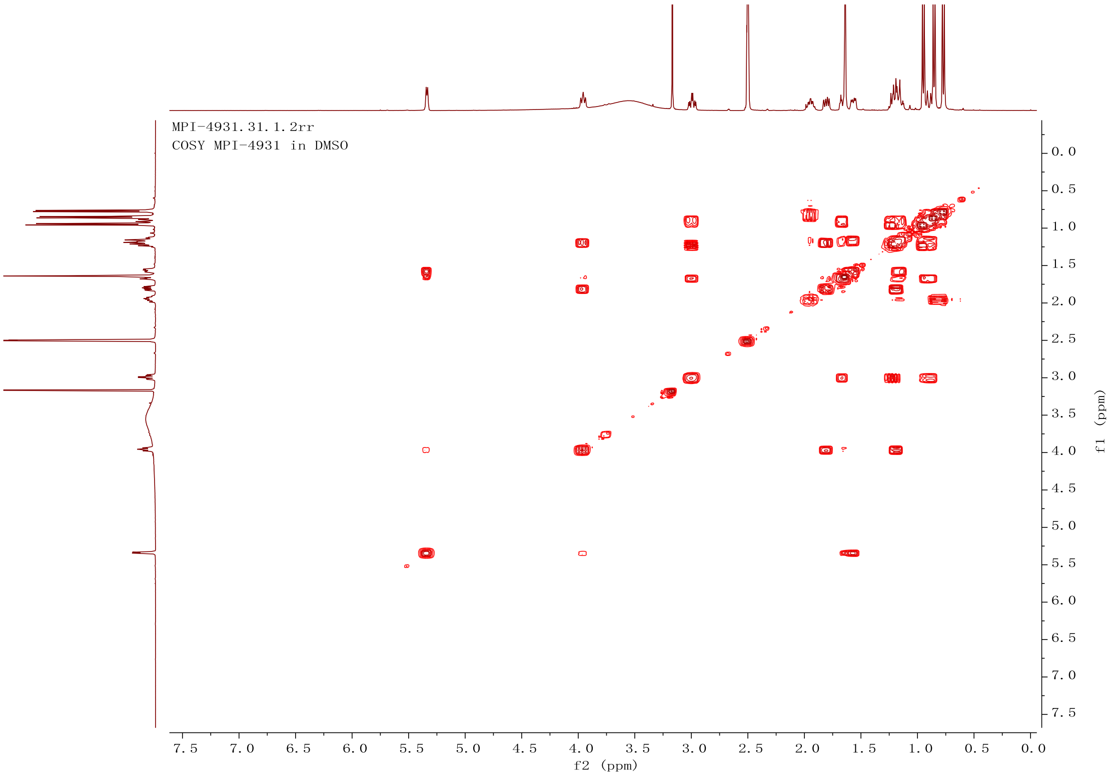

Figure S88. COSY spectrum of the new compound **23**

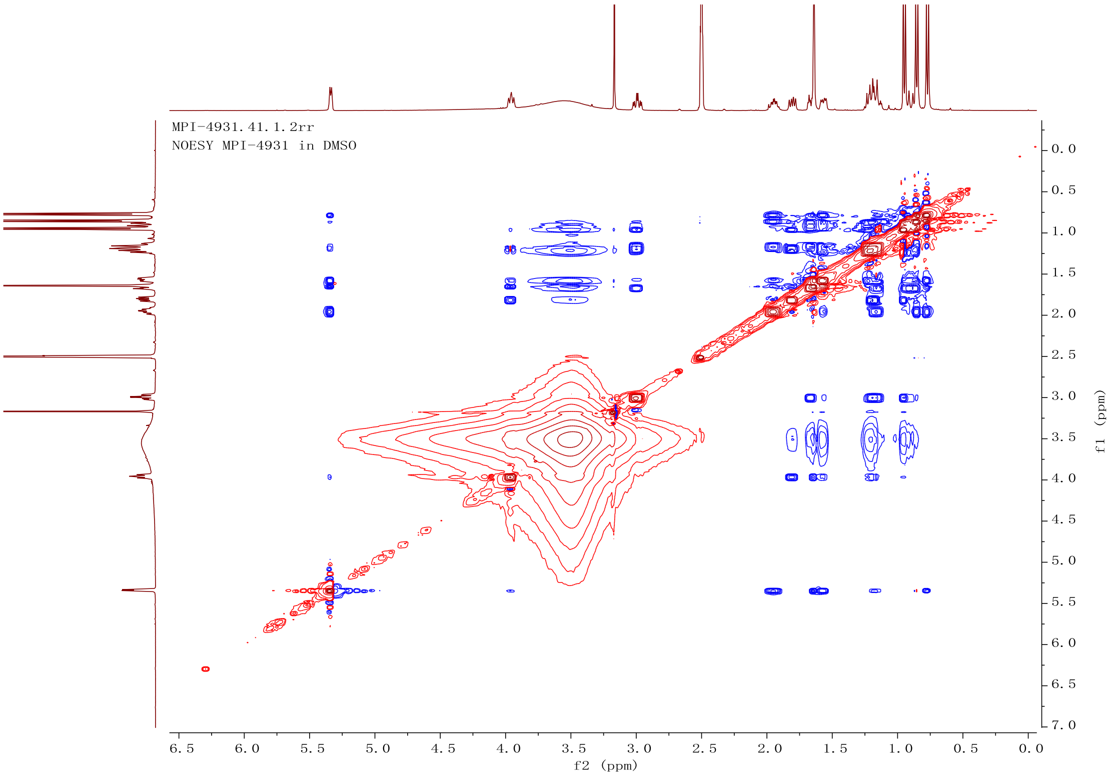

Figure S89. NOESY spectrum of the new compound **23**

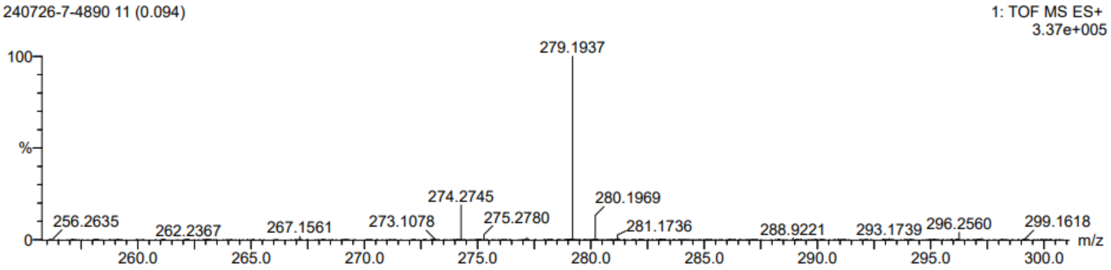

Figure S90. HRESI-MS spectrum of the new compound **25**

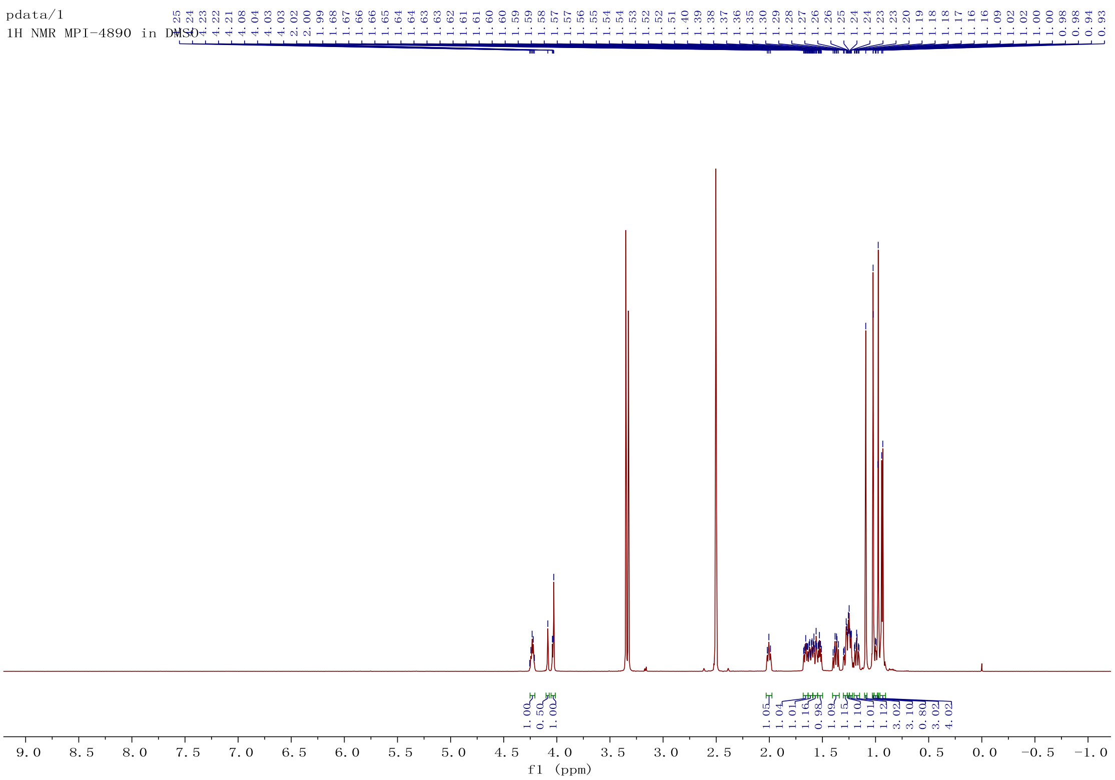

Figure S91. ^{1}H NMR (600 MHz, DMSO-d_6) spectrum of the new compound **25**

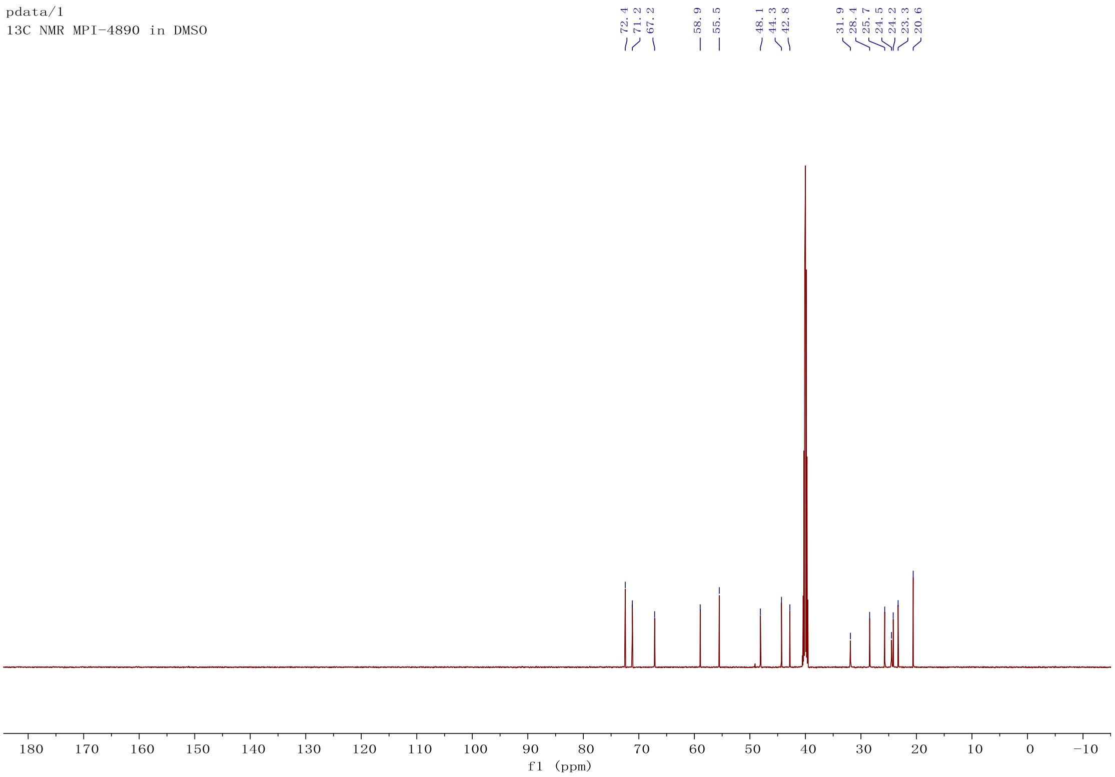

Figure S92. ^{13}C NMR (150 MHz, DMSO-d_6) spectrum of the new compound **25**

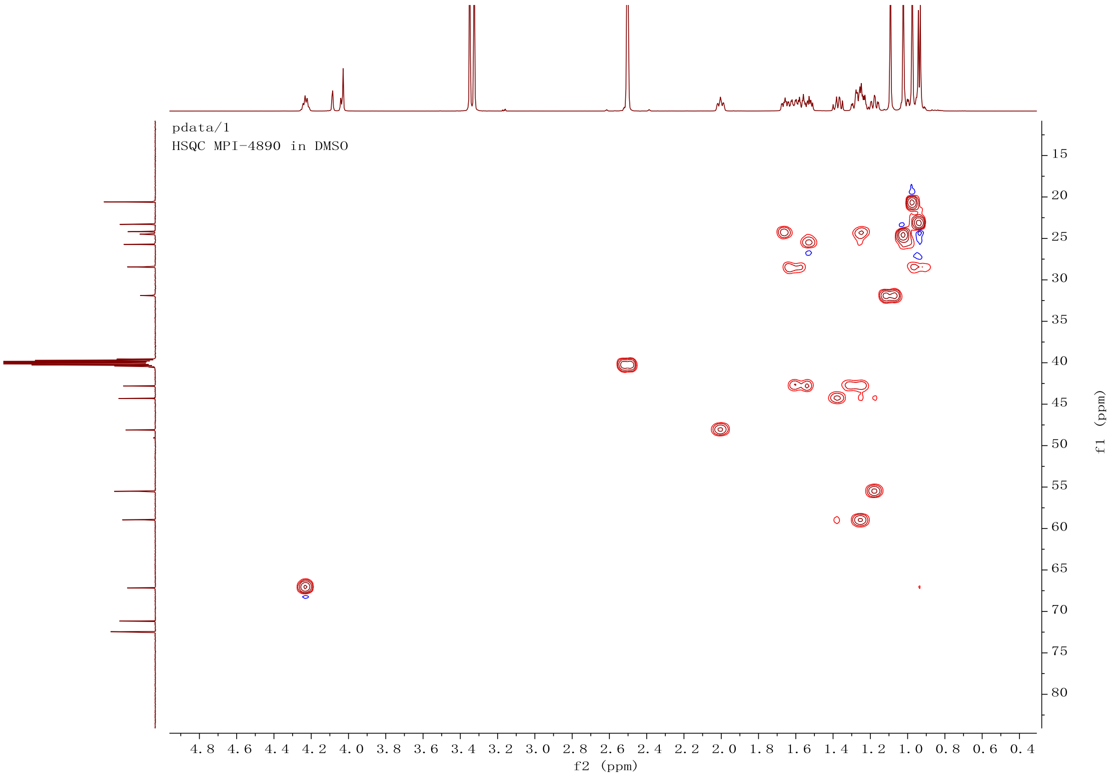

Figure S93. HSQC spectrum of the new compound **25**

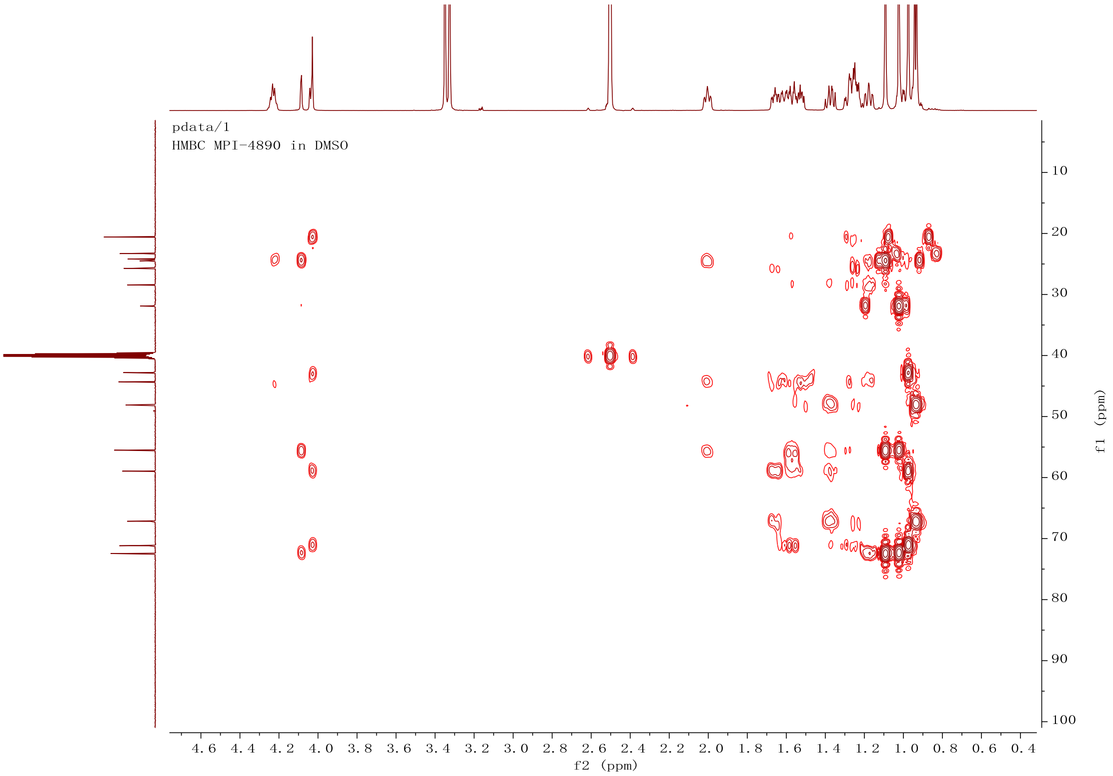

Figure S94. HMBC spectrum of the new compound **25**

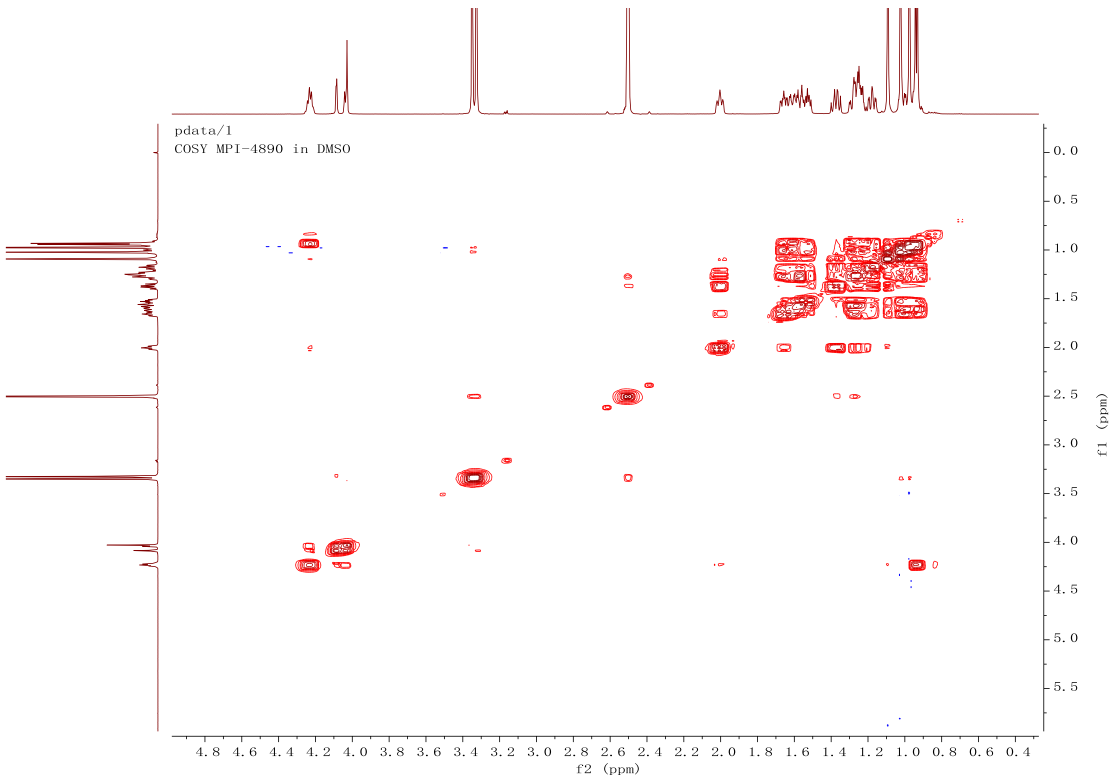

Figure S95. COSY spectrum of the new compound **25**

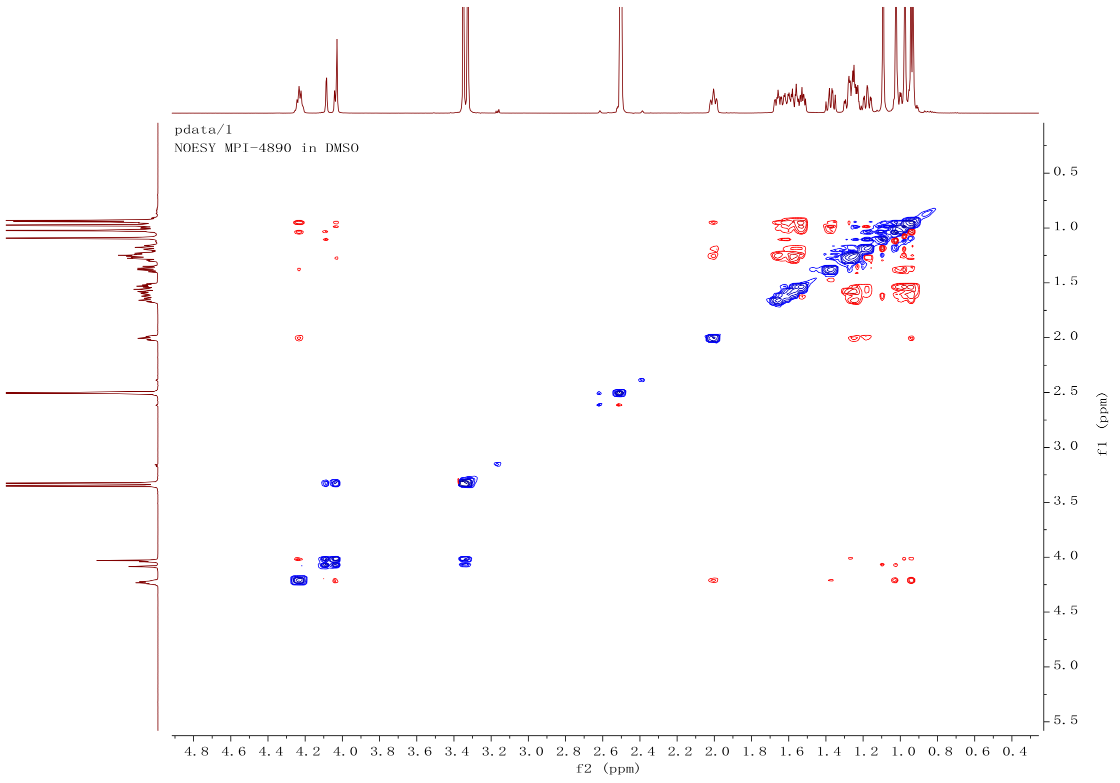

Figure S96. NOESY spectrum of the new compound **25**

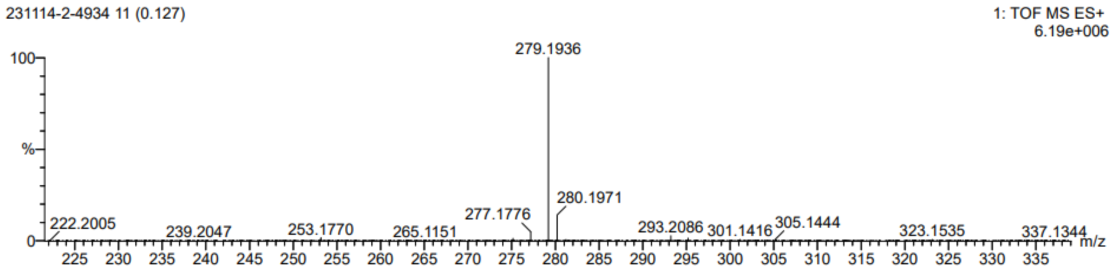

Figure S97. HRESI-MS spectrum of the new compound **26**

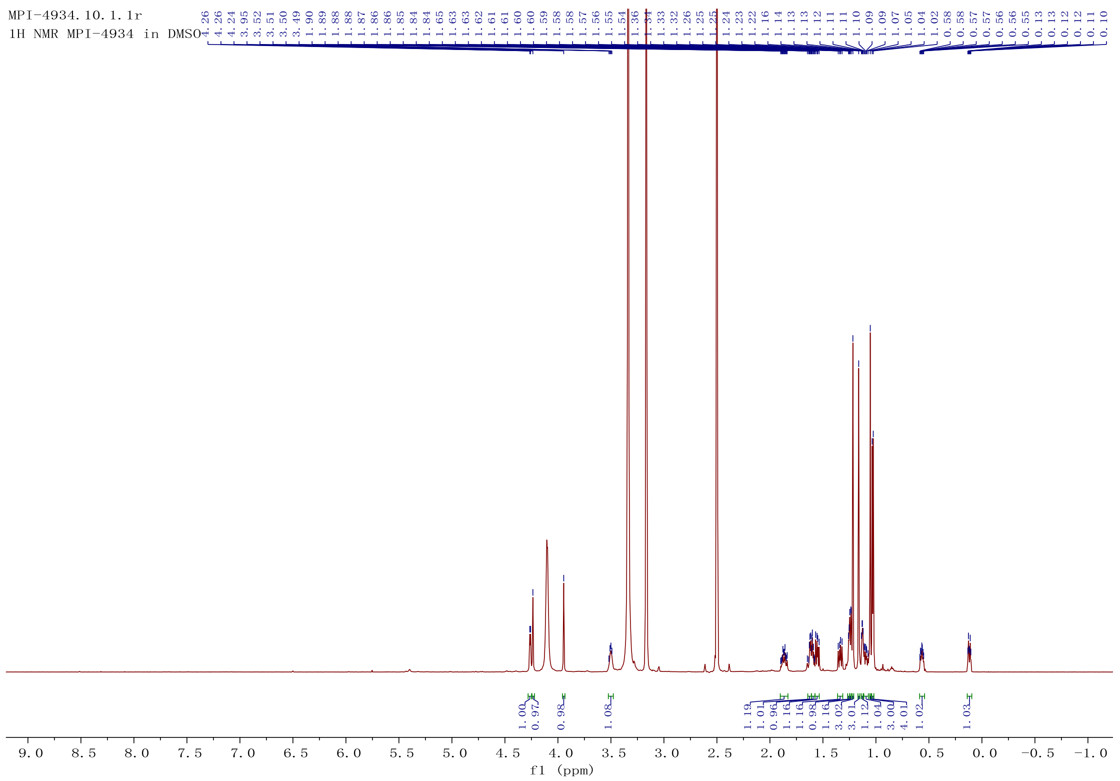

Figure S98. ^{1}H NMR (600 MHz, DMSO-d_6) spectrum of the new compound **26**

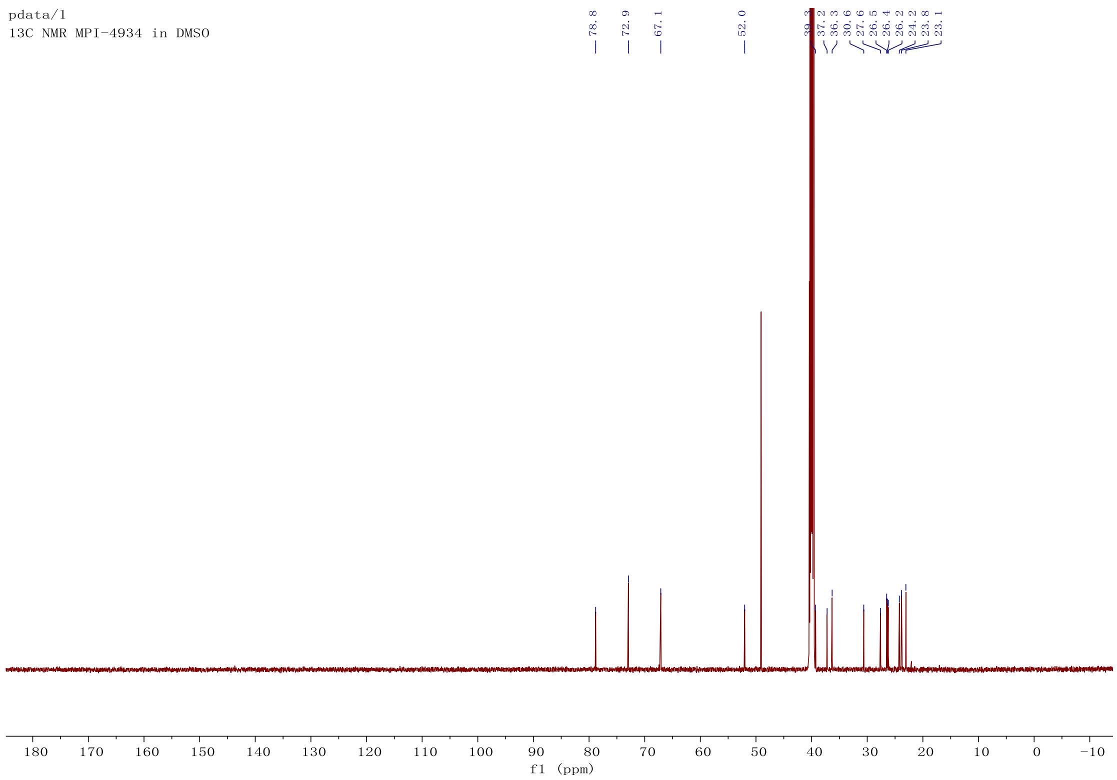

Figure S99. ^{13}C NMR (150 MHz, DMSO-d_6) spectrum of the new compound **26**

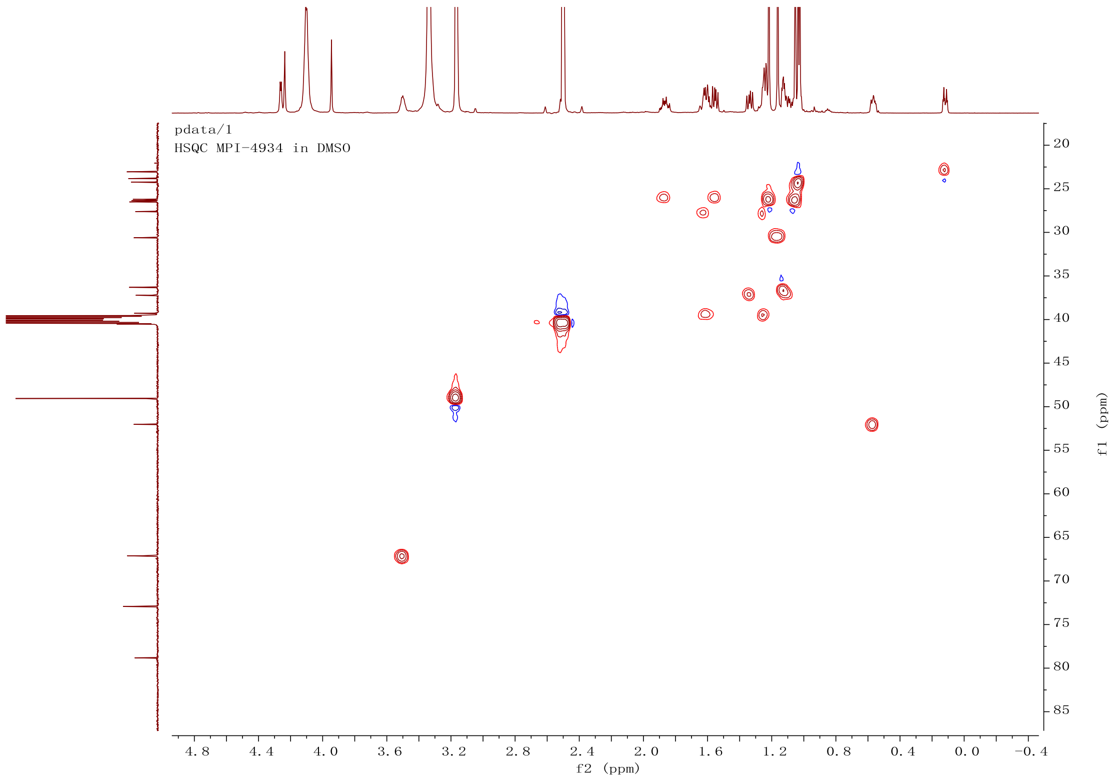

Figure S100. HSQC spectrum of the new compound **26**

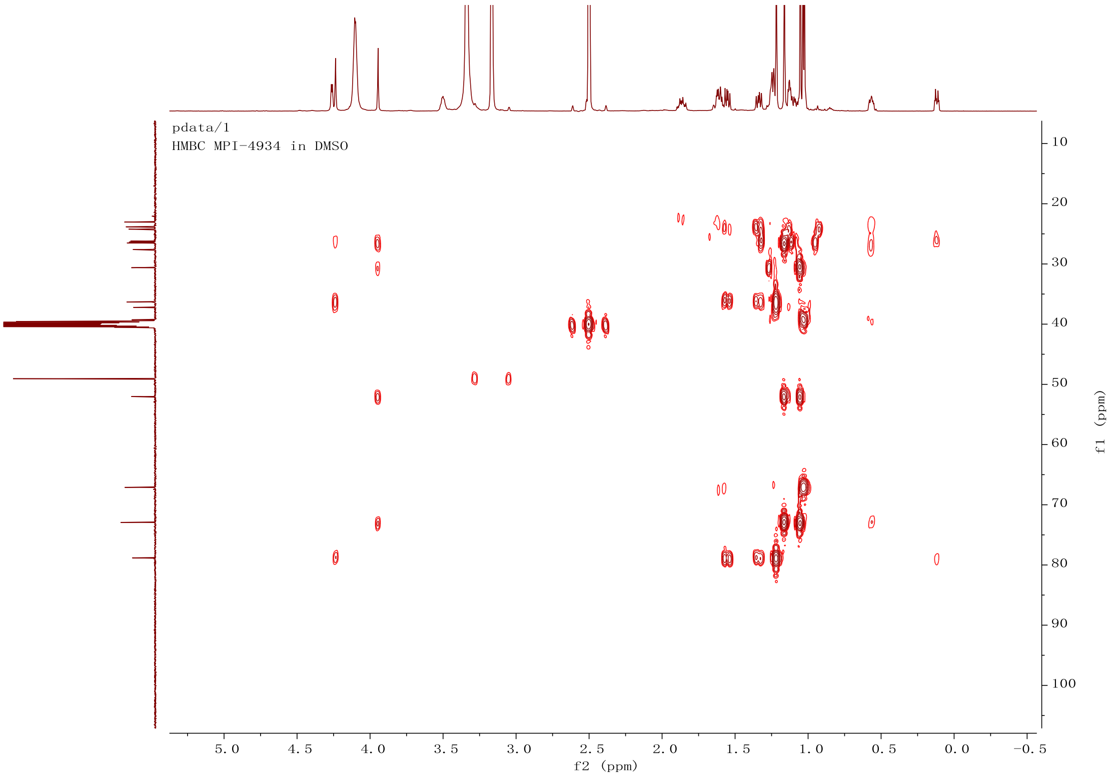

Figure S101. HMBC spectrum of the new compound **26**

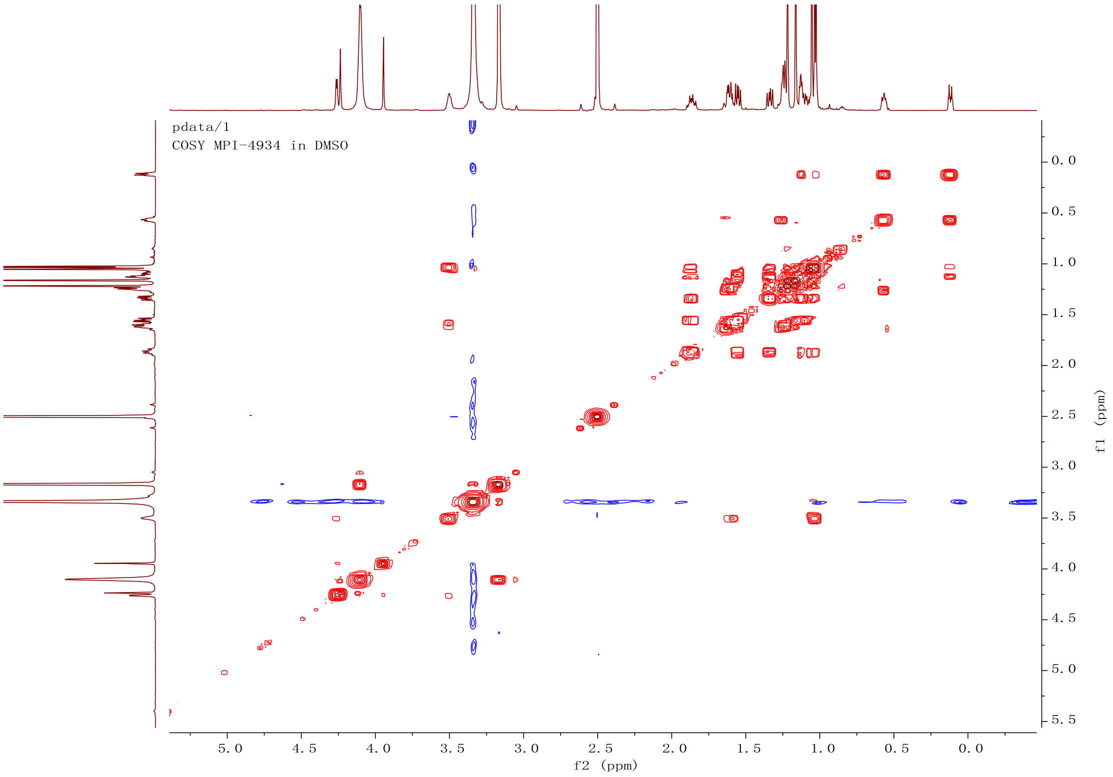

Figure S102. COSY spectrum of the new compound **26**

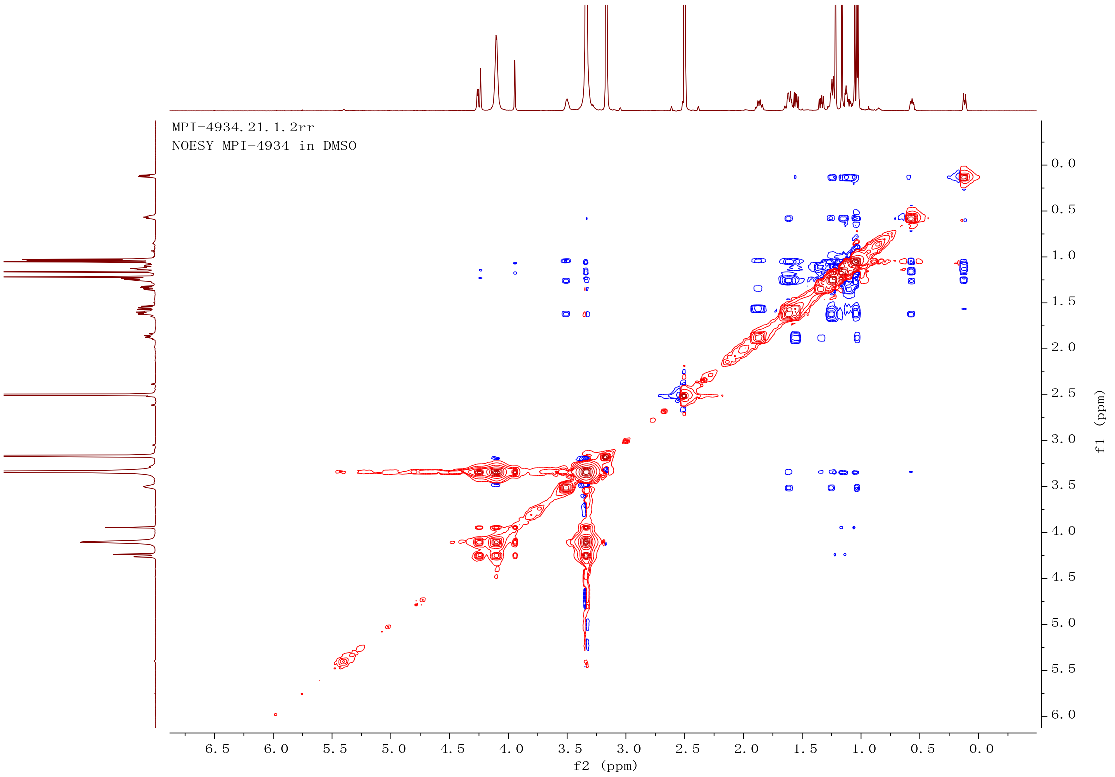

Figure S103. NOESY spectrum of the new compound **26**

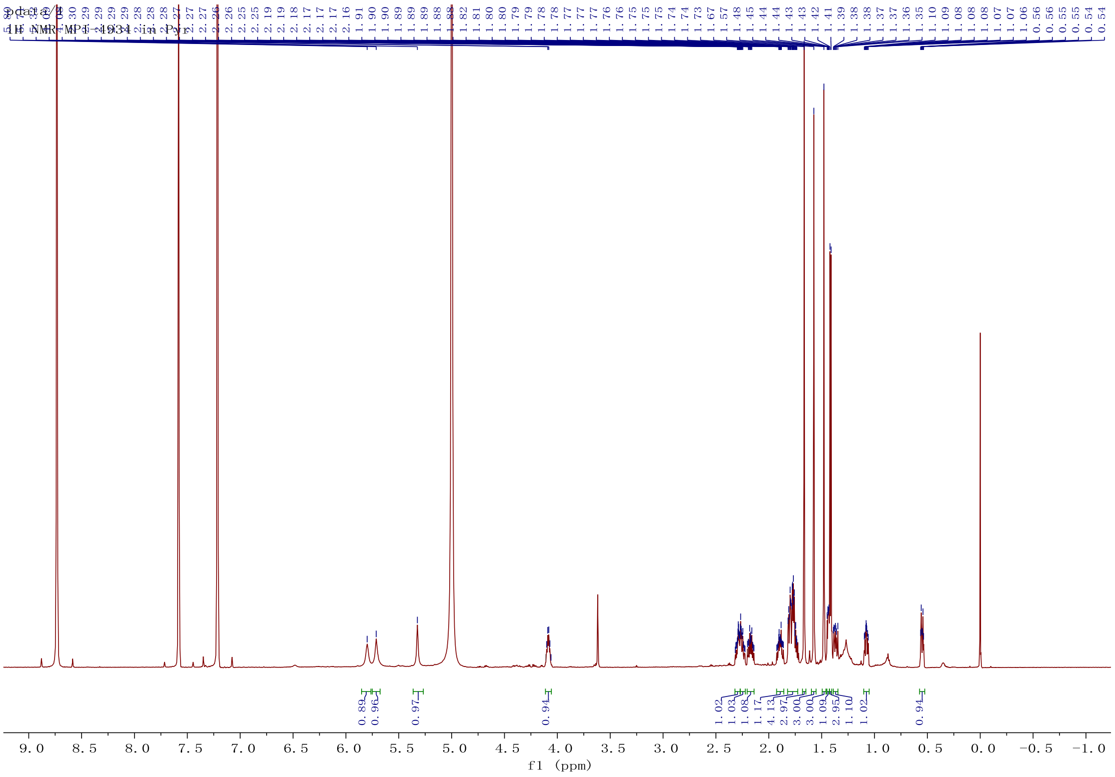

Figure S104. ^{1}H NMR (600 MHz, in pyridine-d_5) spectrum of the new compound **26**

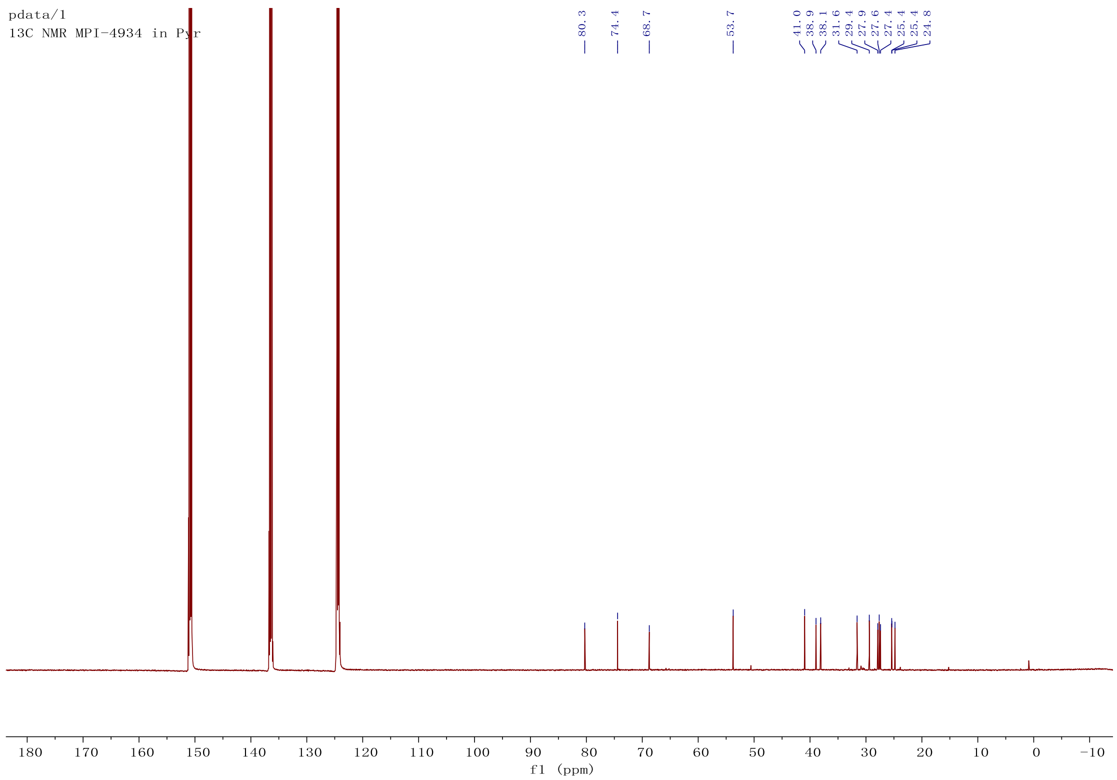

Figure S105. ^{13}C NMR (150 MHz, in pyridine-d_5) spectrum of the new compound **26**

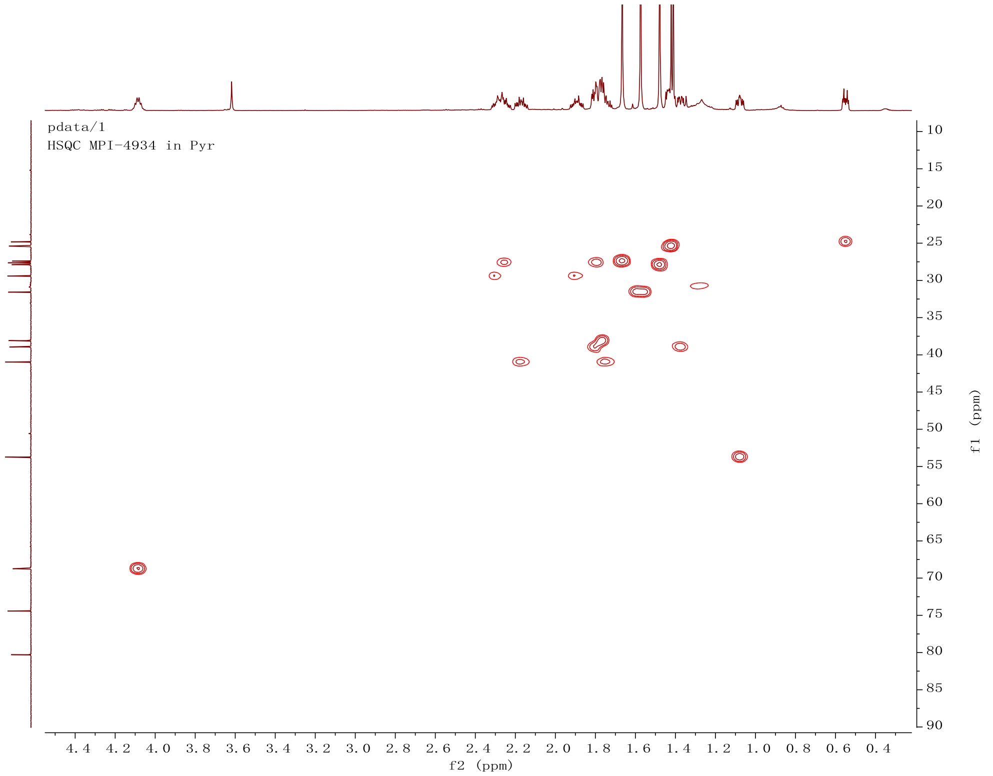

Figure S106. HSQC spectrum of the new compound **26** in pyridine-d_5

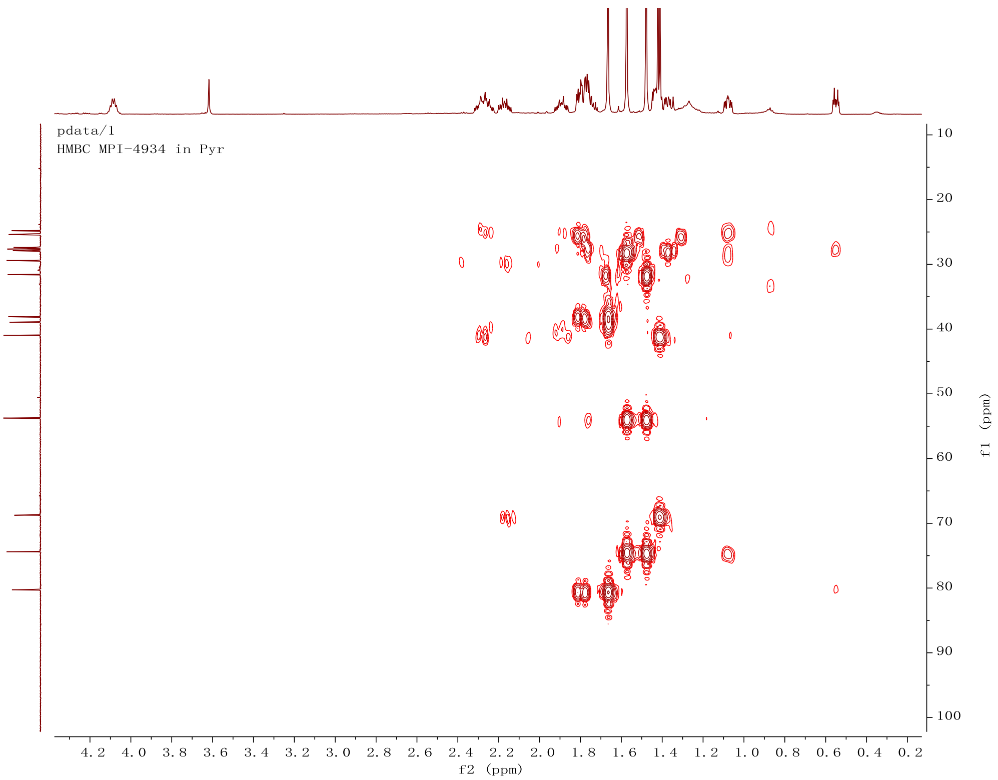

Figure S107. HMBC spectrum of the new compound **26** in pyridine-d_5

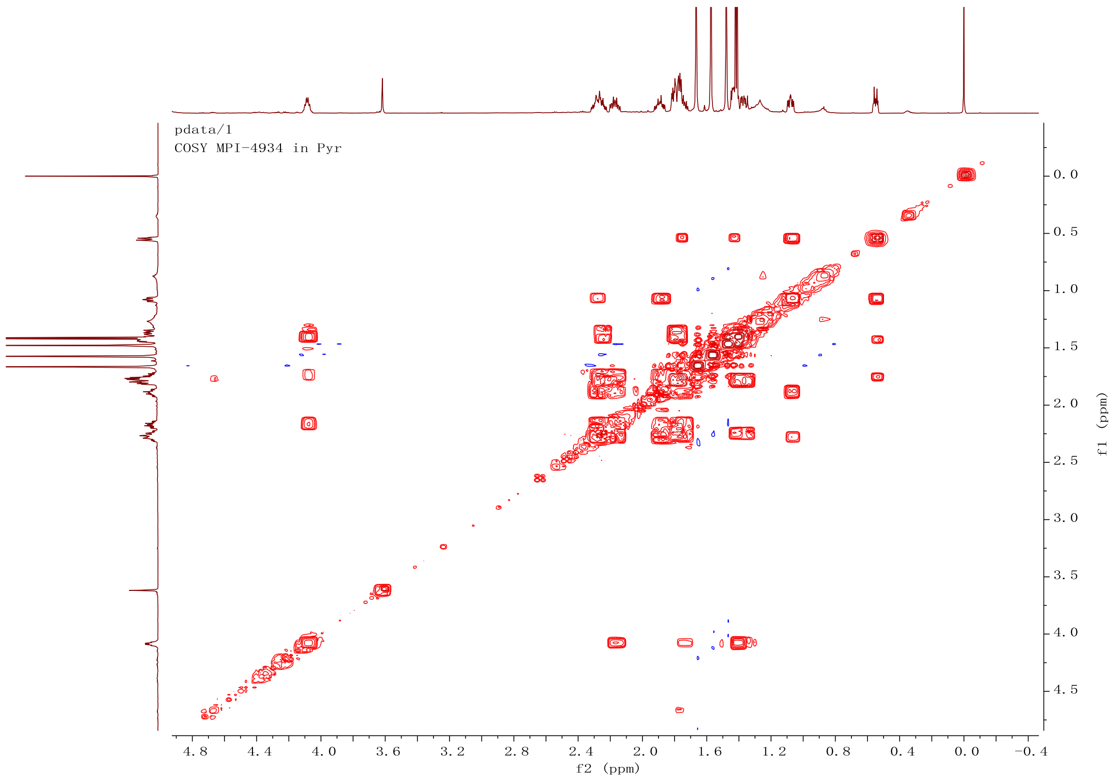

Figure S108. COSY spectrum of the new compound **26** in pyridine-d_5

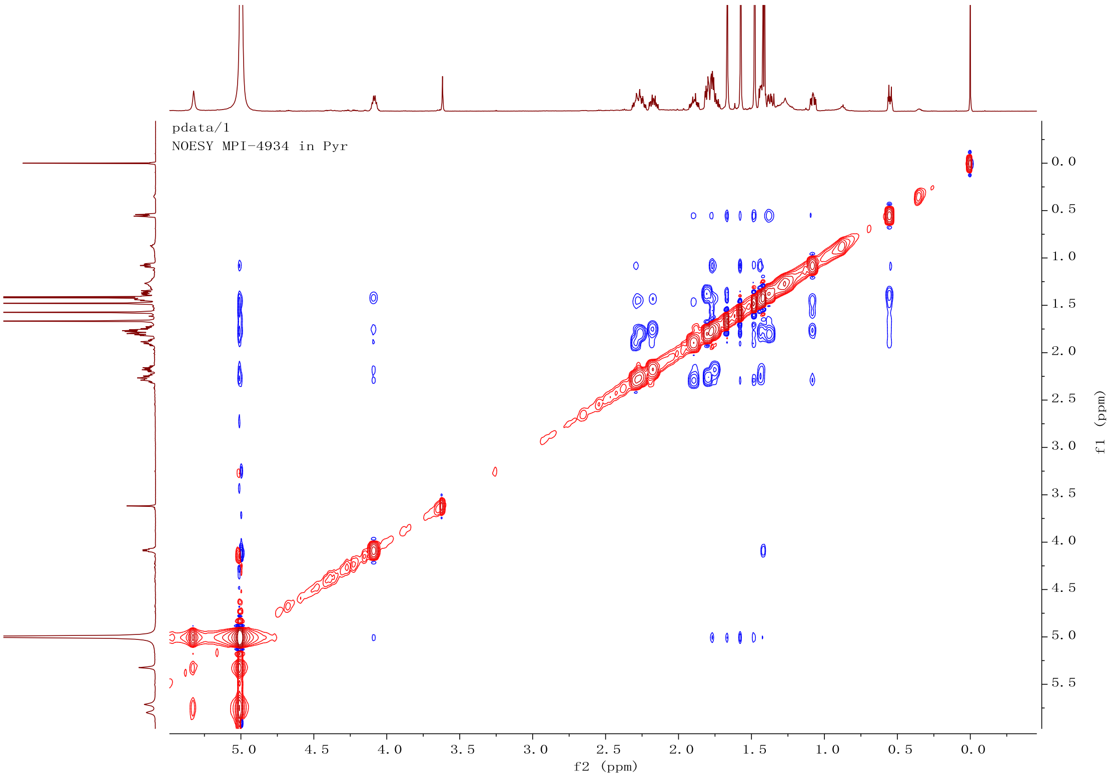

Figure S109. NOESY spectrum of the new compound **26** in pyridine-d_5

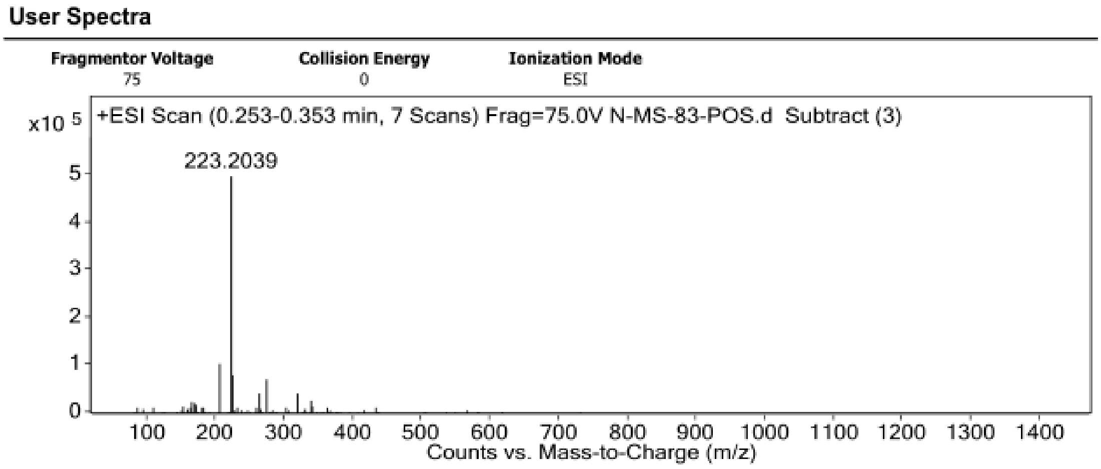

Figure S110. HRESI-MS spectrum of the new compound **27**

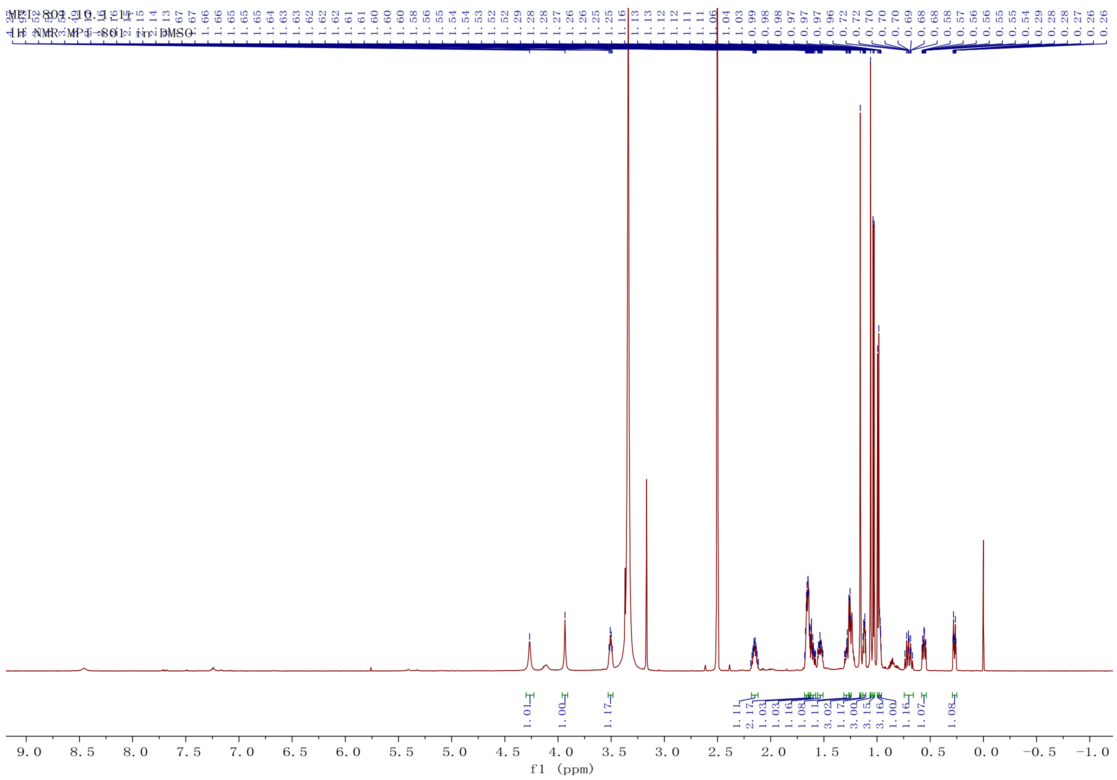

Figure S111. ^{1}H NMR (600 MHz, DMSO-d_6) spectrum of the new compound **27**

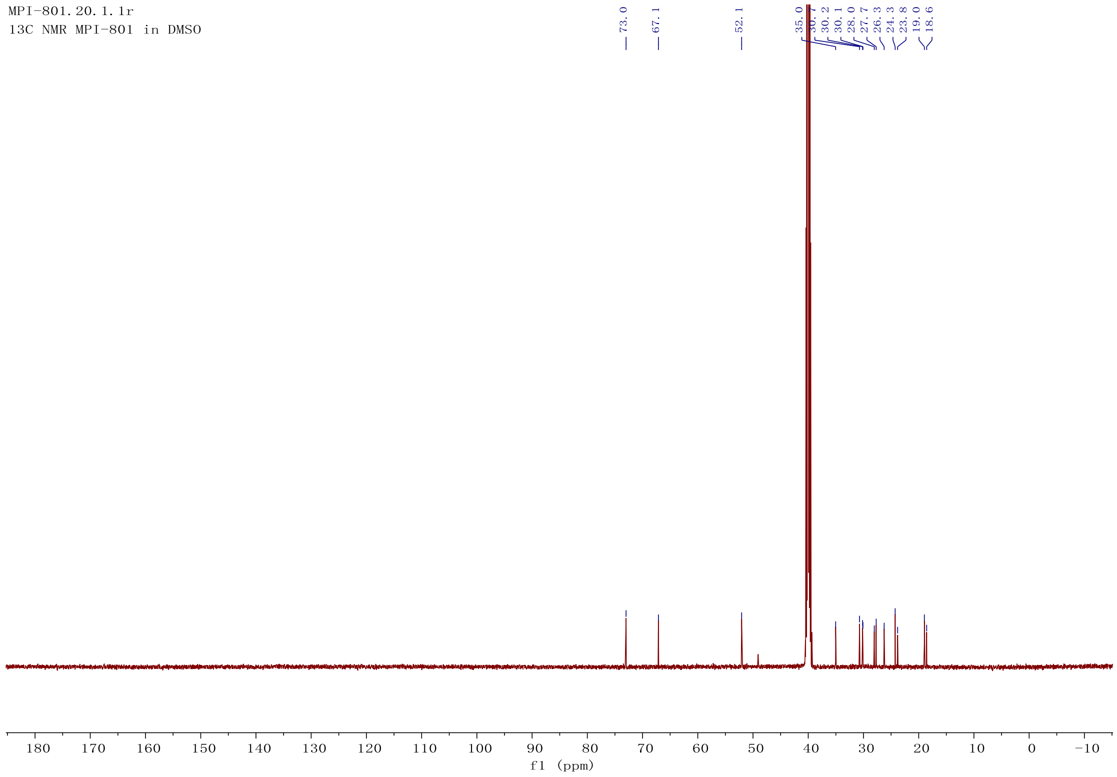

Figure S112. ^{13}C NMR (150 MHz, DMSO-d_6) spectrum of the new compound **27**

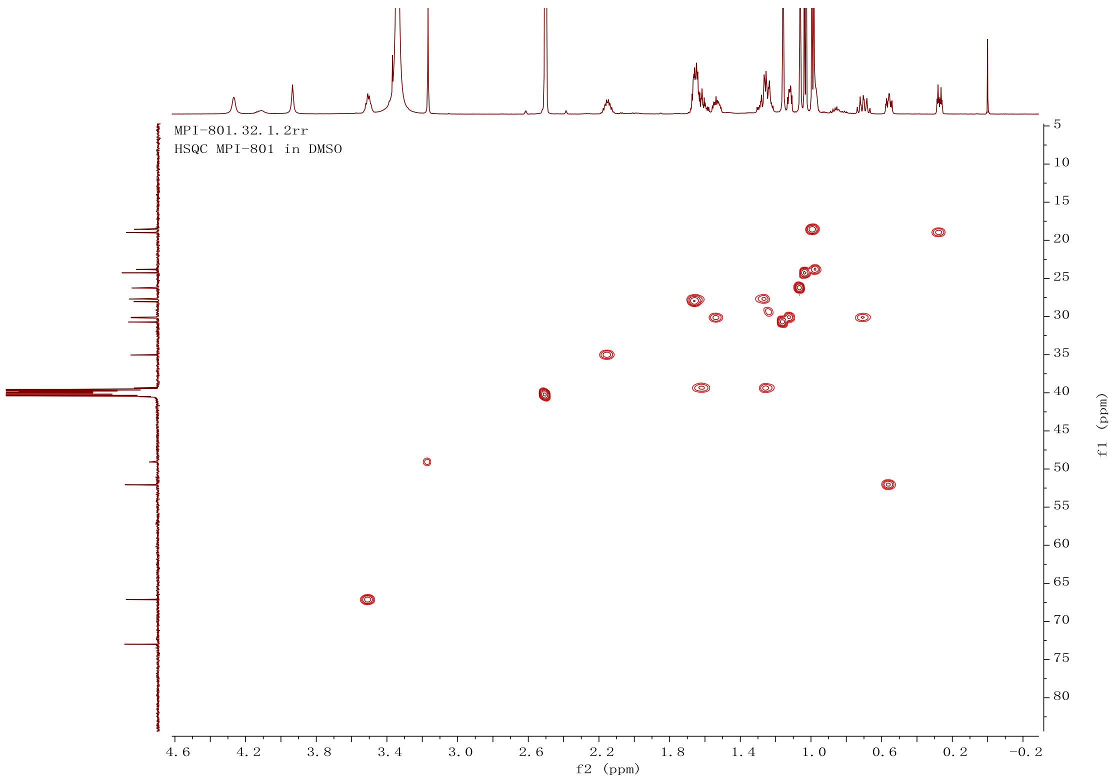

Figure S113. HSQC spectrum of the new compound **27**

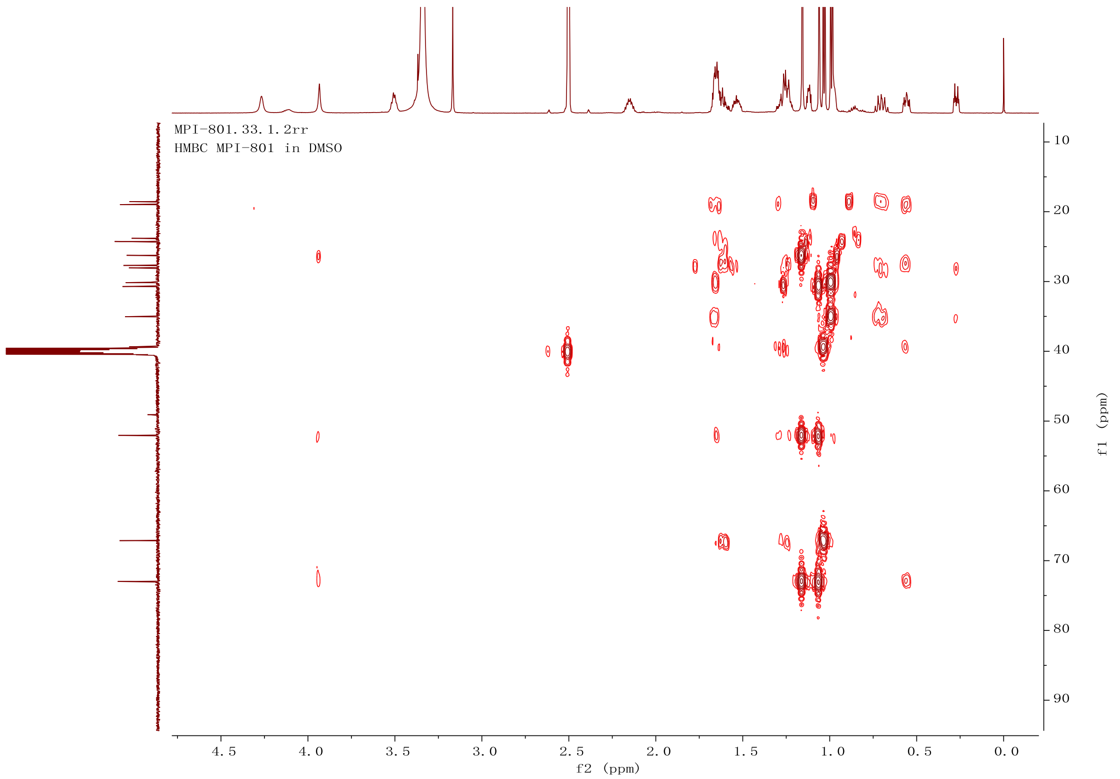

Figure S114. HMBC spectrum of the new compound **27**

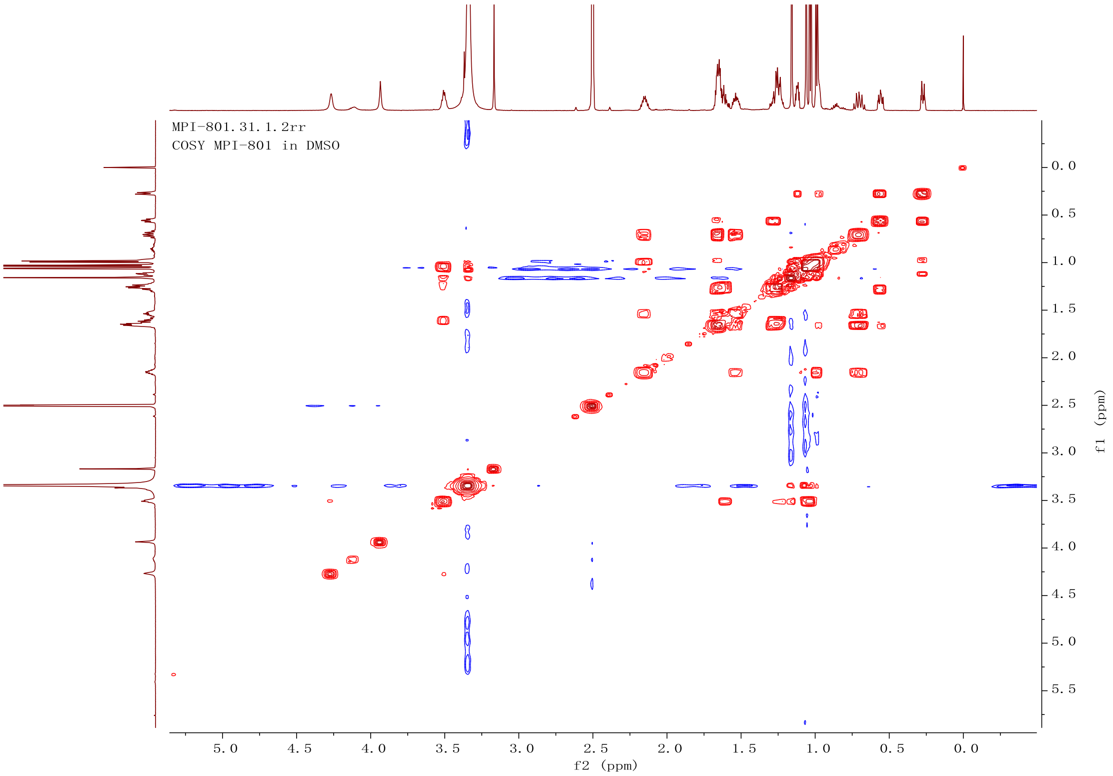

Figure S115. COSY spectrum of the new compound **27**

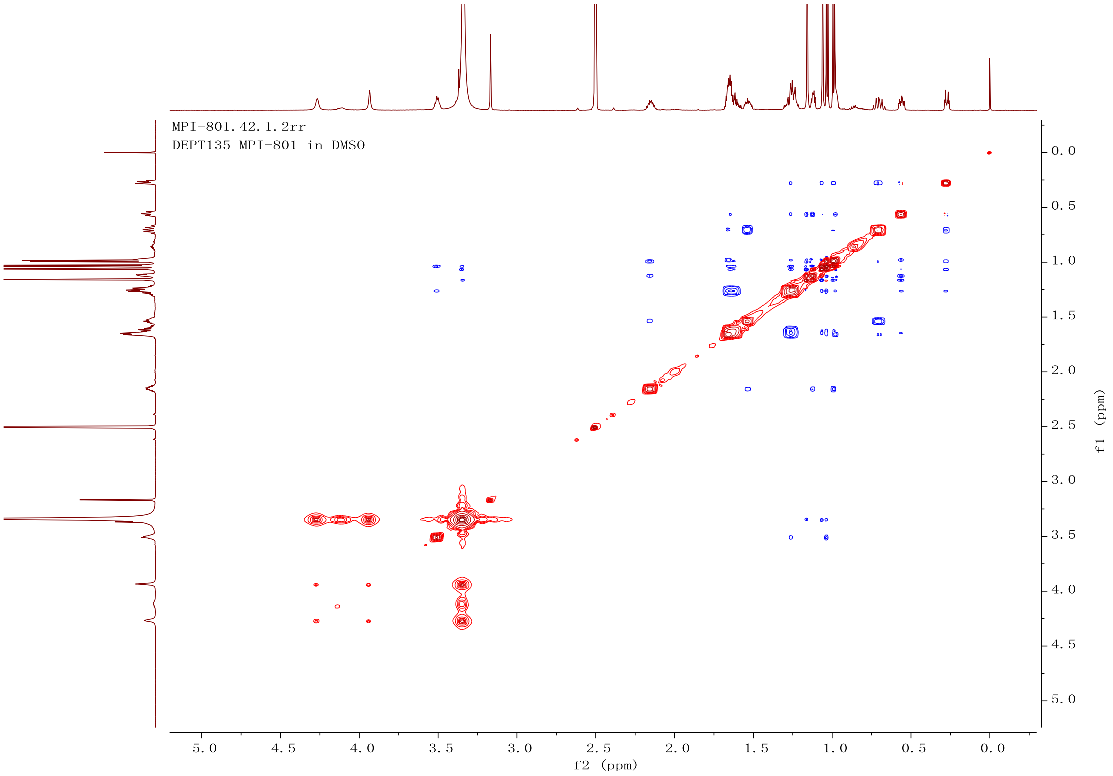

Figure S116. NOESY spectrum of the new compound **27**

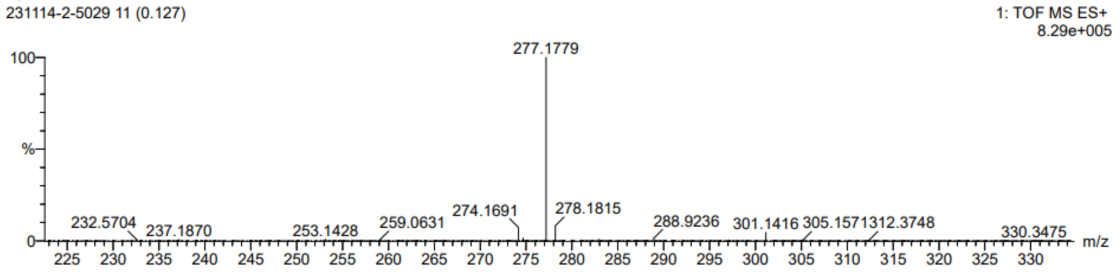

Figure S117. HRESI-MS spectrum of the new compound **28**

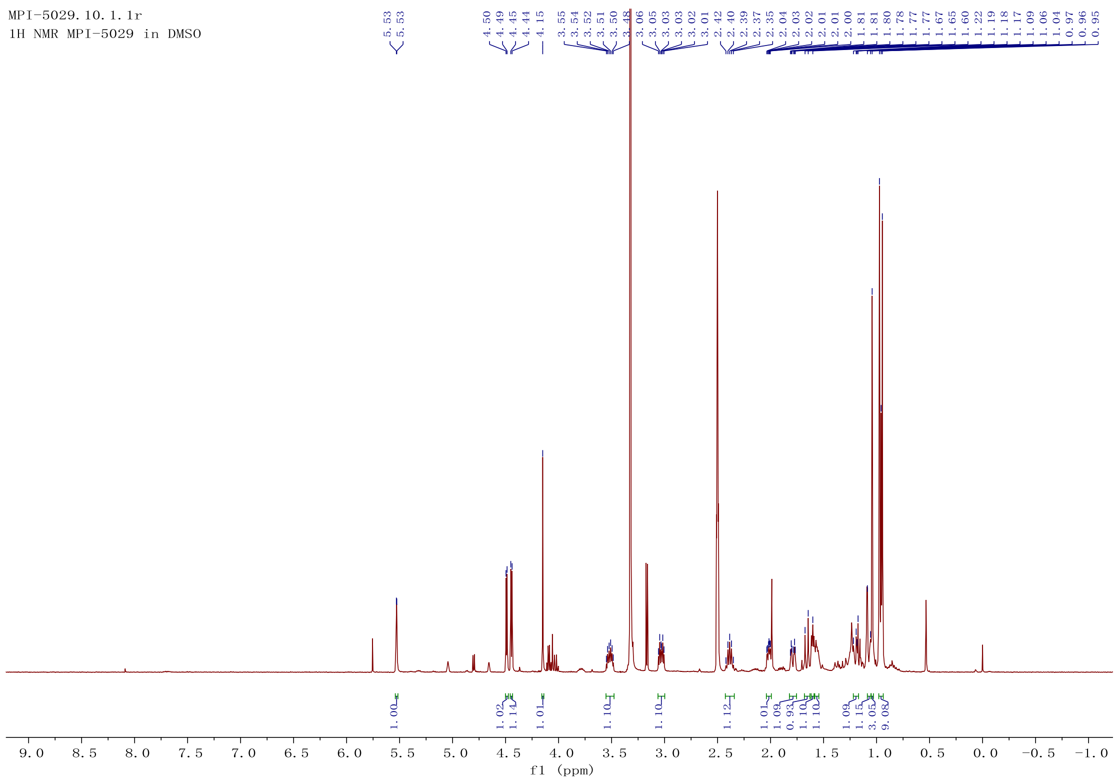

Figure S118. ^{1}H NMR (600 MHz, DMSO-d_6) spectrum of the new compound **28**

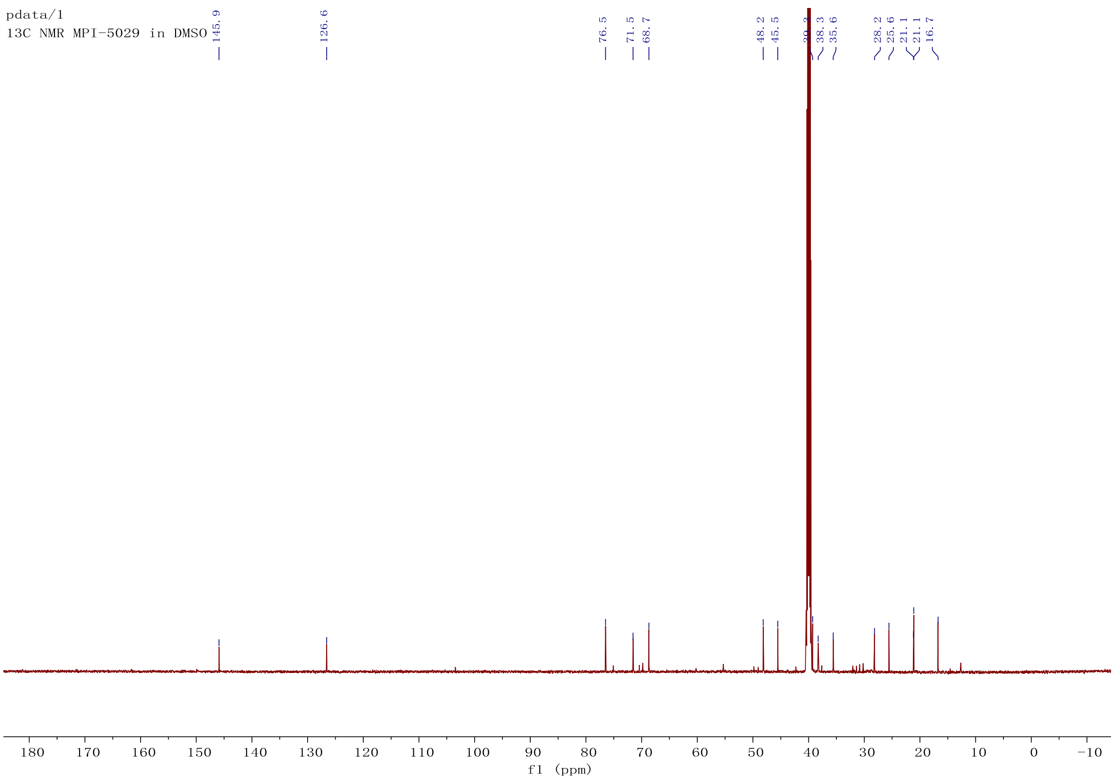

Figure S119. ^{13}C NMR (150 MHz, DMSO-d_6) spectrum of the new compound **28**

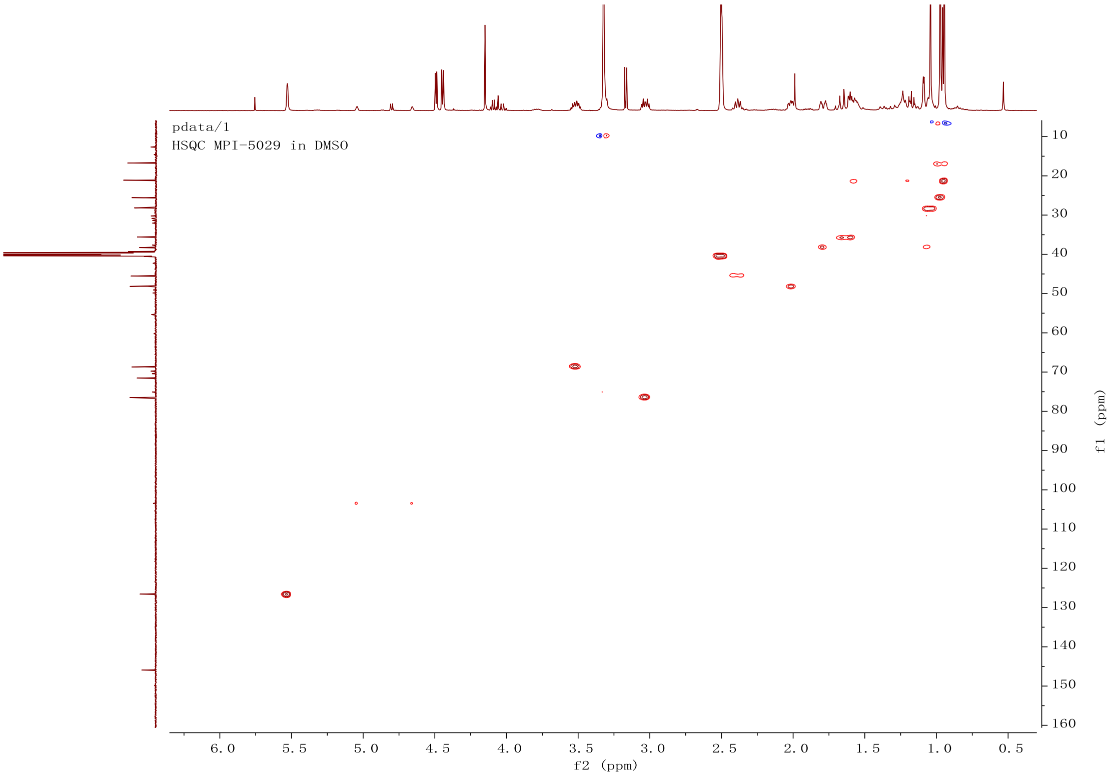

Figure S120. HSQC spectrum of the new compound **28**

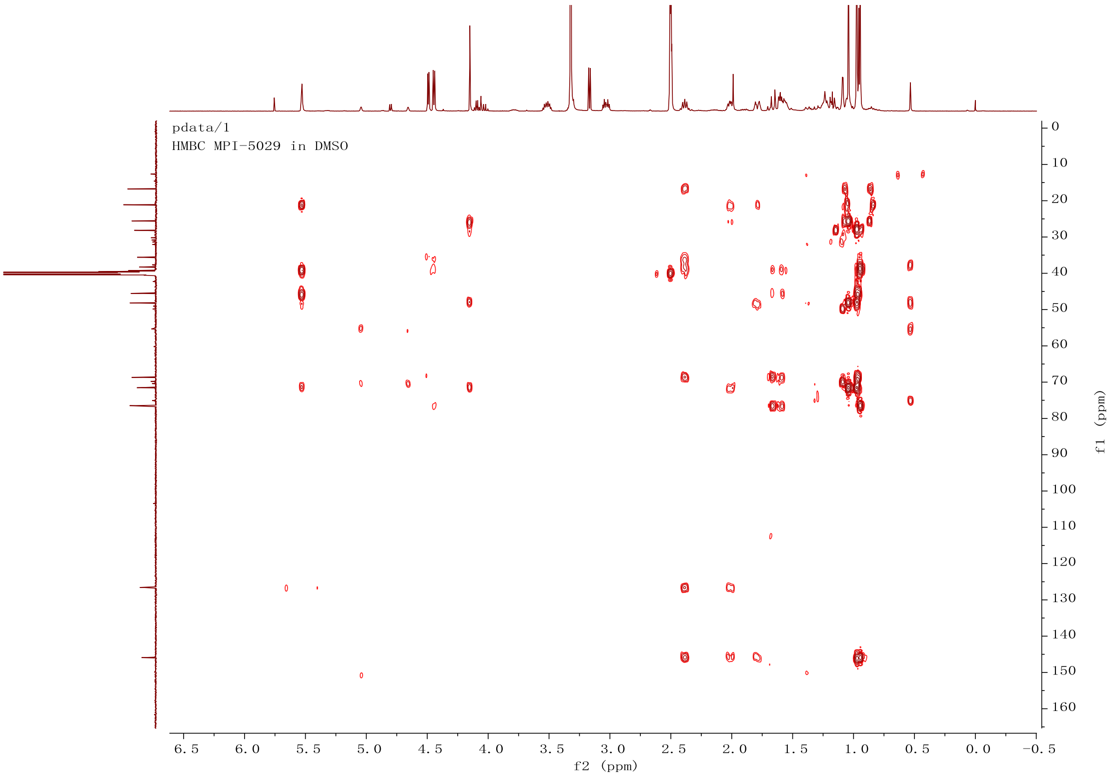

Figure S121. HMBC spectrum of the new compound **28**

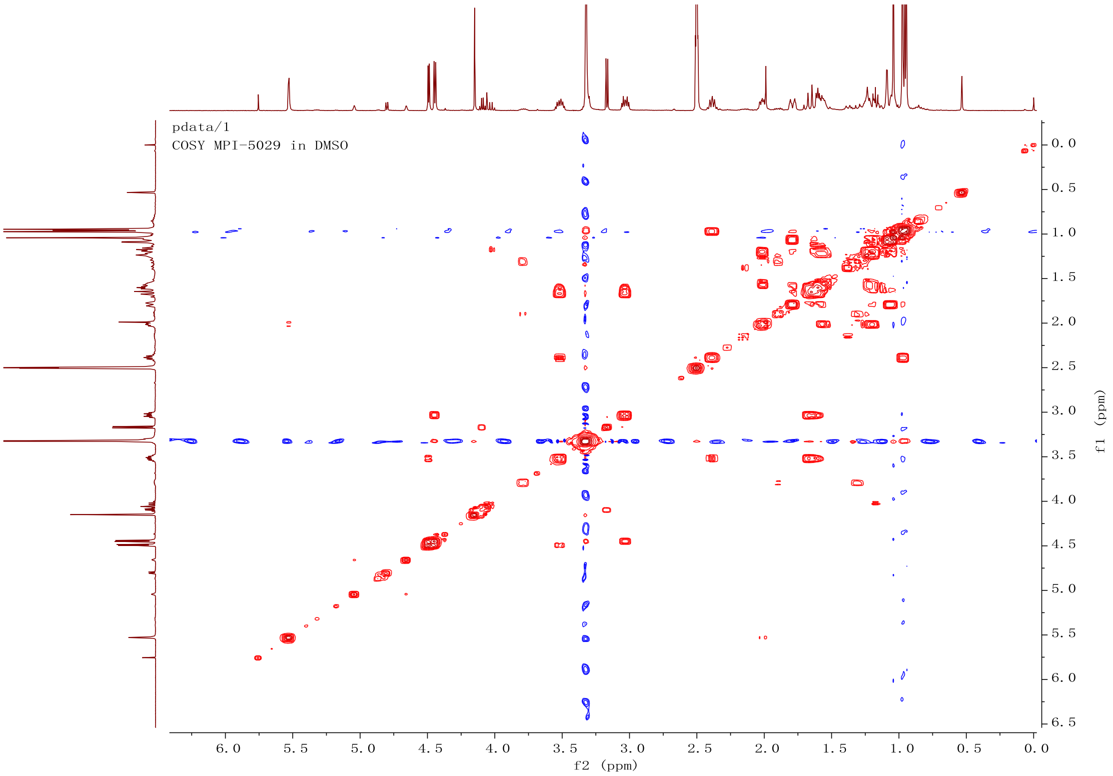

Figure S122. COSY spectrum of the new compound **28**

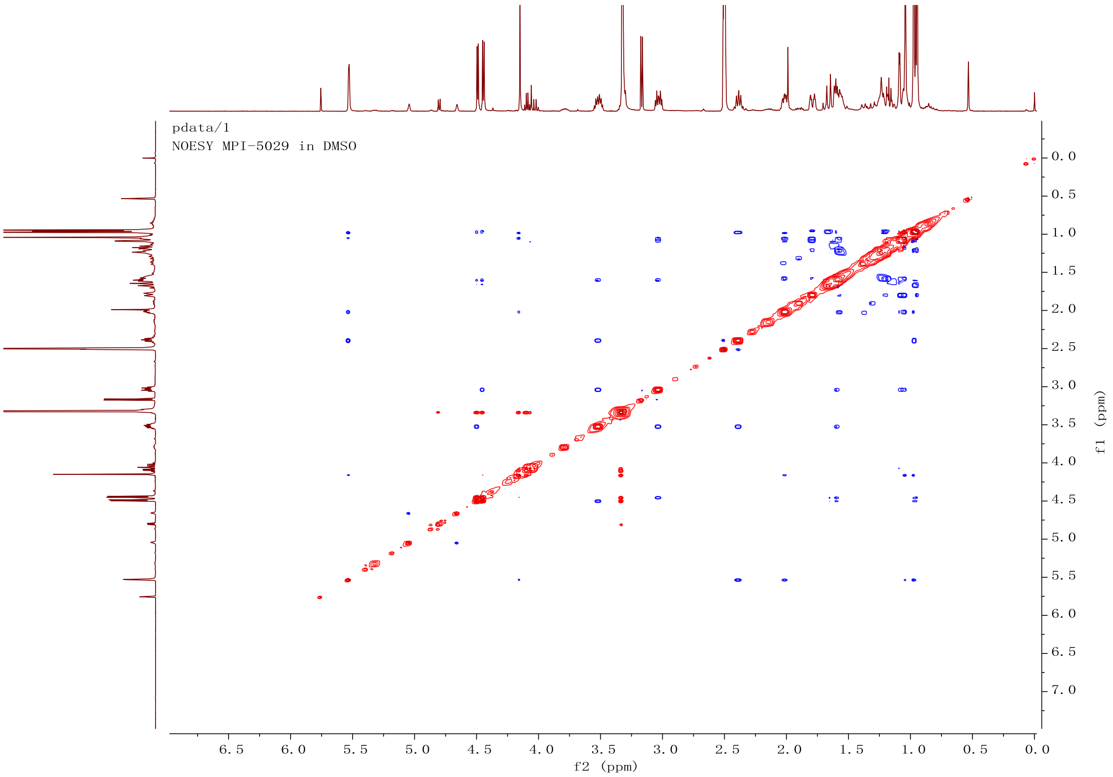

Figure S123. NOESY spectrum of the new compound **28**

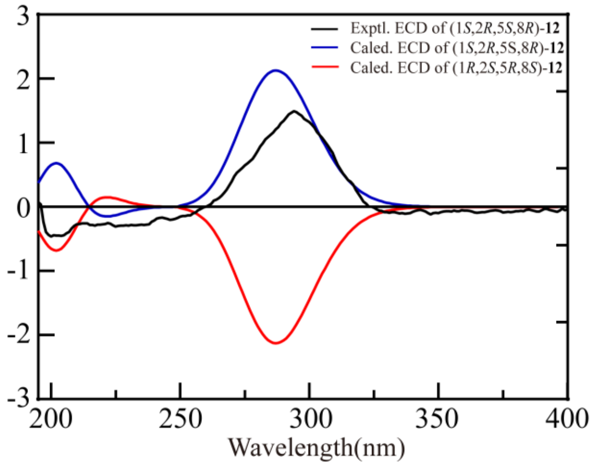

Figure S124. Experimental ECD spectra and calculated ECD spectra of compound **12**
